# Introducing Small Rings into Farnesyl Pyrophosphates Paves the Way for the Enzymatic Generation of Unnatural Sesquiterpene Scaffolds

**DOI:** 10.1021/jacs.5c19651

**Published:** 2026-01-30

**Authors:** Daghan Taser, Catherine Victoria, Leon von Garrel, Jörn Droste, Christopher Tabet, Gerald Dräger, Ahmed Hassanin, Mehdi D. Davari, Andreas Kirschning

**Affiliations:** † Institute of Organic Chemistry, 26555 Leibniz University Hannover, Schneiderberg 1B, 30167 Hannover, Germany; ‡ Department of Bioorganic Chemistry, Leibniz Institute of Plant Biochemistry (IPB), 06120 Halle, Germany; § Department of Pharmacognosy, Faculty of Pharmacy, Assiut University, 71526 Assiut, Egypt; ∥ Uppsala Biomedical Center (BMC), University Uppsala, Husargatan 3, 75237 Uppsala, Sweden

## Abstract

New sesquiterpene skeletons are accessible when the geminal dimethyl group in farnesyl pyrophosphate (FPP) is exchanged by small strained rings, specifically cyclopropane, cyclobutane, and oxetane. When these new FPP derivatives are exposed to sesquiterpene synthases, the additional chemical reactivity installed in the strained rings can interact in a unique way with the carbocation intermediates in the active centers of sesquiterpene synthases BcBOT2, PenA, Omp7, and Cop4, which are known to be substrate promiscuous. As such, they can induce rearrangements and ring enlargements, which can yield completely new, previously unknown sesquiterpene carbon skeletons with additional carbon atoms embedded in the (oligo)­cyclic backbones. A total of 17 new terpenoids are reported and structurally elucidated, 11 of which have so far unknown unnatural terpene backbones. Besides rearrangements of the small rings, we report on the nucleophilic involvement of the oxygen atom in the oxetane ring during the initial cyclization step. As an additional finding, the oxetane analogues of the two known sesquiterpenes africanene and pentalenene were isolated. Molecular modeling studies revealed that the FPP derivatives are optimally oriented for catalysis within the enzymes’ active sites. The simulations unveiled alternative binding poses that facilitate divergent cyclization cascades, ultimately leading to the formation of previously uncharacterized molecular frameworks of sesquiterpenes.

## Graphical abstract

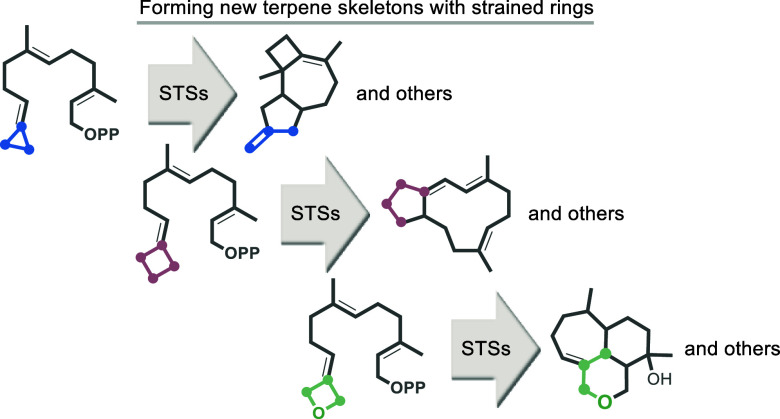

## Introduction

Chemically, the strained cyclopropane ring (strain energy = 28.1 kcal/mol) can be considered a homoalkene group, showing similar but reduced π-reactivity compared to alkenes.^1^ The formation of a carbocation adjacent to the cyclopropyl ring leads to a rapid equilibrium between the cyclopropylmethyl **B**/**D**, the homoallyl **A**, and the cyclobutyl cations **C** (Scheme 1).^2^ Cyclopropylcarbinyl cations are significantly more stable as long as a bent conformation can be adopted. Here, the Walsh orbitals are able to stabilize the cation via hyperconjugation, with primary carbocations included. Nature has utilized this special feature of carbocation chemistry in several ways, particularly in terpene biosynthesis.^3^ For example, the two-step process of head-to-head dimerization of farnesyl pyrophosphate (FPP, **1**) to squalene by squalene synthase (SQS) supposedly proceeds through a cyclopropane intermediate which undergoes a ring expansion to a second intermediate, a cyclobutyl cation **C** (Scheme 1).^4^ Theoretical calculations by Tantillo et al. in cyclopropane-to-cyclopropane rearrangements (**B** ↔ **D**) of sterols indicate that strongly delocalized bicyclobutonium ions **E** could be involved in their biosynthesis.^5^

**Scheme 1:**
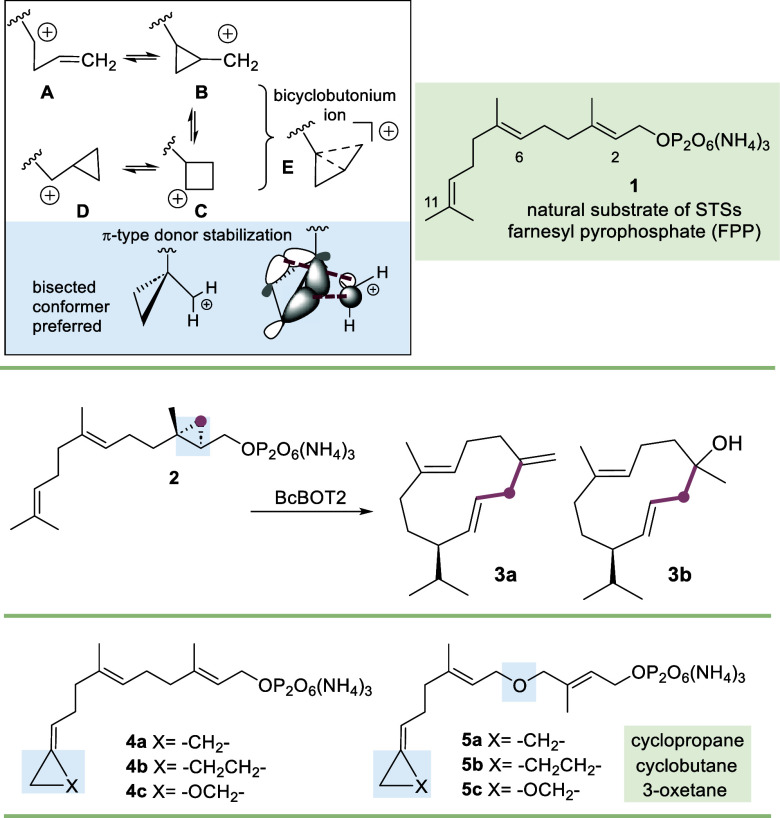
Top: The Chemistry of Cyclopropylmethyl Cations and Ring Expansion (Cyclobutyl Behaves Similarly in that Ring Expansion to the Corresponding Cyclopentyl Cation Occurs) and Structure of Farnesyl Pyrophosphate (FPP, **1**); Central: BcBOT2-Promoted Transformation of 2,3-Cyclopropyl-FPP **2** to Homosesquiterpenes **3a** and **3b**; Bottom: FPP Derivatives **4a**–**c** and **5a**–**c** Studied in This Work (FPPs Are Presented as Tris Ammonium Salts)

Recently, there has been growing interest in terpenes with regard to the expansion of the “terpenome”, with very different (chemo)­enzymatic and molecular biological concepts being pursued.^6^ Our studies contributed to this expansion by using chemically synthesized farnesyl and geranylgeranyl pyrophosphate derivatives and exposing them to terpene synthases (TS).^7^ We and especially Allemann’s, Wessjohann’s, and Dickschat́’s groups^8–10^ found that synthases are promiscuous transforming unnatural FPP and GGPP derivatives into new sesqui- and diterpenes, respectively. Along this line, it was shown in one case that the less reactive cyclopropylmethyl diphosphate group (in **2**) is also activated, e.g., by the fungal sesquiterpene synthase presilphiperfolan-8β-ol synthase (BcBOT2) providing homo sesquiterpenes **3a**,**b**. Clearly, the additional carbon in the cyclopropane ring in FPP derivative **2** has become part of the carbon backbone of the cyclization product (labeled in orange). Also, ether and thioether FPP derivatives were harnessed and transformed, and among them, FPP ether **47** ^2^ proved to be a particularly interesting substrate for sesquiterpene synthases (STSs).

Most of the reported biotransformations lead to macrocycles, but some sesquiterpene synthases, like BcBOT2, protoilludene synthase (Omp7), pentalenene synthase (PenA), caryolan-1-ol *synthase* (GcoA), and cubebene synthase (Cop4), were found to be privileged in that they are able to create oligocyclic products with uniquely new scaffolds.^7^ In selected cases, desirable olfactory properties were determined.^7a^ It is also important to note that these studies not only provide new terpenoids but also provide mechanistic insights with additional information about the mode of action of STSs, especially when supported by molecular modeling or crystal structure analyses of the protein.^11^

In view of the remarkable chemical properties of strained rings discussed above, we investigated FPP derivatives in which the geminal dimethyl moiety at C11 is exchanged by cyclopropane, cyclobutane, or oxetane. The cyclopropane and to a lesser extent the cyclobutane and oxetane rings can be regarded to be sterically “neutral” and likely will not cause significantly enhanced hindrance. As such, they should very well serve as substrates for STSs. We envisaged that the inherent chemical reactivity would lead to completely new terpene backbones after the first macrocyclization, when a cation has been placed in the vicinity of the strained rings.

Additionally, these three derivatives **4a**–**c** were further modified in that an ether group was inserted at C4–C5 into the linear carbon backbone of FPP (**1**) providing derivatives **5a**–**c**. In the past, we showed that this modification provides derivatives that are still transformed by STSs furnishing oxa-terpenoids with interesting olfactory profiles.^7a^ Noteworthily, Cane and Bowser employed cyclopropylidene-FPP **4a** that served as a mechanism-based inhibitor for trichodiene synthase.^12^ The authors mentioned the formation of three products (*m*/*z* 202) but did not elaborate on the isolation of these products.

## Results and Discussion

### Syntheses of FPP Derivatives

The syntheses of cyclopropylidene FPP-derivatives **4a** and **5a** are depicted in Scheme 2. These follow established protocols in their individual steps.^12^ The syntheses commenced from farnesol **6**, which was first *O*-acylated followed by an oxidative cleavage of the olefinic double bond at C10–C11. The resulting aldehyde **7** was subjected to a Wittig-type olefination using ylide **8a** to yield triene **9**. Alternatively, we probed the Julia–Kocienski reaction for generating the olefin.^13,14^ The known sulfone **8b** ^15^ was lithiated, and in the presence of aldehyde **7**, cyclopropylidene derivative **9** could also be prepared. However, we had to acknowledge that both methods were not high yielding.

**Scheme 2:**
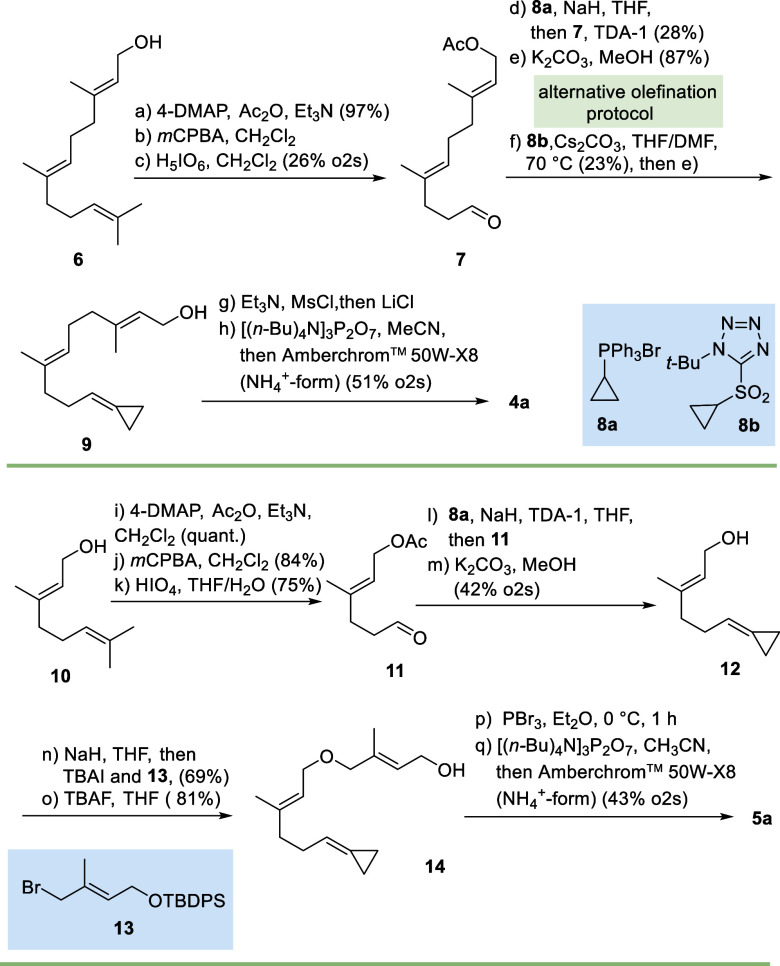
Synthesis of FPP Derivatives **4a** (Top) and **5a** (Bottom); Ac: Acetyl, DMAP: Dimethylamino Pyridine, DMF: Dimethylformamide, *m*CPBA: *Meta*-Chloroperbenzoic Acid, Ms: Mesyl, TDA-1: Tris­[2-(2-methoxyethoxy)­ethyl]­amine, THF: Tetrahydrofuran, TBAF: Tetra-*n*-butylammonium Fluoride, TBAI: Tetra-*n*-butylammonium Iodide

Finally, the diphosphate group was introduced by a standard sequence that yielded allyl chloride via the mesylate, followed by substitution with tris­(tetra-*n*-butylammonium) hydrogen pyrophosphate. Finally, ion exchange chromatography (exchange of *n*Bu_4_N^+^ to H_4_N^+^ form) gave diphosphate **4a** as trisammonium salt.^16^

The synthesis of FPP derivative **5a** started from geraniol **10** which was converted into aldehyde **11** in a sequence analogous to the one carried out with farnesol **6** as the starting point. Removal of the acetate protection yielded the corresponding alcohol **12** which was subjected to a Williamson ether synthesis with bromide **13**. Subsequent deprotection afforded ether **14** which was brominated and further elaborated to the trisammonium pyrophosphate salt **5a**. For the synthesis of cyclobutylidene derivative **4b**, aldehyde **7** served as the starting point which was coupled with reagent **15** (Scheme 3). First, cyclization occurs in situ to yield the *P*-ylide of cyclobutane, and this underwent olefination with the aldehyde, followed by acetate deprotection to furnish cyclobutylidene alcohol **16**. This was diphosphorylated via bromide to yield FPP derivative **4b**. In a similar fashion, FPP ether derivative **5b** was generated starting from aldehyde **11**. The cyclobutylidene containing C-10 fragment **17** was coupled under established conditions, followed by Williamson ether synthesis to yield allyl alcohol **18**. Diphosphate **5b** was then generated according to the protocol described above with tris­(tetra-*n*-butylammonium) hydrogen pyrophosphate as the phosphorylating agent. The corresponding ammonium salt was again obtained by ion exchange chromatography.

**Scheme 3:**
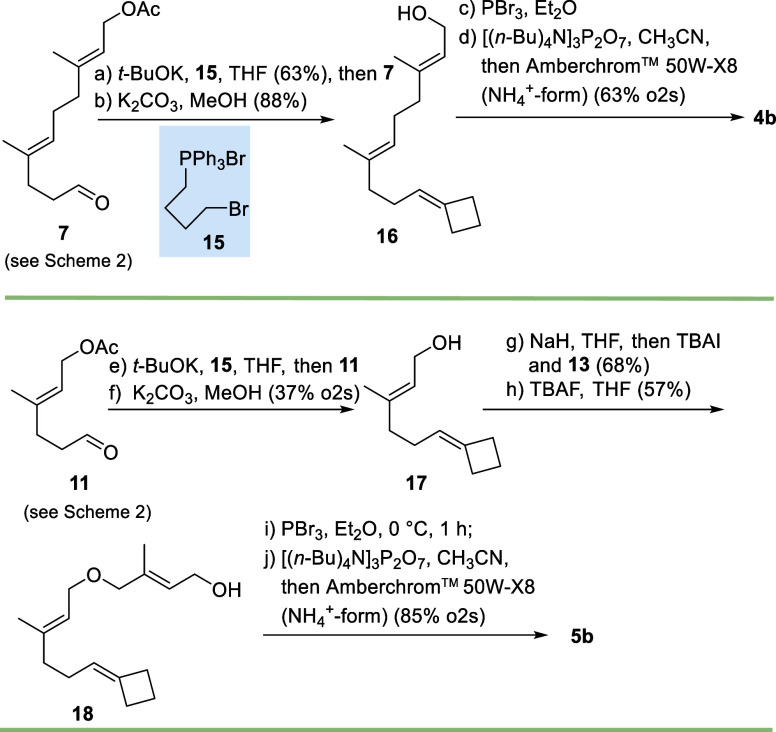
Synthesis of FPP Derivatives **4b** (Top) and **5b** (Bottom)

For the synthesis of oxetane **4c**, the farnesol building block was transferred into Wittig salt **20** via aldehyde **19** which was first reduced and then transformed into the corresponding alkyl iodide (Scheme 4). The subsequent olefination reaction with oxetan-3-one **21** followed by deprotection yielded 3-methyleneoxetane derivative **22**. Next, an alternative protocol to introduce the diphosphate was chosen. Instead of the two-step process using intermediate allyl halides, the allyl alcohol was directly coupled with triethylammonium phosphate (TEAP).^17^ This modified procedure provided diphosphates with improved purity, particularly in the case of the oxetane derivative. Finally, the ether derivative **5c** was synthesized from geraniol **10**, and the Williamson protocol between building blocks **25** and **13** served as the key reaction. After deprotection, alcohol **26** was transformed to FPP derivative **5c** again using TEAP as the pyrophosphorylating agent.

**Scheme 4:**
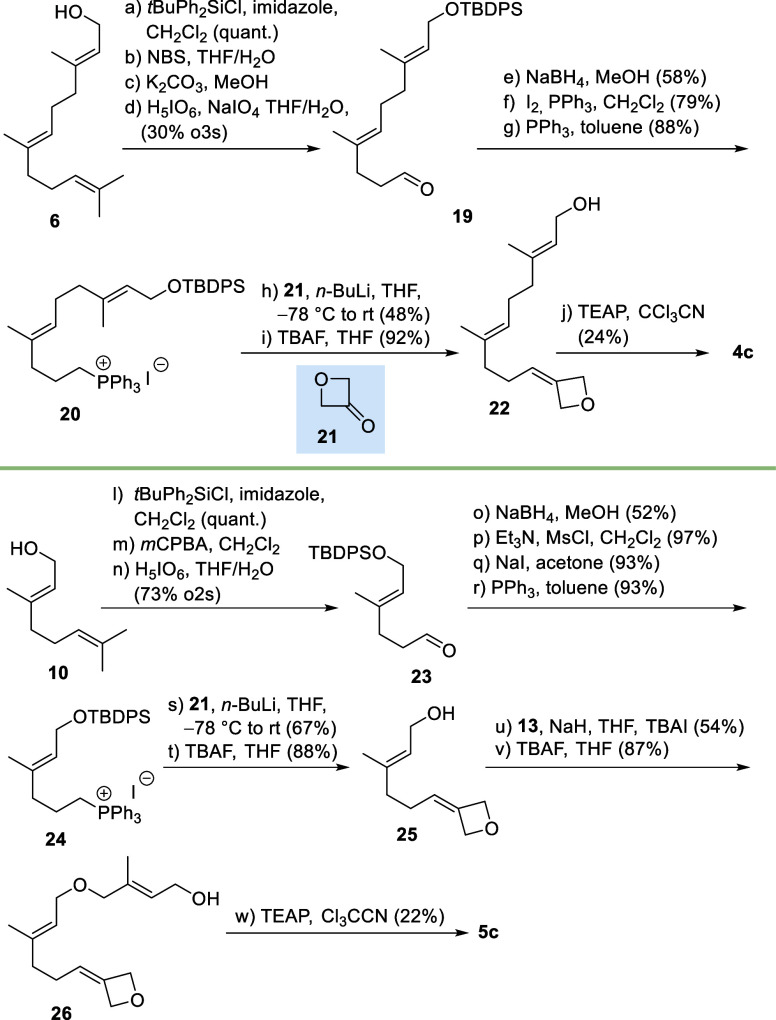
Synthesis of FPP Derivatives **4c** (Top) and **5c** (Bottom); NBS: *N*-Bromosuccinimide

### Biotransformations

After an initial screening using a set of nine different STSs (see the Supporting Information), we finally chose BcBOT2 (from *Botrytis cinerea*),^18^ PenA (first isolated from *Streptomyces exfoliatus* UC5319),^19^ Omp7 (from *Omphalotus olearius*),^20^ and cubebene synthase (from *Coprinus cinereus*)^21^ for biotransformations on a preparative scale. These form not just one, but several cyclization products with the natural substrate FPP (**1**), but the byproducts are commonly present in only minute quantities. The main products generated by these STSs have tricyclic core structures, which means that all three olefinic double bonds in FPP (**1**) are involved in the carbocation cascade. The main products are presilphiperfolan-8β-ol (**27**), pentalenene (**28**), Δ^6^-protoilludene (**29**), and the two isomers α-cubebene (**30**) and β-cubebene (**31**) (Figure 1).

**Figure 1:**
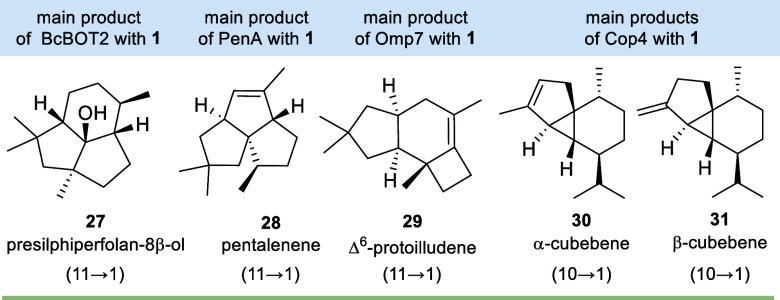
Sesquiterpenes **27**–**31** formed by STSs BcBOT2, PenA, Omp7, and Cop4 with FPP **1** (the initial mode of cyclizations is given).

In previous studies,^7^ we observed that STSs, which yield tricyclic terpene backbones, show a pronounced promiscuity toward unnatural FPP derivatives. These four STSs were cloned and expressed in *Escherichia Coli,* and after isolation, in vitro enzyme tests were conducted to determine enzyme activity using FPP (**1**) and substrate tolerance employing the new derivatives **4a**–**c** as well as **5a**–**c** on an analytical scale (150 μM, 0.01 mg/mL) (for details, see the Supporting Information). Optimization of key parameters such as temperature and pH, substrate, and enzyme concentrations had previously been reported elsewhere.^7^ Gas chromatography coupled with mass spectrometry (GC–MS) analysis provided an overview of the product spectrum and permitted the selection of biotransformations that were subsequently to be repeated on a larger scale. The common upscale conditions were *V* = 50 mL, 50 mM HEPES-buffer, 5 mM DTT, 10 mM MgCl_2_, 50 mM NaCl, 1 mM substrate, 1 u PPase, 0.01% Tween 20, and 0.1 g/L STS (see the Supporting Information for further details). New products were isolated by different chromatographic techniques after pentane extraction, and the structure elucidation mainly relied on different nuclear magnetic resonance (NMR) protocols (Scheme 5 and Figure 2).

**Scheme 5:**
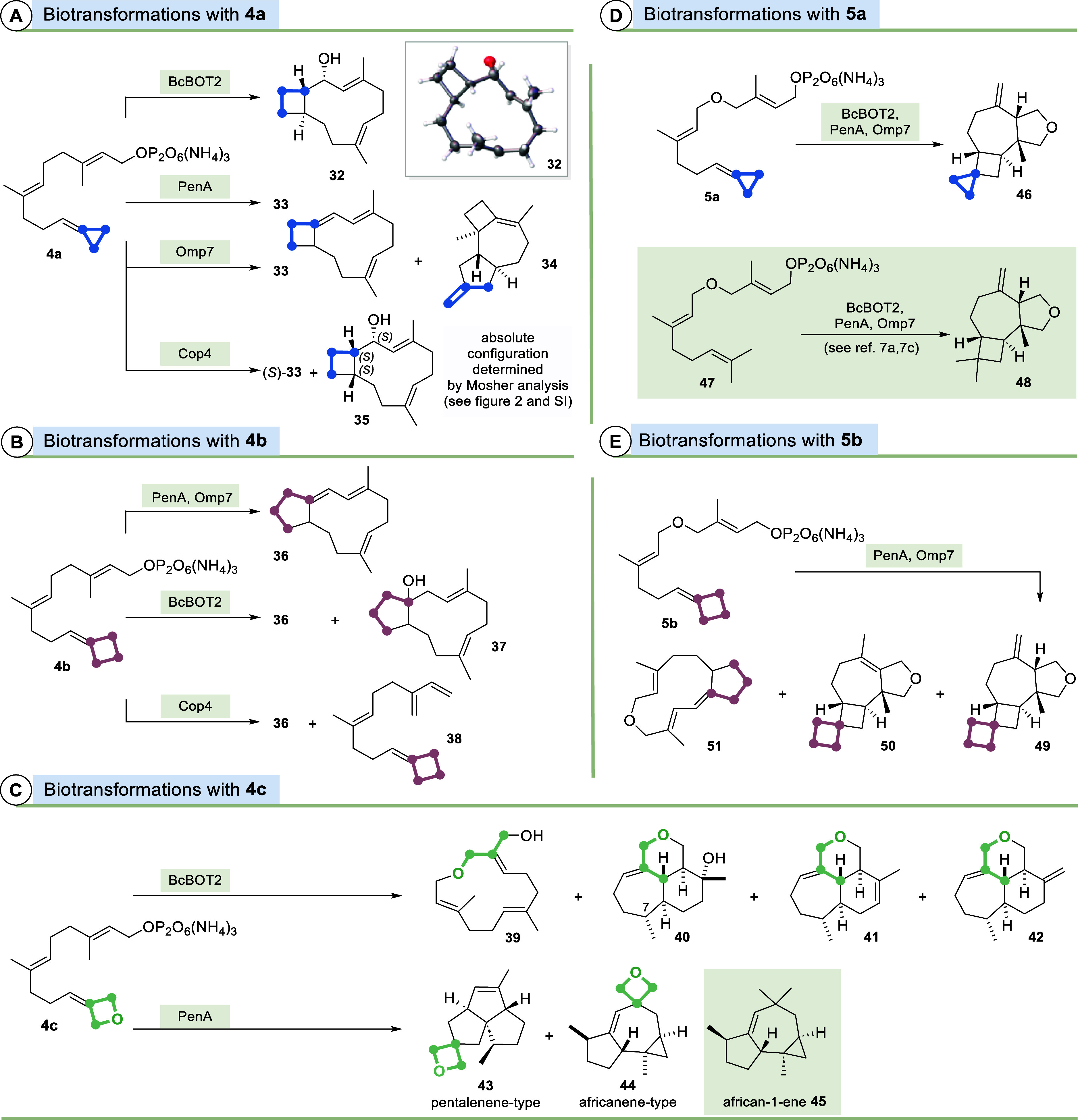
Biotransformations of FPP Derivatives **4a**–**c** and **5a**,**b** with STSs BcBOT2, PenA, Omp7, and Cop4 (FPP Derivatives Are Presented as Trisammonium Salts)

**Figure 2:**
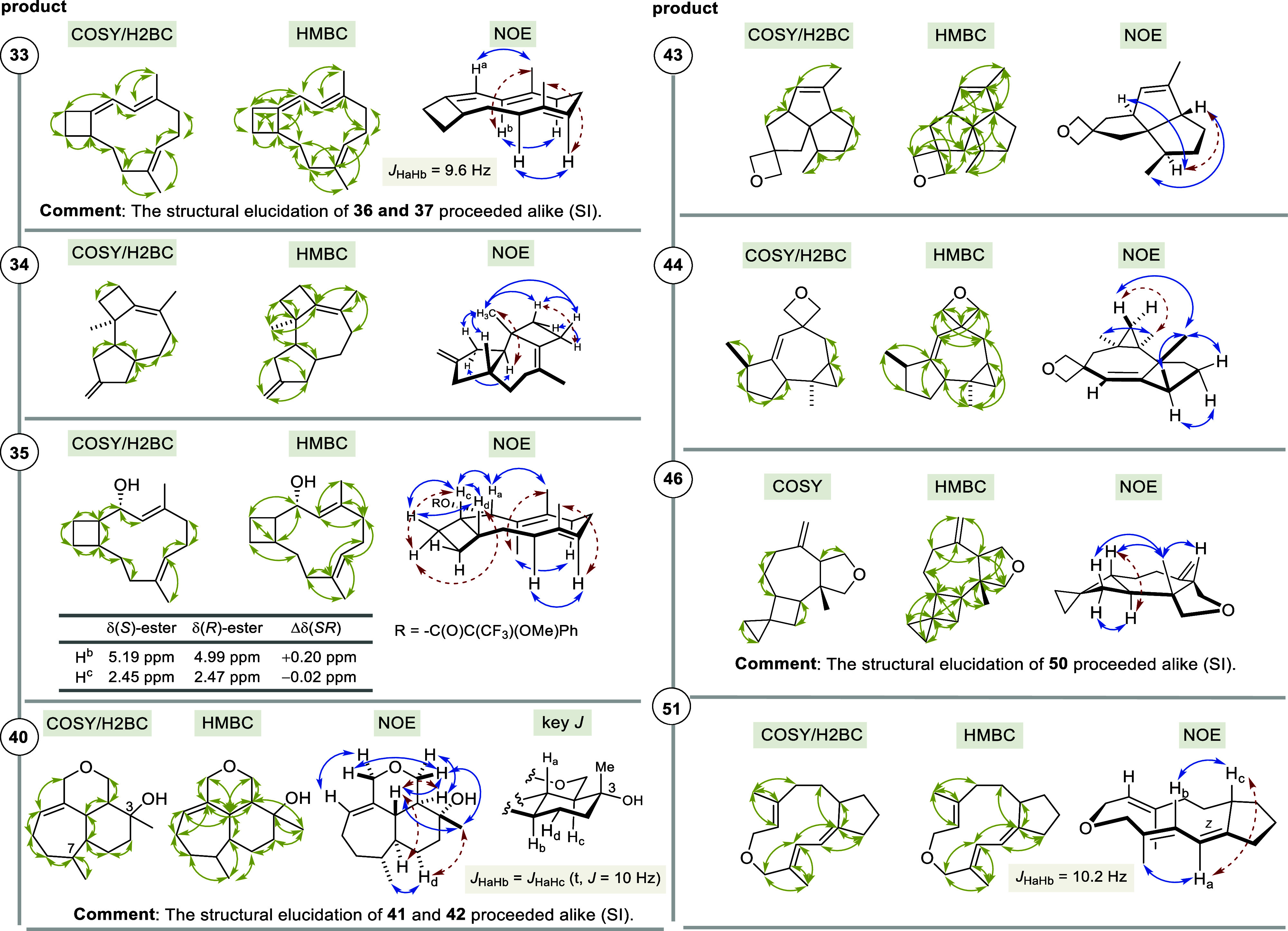
Key NMR spectroscopic correlations essential for the structure elucidation of products **33**–**35**, **40**, **43**, **44**, **46**, and **51** (blue arrows indicate nuclear Overhauser effect (NOE) correlations and the red arrows indicate that no NOE correlations were observed; yellow arrows reveal correlations in correlation spectroscopy (^1^H–^1^H COSY) and heteronuclear multiple–bond correlation/heteronuclear 2-bond correlation (^1^H–^13^C HMBC/H2BC) 2D NMR spectra). In selected cases, data of Mosher-ester analysis are listed.

For FPP derivatives **4a** and **4b** as well as for the FPP ether, **5a** biotransformations with the STSs BcBOT2, Omp7, and PenA provided new products in sufficient amounts. For Cop4, we encountered transformations only for the first two FPP derivatives. Substrate **4c** was accepted by terpene synthases BcBOT2 and PenA, and for derivative **5b**, the STSs PenA and Omp7 furnished the same products. Interestingly, close mechanistic similarities have been noted between the STSs BcBOT2, Omp7, and PenA using unnatural FPP derivatives before.^7c^ Finally, biotransformations with oxetane ether **5c** did not yield new products in sufficient amounts (as judged by GC–MS) for all terpene synthases tested. With substrate **4a**, BcBOT2 yielded terpenoid **32** that bears a bicyclo[9.2.0]­tridecane backbone (Scheme 5A). Its structure was unequivocally determined by X-ray analysis. The STS Omp7 provided the cyclization product **33** and the byproduct **34** that were structurally characterized NMR-spectroscopically after chromatographic purification. Terpenoid **33** was also formed by Cop4 along with the closely related alcohol **35** which resembles the water adduct of **33**. The absolute stereochemistry of the terpenoid **35** was assigned by NMR analysis of the Mosher esters prepared.^22^ Ultimately, the cyclopropane ring actively intervenes or becomes involved after the first step of the carbocation cascade. In this way, the bicyclic backbone is formed under the influence of all four enzymes. This is remarkable because it differs mechanistically from the native route. There, FPP **1** undergoes further cyclization reactions at this point, which are made possible by the remaining olefinic π-bonds and ultimately lead to terpenoids **27**–**31**.

Cyclobutylidene derivative **4b** was transformed to new cyclization products **36** and **37**, depending on the STSs employed (Scheme 5B). The latter formally corresponds to the water adduct of **36**. With Cop4, we observed the formation of terpenoid **36**, but the main product is the linear elimination product **38**. These results and observations correspond to the findings for the smaller cyclopropane analogue **4a** because the cyclobutane ring in **4b** also rearranges to the next larger cyclopentane ring system after the first cyclization step. In contrast, the oxetane moiety present in FPP derivative **4c** turned out to behave differently in this cationic scenario. In the presence of BcBOT2, it undergoes ring opening in which the ether oxygen atom acts as a nucleophile to yield oxa-terpenoids **39**–**42** (Schemes 5C and 7A).

However, this mode of action of the oxetane oxygen atom does not apply to all STSs because in the case of PenA, the oxetane ring is found unaltered in products **43** and **44** corresponding to **28** and **45**, respectively. While the formation of the oxetane derivative of pentalenene-type **43** can reasonably be explained, the formation of tricyclic terpenoid **44** is rather surprising as it represents the oxetane derivative of african-1-ene (**45**) with a completely different carbon backbone compared to the tricyclic pentalenene ring system (Schemes 5C and 7B).^23^

The corresponding ether FPP derivatives **5a**–**c** are less well accepted by the chosen STSs. BcBOT2, PenA, and Omp7 gave the tetrahydrofuran terpenoid **46** from FPP derivative **5a**. This is the cyclopropane derivative of the known tetrahydrofuran **48** obtained by the BcBOT2-promoted transformation of FPP ether **47**.^7a^ Interestingly, both oxa-terpenoids **46** and **48** also form in the presence of STSs PenA and Omp7, which again underlines the close relationship of this enzyme trio from a mechanistic point of view.^7c^ Finally, pyrophosphate **5b** gave an analogous result with STSs Omp7 and PenA. Together with terpenoid **51**, the two isomeric oxa-terpenoids **49** and **50** were isolated as the main products, which are the homoderivatives of **46**.

### Structure Analyses

The structure elucidation of new terpenoids relied on several NMR spectroscopic methods and (high-resolution) mass spectrometry to distinguish between products resulting from deprotonation or water trapping of the final cation (Figure 2). As mentioned above, Mosher ester derivatization^22^ was part of the analytic portfolio to determine the absolute stereochemistry. Terpenoid **32** crystallized which allowed to collect X-ray crystallographic data and unequivocally determine the stereochemistry (Scheme 5A).^24^ These data also facilitated the structural elucidation of terpenoid **35**, which in addition was accompanied by the preparation of the two diastereomeric Mosher esters with subsequent NMR determination of the absolute configuration (see the Supporting Information). The protocol allows differentiation of the two diastereomeric protons H_c_ and H_d_ in the ^1^H NMR spectrum. The *syn* relationship between H_a_ and H_c_ was assigned based on the small coupling constant (*J*_HaHb_ = 3.2 Hz in Mosher esters). Since the chemical shifts (δ) in the ^1^H and ^13^C NMR spectra differ between the terpenoids **35** and **32**, we concluded that this is the *cis*-annulated diastereomer, which found further support from the 2D-NOE data as indicated in Figure 2: (**35**) highlighting the important and missing correlation signals in blue and red, respectively.

The structure elucidations of related terpenoids **33**, **35**, and **36** posed some challenges with respect to the geometry of the diene units in **33** and **36**. The coupling constants *J*_HaHb_ in **33** and **36** were determined to be *J* = 9.6 Hz and *J* = 9.7 Hz, respectively, which is indicative for an *s-trans* conformation around the diene unit. This conformation is further supported by the lack of a ^1^H–^1^H NOESY correlation signal between H_b_ and the methyl group in both terpenoids. In contrast, a ^1^H–^1^H NOE effect correlation signal was detected between H_a_ and the methyl group. The assignment of the absolute stereochemistry of (*S*)-**33** is a result of the aforementioned analysis of **36**.

The proposed structure of tricyclic terpenoid **34** can be explained by ^1^H–^1^H COSY/^1^H–^13^C H2BC NMR correlations and distinct ^1^H–^13^C HMBC correlations to the CH_3_ groups and the exomethylene group. The position of the latter is further supported by a clear lack of ^1^H–^13^C H2BC correlations of the adjacent, allylic methylene groups to each other and the presence of respective correlations to the neighboring CH protons. The anti–anti relationship of the stereocenters is deduced from ^1^H–^1^H NOESY correlations, which are supported by clearly assignable diastereotopic protons (Figure 2; **34**).

The two oxa-terpenoids **43** and **44** are derived from the sesquiterpenes pentalenene (**28**) and african-1-ene (**45**), respectively. Comparison of the published NMR data for these natural products validated the structure determination of the new oxetane derivatives. In the ^13^C NMR spectrum, the chemical shifts of the quaternary centers that form the triquinane backbone differ only slightly (for **43**: δ = 63.7, 61.2, and 58.3 ppm and for **28**: δ = 62.1, 62.4, and 59.0 ppm). Excluding the oxetane subunit, the average deviation of ^13^C shifts (δ) for each corresponding atom is max. 2.1 ppm (see the Supporting Information for a full table comparison). For african-1-ene, key chemical shifts like the cyclopropane motif show high similarity to the oxa-terpenoid derivative (for **44**: δ = 23.3, 21.9, and 19.9 ppm and for **45**: δ = 22.6, 21.8, and 20.6). Overall, the average deviation in the ^13^C NMR spectrum for each corresponding atom is max. 2.2 ppm (see the Supporting Information for a full table comparison). Additionally, the stereochemistry finds further support from ^1^H–^1^H NOESY correlations (Figure 2). The structural elucidations of the spiroterpenoids **46**, **49**, and **50** were carried out in a very similar manner. For this purpose, ^1^H–^1^H COSY and ^1^H–^1^H NOESY correlations were used, as well as a comparison with NMR data collected for sesquiterpene furanoside **48** ^7a,7c^ (Figure 2; **46**).

Oxacyclododecin **51**, which is formed from the ring expansion product after the initial macrocyclization of **5b**, exhibits a certain degree of rigidity due to its triple desaturation and cyclopentane annulation. It is suggested that the diene group is *s-trans* oriented, using the same reasoning as in the cases of **33** and **36**. The coupling constant (*J*_HaHb_) was determined to be 10.2 Hz. Given this rigidity, ^1^H–^1^H NOESY correlations between H_b_ and H_c_ would be expected in the case of a (*Z*)-configured double bond, which would result in a less strained ring structure. An (*E*)-configuration, on the other hand, should lead to correlations between H_a_ and H_c_, which, however, could not be observed. We therefore propose the (*Z*)-configuration shown (Figure 2; **51**).

The backbones of terpenoids **40**–**42** represent a highly unusual saturated cycloheptaisochromene motif. The constitutions of these were solved by the combination of ^1^H–^1^H COSY, ^1^H–^13^C H2BC, and ^1^H–^13^C HMBC NMR experiments, whereas the relative stereochemistry was assigned by determining ^1^H–^1^H NOESY correlations in which the correlation of the CH_3_ group at position 3 with H_a_ was essential. Additional support was gained from the diastereotopic protons of the pyran substructure. Coupling constants (*J*_HaHb_ = 10 Hz) between H_c_–H_a_–H_b_ provided evidence for the *all*-trans stereochemistry around the cyclohexane unit (Figure 2, for **40**). The orientation of the methyl substituent at C7 could not unequivocally be determined due to the overlap of several proton signals in the ^1^H NMR spectrum. ^1^H–^1^H NOESY correlation between the methyl group to the diastereotopic proton H_d_ suggests that it is *syn*-oriented to H_b_. However, the inherent conformational flexibility of the 7-membered ring leads to a residual ambiguity.

### Mechanistic Considerations

In general, the presence of small, strained rings leads to an early separation of the presumed mechanistic pathways from those proposed for FPP **1** after the first cyclization step. The strained rings therefore cause rearrangements and ring openings that are not possible with FPP. Consequently, there are no analogous intermediate cations in the “natural” mechanisms for the newly formed carbocations. Mechanistically, formation of bicyclic terpenoids **32** and **33** as well as **35**–**37** likely proceeds via a similar carbocationic cascade starting from FPP derivatives **4a** or **4b**, respectively (Scheme 6a). In both cases, the sequence is initiated by a (11 → 1) cyclization, which for Cop4 is remarkable as it opposes its natural (10 → 1) mode of cyclization with FPP—and the carbocations **52a** and **52b** undergo ring expansion either to the cationic cyclobutane **53a** or the cationic cyclopentane **52b**, respectively. Then, deprotonation provides trienes **33** (*n* = 1) or **36** (*n* = 2); the (*Z*)-configured alkene is formed in both cases (route a).

**Scheme 6:**
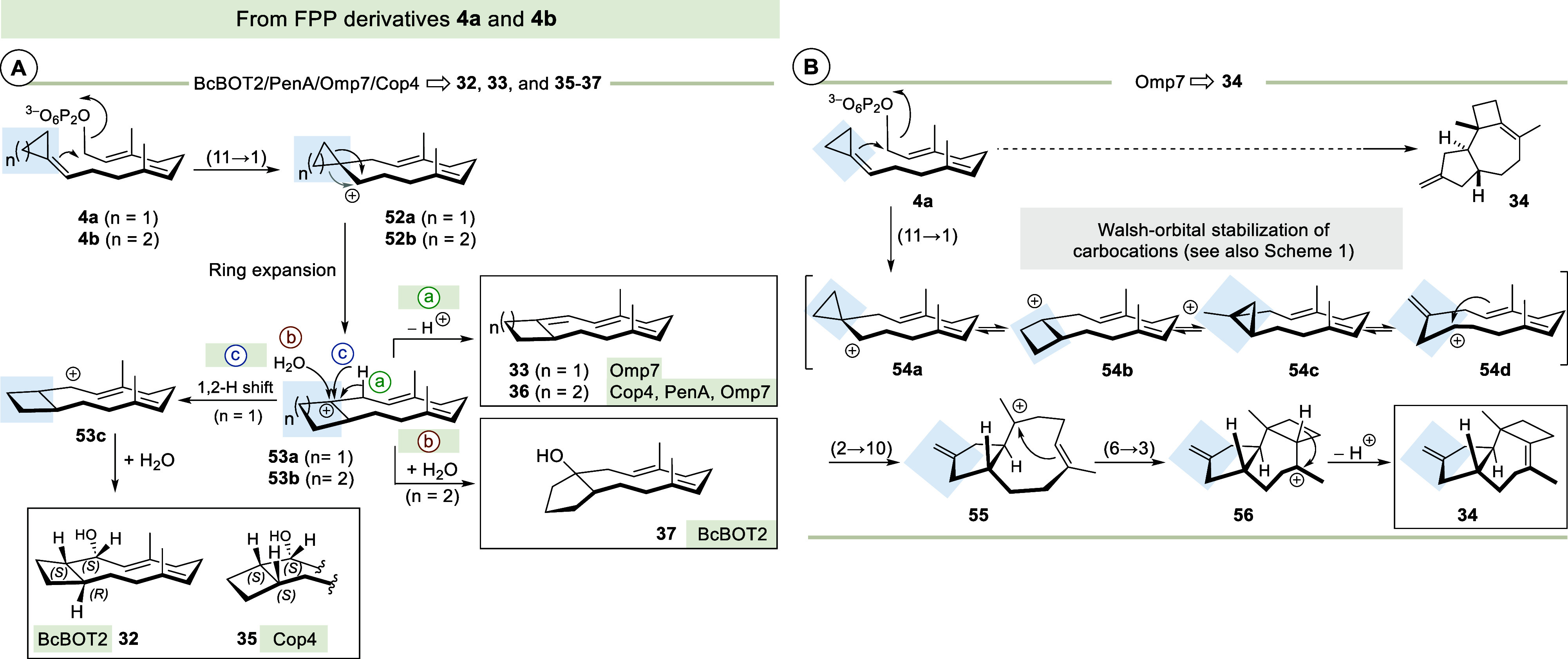
(A) Mechanistic Considerations on the Formation of Terpenoids **32**, **33**, and **35**–**37** and (B) of Terpenoid **34**

At present, no statement can be made about the absolute stereochemistry of the three new products **33**, **36**, and **37**. The reason for this is that the embedding of the strained small rings creates a situation in which two enantiotopic C–C bonds can, in principal, undergo a ring-expanding rearrangement (**52a,b** → **53a,b**). This would lead to opposite absolute configurations in the products. It is not possible to predict which of the two bonds would likely undergo ring enlargement, and alternatively, no further analytical data, such as X-ray structural analyses, could be collected.

Compared to the alternate (*E*)-configured alkene, this double bond configuration leads to a lower ring tension in the 11-membered macrocycle, as it already has to accommodate two (*E*)-configured double bonds. In addition, the terpenoid **37** is formed when carbocation **52c** is trapped by water (route b). For the STSs Cop4 and BcBOT2, an additional 1,2-H shift can be proposed that yields the allylic cation **53d** (route c) which then reacts with water to furnish diastereomeric terpene alcohols **32** and **35**, respectively.

The proposed mechanistic route to the tricyclic terpenoid **34** supposedly proceeds via an initial (11 → 1) cyclization. The resulting cyclopropylcarbinyl cation **54a** undergoes a bicyclobutonium ion rearrangement as depicted in Scheme 1 (top). While intermediate **54b** results in the aforementioned cyclobutane trapping products via intermediate **54c**, a potential homoallyl cation **54d** is formed and finally trapped by the olefinic double bond at C2–C3 (2 → 10 cyclization).

A second (6 → 3) cyclization with intermediate **55** provides cation **56** which after deprotonation furnishes terpenoid **34** (Scheme 6B).

According to our results, two main cyclization scenarios can occur with FPP derivative **4c**. The first route includes ring opening of the oxetane ring in which the oxygen atom serves as nucleophile (promoted by BcBOT2). For the second scenario, the oxetane ring can stay intact during cyclization (promoted by STS PenA; Scheme 7).

**Scheme 7:**
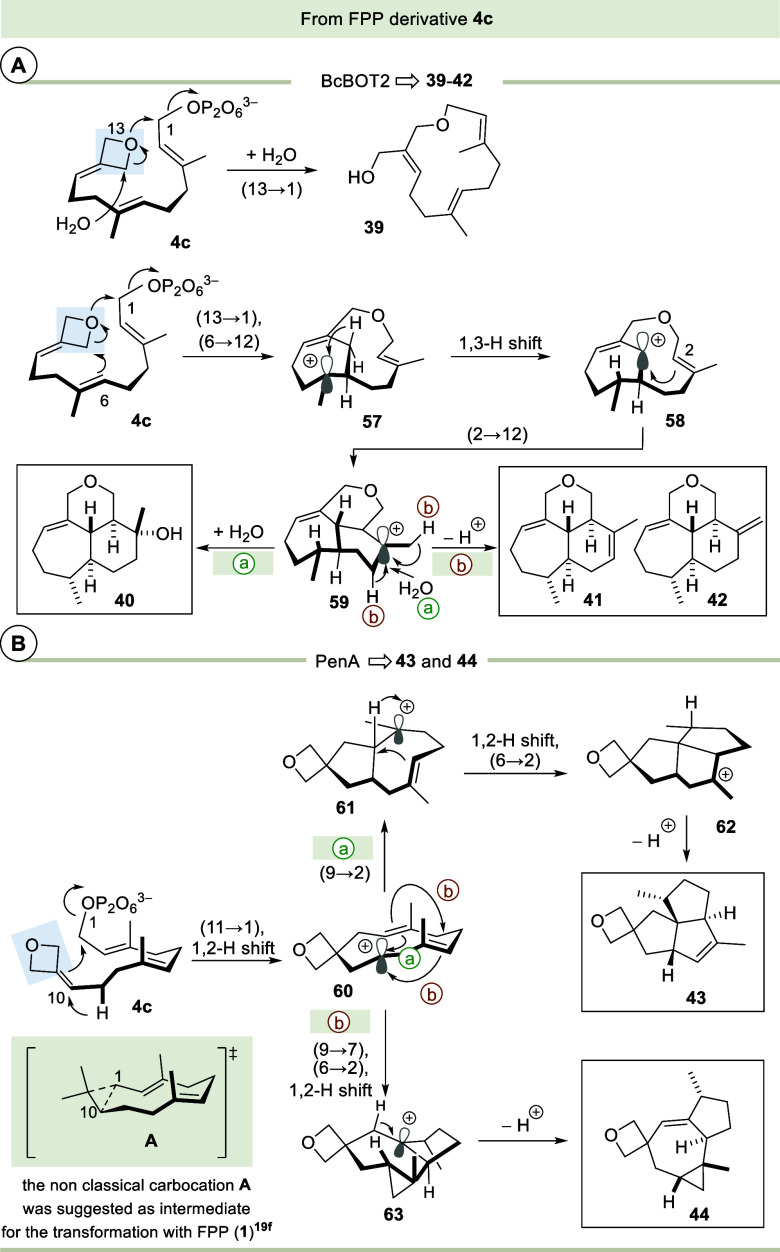
Mechanistic Considerations on the Formation of Terpenoids **39**–**42** and **43**, **44** from the FPP Derivative **4c**

For the first case, again two routes can be proposed that divert right in the first cyclization step. One leads to macrocyclic ether **39** whose formation is initiated by nucleophilic attack onto C1 and the emerging cation at one of the diastereotopic methylene groups is trapped by water. This is the first example of a (13 → 1) cyclization promoted by BcBOT2 or any other STS. Cyclization across an oxygen atom has already been described in a few cases. Allemann and co-workers reported a case involving the STS germacrene A synthase, in which an oxirane acts as a nucleophile and initiates the formation of a macrocyclic ether.^9e^ In addition, it was demonstrated that also alcohols can serve as intramolecular nucleophiles in such cyclization scenarios.^7f,9e^

The cation initially formed by nucleophilic attack of the oxetane oxygen atom can alternatively undergo ring closure with the alkene unit at C6 and C7, which results in cycloheptyl cation **57**. 1,3-Hydride shift leads to allyl cation **58** which induces a second cyclization with C2. The resulting cation **59** finally reacts with water to afford the tricyclic terpenoid **40**. Proton abstraction at **59** produces two regioisomeric elimination products **41** and **42**.

With the STS PenA, the biotransformation of the FPP derivative **4c** proceeds in a different fashion, as it starts with an (11 → 1) cyclization^25^ followed by a 1,2-hydride shift, so that the oxetane ring does not interfere into the cationic cascade. Once carbocation **60** is formed, the cascade splits into two pathways. A (9 → 2) cyclization provides intermediate **61** (route a) which after a 1,2-H shift furnishes carbocation **62** before a final deprotonation yields the oxa-pentalenene derivative **43**. The second route involves a (9 → 7) along with a (6 → 2) cyclization, which establishes the cyclopropane ring present in **63** (route b). An 1,2-hydride shift followed by deprotonation finally would provide oxetane **44**.

The doubly modified FPP derivative **5a** was accepted and converted as a substrate by the three STSs BcBOT2, PenA, and Omp7, while **5b** was transformed by the latter two. This resulted in the formation of the tetracyclic furan terpenoids **46**, **49**, and **50**. As proposed for the case reported before which described the formation of oxa-terpenoid **48** from FPP derivative **47** by the same set of enzymes,^7a,7c^ the formation of **46**, **49** and **50** should proceed via a series of cyclizations starting with a (12 → 1) ring closure (Scheme 8). The (11 → 2)- and (3 → 7)-cyclizations and a final deprotonation step complement the cationic cascade. Interestingly, the cyclopropylcarbinyl cation, which can be postulated after the first ring closure, does not undergo rearrangement but is directly intercepted by the olefinic double bond (C2, C3). This is a strong indication of a concerted cascade mechanism. In the case of reactant **5b**, however, we also isolated the bicyclic terpenoid **51**, which corresponds to the product of a ring expanding Wagner–Meerwein rearrangement preceded by a (12 → 1) macrocyclization and completed by deprotonation. This behavior differs from those observed for FPP derivatives **4a** and **4b**, where rearrangements and ring expansions are commonly observed in the main products. Interestingly, these results provide additional evidence that the three STSs BcBOT2, PenA, and Omp7 show very similar behavior toward unnatural FPP derivatives.^7c^

**Scheme 8:**
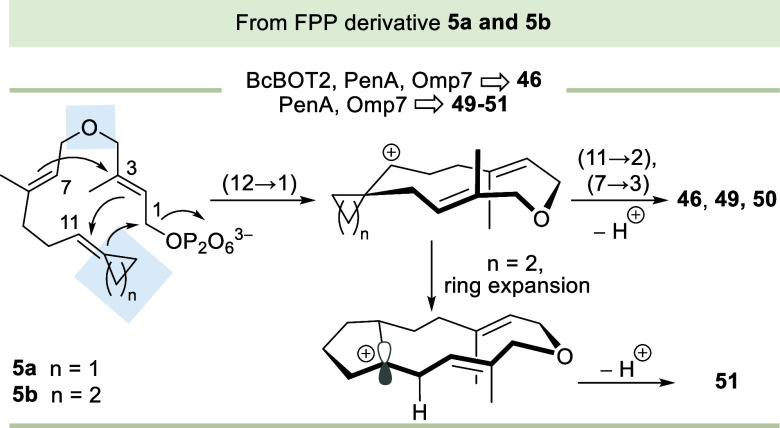
Proposed Mechanism for the Formation of Terpenoids **46** and **49**–**51**

Finally, it is interesting to note that the three furanosesesquiterpenoids **46**, **49**, and **50** show an olfactoric profile similar to that of the known derivative **48**. It was found by GC-O that the tricyclic furan **48** exerts an ethereal, camphoraceous, and peppery scent. In terms of the peppery note, the sensory profile is thus similar to the sesquiterpene rotundone, found in many essential oils such as patchouli oil, agarwood, or various pepper oils.

### Computational Rationalization

Previous high-level quantum chemical calculations along with X-ray structural studies provided concrete basis for modeling the enzymatic reaction of STSs. Such insights revealed that the near-attack conformation (NAC) criteria of the catalytic pose required for the ring closure are defined by the C–C distance of the relevant carbon atoms in the substrate. This distance should be around 4.0 Å.^26^

Relying on those insights, we employed a mechanistic-aware predictive docking method to reliably predict the catalytic binding modes of substrates within the enzyme active site. The docking-based conformational sampling was validated in our previous benchmark and reported by others.^27^ We further evaluated the predicted catalytic binding mode(s) with molecular dynamics (MD) simulations.

To shed light on the different cyclization behavior of FPP derivatives **4a**–**c**, **5a**, and **5b**, we performed mechanism-based docking for all substrates and proposed carbocationic intermediates to rationalize the formation of the different terpene skeletons. For that, we analyzed the possible binding modes of the substrates and the subsequent generated carbocations in the active site of STSs, specifically of BcBOT2, PenA, and Omp7 based on NAC criteria mainly.

First, the analysis of the binding modes revealed that both substrates **4a** and **4b** possess the same catalytic binding mode which favor the initial (11 → 1) cyclization with C1–C11 distances around 3.60 ± 0.20 Å. This catalytic pose exhibits favorable alignment with natural substrate FPP **1** (Figure 3A). Detailed predicted poses for both **4a** and **4b** in the active sites of BcBOT2 and PenA, respectively, are found in the Supporting Information (Figures S22–S31, Supporting Information). Similar to PenA, STS Omp7 exhibited comparable catalytic poses for **4a** and **4b** (Figures S32, Supporting Information).

**Figure 3:**
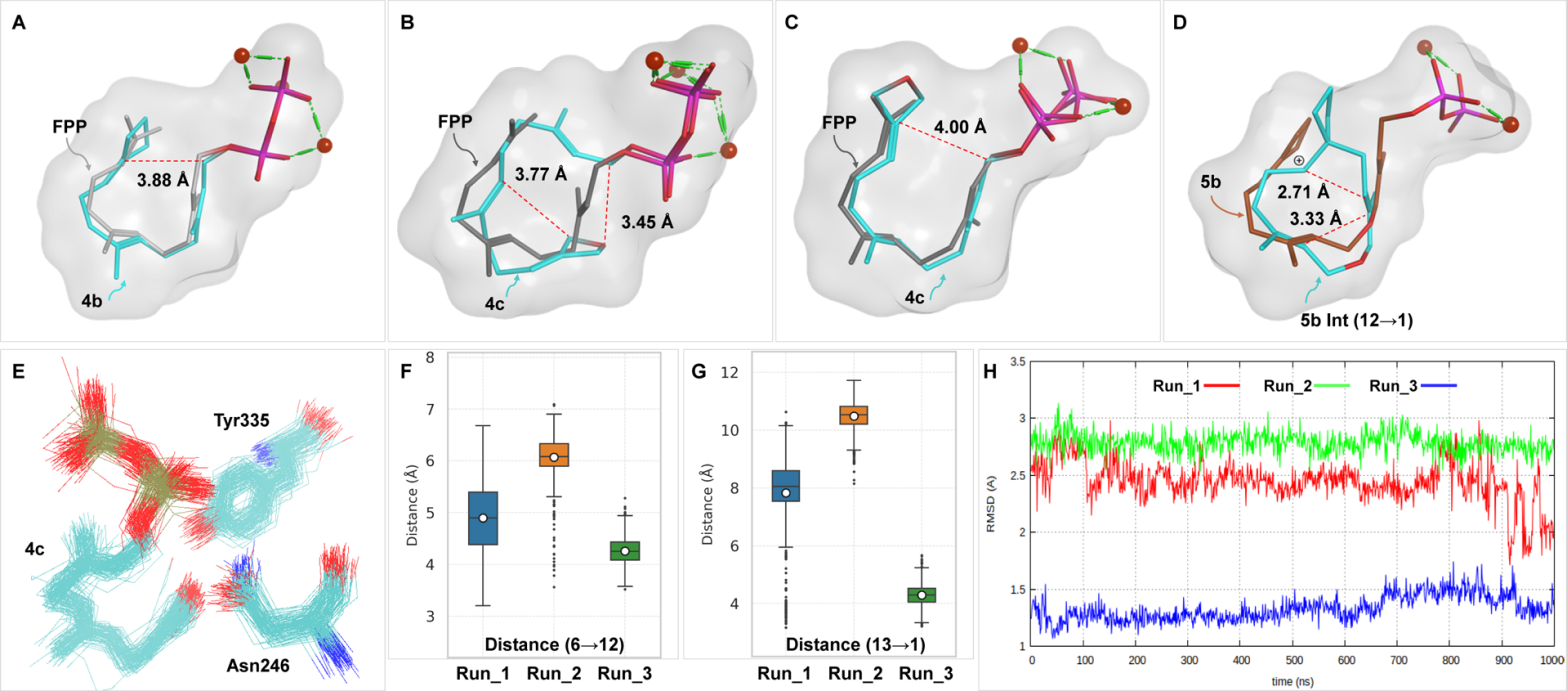
Closeup view of the catalytic binding modes of **4b**, **4c**, and **5b** in the binding pocket of BcBOT2 and PenA enzymes. (A) The catalytic binding mode of **4b** in the active site of STS BcBOT2. The top scoring docking pose is like that of natural FPP **1** fulfilling the NAC criteria for (11 → 1) cyclization with a distance of 3.88 Å and the ionic interactions between the pyrophosphate moiety and the Mg^2+^ ions. Also, all the subsequent carbocations bind in the same way which revealed the stabilization through π-system–cationic interactions. (B,C) The catalytic poses of **4c** in the active sites of STSs BcBOT2 and PenA pockets each aligned to FPP **1**. (B) The 3D visualization of the BcBOT2 active site shows that **4c** has an opposite binding mode to FPP **1**. This conformation satisfies the NAC criteria for a (13 → 1) cyclization via the activation of oxetane oxygen with (6 → 12) cyclization with respective distances 3.45 Å and 3.77 Å. (C) The catalytic pose of **4c** in the PenA pocket exhibits a favorable alignment with FPP **1** conformation via fulfilled NAC criteria for a (11 → 1) cyclization pathway with 4.0 Å. (D) The catalytic pose of **5b** (shown in brown sticks) in the active site of PenA. The top scoring catalytic competent docking poses showed that two concurrent cyclization reactions can occur after a (12 → 1) ring closure attributed to the short catalytic distance between the involved carbon atoms (11 → 2) at 2.71 Å and (7 → 3) at 3.33 Å as shown in cyan sticks. These favorable catalytic distances are lacking in the docking poses of **4a** and **4b**, which instead proceed via distinct ring-expansion pathways. (E) Overlay of MD snapshots simulations show the stability of the **4c** binding mode (C1) in the BcBOT2 active site stabilized by the strong hydrogen bond interactions with either Try335 or Asn246. (F) and (G) The NAC distances distribution over the MD simulation runs for both (6 → 12) and (13 → 1) cyclizations, respectively. (H) The time-evolved root-mean-square deviation for the protein backbone over three independent MD simulation runs illustrates the stability of the **4c** conformation (C1) in the active site of BcBOT2. From (F–H), in MD simulations; run1 and run2 the extended conformation of **4c** is more favored, which was also proposed for FPP derivatives in a previous study.^6a^

Second, we analyzed the catalytically competent binding poses in the pockets of BcBOT2 and PenA for substrate **4c**. Remarkably, for BcBOT2, we determined that it possesses two possible binding modes. The first conformation (C1) has an opposite orientation to the natural FPP **1** while keeping the same Mg^2+^-pyrophosphate interaction and the second conformation (C2) is in a fair alignment with FPP. Both poses fulfill the catalytic criteria for short distances (6 → 12) and (13 → 1) activating the oxetane oxygen atom to generate the carbocation **57**, which proceeds to form the products **40**–**42** (Figure 3B and Scheme 7A). In contrast, for PenA, no favorable catalytically competent pose was found for (6 → 12) and (13 → 1) cyclizations, instead favoring the consensus pathway (11 → 1) which yields carbocation **60**, then falling into the cationic cascade to furnish the products **43** and **44**, respectively (Figure 3C and Scheme 7B).

Such different binding modes of **4c** in the binding sites of BcBOT2 and PenA could be attributed to the differences in the binding pocket volume, which is about 575 and 1450 Å^3^, respectively, as well as in the electrostatic properties of both active sites (Figures S33 and S34, Supporting Information). The unprecedented binding mode (C1) and also conformation C2 of **4c** in the active site of BcBOT2 were further evaluated by molecular dynamic (MD) simulations (three independent runs for each conformation, 1 μs each, 6 μs total). The MD simulations revealed the stability of such a binding mode (C1) that is stabilized beyond the Mg^2+^-pyrophosphate interaction by hydrogen bond interactions with either Tyr335 or Asn246 (Figure 3E–H). The catalytic poses of all proposed intermediates for each path support such scenarios. Detailed poses for both scenarios are shown in the Supporting Information (Figures S35–S46, Supporting Information).

Third, **5a** and **5b** adopt conformations in the active sites of PenA and Omp7 enzymes similar to those of **4a** as well as **4b**, respectively. However, in addition to the (12 → 1) cyclization, these compounds exhibit short catalytic distances between C11–C2 (2.70 Å) and C7–C3 (3.30 Å), which are absent in the cases of **4a** and **4b**. These favorable distances enable two additional concurrent cyclization reactions. These intermediates subsequently undergo a final deprotonation, resulting in the formation of the final products **46** and **50**, respectively (Figure 3D). Detailed docking poses are found in the Supporting Information (Figures S47–S51, Supporting Information).

## Conclusions

An analysis of the biotransformation products with reference to newly generated structural elements resulting from the incorporation of strained rings at the aliphatic end of FPP (**1**) reveals the opportunity to access completely new terpene-derived carbon scaffolds including spiro-derivatives derived from known unnatural terpenoid backbones (Figure 4).

**Figure 4:**
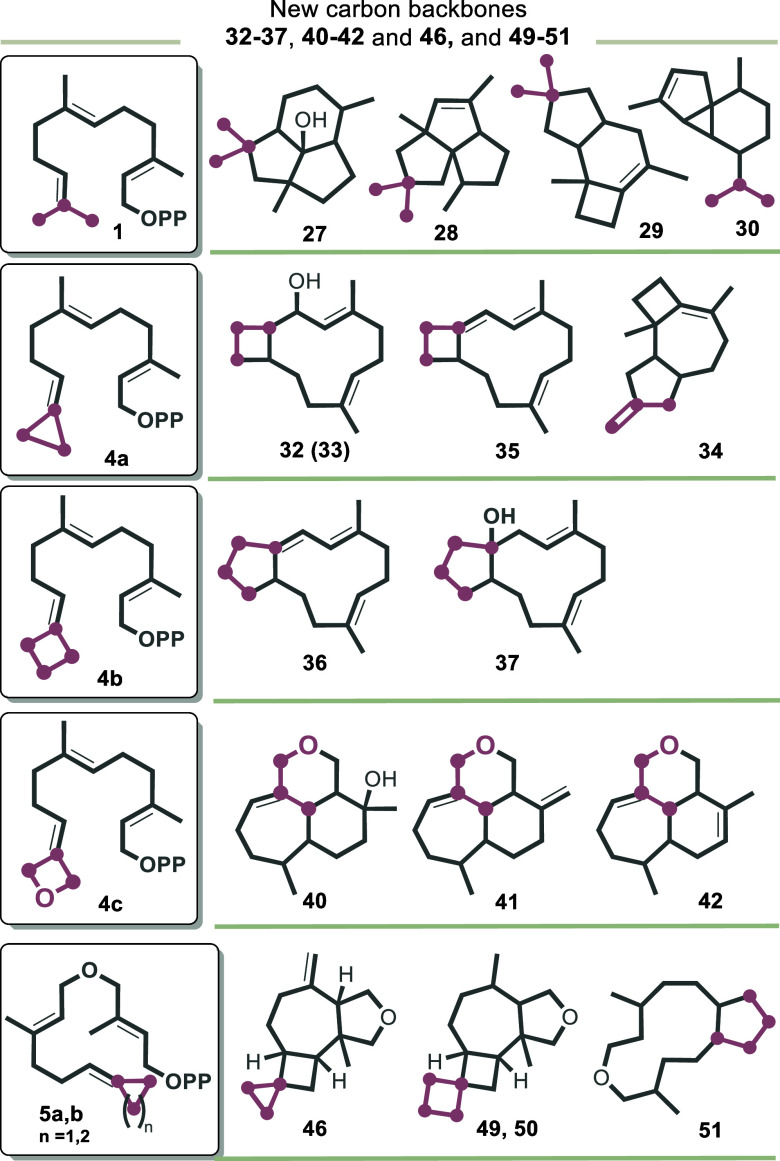
Comparative analysis of carbon backbones found in FPP-derived sesquiterpenes **27**–**30** and four new backbones (in **32**, **35**, **37**, and **41**) generated by introducing strained rings at C11 of FPP (new products **34**, **36**, **37**, **42**, **42a**, and **46** bear the same skeletons as **32**, **37** and **41**, **42**, and **49**).

The two geminal methyl groups in FPP (**1**) generally remain unaltered in the end product when FPP serves as a substrate (see **27**–**30**). Only the connecting carbon atom at position 11 becomes part of the (oligo)­cyclic backbone. This changes in many cases when the cyclopropylidene or cyclobutylidene group is attached to the terminal alkene or if a nucleophilic oxygen atom is present in the strained oxetane unit.

In total, we describe 17 new terpenoids created by a chemoenzymatic approach with STSs as key players. Nine of these new terpenoids contain completely new backbones or are structurally unrelated to known natural sesquiterpenes, which is due to additional atoms being incorporated into the terpene backbone by the controlled rearrangements of the small rings.

However, with the new sesquiterpenoids **46**, **49**, and **50**, there are also some exceptions where the small rings have survived the cation cascade unscathed. Unlike under homogeneous chemical conditions, this reveals the strong conformational control generated by the protein template, which fixes the substrate in its global orientation.

For ring expansion, especially of cyclopropanes and cyclobutanes, the σ-bond involved in ring expansion must be oriented coplanar to the empty p-orbital of the neighboring cation. This conformational requirement appears to be blocked by the synthase during the formation of **46**, **49**, and **50**. This observation is a telling example of how terpene synthases can provide special insights into carbocation chemistry. With this work, we demonstrate once again that terpene synthases can contribute significantly to a deeper understanding of reactive carbocations and their chemistry, especially when unnatural substrates are used. The chiral, three-dimensional active pocket can be regarded as a chemical laboratory in which various cationic cascade sequences are possible that are much more comprehensive, diverse, and controlled than in classical chemical environments of synthetic chemistry.^28^ We are convinced that this space should be used by chemists to deepen their understanding of carbocations and their potential for cationic cascades in a defined environment.^29^ This could lead to the development of chemically generated three-dimensional architectures, as already elegantly demonstrated by Tiefenbacher and his colleagues, for example.^30^

## Supplementary Material



## Notes

Experimental procedures, characterizations, and analytical data of new compounds and NMR spectra (DOCX)

## Notes

Dedicated to Steven V. Ley (University of Cambridge, UK) on the occasion of his 80th birthday.
