# Supplementary material for: Introducing Small Rings into Farnesyl Pyrophosphates Paves the Way for the Enzymatic Generation of Unnatural Sesquiterpene Scaffolds
Source: J Am Chem Soc. 2026 Jan 30;148(5):5496–507. doi: 10.1021/jacs.5c19651 (PMC12903903; doi:10.1021/jacs.5c19651)
Supplement: Supplementary file 1 [file ja5c19651_si_001.docx]

Supporting Information

**Introducing small rings into farnesyl pyrophosphate paves the way for the enzymatic generation of unnatural sesquiterpene scaffolds**

Daghan Taser^a‡^, Catherine Victoria^a‡^, Leon von Garrel^a^, Jörn Droste^a^, Christopher Tabet^a^, Gerald Dräger^a^, Ahmed Hassanin^b,c‡^, Mehdi D. Davari^b^, Andreas Kirschning^a,d^*

^a^ Institute of Organic Chemistry, Leibniz University Hannover, Schneiderberg 1B, 30167 Hannover, Germany;

^b^ Department of Bioorganic Chemistry, Leibniz Institute of Plant Biochemistry (IPB), Weinberg 3, 06120 Halle Germany

^c^ Department of Pharmacognosy, Faculty of Pharmacy, Assiut University, 71526 Assiut, Egypt.

^d^ Uppsala Biomedical Center (BMC), University Uppsala, Husargatan 3, 752 37 Uppsala, Sweden.

* E-Mail: andreas.kirschning@oci.uni-hannover.de

[1. Materials and methods 2](#_Toc213081803)

[1.1 General information 2](#_Toc213081804)

[1.2 GC-MS assisted analysis of enzyme reaction 3](#_Toc213081805)

[1.3 Preparation of NMR samples 3](#_Toc213081806)

[1.4 Biologically relevant information and biochemical protocols 3](#_Toc213081807)

[1.4.1 Media and buffer 3](#_Toc213081808)

[1.4.2 Genes, strains, plasmids and growth conditions 4](#_Toc213081809)

[1.4.3 Protein purification 5](#_Toc213081810)

[1.4.4 Analytical biotransformation assay 5](#_Toc213081811)

[1.4.5 General Procedure: Scale-up biotransformation 5](#_Toc213081812)

[2. Experimental Procedures 7](#_Toc213081813)

[2.1 Chemical Syntheses 7](#_Toc213081814)

2.2 Biotransformation products 35

[3. Computational Section 52](#_Toc213081815)

[4. Abbreviations 69](#_Toc213081816)

[5. GC-MS Data 70](#_Toc213081817)

[5.1 GC-MS assays of sesquiterpene synthases used in scale-up biotransformations 70](#_Toc213081818)

[5.1.1 **4a** Biotransformation GC-MS assays 70](#_Toc213081819)

[5.1.2 **5a** Biotransformation GC-MS assays 73](#_Toc213081820)

[5.1.3 **4b** Biotransformation GC-MS assays 76](#_Toc213081821)

[5.1.4 **5b** Biotransformation GC-MS assays 79](#_Toc213081822)

[5.1.5 **4c** Biotransformation GC-MS assays 81](#_Toc213081823)

[5.1.6 **5c** Biotransformation GC-MS assays 84](#_Toc213081824)

[5.2 GC-MS assays collected from biotransformations not further upscaled 86](#_Toc213081825)

[5.2.1 **4a** Biotransformations with Hvs1, Tri5, Cyc1, Tps32 and GcoA 86](#_Toc213081826)

[5.2.2 **5a** Biotransformations with Hvs1, Tri5, Cyc1, Tps32 and GcoA 88](#_Toc213081827)

[5.2.3 **4b** Biotransformations with Hvs1, Tri5, Cyc1, Tps32 and GcoA 90](#_Toc213081828)

[5.2.4 **5b** Biotransformations with Hvs1, Tri5, Cyc1, Tps32 and GcoA 92](#_Toc213081829)

[5.2.5 **4c** Biotransformations with Hvs1, Tri5, Cyc1, Tps32 and GcoA 94](#_Toc213081830)

[5.2.6 **5c** Biotransformations with Hvs1, Tri5, Cyc1, Tps32 and GcoA 96](#_Toc213081831)

[5.3 GC chromatograms and mass spectra of new terpenoids isolated 98](#_Toc213081832)

[6. Copies of NMR spectra 114](#_Toc213081833)

[7. References 233](#_Toc213081834)

# Materials and methods

## General information

**^1^H, ^13^C, ^31^P, ^19^F and 2D-NMR** experiments were recorded using a Bruker AVANCE I (400 MHz) equipped with a DUL probe, Bruker AVANCE III HD (400 MHz) equipped with either a PRODIGY BBFO or BBO probe, Bruker AVANCE III HD (500 MHz) equipped with a TCI cryoprobe, and Bruker AVANCE NEO (600 MHz) equipped with DUL. Multiplicities are described using the following abbreviations: s = singlet, d = doublet, t = triplet, q = quartet, sex = sextet, m = multiplet, br = broad. Chemical shift values δ of ^1^H and ^13^C NMR spectra are reported in ppm relative to the residual solvent signal as an internal standard. Coupling constant *J* are reported in Hz. Data analysis was performed using Topspin from Bruker and MNova from Mestrelab Research. Structure elucidation of new products was assisted by ^1^H‑^1^H COSY, ^1^H-^13^C HMBC, ^1^H-^13^C HSQC, ^1^H-^13^C H2BC and ^1^H-^1^H NOESY, 2D-NMR experiments.

**HR-ESI-MS (Tof)** was measured with an Alliance 2695 HPLC (Waters) coupled to a LCT premier (Waters with a lock spray dual ion ESI source). For pyrophosphates high-resolution proceeded via Waters QTOF Premier (ESI−) injection in loop-mode without formic acid to prevent hydrolysis.

**Analytical thin-layer chromatography** was performed using precoated silica gel plates (Macherey-Nagel, Düren) and the spots were visualized with UV light at 254 nm or alternatively by staining with permanganate or vanillin. Flash column chromatography was performed using mesh silica by Macherey‑Nagel (grain size 40-63 μm), with the indicated solvent system assisted with compressed air.

**Commercially available reagents**, chromatography type or dry solvents were used as received or purified by standard techniques according to the literature.

**Melting points** were determined on a SRS OptiMelt apparatus and are not corrected.

**Molecule numbering** corresponds to the main manuscript if applicable and molecule names were generated using Chemdraw 21.0.0 by Perkin Elmer.

**Ion exchange polymer** Amberchrom^TM^ 50WX8 (hydrogen form 100-200 mesh) was inserted into a column and then exchanged to NH_4_^+^ form by rinsing with an aqueous 5% NH_3_ solution until the eluate was alkaline. The polymer was then washed with water until the eluate was neutral. Finally it was rinsed with an aqueous ion exchange buffer (2% isopropanol, 0.2% (w/v) NH_4_HCO_3_) until the eluate reached the same pH as the ion exchange buffer.

**Analytic GC/MS analyses** were carried out with an Agilent 7890B GC with 5977B GC/MSD and Gerstel MPS Robotic XL with KAS 4C injector. Samples were analysed on an Optima 5HT column, 30 m x 250 μm i.d. x film thickness 0.25 μm). Carrier gas, He; injector temp.: 60 °C to 300 °C at 12 °C/min, splitless or split ratio 1:40; temp. program: 50 °C (isothermal 1 min) to 300 °C, at 20 °C/min and held isothermal for 6.5 min at 300 °C; FID: 300 °C, H_2_: 30 mL/min, N_2_: 25 mL/min, MSD: ion source: EI 70 eV, 230 °C; detector: quadrupole, EI mass spectra were acquired over the mass range of 30 – 650 amu. Further GC/MS analyses were carried out with an Agilent GC 6890B with 5973 MSD and Gerstel MPS II with KAS injector. Samples were analysed on an Optima WAX column (30 m x 0.25 mm i.d. x film thickness 0.25 µm). Carrier gas, He; injector temp.: 50 °C to 250 °C at 12 °C/min; split ratio 1:40; temp. program: 60  °C (isothermal 1 min) to 240 °C at 9 °C/s held isothermal for 9 min at 240 °C; FID: 250 °C, H_2_: 40 mL/min, N_2_: 45 mL/min, MSD: ion source: EI 70 eV, 230 °C; detector: quadrupole.

**HR-GC/MS** analyses were carried out on a Waters GCT Premier mass spectrometer coupled with an Agilent 6890n GC with CTC CombiPAL sampler. Samples were analysed on an Optima 5HT column (30 m x 250 μm i.d. x film thickness 0.25 μm). Carrier gas, He; injector temp. 300 °C, split ratio 1:40; temp. program: 50 °C (isothermal 1 min) to 300 °C, at 20 °C/min and held isothermal for 6.5 min at 300 °C; FID: 300 °C, H_2_: 30 mL/min, N_2_: 25 mL/min, GCT-Premier: ion source: EI 70 eV, 250 °C; detector-voltage: 2500 V, EI mass spectra were acquired over the mass range of 20 – 800 amu. HRCI-MS was performed with a HP 6890 Series GC-system by Hewlett Packard.

**Preparative GC** was carried out on a HP 6890 Series GC-system by Hewlett Packard with an 7683 Series Injector by Hewlett Packard. Samples were analysed on an Optima 5HT column (30 m x 530 μm i.d. x film thickness 3.00 μm). Carrier gas, He; injector temp. 250 °C, max temp. 300 °C, splitless; FID: 250 °C, H_2_: 40 mL/min, N_2_: 40 mL/min. Analytes were collected automatically after gas chromatographic separation by an Gerstel preparative fraction collector equipped with Gerstel 100 μL sample traps sealed with Gerstel PTFE ferrules (3.00 mm).

## GC-MS assisted analysis of enzyme reaction

Products of the enzymatic reactions described in Section 5 were separated by Optima 5HT column. The temperature program is optimized for C-15 and C-16 terpenoids to have a retention time window of 9 – 13 min. TIC (total ion chromatogram) was used for MS analysis and FID (flame ion detector) for semi-quantification.

Analysis proceeded by calculating the intensity of analytes in comparison to the observed main product in the FID chromatogram. A peak area threshold of 5% relative to the main analyte was applied. Peaks that exceeded this value were integrated and included in the analysis. These were then further analysed based on their mass spectral fragmentation pattern. Only signals that included the expected mass spectral features resulting from elimination or hydrolysis (m/z for **4a**: 202 and 220, for **4b**: 216 and 234, for **4c**: 218 and 236, for **5a**: 218 and 236, for **5b**: 232 and 250, for **5c**: 234 and 252) were deemed significant for analysis and false positives filtered out manually. Relative abundances were then calculated based on peak areas and normalized to 100% for compositional comparison.

Isolation and elucidation of products proceeded by following the scale up biotransformation method described in Section 1.4.5. Products that were observed during silica column chromatography purification were isolated and elucidated.

## Preparation of NMR samples

The biotransformation product containing fractions collected by column chromatography were combined and concentrated to a volume of 50 μL using a light stream of argon gas. To avoid evaporation of the product, deuterated benzene (1 mL) was added to the vial. The mixture was again concentrated to a total volume of 50 μL using a light stream of argon gas. This process cools the mixture, which reduces the degree of removal of the volatile product to a minimum. This co-evaporation protocol using deuterated benzene is a mild way to remove non-deuterated solvents. Subsequently, deuterated benzene (1 mL) was again added to the vial. This time, the mixture was concentrated to approximately 650 mL and transferred to a NMR tube. This protocol allows to obtain samples suited for NMR measurements.

## Biologically relevant information and biochemical protocols

### **Media and buffer**

| 2TY media | 1.00% (w/v) yeast extract  1.60% (w/v) trypton  0.50% (w/v) NaCl | Analytic HEPES buffer | 50 mM HEPES  5 mM DTT  pH 7.6 |
| --- | --- | --- | --- |
| Lysis buffer | 40 mM Tris·HCl  100 mM NaCl  pH 8 | Scale-up HEPES buffer | 50 mM HEPES  5 mM DTT  50 mM NaCl  0.01% Tween20 |
| Ni-NTA buffer | 40 mM Tris·HCl  100 mM NaCl  imidazole (x M) | LB medium | 0.50% (w/v) yeast extract  1.00% (w/v) trypton  0.50% (w/v) |
| Preservation buffer | 20 mM Tris·HCl  20% glycerine  1 mM DTT  100 mM NaCl  pH 8.0 |  |  |

The pH was adjusted with an aqueous HCl solution (1 M) or an aqueous NaOH solution (1 M).

### **Genes, strains, plasmids and growth conditions**

The genes used in this work were synthesized by GENEWIZ LLC as a construct with pUC57 and are optimized for expression in *E. coli*. The fundamental protein sequence, used for the gene synthesis, is listed below. Cloning of sesquiterpene synthases was carried out in *E. coli* TOP10 with pET28a(+) plasmids, leading to a *N*-terminal polyhistidine tag for the enzyme through expression.

*E. coli* cultures were grown at 37 °C and 180 rpm in Luria-Bertani medium (LB medium) supplemented with 50 µg mL^−1^ kanamycine. For heterologous expression 2TY (2x yeast extract tryptone) medium supplemented with 50 µg mL^−1^ kanamycin was inoculated by 2% pre-culture of *E. coli* BL21 (DE3) and grown at 37 °C and 180 rpm to an optical density (OD_600_) of 0.6. Then, the expression controlled by the T7lac-promotor was induced by adding 0.5 mM IPTG (isopropyl-β thiogalactopyranoside) and the temperature was decreased to 16 °C for overnight cultivation. The medium was removed by centrifugation and the cells used immediately or stored at ‑20 °C.

Table S1. Gene Bank Accession Numbers of sesquiterpene synthases used in this work.

| Enzyme | Gene bank accession number | Reference |
| --- | --- | --- |
| BcBOT2 | Q6WP50 | [S1] |
| Omp7 | MUStwsD_GLEAN_10003831 | [S2] |
| PenA | AAA19131 | [S3] |
| Cop4 | A8NU13 | [S4] |
| Hvs1 | Q39978 | [S5] |
| Tri5 | P13513 | [S6] |
| Cyc1 | Q9K499 | [S7] |
| Tps32 | G5CV45 | [S8] |
| GcoA | B1W019 | [S9] |

### **Protein purification**

After overexpression of the sesquiterpene synthases as described above, the cells were harvested and resuspended in lysis buffer. The cells were disrupted by sonification (amplitude: 40%, on: 4 sec, off: 6 sec, total time: 20 min/100 mL culture) and centrifuged (8500 rpm, 20 min). The supernatant was subjected to an IMAC column (Protino® Ni-NTA-Agarose by Macherey-Nagel GmbH&Co. KG). The IMAC column was prepared by washing with water (10 x column volume) and lysis buffer (5 x column volume; no imidazole). The supernatant of the cell lysis was loaded two times. Impurities were washed off with lysis buffer (10 x column volume; 25 mM imidazole). The protein of interest was eluted NI-NTA buffer (3 x column volume; 250 mM imidazole), which was directly subjected to a PD10-desalting column (GE Healthcare) to remove imidazole. The desalting column was prepared by washing with water (2 x column volume) and lysis buffer (2 x column volume; no imidazole). All elution was collected and concentrated with Amicon Ultra-15 Centrifugal Filter Units, 30 kDa (Merck) by centrifugation (4500 rpm, 20 min). The concentrated protein was preserved by the addition of the same volume of preservation buffer (200 μL) and stored at −78 °C. Concentrations were measured on a DeNovix spectrometer.

### **Analytical biotransformation assay**

Analytical biotransformation assays were carried out in 500 μL scale. FPP derivative (150 μM) was incubated in analytic HEPES buffer (see Section 1.4.1) with the corresponding purified sesquiterpene synthase (0.1 g/L) and MgCl_2_ (5 mM). The reaction was incubated (37 °C, 30 min), followed by the addition of *n*-hexane (100 μL). The mixture was centrifuged (3000 rpm, 10 min) and the *n*‑hexane layer transferred to a GC-MS vial containing an inlet (200 μL) using an Eppendorf pipette (100 μL). The sample was then injected and analyzed (see Section 1.2).

The same procedure was done simultaneously with farnesyl pyrophosphate as a positive control for each enzyme. Furthermore, the same procedure was performed with the FPP derivative in the absence of the enzyme by just using the buffer system (negative control).

### **General Procedure: Scale-up biotransformation**

Scale-up biotransformations were carried out in 25 mL scale in a capped 50 mL Erlenmeyer flask. FPP derivative (1 mM) was added to scale‑up HEPES buffer (see Section 1.4.1), followed by the addition of PPase (1 U, to prevent enzyme inhibition by the by-product diphosphate), Tween20 (0.01% (v/v), to improve solubility) and the purified sesquiterpene synthase (0.1 g/L). The transformation was incubated (100 rpm, 37 °C, 24 h) and then extracted using a stir bar in a capped 100 mL Erlenmeyer flask with *n*-pentane (20 mL, 300 rpm, rt, 12 h). The layers were separated and the aqueous layer was extracted with *n*-pentane (3 x 20 mL). The collected organic layers were washed with brine (1 x), dried over Na_2_SO_4_, filtered and concentrated at 650 mbar without achieving full evaporation to minimize loss of product. The resulting crude product was purified by silica column chromatography (elution according to product as described in the experimental section, while Et_2_O was distilled before use to remove stabilizer such as BHT which would otherwise interfere with NMR analyses). The fractions were again concentrated at 650 mbar and 40 °C, without full evaporation and the NMR sample was then prepared as described in Section 1.3, which allowed for solvent free analysis – however limited the quantification and prevents specification of yields. If no sufficient purity was achieved after liquid silica column chromatography, the sample was further purified by means of preparative GC or semipreparative HPLC carried out with a L‑7100 and L-7170 pump, D 7000 interface and L-7455 diode array detector (λ = 200-350 nm) by Merck Hitachi and a VP 250/10 Nucleodur 100-5 CN-RP column (5 μm, 250 mm, Ø 10 mm) by Macherey-Nagel.

# Experimental Procedures

## Chemical Syntheses

#### (2*E*,6*E*)-3,7,11-Trimethyldodeca-2,6,10-trien-1-ol (SI-1)

|  |
| --- |

To a solution of farnesol (**6**, 20.0 g, 89.9 mmol, 1.00 eq.), Et_3_N (18.8 mL, 135 mmol, 1.50 eq.) and DMAP (220 mg, 1.80 mmol, 0.02 eq.) in dry CH_2_Cl_2_ (300 mL) at 0 °C was added Ac_2_O (11.0 mL, 117 mmol, 1.30 eq.) and stirred for 30 min at this temperature. Upon completion (judged by TLC) the reaction was terminated by addition of water. The phases were separated, and the organic phase washed with an aqueous HCl solution (1 M, 100 mL), a sat. aqueous NaHCO_3_ solution and brine. The organic layer was dried over MgSO_4_, filtered, concentrated *in vacuo* and the crude purified by silica column chromatography (SiO_2_; PE/EA 10:1) to furnish **SI-1** as a colorless oil (23.1 g, 87.4 mmol, 97%).

**TLC**: *R*_F_ = 0.80 (SiO_2_; PE/EA 10:1).

**^1^H NMR** (400 MHz, CDCl_3_) δ 5.34 (tt, *J* = 7.1, 1.3 Hz, 1H, H-2), 5.09 (dddt, *J* = 6.9, 4.2, 2.9, 1.3 Hz, 1H, H-3), 4.58 (dd, *J* = 7.2, 0.9 Hz, 2H, H-1), 2.14 – 1.94 (m, 8H, H-4, H-5, H-8 and H-9), 2.05 (s, 3H, OC(O)CH_3_), 1.70 (s, 3H, H-1’), 1.68 (s, 3H, H-2’), 1.60 (s, 6H, H-12 and H-13).

**^13^C NMR** (101 MHz, CDCl_3_) δ 171.3 (O*C*(O)CH_3_), 142.4 (C-3), 135.6 (C-7), 131.5 (C-11), 124.4 (C-6), 123.7 (C-10), 118.4 (C-2), 61.5 (C-1), 39.8 (C-4), 39.7 (C-8), 26.8 (C-5), 26.3 (C-9), 25.8 (C-12), 21.2 (O*C*(O)*C*H_3_), 17.8 (C-13), 16.6 (C-1’), 16.1 (C-2’).

The analytic data are in accordance with those reported in the literature.^S10^

#### (2*E*,6*E*)-3,7,11-Trimethyldodeca-2,6,10-trien-1-yl acetate (7)

|  |
| --- |

**Step 1: Epoxidation**

**SI-1** (15.0 g, 56.7 mmol, 1.00 eq.) was dissolved in dry CH_2_Cl_2_ (1.13 L) and cooled to 0 °C. To the solution was added *m*CPBA (77%, 17.8 g, 79.4 mmol, 1.40 eq.) in two portions (in a 15 min interval) then allowed to warm up to rt and the mixture was stirred for 1 h. The reaction was terminated by the addition of an aqueous NaOH solution (1 M). The phases were separated, and the aqueous layer was extracted with CH_2_Cl_2_ (3 x), the combined organic phases were dried over Na_2_SO_4_, filtered and the solvent was removed under reduced pressure. The crude epoxide was used in the next step without further purification.

**Step 2: Oxidative cleavage to aldehyde**

The crude epoxide was dissolved in a mixture of THF/H_2_O (2:1, 142 mL) and H_5_IO_6_ (18.1 g, 79.4 mmol, 1.40 eq.) added at 0 °C and stirred for 1 h at the same temperature. To the mixture was added brine and the aqueous phase extracted with EA (3 x). The organic solvent was dried over MgSO_4_, filtered, and concentrated under reduced pressure. The resulting oil was subjected to flash chromatography (SiO_2_; PE/EA 9:1) and title compound **7** was isolated as a pale yellow oil (3.53 g, 14.8 mmol, 26% o2s).

**TLC**: *R*_F_ = 0.23 (SiO_2_; PE/EA 10:1).

**^1^H NMR** (400 MHz, CDCl_3_) δ 9.74 (s, 1H, H-10), 5.32 (tq, *J* = 7.1, 1.4 Hz, 2H, H-2), 5.12 (tt, *J* = 5.6, 1.6 Hz, 1H, H-6), 4.58 (d, *J* = 7.1 Hz, 2H, H-1), 2.51 (td, *J* = 7.5, 1.9 Hz, 2H, H-9), 2.31 (t, *J* = 7.5 Hz, 2H, H-8), 2.15 – 2.03 (m, 4H, H-4 and H-5), 2.05 (s, 3H, OC(O)CH_3_) 1.69 (s, 3H, H‑1’), 1.61 (s, 3H, H‑2’).

**^13^C NMR** (101 MHz, CDCl_3_) δ 202.8 (C-10), 171.3 (O*C*(O)CH_3_), 142.0 (C-3), 133.6 (C-7), 124.9 (C-6), 118.6 (C‑2), 61.5 (C‑1), 42.2 (C‑9), 39.4 (C‑4), 31.9 (C‑8), 26.2 (C‑5), 21.2 (OC(O)*C*H_3_), 16.6 (C‑1’), 16.3 (C‑2’).

The analytic data are in accordance with those reported in the literature.^S10^

#### (2*E*,6*E*)-10-Cyclopropylidene-3,7-dimethyldeca-2,6-dien-1-yl acetate (SI-2)

|  |
| --- |

**Procedure 1: Wittig olefination**

Cyclopropyltriphenylphosphonium bromide (**8a**, 5.31 g, 13.9 mmol, 1.40 eq.) was added to a suspension of NaH (90%, 554 mg, 13.9 mmol, 1.40 eq.) in dry THF (44 mL). The mixture was stirred under refluxing conditions for 16 h and a solution of aldehyde **7** (2.38 g, 10.0 mmol, 1.00 eq.) in dry THF (11 mL) was added to the orange suspension. The reaction mixture was stirred for another 7 h at this temperature and the reaction was terminated by the addition of water at rt. After extraction with Et_2_O (3 x) the combined organic layers were washed with a solution of 40% MeOH in a semisat. aqueous NH_4_Cl solution, water, brine, then dried over Na_2_SO_4_, filtered and concentrated *in vacuo*. The residue was purified by column chromatography (SiO_2_; PE/EA 70:1) to afford the title compound **SI-2** (719 mg, 2.74 mmol, 28%) as a colorless oil.

**Procedure 2: Julia-Kocienski olefination**

Aldehyde **7** (500 mg, 2.10 mmol, 1.00 eq.) and sulfone **8b** (2.05 g, 6.29 mmol, 3.00 eq.) were dissolved in dry THF/DMF (3:1, 7.0 mL) and Cs_2_CO_3_ (2.74 g, 8.40 mmol, 4.00 eq.) was added. The orange suspension was stirred at 70 °C for 3 d. The reaction was terminated by the addition of a sat. aqueous NH_4_Cl solution. The resulting aqueous solution was extracted with EA (3 x) and the combined, organic layers were washed with brine and dried over Na_2_SO_4_, filtered, and concentrated *in vacuo.* The crude material was purified by column chromatography (SiO_2_; PE/EA 10:1) and **SI-2** was collected as a colorless oil (125 mg, 476 μmol, 23%).

**TLC**: *R*_F_ = 0.59 (SiO_2_; PE/EA 10:1).

**^1^H NMR** (400 MHz, CDCl_3_) δ 5.73 (tp, *J* = 6.3, 2.1 Hz, 1H, H‑10), 5.34 (tp, *J* = 7.0, 1.3 Hz, 1H, H‑2), 5.11 (tq, *J* = 7.0, 1.4 Hz, 1H, H‑6), 4.58 (d, *J* = 7.4 Hz, 1H, H‑1) 2.32 – 2.22 (m, 2H, H‑9), 2.16 – 2.02 (m, 6H, H‑4, H‑5 and H‑8), 2.05 (s, 3H, OC(O)CH_3_), 1.70 (s, 3H, H‑1’), 1.61 (s, 3H, H‑2’), 1.06 – 0.96 (m, 4H, H‑12 and H‑13).

**^13^C NMR** (101 MHz, CDCl_3_) δ 171.3 (O*C*(O)CH_3_), 142.4 (C‑3), 135.5 (C‑7), 123.8 (C‑6), 121.2 (C‑11), 118.4 (C‑2), 118.1 (C‑11), 61.5 (C‑1), 39.6 (C‑4), 39.5 (C‑8), 30.6 (C‑9), 26.3 (C‑5), 21.2 (O*C*(O)*C*H_3_), 16.6 (C‑1’), 16.1 (C‑2’), 2.3 (C‑12 or C‑13), 1.9 (C‑13 or C‑12).

The analytic data are in accordance with those reported in the literature.^S11^

#### (2*E*,6*E*)-10-Cyclopropylidene-3,7-dimethyldeca-2,6-dien-1-ol (9)

|  |
| --- |

To a solution of acetate **SI-2** (70.0 mg, 267 μmol, 1.00 eq.) in MeOH (3.6 mL) was added K_2_CO_3_ (36.9 mg, 267 μmol, 1.00 eq.) and the mixture was stirred for 1 h. Then, the reaction was terminated by addition of brine and the solution was extracted with Et_2_O (3 x). The combined organic layers were washed with brine, dried over Na_2_SO_4_, filtered and concentrated under reduced pressure. After purification by column chromatography (SiO_2_; PE/EA 4:1) alcohol **9** was isolated as a colorless oil (50.9 mg, 231 μmol, 87%).

**TLC**: *R*_F_ = 0.13 (SiO_2_; PE/EA 6:1).

**^1^H NMR** (400 MHz, CDCl_3_) δ 5.73 (tp, *J* = 6.3, 2.1 Hz, 1H, H-10), 5.42 (tq, *J* = 6.9, 1.3 Hz, 1H, H‑2), 5.17 – 5.08 (m, 1H, H‑6), 4.15 (dd, *J* = 6.9, 0.7 Hz, 2H, H‑1), 2.32 – 2.21 (m, 2H, H‑9), 2.15 – 2.08 (m, 4H, H‑5 and H‑8), 2.07 – 2.00 (m, 2H, H‑4), 1.68 (s, 3H, H‑1‘), 1.61 (s, 3H, H‑2‘), 1.31 (br. s, 1H, OH), 1.01 (m, 4H, H‑12 and H‑13).

**^13^C NMR** (101 MHz, CDCl_3_) δ 140.0 (C‑3), 135.4 (C‑7), 124.0 (C‑6), 123.5 (C‑2), 121.2 (C‑11), 118.1 (C‑10), 59.6 (C‑1), 39.7 (C‑4), 39.5 (C‑8), 30.6 (C‑9), 26.4 (C‑5), 16.4 (C‑1‘), 16.1 (C‑2‘), 2.3 (C‑12 or C‑13), 2.0 (C‑13 or C‑12).

The analytic data are in accordance with those reported in the literature.^S11^

#### Triammonium (2*E*,6*E*)-10-cyclopropylidene-3,7-dimethyldeca-2,6-dien-1-yl diphosphate (4a)

|  |
| --- |

**Step 1: Chlorination**

Allyl alcohol **9** (109 mg, 495 μmol, 1.00 eq.) was dissolved in dry DMF (21 mL), 2,4,6-collidine (393 μl, 2.97 mmol, 6.00 eq.) was added and the solution was cooled to 0 °C. To the reaction mixture was added MsCl (77.1 μL, 989 μmol, 2.00 eq.) and stirring was continued for 25 min at this temperature. Upon addition of LiCl (83.9 mg, 1.98 mmol, 4.00 eq.) the reaction was stirred for 2 h at 0 °C and the reaction was terminated by the addition of water. The aqueous phase was extracted with hexane (3 x) and the combined, organic layers were washed with a sat. aqueous NaHCO_3_ solution and brine. The organic layer was dried over MgSO_4_, filtered, and concentrated under reduced pressure. The crude orange oil was used for the next step without further purification.

**Step 2: Pyrophosphorylation**

To a suspension of (*n*-Bu_4_N)_3_P_2_O_7_ (669 mg, 741 μmol, 1.50 eq.) in dry CH_3_CN (1.4 mL) was added the crude allyl chloride of the previous step which was dissolved in dry CH_3_CN (800 μL) at 0 °C. The mixture was allowed to reach rt and stirring was continued overnight. Subsequently, the solvent was removed under reduced pressure and the remaining solid was dissolved in IEB (2 mL). The solution was passed through an Amberchrom^TM^ 50WX8 Ion Exchange Column (NH_4_^+^ form). The combined aqueous fractions containing the product were concentrated *in vacuo* and the resulting crude solids were dissolved in NH_4_HCO_3_ (1 M, 2 mL). A mixture of CH_3_CN/IPA (1:1, 8 mL) was added and the suspension was subjected to centrifugation (4500 rpm, 4 °C, 5 min). The supernatant was separated and subjected to the same procedure. This procedure was repeated three times in total. The resulting supernatant was concentrated and lyophilized overnight to deliver pyrophosphate **4a** as a pale yellow solid (108 mg, 250 μmol, 51% o2s).

**^1^H NMR** (400 MHz, D_2_O) δ 5.82 (tp, *J* = 6.3, 2.0 Hz, 1H, H‑10), 5.48 (tq, *J* = 7.2, 1.3 Hz, 1H, H‑2), 5.25 (ddt, *J* = 7.0, 5.7, 1.4 Hz, 1H, H‑6), 4.51 (t, *J* = 6.8 Hz, 2H, H‑1), 2.40 – 2.30 (m, 2H, H‑9), 2.22 – 2.10 (m, 6H, H‑4, H‑5 and H‑8), 1.74 (d, *J* = 1.3 Hz, 3H, H‑1’), 1.66 (d, *J* = 1.3 Hz, 3H, H‑2’), 1.10 – 1.00 (m, 4H, H‑12 and H‑13).

**^13^C NMR** (101 MHz, D_2_O) δ 143.0 (C‑3), 136.7 (C‑7), 124.2 (C‑6), 122.3 (C‑11), 119.5 (d, *J* = 8.2 Hz, C‑2), 118.1 (C‑10), 62.9 (d, *J* = 5.5 Hz, C‑1), 38.8 (C‑4 or C‑5), 38.4 (C‑5 or C‑4), 29.4 (C‑9), 25.5 (C‑8), 15.5 (C‑1’), 15.2 (C‑2’), 1.6 (C‑12 or C‑13), 1.2 (C‑13 or C‑12).

**^31^P NMR** (162 MHz, D_2_O) δ −9.9 (d, *J* = 20.9 Hz), −10.4 (d, *J* = 20.8 Hz).

The analytic data are in accordance with those reported in the literature.^S11^

#### 1-(*tert*-Butyl)-1*H*-tetrazole-5-thiol (SI-3)

|  |
| --- |

NaN_3_ (1.41 g, 21.7 mmol, 1.00 eq.) was dissolved in H_2_O (6.8 mL) and heated under refluxing conditions. To the solution was added dropwise 2‑isothiocyanato‑2‑methylpropane (2.75 mL, 21.7 mmol, 1.00 eq.) dissolved in IPA (2.8 mL) and stirring was continued at this temperature overnight. The mixture was cooled to 0 °C and a concentrated, aqueous HCl solution (3.23 mL, 39.1 mmol, 1.80 eq,) was added slowly. The IPA was removed under reduced pressure and the remaining mixture was stored at 7 °C overnight. The pale yellow solid was filtered, washed with ice cold water and the thiol was lyophilized which provided thiol **SI-3** (2.86 g, 18.1 mmol, 83%).

**TLC**: *R*_F_ = 0.78 (SiO_2_; PE/EA 1:1).

**^1^H NMR** (400 MHz, CDCl_3_) δ 1.88 (s, 9H, NC_q_(C*H*_3_)_3_).

**^13^C NMR** (101 MHz, CDCl_3_) δ 163.0 (C_q_S), 63.7 (N*C*_q_(CH_3_)_3_), 27.7 (NC_q_(*C*H_3_)_3_).

The analytic data are in accordance with those reported in the literature.^S12^

#### 5-((3-Bromopropyl)thio)-1-(*tert*-butyl)-1*H*-tetrazole (SI-4)

|  |
| --- |

To thiol **SI-3** (200 mg, 1.26 mmol, 1.00 eq.) dissolved in absolute EtOH (25 mL) was added *t*-BuOK (142 mg, 1.26 mmol, 1.00 eq.). The suspension was stirred at rt for 15 min and the solvent was evaporated *in vacuo*. The residue was dissolved in DCE (8.4 mL) and TBAI (46.5 mg, 126 μmol, 0.1 eq.) was added. To the mixture was added 1,3-dibromopropane (129 μL, 1.26 mmol, 1.00 eq.) dropwise and the resulting solution was stirred at rt overnight. The solvent was evaporated under reduced pressure, the remaining solids were dissolved in EA and the organic phase was washed with an aqueous Na_2_CO_3_ solution (10%) and brine. The organic layer was then dried over Na_2_SO_4_, filtered, concentrated under reduced pressure and the title compound **SI-4** was isolated after column chromatography (SiO_2_; PE/EA 8:1) as a white solid (190 mg, 681 μmol, 54%).

**TLC**: *R*_F_ = 0.81 (SiO_2_; PE/EA 1:1).

**^1^H NMR** (400 MHz, CDCl_3_) δ 3.53 (dt, *J* = 10.0, 6.6 Hz, 4H, C*H*_2_Br and C*H*_2_S), 2.41 (p, *J* = 6.6 Hz, 2H, CH_2_), 1.72 (s, 9H, NC_q_(C*H*_3_)_3_).

**^13^C NMR** (101 MHz, CDCl_3_) δ 152.2 (C_q_S), 61.2 (N*C*_q_(CH_3_)_3_), 36.0 (N*C*_q_(CH_3_)_3_). 32.1 (CH_2_S), 31.7 (CH_2_Br), 28.9 (NC_q_(*C*H_3_)_3_).

The analytic data are in accordance with those reported in the literature.^S13^

#### 1-(*tert*-Butyl)-5-(cyclopropylthio)-1*H*-tetrazole (8b)

|  |
| --- |

**Step 1: Oxidation**

Tetrazole **SI-4** (1.45 g, 5.19 mmol, 1.00 eq.) was dissolved in absolute EtOH (9.3 mL) and hexaammonium heptamolybdate tetrahydrate (121 mg, 104 μmol, 0.02 eq.) was added at 0 °C. Subsequently, aqueous H_2_O_2_ (50%, 1.27 mL, 20.8 mmol, 4.00 eq.) was added dropwise at the same temperature and the reaction mixture was allowed to reach rt. After 1 d water and CH_2_Cl_2_ in equal parts were added to the yellow solution, and the aqueous phase was extracted with CH_2_Cl_2_ (3 x). The combined organic layers were washed with a sat. aqueous Na_2_S_2_O_3_ solution, brine, dried over MgSO_4_, filtered and concentrated under reduced pressure.

**Step 2: Ring closure**

To crude sulfone in dry CH_3_CN (17 mL) was added Cs_2_CO_3_ (6.77 g, 20.8 mmol, 4.00 eq.) and the suspension was stirred for 3 d at rt. The solvent was evaporated *in vacuo* and the residue was purified by flash column chromatography (SiO_2_; PE/EA 10:1 to 3:2) and **8b** isolated as a white solid (752 mg, 3.27 mmol, 63%).

**TLC**: *R*_F_ = 0.38 (SiO_2_; PE/EA 5:1).

**^1^H NMR** (400 MHz, CDCl_3_) δ 3.28 (tt, *J* = 7.9, 4.8 Hz, 1H, SC*H*), 1.85 (s, 9H, NC_q_(C*H*_3_)_3_), 1.50 (ddd, *J* = 4.2, 2.9, 1.9 Hz, 2H, CH_2_), 1.43 – 1.34 (m, 2H, CH_2_).

**^13^C NMR** (101 MHz, CDCl_3_) δ 154.9 (C_q_S), 65.4 (N*C*_q_(CH_3_)_3_), 33.4 (SCH), 29.9 (N*C*_q_(CH_3_)_3_), 7.1 ((CH_2_)_2_).

The analytic data are in accordance with those reported in the literature.^S13^

#### (*E*)-3,7-Dimethylocta-2,6-dien-1-yl acetate (SI-5)

4-DMAP (353 mg, 2.89 mmol, 0.02 eq.) and Et_3_N (30.2 mL, 217 mmol, 1.50 eq.) were added to a solution of geraniol (**10**, 22.3 g, 145 mmol, 1.00 eq.) in CH_2_Cl_2_ (482 mL). The solution was cooled to 0 °C. Ac_2_O (17.7 mL, 188 mmol, 1.30 eq.) was slowly added to the reaction mixture at this temperature and stirring was continued for 30 min. Then the reaction was terminated by the addition of water. The phases were separated, and the organic phase was washed with an aqueous HCl solution (1 M), a sat. aqueous NaHCO_3_ solution and brine. The organic layer was dried over MgSO_4_, filtered, concentrated *in vacuo* and the crude product was purified by column chromatography (SiO_2_; PE/EA 10:1) to furnish **SI-5** as a colorless oil (28.4 g, 145 mmol, quant.).

**TLC**: *R*_F_ = 0.47 (SiO_2_; PE/EA 20:1).

**^1^H NMR** (400 MHz, CDCl_3_) δ 5.45 – 5.29 (m, 1H, H-2), 5.10 (ddt, *J* = 6.8, 5.4, 1.4 Hz, 1H, H-6), 4.61 (d, *J* = 7.1 Hz, 2H, H-1), 2.16 – 2.03 (m, 4H, H-4 and H-5), 2.07 (s, 3H, OC(O)CH_3_), 1.72 (s, 3H, H-1’), 1.70 (s, 3H, H-2’), 1.62 (s, 3H, H-3’).

**^13^C NMR** (101 MHz, CDCl_3_) δ 171.3 (O*C*(O)CH_3_), 142.4 (C-3), 132.0 (C-7), 123.9 (C-6), 118.4 (C-2), 61.5 (C-1), 39.7 (C-4), 26.4 (C-5), 25.8 (C-2’), 21.2 (OC(O)*C*H_3_), 17.8 (C-3’), 16.6 (C-1’).

The analytic data are in accordance with those reported in the literature.^S14^

#### (*E*)-5-(3,3-Dimethyloxiran-2-yl)-3-methylpent-2-en-1-yl acetate (SI-6)

**SI-5** (11.1 g, 56.6 mmol, 1.00 eq.) was dissolved in dry CH_2_Cl_2_ (115 mL) and cooled to 0 °C. To the solution was added *m*CPBA (77%, 15.2 g, 67.9 mmol, 1.20 eq.) in two portions in a 15 min interval, then allowed to warm to rt and stirred for 1 h. The reaction was terminated by the addition of an aqueous NaOH solution (1 M). The phases were separated, and the aqueous layer was extracted with CH_2_Cl_2_ (3 x), the combined organic phases were dried over Na_2_SO_4_, filtered and the solvent was removed under reduced pressure. The crude product was purified by column chromatography (SiO_2_; PE/EA 10:1) to yield epoxide **SI-6** as a colorless oil (10.1 g, 47.4 mmol, 84%).

**TLC**: *R*_F_ = 0.50 (SiO_2_; PE/EA 10:1).

**^1^H NMR** (400 MHz, CDCl_3_) δ 5.38 (tq, *J* = 7.1, 1.3 Hz, 1H, H-2), 4.59 (d, *J* = 7.1 Hz, 2H, H-1), 2.70 (t, *J* = 6.2 Hz, 1H, H-6), 2.29 – 2.09 (m, 2H, H-4), 2.05 (s, 3H, OC(O)CH_3_), 1.72 (s, 3H, H-1’), 1.69 – 1.63 (m, 2H, H-5), 1.30 (s, 3H, H-2’ or H-3’), 1.26 (s, 3H, H-3’ or H-2’).

**^13^C NMR** (101 MHz, CDCl_3_) δ 171.2 (O*C*(O)CH_3_), 141.4 (C-3), 119.0 (C-2), 64.1 (C-6), 61.4 (C-1), 58.5 (C‑7), 36.3 (C-4), 27.2 (C-5), 25.0 (C-3’), 21.2 (OC(O)*C*H_3_), 18.9 (C-2’), 16.6 (C-1’).

The analytic data are in accordance with those reported in the literature.^S15^

#### (*E*)-3-Methyl-6-oxohex-2-en-1-yl acetate (11)

To a solution of epoxide **SI-6** (10.0 g, 47.1 mmol, 1.00 eq.) in THF/H_2_O (3:2, 120 mL) was added H_5_IO_6_ (14.0 g, 47.1 mmol, 1.00 eq.) at 0 °C. The reaction mixture was stirred for 1 d and the reaction was terminated by the addition of brine. After extraction with Et_2_O (3 x), the combined organic phases were dried over MgSO_4_, filtered and concentrated *in vacuo*. The crude product was purified by column chromatography (SiO_2_; PE/EA 5:1) to yield aldehyde **11** as a yellow oil (6.03 g, 35.4 mmol, 75%).

**TLC**: *R*_F_ = 0.28 (SiO_2_; PE/EA 5:1).

**^1^H NMR** (400 MHz, CDCl_3_) δ 9.77 (t, *J* = 1.6 Hz, 1H, H-6), 5.42 – 5.29 (m, 1H, H-2), 4.57 (dd, *J* = 7.0, 0.8 Hz, 2H, H-1), 2.60 – 2.54 (m, 2H, H-5), 2.40 – 2.34 (m, 2H, H-4), 2.04 (s, 3H, OC(O)CH_3_), 1.72 (s, 3H, H-1’).

**^13^C NMR** (101 MHz, CDCl_3_) δ 201.8 (C-6), 171.2 (O*C*(O)CH_3_), 140.1 (C-3), 119.4 (C-2), 61.2 (C-1), 41.8 (C‑4), 31.6 (C-5), 21.1 (OC(O)*C*H_3_), 16.7 (C-1’).

The analytic data are in accordance with those reported in the literature.^S15^

#### (*E*)-6-Cyclopropylidene-3-methylhex-2-en-1-ol (12)

**Step 1: Wittig olefination**

To a suspension of cyclopropyltriphenylphosphonium bromide (**8a**, 6.26 g, 16.3 mmol, 1.40 eq.) in freshly distilled THF (51 mL) was added NaH (90%, 410 mg, 15.2 mmol, 1.30 eq.). The mixture was stirred at 75 °C for 4 h and stirring continued overnight at 55 °C to give an orange suspension. TDA-1 (530 μL, 1.65 mmol, 0.14 eq.) was added to the suspension, followed by the slow addition of aldehyde **11** (2.00 g, 11.8 mmol, 0.05 mL/min, neat, 1.00 eq.) via a syringe pump. The mixture was stirred at the same temperature for 3 h and upon completion as judged by TLC, terminated at rt by the addition of water. The layers were separated and the aqueous phase was extracted with Et_2_O (3 x). The combined organic phases were washed with 40% MeOH in a half saturated NH_4_Cl solution, water, brine, dried over MgSO_4_ and concentrated *in vacuo*. The resulting cyclopropylidene acetate was purified by column chromatography (SiO_2_; PE/EA 20:1) and collected as a light yellow oil (1.15 g, 5.92 mmol).

**TLC**: *R*_F_ = 0.62 (SiO_2_; PE/EA 10:1).

**Step 2: Acetate deprotection**

To a solution of the acetate (1.15 g, 5.92 mmol, 1.00 eq.) from above dissolved in MeOH (17 mL) was added K_2_CO_3_ (818 mg, 5.92 mmol, 1.00 eq.) and the mixture was stirred for 1 h. In the following, the reaction was terminated by the addition of brine and the solution was extracted with Et_2_O (3 x). The combined, organic layers were washed with brine, dried over Na_2_SO_4_, filtered and concentrated under reduced pressure. After purification by column chromatography (SiO_2_; PE/EA 4:1) alcohol **12** was isolated as a colorless oil (741 mg, 4.91 mmol, 42% o2s).

**TLC**: *R*_F_ = 0.35 (SiO_2_; PE/EA 4:1).

**^1^H NMR** (400 MHz, CDCl_3_) δ 5.76 (tp, *J* = 6.1, 1.9 Hz, 1H, H‑6), 5.46 (tq, *J* = 6.9, 1.3 Hz, 1H, H‑2), 4.17 (d, *J* = 6.9 Hz, 2H, H‑1), 2.39 – 2.29 (m, 2H, H‑5), 2.22 – 2.15 (m, 2H, H‑4), 1.71 (s, 3H, H‑1’), 1.07 – 1.00 (m, 4H, H‑8 and H‑9).

**^13^C NMR** (101 MHz, CDCl_3_) δ 139.9 (C‑3), 123.5 (C‑2), 121.6 (C‑7), 117.7 (C‑6), 59.6 (C‑1), 39.3 (C‑4), 30.2 (C‑5), 16.4 (C‑1’), 2.3 (C‑8 or C‑9), 2.0 (C‑9 or C‑8).

**CI-MS** (CI+) m/z Calculated for C_10_H_15_O [M] 151.1123; Found 151.1131.

#### *tert-*Butyl(((*E*)-4-(((*E*)-6-cyclopropylidene-3-methylhex-2-en-1-yl)oxy)-3-methylbut-2-en-1-yl)oxy)diphenylsilane (SI-7)

Alcohol **12** (200 mg, 1.31 mmol, 1.00 eq.) in dry THF (1.3 mL) was added dropwise to a suspension of NaH (90%, 69.9 mg, 2.62 mmol, 2.00 eq.) in dry THF (1.3 mL). The mixture was stirred under refluxing conditions for 2 h. To the brown suspension was added TBAI (16.1 mg, 43.7 μmol, 0.03 eq.) and bromide **13** (636 mg, 1.58 mmol, 1.20 eq.) in dry THF (500 μL) at rt and stirring was continued under refluxing conditions for 16 h. The reaction was terminated at rt by the addition of an aqueous HCl solution (1 M). The aqueous phase was extracted with Et_2_O (5 x). The combined organic phases were washed with brine, dried over MgSO_~~4~~_, filtered and concentrated *in vacuo*. The crude material was purified by column chromatography (SiO_2_; PE/EA 100:1 then 10:1 then 2:1) to yield title compound **SI‑7** as a clear yellow oil (390 mg, 821 μmol, 69%).

**TLC**: *R*_F_ = 0.55 (SiO_2_; PE/EA 10:1).

**^1^H NMR** (400 MHz, CDCl_3_) δ 7.76 – 7.59 (m, 4H, ArH), 7.42 – 7.34 (m, 6H, ArH), 5.75 (tp, *J* = 6.3, 2.1 Hz, 1H, H‑11), 5.64 (ddt, *J* = 6.1, 4.8, 1.3 Hz, 1H, H‑2), 5.36 (tq, *J* = 6.8, 1.3 Hz, 1H, H‑7), 4.27 (dd, *J* = 6.1, 1.0 Hz, 2H, H‑1), 3.91 (d, *J* = 6.8 Hz, 1H, H‑6), 3.82 (s, 2H, H‑4), 2.36 – 2.27 (m, 2H, H‑10), 2.20 – 2.13 (m, 2H, H‑9), 1.67 (s, 3H, H‑2’), 1.49 (d, *J* = 1.2 Hz, 3H, H‑1’), 1.04 (s, 9H, SiC_q_(C*H*_3_)_3_), 1.03 – 1.01 (m, 4H, H‑13 and H‑14).

**^13^C NMR** (101 MHz, CDCl_3_) δ 140.1 (C‑8), 135.7 (ArC), 134.0 (C‑3), 133.9 (ArC_q_), 129.7 (ArC), 127.8 (ArC), 127.3 (C‑2), 121.5 (C‑12), 121.2 (C‑7), 117.8 (C‑11), 75.5 (C‑4), 66.1 (C‑6), 60.9 (C‑1), 39.4 (C‑9), 30.3 (C‑10), 27.0 (SiC_q_(*C*H_3_)_3_), 19.3 (Si*C*_q_(CH_3_)_3_), 16.6 (C‑2’), 14.2 (C‑1’), 2.3 (C‑13 or C‑14), 2.0 (C‑14 or C‑13).

**ESI-MS** (ESI+) m/z Calculated for C_31_H_42_O_2_NaSi [M+Na]^+^ 497.2852; Found 497.2868.

#### (*E*)-4-(((*E*)-6-Cyclopropylidene-3-methylhex-2-en-1-yl)oxy)-3-methylbut-2-en-1-ol (14)

To silyl ether **SI-7** (390 mg, 821 μmol, 1.00 eq.) in dry THF (8.0 mL) was added TBAF (1 M in THF, 2.46 mL, 2.46 mmol, 3.00 eq.) at 0 °C and the reaction mixture was stirred at rt for 1 d. The reaction was terminated by the addition of a sat. aqueous NH_4_Cl solution and the layers were separated. The aqueous phase was extracted with EA (3 x) and the combined organic layers were washed with brine, dried over Na_2_SO_4_, filtered and concentrated under reduced pressure. The residue was purified by flash column chromatography (SiO_2_; PE/EA 4:1) to yield alcohol **14** as a yellow oil (157 mg, 664 μmol, 81%).

**TLC**: *R*_F_ = 0.19 (SiO_2_; PE/EA 4:1).

**^1^H NMR** (400 MHz, CDCl_3_) δ 5.79 – 5.70 (m, 1H, H‑12), 5.71 – 5.61 (m, 1H, H‑2), 5.43 – 5.31 (m, 1H, H‑7), 4.21 (d, *J* = 6.7 Hz, 2H, H‑1), 3.95 (d, *J* = 6.9 Hz, 2H, H‑6), 3.85 (s, 2H, H‑4), 2.35 – 2.24 (m, 2H, H‑10), 2.24 – 2.11 (m, 2H, H‑9), 1.70 (s, 3H, H‑1’), 1.67 (s, 3H, H‑2’), 1.04 – 0.94 (m, 4H, H‑13 and H‑14).

**^13^C NMR** (101 MHz, CDCl_3_) δ 140.3 (C‑8), 136.3 (C‑3), 126.0 (C‑2), 121.5 (C‑12), 121.0 (C‑7), 117.8 (C‑11), 75.3 (C‑4), 66.6 (C‑6), 59.3 (C‑1), 39.4 (C‑9), 30.2 (C‑10), 16.6 (C‑2’), 14.2 (C‑1’), 2.3 (C‑13 or C‑14), 2.0 (C‑14 or C‑13).

**ESI-MS** (ESI+) m/z Calculated for C_15_H_24_O_2_Na [M+Na]^+^ 259.1674; Found 259.1678.

#### Triammonium (*E*)-4-(((*E*)-6-cyclopropylidene-3-methylhex-2-en-1-yl)oxy)-3-methylbut-2-en-1-yl diphosphate (5a)

**Step 1: Bromination**

To a solution of **14** (80 mg, 338 μmol, 1.00 eq.) in dry Et_2_O (4 mL) was added PBr_3_ (12.8 μL, 135 μmol, 0.40 eq.) in two portions at 0 °C. The mixture was stirred at rt for 30 min between the addition steps. Upon completion the reaction mixture was diluted with Et_2_O and brine. The layers were separated and the aqueous layer was extracted with Et_2_O (3 x). The combined, organic phases were washed with water and brine, dried over MgSO_4_, filtered and concentrated *in vacuo*. The crude product was purified by column chromatography (SiO_2_, PE/EA 4:1) to give the crude bromide as a colorless oil (54.0 mg, 180 μmol).

**TLC**: *R*_F_ = 0.65 (SiO_2_; PE/EA 20:1).

**Step 2: Pyrophosphorylation**

To a solution of (*n*-Bu_4_N)_3_P_2_O_7_ (316 mg, 271 μmol, 1.50 eq.) in dry CH_3_CN (4.0 mL) was added the bromide from above (54.0 mg, 180 μmol, 1.00 eq.) in the presence of 3 Å molecular sieves at 0 °C. The mixture was allowed to reach rt and stirring was continued overnight. Subsequently, the solvent was removed under reduced pressure and the remaining solid was dissolved in IEB (3 mL). The solution was passed through an Amberchrom^TM^ 50WX8 Ion Exchange Column (NH_4_^+^ form). The aqueous, fractions that contained the product were combined and concentrated *in vacuo* and the resulting crude solids were dissolved in a NH_4_HCO_3_ solution (1 M, 2 mL). A mixture of CH_3_CN/IPA (1:1, 8 mL) was added and the suspension was subjected to centrifugation (4500 rpm, 4 °C, 5 min). The supernatant was separated and subjected to the same procedure. This protocol was repeated three times in total. The resulting supernatant was concentrated and lyophilized overnight to deliver pyrophosphate **5a** as a beige solid (66.0 mg, 150 μmol, 43% o2s).

**^1^H NMR** (400 MHz, D_2_O) δ 5.81 (tp, *J* = 6.3, 2.1 Hz, 1H, H‑11), 5.70 (td, *J* = 6.8, 1.4 Hz, 1Hm H‑2), 5.39 (tq, *J* = 7.2, 1.3 Hz, 1H, H‑7), 4.55 (t, *J* = 6.6 Hz, 2H, H‑1), 4.03 (d, *J* = 7.2 Hz, 2H, H‑6), 3.96 (s, 2H, H‑4), 2.43 – 2.31 (m, 2H, H‑10), 2.23 (t, *J* = 7.3 Hz, 2H, H‑9), 1.72 (s, 3H, H‑2’), 1.70 (d, *J* = 1.3 Hz, 3H, H‑1’), 1.09 – 0.99 (m, 4H, H‑13 and H‑14).

**^13^C NMR** (101 MHz, D_2_O) δ 143.3 (C‑8), 136.6 (C‑3), 124.4 (d, *J* = 8.4 Hz, C‑2), 122.4 (C‑12), 119.2 (C‑7), 117.8 (C‑11), 74.7 (C‑4), 65.6 (C‑6), 62.1 (d, *J* = 5.0 Hz, C‑1), 38.3 (C‑9), 29.2 (C‑9), 15.5 (C‑1’), 13.5 (C‑2’), 1.6 (C‑13 or C‑14), 1.2 (C‑14 or C‑13).

**^31^P NMR** (162 MHz, D_2_O) δ −6.8 (d, *J* = 22.2 Hz), −10.2 (d, *J* = 22.2 Hz).

**ESI-MS** (ESI−) m/z Calculated for C_15_H_25_O_8_P_2_ [M−H]^−^ 395.1025; Found 395.1009.

#### (2*E*,6*E*)-10-Cyclobutylidene-3,7-dimethyldeca-2,6-dien-1-yl acetate (SI‑8)

|  |
| --- |

To a suspension of 4-bromobutyl(triphenyl)phosphonium bromide (**15**, 2.44 g, 5.09 mmol, 2.50 eq.) in dry THF (16 mL) was added *t*-BuOK (1.15 g, 10.2 mmol, 5.00 eq.) at 0 °C in two portions. The red solution was stirred for another 30 min before aldehyde **7** (500 mg, 2.10 mmol, 1.00 eq.) was added dropwise over a period of 30 min. The reaction mixture was stirred for another hour at 0 °C. Then, the reaction was terminated by the addition of water. The aqueous phase was extracted with Et_2_O (3 x) and the combined organic layers were washed with a sat. aqueous NH_4_Cl solution and brine, dried over MgSO_4_, filtered and the solvent was removed under reduced pressure. The title compound was purified by column chromatography (SiO_2_; PE/EA 50:1 to 25:1) to yield a light yellow oil (362 mg, 1.31 mmol, 63%).

**TLC**: *R*_F_ = 0.56 (SiO_2_; PE/EA 10:1).

**^1^H NMR** (400 MHz, CDCl_3_) δ 5.34 (tq, *J* = 7.1, 1.3 Hz, 1H, H‑2), 5.09 (ddt, *J* = 6.8, 5.6, 1.3 Hz, 1H, H‑6), 5.02 (dtq, *J* = 6.8, 4.5, 2.3 Hz, 1H, H‑10), 4.59 (d, *J* = 7.0 Hz, 2H, H‑1), 2.62 – 2.58 (m, 4H, H‑12 and H‑14), 2.17 – 2.07 (m, 4H, H‑4 and H‑5), 2.05 (s, 3H, OC(O)CH_3_), 2.05 – 1.96 (m, 4H, H‑8 and H‑9), 1.95 – 1.86 (m, 2H, C‑13), 1.70 (s, 3H, H‑1’), 1.59 (s, 3H, H‑2’).

**^13^C NMR** (101 MHz, CDCl_3_) δ 171.3 (O*C*(O)CH_3_), 142.4 (C-3), 140.0 (C-11), 135.6 (C-7), 123.8 (C‑6), 120.2 (C‑10), 118.4 (C‑2), 61.6 (C‑1), 39.8 (C‑4 or C‑5), 39.7 (C‑8 or C‑9), 31.0 (C‑12 or C‑14), 29.4 (C‑14 or C‑12), 26.7 (C‑9 or C‑8), 26.3 (C‑5 or C‑4), 21.2 (OC(O)*C*H_3_), 17.2 (C‑13), 16.6 (C‑1’), 16.1 (C‑2’).

**ESI-MS** (ESI+) m/z Calculated for C_18_H_28_O_2_Na [M+Na]^+^ 299.1987; Found 299.1992.

#### (2*E*,6*E*)-10-Cyclobutylidene-3,7-dimethyldeca-2,6-dien-1-ol (16)

|  |
| --- |

Acetate **SI‑8** (333 mg, 1.20 mmol, 1.00 eq.) was dissolved in MeOH (16 mL) and K_2_CO_3_ (166 mg, 1.20 mmol, 1.00 eq.) was added at rt. The mixture was stirred at the same temperature for 1 h and the reaction was stopped by the addition of brine. The solution was extracted with CH_2_Cl_2_ (3 x) and the combined organic layers were washed with brine, dried over MgSO_4_, filtered and concentrated under reduced pressure. After flash column chromatography (SiO_2_; PE/EA 10:1 to 4:1) alcohol **16** was collected as a colorless oil (247 mg, 1.05 mmol, 88%).

**TLC**: *R*_F_ = 0.31 (SiO_2_; PE/EA 6:1).

**^1^H NMR** (400 MHz, CDCl_3_) δ 5.42 (ddq, *J* = 6.9, 5.5, 1.3 Hz, 1H, H‑2), 5.14 – 5.06 (m, 1H, H‑6), 5.01 (tq, *J* = 4.5, 2.2 Hz, 1H, H‑10), 4.15 (dd, *J* = 6.9, 0.7 Hz, 2H, H‑1), 2.62 (dddd, *J* = 9.2, 7.7, 2.5, 1.3 Hz, 4H, H‑12 and H‑14), 2.17 – 2.09 (m, 2H, H‑5), 2.07 – 2.02 (m, 2H, H‑4), 2.00 – 1.95 (m, 4H, H‑8 and H‑9), 1.94 – 1.86 (m, 2H, C‑13), 1.68 (d, *J* = 0.9 Hz, 3H, H‑1’), 1.59 (d, *J* = 1.2 Hz, 3H, H‑2’), 1.36 (br. s, 1H, OH).

**^13^C NMR** (101 MHz, CDCl_3_) δ 140.02 (C‑3 or C‑11), 139.98 (C‑11 or C‑3), 135.5 (C‑7), 123.9 (C‑6), 123.5 (C‑2), 120.2 (C‑10), 59.6 (C‑1), 39.8 (C‑8 or C‑9), 39.7 (C‑4), 31.0 (C‑12 or C‑14), 29.4 (C‑14 or C‑12), 26.7 (C‑9 or C‑8), 26.4 (C‑5), 17.2 (C‑13), 16.4 (C‑1’), 16.1 (C‑2’).

**ESI-MS** (ESI+) m/z Calculated for C_16_H_25_ [M−OH]^+^ 217.1956; Found 217.1950.

#### Triammonium (2*E*,6*E*)-10-cyclobutylidene-3,7-dimethyldeca-2,6-dien-1-yl diphosphate (4b)

|  |
| --- |

**Step 1: Bromination**

To a solution of **16** (225 mg, 960 μmol, 1.00 eq.) in dry Et_2_O (11 mL) was added PBr_3_ (29.9 μL, 317 μmol, 0.33 eq.) at 0 °C. The reaction mixture was allowed to reach rt and stirring was continued for another 30 min. Upon full consumption (as judged by TLC), the reaction was terminated by the addition of Et_2_O and brine. The layers were separated, and the aqueous phase was extracted with Et_2_O (3 x). The combined organic layers were washed with water and brine, dried over MgSO_4_, filtered and concentrated *in vacuo* to yield the crude bromide which was used in the next step without further purification.

**Step 2: Pyrophosphorylation**

To (*n*-Bu_4_N)_3_P_2_O_7_ (1.23 g, 1.36 mmol, 1.50 eq.) dissolved in dry CH_3_CN (12 mL) was added dropwise the crude bromide from above dissolved in dry CH_3_CN (2 mL) in the presence of 3 Å molecular sieves at 0 °C. The mixture was allowed to reach rt and was stirred overnight. Subsequently, the solvent was removed under reduced pressure and the remaining solid was dissolved in IEB (3 mL). The solution was passed through an Amberchrom^TM^ 50WX8 Ion Exchange Column (NH_4_^+^ form). The aqueous, fractions that contained the product were combined and concentrated *in vacuo* and the resulting crude solids were dissolved in an aqueous NH_4_HCO_3_ solution (1 M, 2 mL). A mixture of CH_3_CN/IPA (1:1, 8 mL) was added and the suspension was subjected to centrifugation (4500 rpm, 4 °C, 5 min). The supernatant was separated and subjected to the same procedure. This protocol was repeated three times in total. The resulting supernatant was concentrated and lyophilized overnight to yield pyrophosphate **4b** as a pale yellow solid (269 mg, 604 μmol, 63% o2s).

**^1^H NMR** (400 MHz, D_2_O) δ 5.48 (s, 1H, H‑2), 5.22 (t, *J* = 6.6 Hz, 1H, H‑6), 5.12 (tt, *J* = 4.7, 2.4 Hz, 1H, H‑10), 4.50 (s, 2H, H‑1), 2.67 – 2.61 (m, 4H, H‑8 and H‑9), 2.24 – 2.15 (m, 2H, H‑5), 2.14 – 2.09 (m, 2H, H‑4), 2.08 – 1.99 (m, 4H, H‑12 and H‑14), 1.92 (p, *J* = 8.0 Hz, 2H, H‑13), 1.74 (s, 3H, H‑1’), 1.63 (s, 3H, H‑2’).

**^13^C NMR** (126 MHz, D_2_O) δ 143.1 (C‑3), 141.9 (C‑11), 136.6 (C‑7), 124.2 (C‑6), 120.2 (C‑10), 119.5 (d, *J* = 8.7 Hz C‑2), 62.9 (d, *J* = 5.2 Hz, C‑1), 38.8 (C‑4), 38.6 (C‑8), 30.5 (C‑12 or C‑14), 28.8 (C‑14 or C‑12), 25.6 (C‑9), 25.5 (C‑8), 16.5 (C‑13), 15.6 (C‑1’), 15.2 (C‑2’).

**^31^P NMR** (162 MHz, D_2_O) δ −6.3 (d, *J* = 22.3 Hz), −10.1 (d, *J* = 22.3 Hz).

**ESI-MS** (ESI−) m/z Calculated for C_16_H_26_O_7_P_2_ [M−H]^−^ 393.1232; Found 393.1237.

#### (*E*)-6-Cyclobutylidene-3-methylhex-2-en-1-ol (17)

**Step 1: Wittig olefination**

To a suspension of (4-bromobutyl)triphenylphosphonium bromide (**15**, 8.21 g, 13.2 mmol, 1.50 eq.) in THF (66 mL) was added *t*-BuOK (2.97 g, 26.4 mmol, 3.00 eq.) in two portions at 0 °C in a 30 min interval. To the resulting deep-orange suspension was added **7** (1.50 g, 8.81 mmol, 1.00 eq.) via a syringe pump (0.05 mL/min, neat). The suspension was stirred for 1 h at the same temperature and terminated by the addition of water and extracted with Et_2_O (3 x). The combined organic layers were washed with a sat. aqueous NH_4_Cl solution and brine, dried over MgSO_4_, filtered and concentrated *in vacuo*. The crude product was purified by column chromatography (SiO_2_; PE/EA 20:1 to 15:1) to give the corresponding cyclobutylidene acetate as a light-yellow oil (783 mg, 3.76 mmol).

**TLC**: *R*_F_ = 0.63 (SiO_2_; PE/EA 4:1).

**Step 2: Acetate deprotection**

To a solution of the cyclobutylidene acetate (783 mg, 3.76 mmol, 1.00 eq.) collected from above in MeOH (13 mL) was added K_2_CO_3_ (520 mg, 3.76 mmol, 1.00 eq.). The reaction was stirred at rt for 1 h, before it was terminated by the addition of brine. The mixture was extracted with CH_2_Cl_2_ (3 x) and the combined organic phases were washed with brine, dried over MgSO_4_ filtered and concentrated *in vacuo*. The residue was purified by column chromatography (SiO_2_; PE/EA 10:1 to 5:1) and the title compound was isolated as a colorless oil (537 mg, 3.23 mmol, 37% o2s).

**TLC**: *R*_F_ = 0.25 (SiO_2_; PE/EA 4:1).

**^1^H NMR** (400 MHz, CDCl_3_) δ 5.41 (td, *J* = 6.9, 1.3 Hz, 1H, H‑2), 5.17 – 4.95 (m, 1H, H‑6), 4.15 (d, *J* = 6.9 Hz, 2H, H‑1), 2.74 – 2.56 (m, 4H, H‑4 and H‑5), 2.07 – 1.98 (m, 4H, H‑8 and H‑10), 1.92 (p, *J* = 7.9 Hz, 2H, H‑9), 1.67 (s, 3H, H‑1’).

**^13^C NMR** (101 MHz, CDCl_3_) δ 140.4 (C‑7), 139.9 (C‑3), 123.5 (C‑2), 119.7 (C‑6), 59.6 (C‑1), 39.6 (C‑8), 31.0 (C‑4), 29.4 (C‑5), 26.4 (C‑10), 17.2 (C‑9), 16.4 (C‑1’).

**ESI-MS** (ESI+) m/z Calculated for C_17_H_11_^+^ [M−OH]^+^ 149.1330; Found 149.1325.

#### *tert*-Butyl(((*E*)-4-(((*E*)-6-cyclobutylidene-3-methylhex-2-en-1-yl)oxy)-3-methylbut-2-en-1-yl)oxy)diphenylsilane (SI‑9)

|  |
| --- |

To a suspension of NaH (90%, 64.2 mg, 2.41 mmol, 2.00 eq.) in dry THF (2.0 mL) was added alcohol **17** (200 mg, 1.20 mmol, 1.00 eq.) in dry THF (1.5 mL) dropwise and stirred under refluxing conditions for 2 h. TBAI (13.3 mg, 36.1 μmol, 0.03 eq.) and bromide **13** (562 mg, 1.44 mmol, 1.20 eq.) in dry THF (500 μL) were added to the suspension and the reaction mixture was stirred under refluxing conditions overnight. The reaction was stopped by the addition of an aqueous HCl solution (1 M) and the aqueous phase was extracted with Et_2_O (3 x). The combined organic phases were washed with brine, dried over MgSO_4_, filtered and the solvent was removed under reduced pressure. The crude product was purified by means of silica column chromatography (SiO_2_; PE/CH_2_Cl_2_ 100:1 to 10:1 to PE/EA 10:1) to yield the title compound **SI‑9** as a yellow oil (398 mg, 813 μmol, 68%).

**TLC**: *R*_F_ = 0.68 (SiO_2_; PE/EA 10:1).

**^1^H NMR** (400 MHz, CDCl_3_) δ 7.72 – 7.65 (m, 4H, ArH), 7.46 – 7.33 (m, 6H, ArH), 5.74 – 5.57 (m, 1H, H‑2), 5.34 (ddd, *J* = 8.4, 6.3, 1.5 Hz, 1H, H‑7), 5.04 (ddt, *J* = 6.8, 4.5, 2.3 Hz, 1H, H‑11), 4.27 (d, *J* = 6.1 Hz, 2H, H‑1), 3.91 (d, *J* = 6.8 Hz, 2H, H‑6), 3.83 (s, 2H, H‑4), 2.63 (ddd, *J* = 8.8, 5.2, 2.0 Hz, 4H, H‑13 and H‑15), 2.08 – 1.98 (m, 4H, H‑9 and H‑10), 1.92 (p, *J* = 7.9 Hz, 2H, H‑14), 1.65 (d, *J* = 1.3 Hz, 3H, H‑2’), 1.49 (d, *J* = 1.4 Hz, 3H, H‑1’), 1.04 (s, 9H, SiC_q_(C*H*_3_)_3_).

**^13^C NMR** (101 MHz, CDCl_3_) δ 140.3 (C‑12), 140.2 (C‑8), 135.7 (ArC), 134.0 (ArC_q_), 133.9 (C-3), 129.7 (ArC), 127.8 (ArC), 127.3 (C‑2), 121.1 (C‑7), 119.9 (C‑11), 75.5 (C‑4), 66.1 (C‑6), 60.9 (C‑1), 39.7 (C‑9 or C‑10), 31.0 (C‑13 or C‑15), 29.4 (C‑15 or C‑13), 27.0 (SiC_q_(*CH*_3_)_3_), 26.4 (C‑10 or C‑9), 19.3 (Si*C*_q_(CH_3_)_3_), 17.2 (C‑14), 16.6 (C‑2’), 14.2 (C‑1’).

**ESI-MS** (ESI+) m/z Calculated for C_32_H_44_O_2_SiNa^+^ [M+Na]^+^ 511.3008; Found 511.2998.

#### (*E*)-3-(((*E*)-6-Cyclobutylidene-3-methylhex-2-en-1-yl)oxy)but-2-en-1-ol (18)

|  |
| --- |

TBAF (1 M in THF, 945 μL, 945 μmol, 3.00 eq.) was added dropwise to a solution of silyl ether **SI‑9** (154 mg, 315 μmol, 1.00 eq.) in dry THF (3.0 mL) at 0 °C. The reaction mixture was stirred overnight at rt and stopped by the addition of a sat. aqueous NH_4_Cl solution. The aqueous layer was extracted with EA (3 x), the combined organic phases were washed with brine, dried over MgSO_4_, filtered and concentrated under reduced pressure. After flash column chromatography (SiO_2_; PE/EA 5:1 to 2:1) alcohol **18** was isolated as a colorless oil (45.1 mg, 180 μmol, 57%).

**TLC**: *R*_F_ = 0.30 (SiO_2_; PE/EA 4:1).

**^1^H NMR** (400 MHz, CDCl_3_) δ 5.67 (tq, *J* = 6.8, 1.3 Hz, 1H, H‑2), 5.34 (tq, *J* = 6.8, 1.3 Hz, 1H), 5.02 (ddt, *J* = 6.9, 4.6, 2.4 Hz, 1H, H‑7), 4.21 (dq, *J* = 6.7, 0.8 Hz, 2H, H‑1), 3.96 (dq, *J* = 6.8, 0.8 Hz, 2H, H‑6), 3.86 (s, 2H, H‑4), 2.67 – 2.57 (m, 4H, H‑13 and H‑15), 2.04 – 1.97 (m, 4H, H‑9 and H‑10), 1.96 – 1.85 (m, 2H, H‑14), 1.70 (s, 3H, H‑1’), 1.65 (s, 3H, H‑2’), 1.40 (br. s, 1H, OH).

**^13^C NMR** (101 MHz, CDCl_3_) δ 140.4 (C‑8), 140.3 (C‑12), 136.3 (C‑3), 126.0 (C‑2), 120.9 (C‑7), 119.8 (C‑11), 75.3 (C‑4), 66.6 (C‑7), 59.3 (C‑1), 39.7 (C‑9 or C‑10), 31.0 (C‑13 or C‑15), 29.4 (C‑15 or C‑13), 26.4 (C‑10 or C‑9), 17.2 (C‑14), 16.6 (C‑2’), 14.2 (C‑1’).

**ESI-MS** (ESI+) m/z Calculated for C_16_H_26_O_2_Na^+^ [M+Na]^+^ 273.1831; Found 273.1818.

#### Triammonium (*E*)-4-(((*E*)-6-cyclobutylidene-3-methylhex-2-en-1-yl)oxy)-3-methylbut-2-en-1-yl diphosphate (5b)

**Step 1: Bromination**

To a solution of **18** (50 mg, 212 μmol, 1.00 eq.) in dry Et_2_O (2.5 mL) was added PBr_3_ (6.98 μL, 74.0 μmol, 0.35 eq.) at 0 °C. the mixture was stirred for 30 min at this temperature. Upon completion (judged by TLC) the reaction mixture was diluted with Et_2_O and brine. The layers were separated and the aqueous layer was extracted with Et_2_O (3 x). The combined organic phases were washed with water, brine, dried over MgSO_4_, filtered and concentrated *in vacuo*. The crude bromide was used in the next step without further purification.

**Step 2: Pyrophosphorylation**

To (*n*-Bu_4_N)_3_P_2_O_7_ (272 mg, 302 μmol, 1.50 eq.) dissolved in dry CH_3_CN (4.0 mL) was added the bromide from above in the presence of 3 Å molecular sieves at 0 °C. The mixture was allowed to reach rt and was stirred overnight. Subsequently, the solvent was removed under reduced pressure and the remaining solid was dissolved in IEB (3 mL). The solution was passed through an Amberchrom^TM^ 50WX8 Ion Exchange Column (NH_4_^+^ form). The aqueous fractions that contained product were concentrated *in vacuo* and the resulting crude material was dissolved in a NH_4_HCO_3_ solution (1 M, 2 mL). A mixture of CH_3_CN/IPA (1:1, 8 mL) was added and the suspension was subjected to centrifugation (4500 rpm, 4 °C, 5 min). The supernatant was separated and subjected to the same procedure. This protocol was repeated three times in total. The resulting supernatant was concentrated and lyophilized overnight to deliver pyrophosphate **5b** as a light beige solid (78.1 mg, 170 μmol, 85% o2s).

**^1^H NMR** (400 MHz, D_2_O) δ 5.73 – 5.62 (m, 1H, H‑2), 5.35 (t, *J* = 7.3 Hz, 1H, H‑7), 5.15 – 5.05 (m, 1H, H‑11), 4.64 – 4.49 (m, 2H, H‑1), 4.03 (d, *J* = 7.1 Hz, 2H, H‑6), 3.94 (s, 2H, H‑4), 2.72 – 2.46 (m, 4H, H‑13 and H‑15), 2.13 – 2.01 (m, 4H, H‑9 and H‑10), 1.91 (p, *J* = 7.9 Hz, 2H, H‑14), 1.72 (s, 3H, H‑1’), 1.68 (s, 3H, H‑2’).

**^13^C NMR** (151 MHz, D_2_O) δ 142.8 (C‑8), 141.4 (C‑12), 137.1 (C‑3), 123.7 (C‑2), 120.0 (C‑11), 119.6 (C‑7), 74.6 (C‑4), 65.9 (C‑6), 62.4 (C‑1), 38.9 (C‑9), 30.6 (C‑13 or C‑15), 28.9 (C‑15 or C‑13), 25.6 (C‑10), 16.7 (C‑14), 15.6 (C‑2’), 13.6 (C‑1’).

**^31^P NMR** (162 MHz, D_2_O) δ −6.4, −10.1.

**ESI-MS** (ESI−) m/z Calculated for C_16_H_27_O_8_P_2_^−^ [M−H]^−^ 409.1181; Found 409.1183.

#### *tert*-Butyldiphenyl(((2*E*,6*E*)-3,7,11-trimethyldodeca-2,6,10-trien-1-yl)oxy)silane (SI‑10)

|  |
| --- |

To farnesol (**6**, 16.9 mL, 67.5 mmol, 1.00 eq.) dissolved in dry CH_2_Cl_2_ (112 mL) was added imidazole (10.1 g, 148 mmol, 2.20 eq.) and TBDPSCl (18.9 mL, 74.2 mmol, 1.10 eq.) at 0 °C. The mixture was allowed to warm to rt and stirred overnight. After completion (as judged by TLC) the reaction was stopped by the addition of water and the separated aqueous layer was extracted with CH_2_Cl_2_ (3 x). The combined organic phases were dried over MgSO_4_, filtered, the solvent was removed *in vacuo* and silyl ether **SI‑10** was isolated as a colorless oil (31.1 g, 67.5 mmol, quant.).

**TLC**: *R*_F_ = 0.85 (SiO_2_; PE/EA 16:1).

**^1^H NMR** (400 MHz, CDCl_3_) δ 7.73 – 7.65 (m, 4H, ArH), 7.46 – 7.33 (m, 6H, ArH), 5.39 (ddq, *J* = 6.3, 5.1, 1.3 Hz, 1H, H‑2), 5.16 – 5.10 (m, 2H, H‑6 and H‑10), 4.22 (dq, *J* = 6.3, 0.9 Hz, 2H, H‑1), 2.11 – 2.04 (m, 4H, H‑5 and H‑8), 2.00 – 1.97 (m, 4H, H‑4 and H‑9), 1.68 (d, *J* = 1.3 Hz, 3H, H‑2’), 1.60 (s, 6H, H‑12 and H‑13) 1.44 (d, *J* = 1.2 Hz, 3H, H‑1’), 1.04 (s, 9H, SiC_q_(C*H*_3_)_3_).

**^13^C NMR** (101 MHz, CDCl_3_) δ 137.2 (C‑3), 135.8 (ArC), 135.3 (ArC), 134.9 (C‑11), 131.4 (C‑7), 129.6 (ArC), 127.7 (ArC), 124.5 (C‑10), 124.2 (C‑6), 124.1 (C‑2), 61.3 (C‑1), 39.9 (C‑4 or C‑9), 39.7 (C‑5 or C‑8), 27.0 (C‑8 or C‑5), 26.9 (SiC_q_(*C*H_3_)_3_), 26.5 (C‑9 or C‑4), 25.9 (C‑2’), 19.3 (Si*C*_q_(CH_3_)_3_), 17.8 (C‑12 or C‑13), 16.5 (C‑1’), 16.2 (C‑13 or C‑12).

The analytic data are in accordance with those reported in the literature.^S16^

#### (4*E*,8*E*)-10-((*tert*-Butyldiphenylsilyl)oxy)-4,8-dimethyldeca-4,8-dienal (19)

|  |
| --- |

**Step 1: Epoxidation**

To silyl ether **SI‑10** (27.0 g, 58.6 mmol, 1.00 eq.) in THF/H_2_O (3:1, 780 mL) was added NBS (14.6 g, 82.1 mmol, 1.40 eq.) at 0 °C and the mixture was allowed to warm to rt. After 1 h the mixture was diluted by the addition of water and extracted with PE (4 x). The combined organic layers were washed with brine and dried over MgSO_4_, filtered and concentrated under reduced pressure. The residue was dissolved in MeOH (300 mL) and K_2_CO_3_ (16.2 g, 117 mmol, 2.00 eq.) was added at rt and stirring was continued for 1 h. The solvent was evaporated under reduced pressure and the residue was dissolved in water and EA. The phases were separated and the aqueous layer was extracted with EA (3 x). The combined organic layers, were washed with brine, dried over Na_2_SO_4_, filtered and the organic solvent was removed *in vacuo*. The crude material obtained was used in the next step without further purification

**Step 2: Oxidative cleavage to aldehyde**

The crude epoxide from above was dissolved in THF/H_2_O (3:1, 665 mL) and cooled to 0 °C. To the solution was added H_5_IO_6_ (14.7 g, 64.5 mmol, 1.10 eq.) and NaIO_4_ (8.28 g, 38.7 mmol, 0.66 eq.) and the mixture was stirred for 1.5 h at this temperature. The reaction was terminated by the addition of a sat. aqueous NaHCO_3_ solution. The aqueous phase was extracted with EA (3 x) and the combined organic layers were washed with brine, dried over Na_2_SO_4_, filtered and concentrated under reduced pressure. The title compound **19** was isolated after flash column chromatography (SiO_2_; PE/EA 10:1) as a colorless oil (7.69 g, 17.7 mmol, 30% o2s).

**TLC**: *R*_F_ = 0.50 (SiO_2_; PE/EA 95:5).

**^1^H NMR** (400 MHz, CDCl_3_) δ 9.63 (t, *J* = 2.0 Hz, 1H, H‑10), 7.63 – 7.56 (m, 4H, ArH), 7.36 – 7.23 (m, 6H, ArH), 5.28 (t, *J* = 6.4 Hz, 1H, H‑2), 5.08 – 4.96 (m, 1H, H‑6), 4.13 (d, *J* = 6.3 Hz, 2H, H‑1), 2.40 (td, *J* = 7.5, 1.9 Hz, 2H, H‑9), 2.20 (dd, *J* = 13.7, 6.2 Hz, 2H, H‑8), 1.97 (t, *J* = 7.1 Hz, 2H, H‑5), 1.88 (dd, *J* = 8.9, 6.1 Hz, 2H, H‑4), 1.52 (s, 3H, H‑2’), 1.34 (s, 3H, H‑1’), 0.95 (s, 6H, SiC_q_(C*H*_3_)_3_).

**^13^C NMR** (101 MHz, CDCl_3_) δ 202.7 (C‑10), 136.8 (C‑7), 135.7 (ArC), 134.2 (ArC_q_), 133.3 (C‑3), 129.6 (ArC), 127.7 (ArC), 125.2 (C‑6), 124.4 (C‑2), 61.2 (C‑1), 42.3 (C‑9), 39.4 (C‑4), 31.9 (C‑8), 27.0 (SiC_q_(*C*H_3_)_3_), 26.3 (C‑5), 19.3 (Si*C*_q_(CH_3_)_3_), 16.4 (C‑1’), 16.3 (C‑2’).

The analytic data are in accordance with those reported in the literature.^S16^

#### (4*E*,8*E*)-10-((*tert*-Butyldiphenylsilyl)oxy)-4,8-dimethyldeca-4,8-dien-1-ol (SI‑11)

|  |
| --- |

To aldehyde **19** (4.50 g, 10.4 mmol, 1.00 eq.) dissolved in MeOH (122 mL) was added NaBH_4_ (783 mg, 20.7 mmol, 2.00 eq.) portionwise at 0 °C. The suspension was allowed to reach rt and stirring was continued for 1 d. The reaction was stopped by slow addition of water and the mixture was extracted with EA (5 x) and the combined organic layers were washed with brine, dried over Na_2_SO_4_, filtered and concentrated *in vacuo*. The crude residue was purified *via* silica column chromatography (SiO_2_; PE/EA 4:1) to give alcohol **SI‑11** as a colorless oil (2.61 g, 5.98 mmol, 58%).

**TLC**: *R*_F_ = 0.26 (SiO_2_; PE/EA 1:1).

**^1^H NMR** (400 MHz, CDCl_3_) δ 7.73 – 7.65 (m, 4H, ArH), 7.44 – 7.33 (m, 6H, ArH), 5.42 – 5.31 (m, 1H, H‑2), 5.20 – 5.09 (m, 1H, H‑6), 4.22 (d, *J* = 6.2 Hz, 2H, H‑1), 3.61 (t, *J* = 6.5 Hz, 2H, H‑10), 2.13 – 2.02 (m, 4H, H‑5 and H‑8), 2.02 – 1.94 (m, 2H, H‑4), 1.71 – 1.62 (m, 2H, H‑9), 1.62 (s, 3H, H‑2’), 1.44 (s, 3H, H‑1’), 1.04 (br. s, 1H, OH).

**^13^C NMR** (101 MHz, CDCl_3_) δ 137.0 (C‑3), 135.8 (ArC), 134.9 (C‑7), 134.2 (ArC_q_), 129.6 (ArC), 127.7 (ArC), 124.7 (C‑6), 124.3 (C‑2), 62.9 (C‑10), 61.3 (C‑1), 39.6 (C‑4), 36.1 (C‑8), 30.8 (C‑9), 27.0 (SiC_q_(*C*H_3_)_3_), 26.3 (C‑5), 19.3 (Si*C*_q_(CH_3_)_3_), 16.4 (C‑1’), 16.0 (C‑2’).

The analytic data are in accordance with those reported in the literature.^S17^

#### *tert*-Butyl(((2*E*,6*E*)-10-iodo-3,7-dimethyldeca-2,6-dien-1-yl)oxy)diphenylsilane (SI‑12)

|  |
| --- |

PPh_3_ (2.03 g 7.74 mmol, 1.30 eq.) and imidazole (689 mg, 10.1 mmol, 1.70 eq.) were added to a solution of alcohol **SI‑11** (2.60 g, 5.95 mmol, 1.00 eq.) in CH_2_Cl_2_ (23 mL) at 0 °C. To this mixture was added iodine (1.96 g, 7.74 mmol, 1.30 eq.) and stirring was continued at this temperature for 30 min. It was warmed up to rt and stirring was continued for further 1.5 h. The reaction was stopped by the addition of an aqueous Na_2_SO_3_ solution (10%) and the mixture was stirred for 30 min. The phases were separated and the aqueous layer was extracted with CH_2_Cl_2_ (3 x), and the combined organic phases were washed with brine, dried over MgSO_4_, filtered and the solution concentrated under reduced pressure. After column chromatography (SiO_2_; PE/EA 20:1) the title compound **SI‑12** was collected as a colorless oil (2.58 g, 4.72 mmol, 79%).

**TLC**: *R*_F_ = 0.79 (SiO_2_; PE/EA 100:1).

**^1^H NMR** (400 MHz, CDCl_3_) δ 7.73 – 7.66 (m, 4H, ArH), 7.39 (tt, *J* = 8.0, 5.7 Hz, 6H, ArH), 5.38 (ddd, *J* = 6.3, 5.0, 2.5 Hz, 1H, H‑2), 5.17 (td, *J* = 6.8, 6.3, 3.2 Hz, 1H, H‑6), 4.22 (d, *J* = 6.3 Hz, 2H, H‑2), 3.13 (t, *J* = 6.9 Hz, 2H, H‑10), 2.12 – 2.03 (m, 4H, H‑5, H‑8), 2.02 – 1.96 (m, 2H, H‑4), 1.95 – 1.84 (m, 2H, H‑9), 1.59 (d, *J* = 1.4 Hz, 3H, H‑2’), 1.44 (d, *J* = 1.3 Hz, 3H, H‑1’), 1.05 (s, 9H, SiC_q_(C*H*_3_)_3_).

**^13^C NMR** (101 MHz, CDCl_3_) δ 137.0 (C‑2), 135.8 (ArC), 134.2 (ArC_q_), 133.2 (C‑7), 129.6 (ArC), 127.7 (ArC), 125.7 (C‑6), 124.3 (C‑2), 61.3 (C‑1), 40.2 (C‑5 or C‑8), 39.5 (C‑4), 31.7 (C‑9), 27.0 (SiC_q_(*C*H_3_)_3_), 26.4 (C‑8 or C‑5), 19.3 (Si*C*_q_(CH_3_)_3_), 16.5 (C‑1’), 16.0 (C‑2’), 6.8 (C‑10).

The analytic data are in accordance with those reported in the literature.^S17^

#### ((4*E*,8*E*)-10-((*tert*-Butyldiphenylsilyl)oxy)-4,8-dimethyldeca-4,8-dien-1-yl)iodotriphenyl-l5-phosphane (20)

|  |
| --- |

To iodide **SI‑12** (850 mg, 1.56 mmol, 1.00 eq.) in dry toluene (35 mL) was added PPh_3_ (489 mg, 1.87 mmol, 1.20 eq.) and the resulting yellow solution was stirred under refluxing conditions for 3 d. Next, the solvent was removed under reduced pressure and the residue was purified by flash column chromatography (SiO_2_; CH_2_Cl_2_/MeOH 20:1 to 5:1) which provided **20** as a pale yellow foam (1.11 g, 1.37 mmol, 88%).

**TLC**: *R*_F_ = 0.30 (SiO_2_; CH_2_Cl_2_/MeOH 20:1).

**^1^H NMR** (400 MHz, CDCl_3_) δ 7.81 – 7.68 (m, 9H, ArH), 7.71 – 7.56 (m, 10H, ArH), 7.45 – 7.25 (m, 6H, ArH), 5.29 (td, *J* = 6.3, 3.3 Hz, 1H, H‑2), 5.10 (t, *J* = 6.8 Hz, 1H, H‑6), 4.12 (d, *J* = 6.3 Hz, 2H, H‑1), 3.54 – 3.42 (m, 2H, H‑10), 2.25 (t, *J* = 7.2 Hz, 2H, H‑8), 2.04 – 1.93 (m, 2H, H‑5), 1.88 (dd, *J* = 9.3, 5.9 Hz, 2H, H‑4), 1.73 – 1.68 (m, 2H, H‑9), 1.41 (s, 3H, H‑2’), 1.36 (s, 3H, H‑1’), 0.98 (d, *J* = 1.0 Hz, 9H, SiC_q_(C*H*_3_)_3_).

**^13^C NMR** (101 MHz, CDCl_3_) δ 136.9 (C‑3), 135.4 (ArC), 135.1 (d, *J* = 3.0 Hz, ArC), 133.9 (ArC_q_), 133.5 (d, *J* = 10.0 Hz, ArC), 132.6 (s, C‑7), 130.5 (d, *J* = 12.5 Hz, ArC), 129.4 (ArC), 127.5 (ArC), 126.7 (C‑6), 123.9 (C‑2), 117.5 (d, *J* = 1.1 Hz, ArC_q_), 61.0 (C‑1), 39.4 (d, *J* = 15.6 Hz, C‑8), 39.2 (C‑4), 26.8 (SiC_q_(*C*H_3_)_3_), 26.3 (C‑5), 22.1 (d, *J* = 50.8 Hz, C‑10), 20.4 (d, *J* = 3.9 Hz), 19.1 (Si*C*_q_(CH_3_)_3_), 16.3 (C‑1’), 15.7 (C‑2’).

The analytic data are in accordance with those reported in the literature.^S18^

#### *tert*-Butyl(((2*E*,6*E*)-3,7-dimethyl-10-(oxetan-3-ylidene)deca-2,6-dien-1-yl)oxy)diphenylsilane (SI‑13)

|  |
| --- |

To a solution of **20** (2.00 g, 2.47 mmol, 1.00 eq.) in dry THF (12 mL) was added *n*-BuLi (1.6 M, 2.47 mL, 3.96 mmol, 1.60 eq.) at −78 °C and the resulting red mixture was stirred for 1 h at this temperature. Oxetan-3-one (**21**, 254 μL, 3.96 mmol, 1.60 eq.) in dry THF (1.8 mL) was added dropwise to the dark red solution and the reaction was stirred overnight at rt. The reaction was terminated by the addition of water and the suspension was extracted with EA (3 x). The combined organic layers were washed with brine, dried over MgSO_4_, filtered and the solvent was removed *in vacuo*. The title compound **SI‑13** was isolated after column chromatography (SiO_2_; PE/EA 20:1) as a colorless oil (560 mg, 1.18 mmol, 48%).

**TLC**: *R*_F_ = 0.26 (SiO_2_; PE/EA 1:1).

**^1^H NMR** (400 MHz, CDCl_3_) δ 7.72 – 7.66 (m, 4H, ArH), 7.46 – 7.33 (m, 6H, ArH), 5.38 (tt, *J* = 5.2, 2.8 Hz, 1H, H‑2), 5.24 – 5.13 (m, 4H, H‑12 and H‑14), 5.15 – 5.03 (m, 2H, H‑6 and H‑10), 4.22 (d, *J* = 6.3 Hz, 2H, H‑1), 2.13 – 1.90 (m, 8H, H‑4, H‑5, H‑8 and H‑9), 1.59 (d, *J* = 1.3 Hz, 3H, H‑2’), 1.44 (d, *J* = 1.4 Hz, 3H, H‑1’), 1.04 (s, 9H, SiC_q_(C*H*_3_)_3_).

**^13^C NMR** (101 MHz, CDCl_3_) δ 137.1 (C‑3), 135.8 (ArC), 134.4 (C‑11), 134.2 (C‑7), 133.8 (ArC_q_), 129.6 (ArC), 127.7 (ArC), 124.8 (C‑6), 124.3 (C‑2), 119.5 (C‑10), 79.7 (C‑12 or C‑14), 79.0 (C‑14 or C‑12), 61.3 (C‑1), 39.6 (C‑8), 39.0 (C‑4), 27.0 (SiC_q_(*C*H_3_)_3_), 26.9 (C‑5 or C‑9), 26.4 (C‑9 or C‑5), 19.3 (Si*C*_q_(CH_3_)_3_), 16.5 (C‑1’), 16.1 (C‑2’).

**ESI-MS** (ESI+) m/z Calculated for C_31_H_42_O_2_SiNa^+^ [M+Na]^+^ 497.2852; Found 497.2837.

#### (2*E*,6*E*)-3,7-Dimethyl-10-(oxetan-3-ylidene)deca-2,6-dien-1-ol (22)

|  |
| --- |

To silyl ether **SI‑13** (251 mg, 529 μmol, 1.00 eq.) in dry THF (8.0 mL) was added TBAF (1 M, 1.59 mL, 1.59 mmol, 3.00 eq.) at 0 °C and the reaction mixture was stirred at rt for 1 d. The reaction was terminated by the addition of a sat. aqueous NH_4_Cl solution and the layers were separated. The aqueous phase was extracted with EA (3 x) and the combined organic layers were washed with brine, dried over Na_2_SO_4_, filtered and concentrated under reduced pressure. The residue was purified by flash column chromatography (SiO_2_; PE/EA 6:1) to yield alcohol **22** as a colorless oil (115 mg, 486 μmol, 92%).

**TLC**: *R*_F_ = 0.57 (SiO_2_; PE/EA 1:1).

**^1^H NMR** (400 MHz, CDCl_3_) δ 5.38 (tq, *J* = 6.9, 1.3 Hz, 1H, H‑2), 5.21 – 5.12 (m, 4H, H‑12 and H‑14), 5.12 – 5.02 (m, 2H, H‑6 and H‑10), 4.11 (dd, *J* = 6.9, 1.1 Hz, 2H, H‑1), 2.16 – 2.05 (m, 2H, H‑5), 2.05 – 1.96 (m, 4H, H‑4 and H‑8), 1.96 – 1.86 (m, 2H, H‑9), 1.65 (d, *J* = 1.4 Hz, 3H, H‑1’), 1.56 (d, *J* = 1.4 Hz, 3H, H‑2’).

**^13^C NMR** (101 MHz, CDCl_3_) δ 139.7 (C‑3), 134.6 (C‑7), 133.9 (C‑11), 124.6 (C‑6), 123.6 (C‑2), 119.5 (C‑10), 79.7 (C‑12 or C‑14), 79.0 (C‑14 or C‑12), 59.6 (C‑1), 39.6 (C‑4), 39.0 (C‑8), 26.8 (C‑9), 26.4 (C‑5), 16.4 (C‑1’), 16.1 (C‑2’).

**ESI-MS** (ESI+) m/z Calculated for C_15_H_24_O_2_Na^+^ [M+Na]^+^ 259.1674; Found 259.1668.

#### Triammonium (2*E*,6*E*)-3,7-dimethyl-10-(oxetan-3-ylidene)deca-2,6-dien-1-yl diphosphate (4c)

|  |
| --- |

**Step 1: Preparation of the TEAP solution**

Solution A: Concentrated phosphoric acid (1.50 mL) dissolved in CH_3_CN (9.40 mL); Solution B: Freshly distilled Et_3_N (11.0 mL) dissolved in CH_3_CN (10.0 mL).

To freshly generate TEAP, Solution A (0.91 mL) and Solution B (1.50 mL) were mixed and stirred for 5 min at 37 °C.

**Step 2: Pyrophosphorylation**

To alcohol **22** (47.3 mg, 200 μmol, 1.00 eq.) in CCl_3_CN (500 μL) was added a freshly prepared TEAP solution in three portions (each 500 μL) in an interval of 5 min between each addition. After the final addition the orange solution was stirred for further 5 min and the reaction mixture was loaded on a flash column and eluted (SiO_2_; IPA/NH_3_/H_2_O 6:3:1). After a second flash column purification under identical conditions pyrophosphate **4c** was isolated as a pale yellow solid (21.4 mg, 47.8 μmol, 24%).

**TLC**: *R*_F_ = 0.38 (SiO_2_; IPA/NH_3_/H_2_O 6:3:1).

**^1^H NMR** (400 MHz, D_2_O) δ 5.57 – 5.42 (m, 1H, H‑2), 5.33 – 5.28 (m, 4H, H‑12 and H‑14), 5.26 – 5.17 (m, 2H, H‑6 and H‑10), 4.48 (t, *J* = 6.6 Hz, 2H, H‑1), 2.17 (t, *J* = 6.9 Hz, 2H, H‑5), 2.13 (d, *J* =  6.7 Hz, 2H, H‑4), 2.05 (d, *J* = 6.2 Hz, 2H, H‑8), 2.03 – 1.96 (m, 2H, H‑9), 1.74 (s, 3H, H‑1’), 1.62 (s, 2H, H‑2’).

**^13^C NMR** (151 MHz, D_2_O) δ 145.8 (C-3), 138.7 (C‑7), 134.6 (C‑11), 127.4 (C‑6), 123.2 (C‑10), 122.2 (C‑2), 83.0 (C‑12 or C‑14), 82.4 (C‑14 or C‑12), 65.7 (t, *J* = 2.7 Hz, C‑1), 41.5 (C‑4), 40.7 (C‑8), 28.6 (C‑9), 28.2 (C‑5), 18.3 (C‑1’), 17.8 (C‑2’).

**^31^P NMR** (162 MHz, D_2_O) δ −9.0, −10.7.

**ESI-MS** (ESI−) m/z Calculated for C_15_H_25_O_8_P_2_^−^ [M−H]^−^ 395.1025; Found 395.1031.

#### (*E*)-*tert*-Butyl((3,7-dimethylocta-2,6-dien-1-yl)oxy)diphenylsilane (SI‑14)

|  |
| --- |

To geraniol (**10**, 11.4 mL, 67.5 mmol, 1.00 eq.) dissolved in dry CH_2_Cl_2_ (108 mL) was added imidazole (9.71 g, 143 mmol, 2.20 eq.) and TBDPSCl (18.2 mL, 71.3 mmol, 1.10 eq.) at 0 °C. The mixture was allowed to warm to rt and stirring was continued overnight. Afterwards, the reaction was stopped by the addition of water. The aqueous layer was extracted with CH_2_Cl_2_ (3 x), the combined organic phases were dried over MgSO_4_, filtered, the solvent was removed *in vacuo* and silyl ether **SI‑14** was isolated as a colorless oil (25.5 g, 64.8 mmol, quant.).

**TLC**: *R*_F_ = 0.87 (SiO_2_; PE/EA 8:1).

**^1^H NMR** (400 MHz, CDCl_3_) δ 7.73 – 7.66 (m, 4H, ArH), 7.44 – 7.35 (m, 6H, ArH), 5.38 (tq, *J* = 6.3, 1.3 Hz, 1H, H‑2), 5.10 (dddd, *J* = 6.9, 5.5, 2.8, 1.4 Hz, 1H, H‑6), 4.23 (dd, *J* = 6.3, 0.9 Hz, 1H, H‑1), 2.11 – 2.04 (m, 2H, H‑5), 2.02 – 1.94 (m, 2H, H‑4), 1.69 (d, *J* = 1.3 Hz, 3H, H‑2’), 1.61 (s, 1H, H‑3’), 1.44 (s, 2H, H‑1’), 1.05 (s, 9H, SiC_q_(C*H*_3_)_3_).

**^13^C NMR** (101 MHz, CDCl_3_) δ 137.2 (C‑3), 135.8 (ArC), 134.3 (ArC_q_), 131.7 (C‑7), 129.6 (ArC), 127.7 (ArC), 124.3 (C‑6), 124.2 (C‑2), 61.3 (C‑1), 39.6 (C‑4), 27.0 (SiC_q_(*C*H_3_)_3_), 26.5 (C‑5), 25.8 (C‑2’), 19.3 (Si*C*_q_(CH_3_)_3_), 17.9 (C‑3’), 16.5 (C‑1’).

The analytic data are in accordance with those reported in the literature.^S14^

#### (*E*)-6-((*tert*-Butyldiphenylsilyl)oxy)-4-methylhex-4-enal (23)

|  |
| --- |

**Step 1: Epoxidation**

To silyl ether **SI‑14** (25.0 g, 63.7 mmol, 1.00 eq.) in CH_2_Cl_2_ (212 mL) was added *m*CPBA (77%, 15.7 g, 70.0 mmol, 1.10 eq.) portionwise at ‑10 °C. The suspension was stirred at the same temperature for 30 min and subsequently terminated by the addition of an aqueous NaOH solution (1 M). The phases were separated and the aqueous layer was extracted with CH_2_Cl_2_ (3 x), the organic layers were combined, washed with a sat. aqueous NaHCO_3_ solution and brine, then dried over MgSO_4_, filtered and after concentration under reduced pressure, the crude epoxide was collected, which was used in the next step without further purification.

**Step 2: Oxidative cleavage to aldehyde**

H_5_IO_6_ (14.5 g, 63.7 mmol, 1.00 eq.) was added portionwise to an ice-cold solution of crude epoxide in THF/H_2_O (159 mL, 3:2) and stirring was continued at the same temperature for 1 h. Subsequently, the mixture was diluted by the addition of brine and the solution was extracted with Et_2_O (3 x). The combined organic phases were dried over MgSO_4_, filtered and concentrated *in vacuo*. Aldehyde **23** was isolated after column chromatography (SiO_2_; PE/EA 10:1) as a colorless oil (17.0 g, 46.3 mmol, 73% o2s).

**TLC**: *R*_F_ = 0.40 (SiO_2_; PE/EA 8:1).

**^1^H NMR** (400 MHz, CDCl_3_) δ 9.75 (t, *J* = 1.8 Hz, 1H, H‑6), 7.71 – 7.66 (m, 4H, ArH), 7.42 – 7.37 (m, 6H, ArH), 5.43 – 5.34 (m, 1H, H‑2), 4.22 (dd, *J* = 6.3, 0.9 Hz, 2H, H‑1), 2.54 – 2.45 (m, 2H, H‑5), 2.30 (t, *J* = 7.5 Hz, 3H, H‑4), 1.45 (s, 3H, H‑1’), 1.05 (s, 9H, SiC_q_(C*H*_3_)_3_).

**^13^C NMR** (101 MHz, CDCl_3_) δ 202.4 (C‑6), 135.8 (ArC), 135.1 (C‑3), 134.1 (ArC_q_), 129.7 (ArC), 127.8 (ArC), 125.2 (C‑2), 61.1 (C‑1), 42.0 (C‑5), 31.7 (C‑4), 27.0 (SiC_q_(*C*H_3_)_3_), 19.3 (Si*C*_q_(CH_3_)_3_), 16.6 (C‑1’).

The analytic data are in accordance with those reported in the literature.^S19^

#### (*E*)-6-((*tert*-Butyldiphenylsilyl)oxy)-4-methylhex-4-en-1-ol (SI‑15)

|  |
| --- |

To a solution of **23** (5.00 g, 13.6 mmol, 1.00 eq.) in MeOH (136 mL) was added NaBH_4_ (1.03 g, 27.3 mmol, 2.00 eq.) at 0 °C. The mixture was stirred for 3 h at this temperature and upon full consumption (as judged by tlc) the solvent was evaporated under reduced pressure. The residue was dissolved in a sat. aqueous NaHCO_3_ solution and EA. The layers were separated and the aqueous phase was extracted with EA (3 x). The combined organic layers were washed with brine, dried over Na_2_SO_4_, filtered and concentrated *in vacuo*. The crude product was purified by flash column chromatography (SiO_2_; PE/EA 6:1) to give title compound **SI‑15** as a pale yellow oil (2.60 g, 7.05 mmol, 52%).

**TLC**: *R*_F_ = 0.13 (SiO_2_; PE/EA 8:1).

**^1^H NMR** (400 MHz, CDCl_3_) δ 7.75 – 7.65 (m, 4H, ArH), 7.43 – 7.35 (m, 6H, ArH), 5.41 (tdd, *J* = 5.0, 2.7, 1.4 Hz, 1H, H‑2), 4.22 (d, *J* = 6.3 Hz, 1H, H‑1), 3.62 (t, *J* = 6.5 Hz, 2H, H‑6), 2.09 – 2.01 (m, 2H, H‑4), 1.72 – 1.60 (m, 2H, H‑5), 1.46 (d, *J* = 1.1 Hz, 3H, H‑1’), 1.05 (s, 9H, SiC_q_(C*H*_3_)_3_).

**^13^C NMR** (101 MHz, CDCl_3_) δ 137.0 (C‑3), 135.8 (ArC), 134.2 (ArC_q_), 129.7 (ArC), 127.7 (ArC), 124.6 (C‑2), 62.8 (C‑6), 61.2 (C‑1), 35.9 (C‑4), 30.7 (C‑5), 27.0 (SiC_q_(*C*H_3_)_3_), 19.3 (Si*C*_q_(CH_3_)_3_), 16.3 (C‑1’).

The analytic data are in accordance with those reported in the literature.^S20^

#### (*E*)-6-((*tert*-Butyldiphenylsilyl)oxy)-4-methylhex-4-en-1-yl methanesulfonate (SI‑16)

|  |
| --- |

Et_3_N (1.97 mL, 14.1 mmol, 2.00 eq.) and MsCl (655 μL, 8.46 mmol, 1.20 eq.) were added at 0 °C to a solution of alcohol **SI‑15** (2.60 g, 7.05 mmol, 1.00 eq.) in CH_2_Cl_2_ (47 mL). The mixture was stirred for 2 h at this temperature and afterwards, the reaction was terminated by the addition of a sat. aqueous NaHCO_3_ solution and the aqueous phase was separated and extracted with CH_2_Cl_2_ (3 x). The combined organic layers were washed with an aqueous HCl solution (1 M), dried over MgSO_4_, filtered and concentrated under reduced pressure. The title compound was obtained as a pale yellow oil and used without further purification (3.05 g, 6.83 mmol, 97%).

**TLC**: *R*_F_ = 0.50 (SiO_2_; PE/EA 4:1).

**^1^H NMR** (400 MHz, CDCl_3_) δ 7.70 – 7.66 (m, 4H, ArH), 7.43 – 7.37 (m, 6H, ArH), 5.40 (tq, *J* = 6.2, 1.3 Hz, 1H, H‑2), 4.22 (dd, *J* = 6.3, 0.9 Hz, 2H, H‑1), 4.19 (t, *J* = 6.5 Hz, 2H, H‑6), 2.99 (s, 3H, SO_2_C*H*_3_), 2.08 (t, *J* = 7.6 Hz, 2H, H‑4), 1.90 – 1.78 (m, 2H, H‑5), 1.45 (d, *J* = 1.1 Hz, 3H, H‑1’), 1.04 (s, 9H, SiC_q_(C*H*_3_)_3_).

**^13^C NMR** (101 MHz, CDCl_3_) δ 135.7 (ArC), 135.2 (C‑3), 134.1 (ArC_q_), 129.7 (ArC), 127.8 (ArC), 125.6 (C‑2), 69.7 (C‑6), 61.1 (C‑1), 37.5 (C‑4), 35.1 (C‑5), 27.0 (SiC_q_(*C*H_3_)_3_), 19.3 (Si*C*_q_(CH_3_)_3_), 16.3 (C‑1’).

The analytic data are in accordance with those reported in the literature.^S20^

#### (*E*)-*tert*-Butyl((6-iodo-3-methylhex-2-en-1-yl)oxy)diphenylsilane (SI‑17)

|  |
| --- |

To a solution of **SI‑16** (3.05 g, 6.83 mmol, 1.00 eq.) in acetone (98 mL) was added sodium iodide (1.84 g, 12.3 mmol, 1.80 eq.). The mixture was heated to 50 °C and stirred overnight at the same temperature. In the following, the reaction mixture was diluted with a mixture of a sat. aqueous NaHCO_3_ solution and a sat. aqueous Na_2_SO_3_ solution (1:1). The solution was extracted with EA (3 x) and the combined organic layers were dried over MgSO_4_, filtered and concentrated *in vacuo*. The title compound was isolated as a pale yellow oil and used in the next step without further purification (3.03 g, 6.33 mmol, 93%).

**TLC**: *R*_F_ = 0.83 (SiO_2_; PE/EA 4:1).

**^1^H NMR** (400 MHz, CDCl_3_) δ 7.71 – 7.66 (m, 4H, ArH), 7.45 – 7.36 (m, 6H, ArH), 5.46 – 5.38 (m, 1H, H‑2), 4.22 (dd, *J* = 6.3, 0.9 Hz, 2H, H‑1), 3.13 (t, *J* = 7.0 Hz, 2H, H‑6), 2.06 (t, *J* = 7.1 Hz, 2H, H‑4), 1.95 – 1.83 (m, 2H, H‑5), 1.43 (d, *J* = 1.1 Hz, 3H, H‑1’), 1.05 (s, 9H, SiC_q_(C*H*_3_)_3_).

**^13^C NMR** (101 MHz, CDCl_3_) δ 135.7 (ArC), 135.1 (C‑3), 134.1 (ArC_q_), 129.7 (ArC), 127.8 (ArC), 125.6 (C‑2), 61.1 (C‑1), 40.0 (C‑4), 31.5 (C‑5), 27.0 (SiC_q_(*C*H_3_)_3_), 19.3 (Si*C*_q_(CH_3_)_3_), 16.3 (C‑1’), 6.5 (C‑6).

The analytic data are in accordance with those reported in the literature.^S20^

#### (*E*)-(6-((*tert*-Butyldiphenylsilyl)oxy)-4-methylhex-4-en-1-yl)iodotriphenyl-l5-phosphane (24)

|  |
| --- |

To iodide **SI‑17** (3.29 g, 7.34 mmol, 1.00 eq.) in dry toluene (63 mL) was added PPh_3_ (2.31 g, 8.81 mmol, 1.20 eq.) and the resulting yellow solution was stirred under refluxing conditions for 3 d. In the following, the solvent was removed under reduced pressure and the residue was purified by flash column chromatography (SiO_2_; CH_2_Cl_2_/MeOH 20:1) to yield **24** as a pale yellow foam (5.08 g, 6.86 mmol, 93%).

**TLC**: *R*_F_ = 0.41 (SiO_2_; CH_2_Cl_2_/MeOH 20:1).

**^1^H NMR** (400 MHz, CDCl_3_) δ 7.85 – 7.74 (m, 9H, ArH), 7.73 – 7.66 (m, 6H, ArH), 7.64 – 7.56 (m, 4H, ArH), 7.41 – 7.27 (m, 6H, ArH), 5.32 (td, *J* = 6.3, 1.3 Hz, 1H, H‑2), 4.15 (d, *J* = 7.1 Hz, 1H, H‑1), 3.72 – 3.60 (m, 2H, H‑6), 2.31 (t, *J* = 7.2 Hz, 2H, H‑4), 1.75 (dq, *J* = 15.7, 7.7 Hz, 2H, H‑5), 1.32 (d, *J* = 1.4 Hz, 3H, H‑1’), 0.97 (s, 9H, SiC_q_(C*H*_3_)_3_).

**^13^C NMR** (101 MHz, CDCl_3_) δ 135.6 (ArC), 135.2 (d, *J* = 3.0 Hz, Ar_P_C), 133.9 (ArC_q_), 133.8 (d, *J* = 10.0 Hz, Ar_P_C), 130.7 (d, *J* = 12.5 Hz, Ar_P_C), 129.7 (ArC), 127.7 (ArC), 126.1 (C‑2), 118.2 (d, *J* = 12.5 Hz, Ar_P_C_q_), 60.9 (C‑1), 39.2 (d, *J* = 15.6 Hz, C‑4), 26.9 (SiC_q_(*C*H_3_)_3_), 22.3 (d, *J* = 50.7 Hz), 20.4 (d, *J* = 3.7 Hz, C‑5), 19.2 (Si*C*_q_(CH_3_)_3_), 16.4 (C‑1’).

The analytic data are in accordance with those reported in the literature.^S19^

#### (*E*)-*tert*-Butyl((3-methyl-6-(oxetan-3-ylidene)hex-2-en-1-yl)oxy)diphenylsilane (SI‑18)

|  |
| --- |

To a solution of **24** (1.37 g, 1.85 mmol, 1.00 eq.) in dry THF (7.4 mL) was added *n*-BuLi (2.0 M in hexanes, 1.48 mL, 2.96 mmol, 1.60 eq.) at −78 °C and the resulting red mixture was stirred for 1 h at this temperature. Oxetan-3-one (**21**, 190 μL, 2.96 mmol, 1.60 eq.) in dry THF (3.0 mL) was added dropwise to the dark red solution and the reaction was stirred overnight at rt. The reaction was terminated by the addition of water and the suspension was extracted with EA (3 x). The combined organic layers were washed with brine, dried over MgSO_4_, filtered and the solvent was removed *in vacuo*. The title compound **SI‑18** was isolated after column chromatography (SiO_2_; PE/EA 20:1) as a colorless oil (506 mg, 1.24 mmol, 67%).

**TLC**: *R*_F_ = 0.72 (SiO_2_; PE/EA 4:1).

**^1^H NMR** (400 MHz, CDCl_3_) δ 7.72 – 7.61 (m, 4H, ArH), 7.47 – 7.33 (m, 6H, ArH), 5.36 (tq, *J* = 6.3, 1.3 Hz, 1H, H‑2), 5.26 – 5.12 (m, 4H, H‑8 and H‑10), 5.09 (ddp, *J* = 6.8, 4.5, 2.3 Hz, 1H, H‑6), 4.22 (dd, *J* = 6.3, 0.9 Hz, 1H, H‑1), 2.06 – 1.97 (m, 2H, H‑4), 1.98 – 1.84 (m, 2H, H‑5), 1.43 (s, 3H, H‑1’), 1.04 (s, 9H, SiC_q_(C*H*_3_)_3_).

**^13^C NMR** (101 MHz, CDCl_3_) δ 136.3 (C‑3), 135.8 (ArC), 134.2 (ArC_q_), 134.1 (C‑7), 129.7 (ArC), 127.7 (ArC), 124.8 (C‑2), 119.3 (C‑6), 79.6 (C‑8 or C‑10), 79.0 (C‑10 or C‑8), 61.2 (C‑1), 38.8 (C‑4), 27.0 (SiC_q_(*C*H_3_)_3_), 26.6 (C‑5), 19.3 (Si*C*_q_(CH_3_)_3_), 16.4 (C‑1’).

**ESI-MS** (ESI+) m/z Calculated for C_26_H_34_O_2_SiNa^+^ [M+Na]^+^ 429.2246; Found 429.2246.

#### (*E*)-3-Methyl-6-(oxetan-3-ylidene)hex-2-en-1-ol (25)

|  |
| --- |

To silyl ether **SI-18** (494 mg, 1.21 mmol, 1.00 eq.) in dry THF (3.0 mL) was added TBAF (1 M in THF, 1.94 mL, 1.94 mmol, 1.60 eq.) at 0 °C and the reaction mixture was stirred at rt for 1 d. The reaction was terminated by the addition of a sat. aqueous NH_4_Cl solution and the layers were separated. The aqueous phase was extracted with EA (3 x) and the combined organic layers were washed with brine, dried over Na_2_SO_4_, filtered and concentrated under reduced pressure. The residue was purified by flash column chromatography (SiO_2_; PE/EA 1:1) to yield alcohol **25** as a colorless oil (180 mg, 1.07 mmol, 88%).

**TLC**: *R*_F_ = 0.40 (SiO_2_; PE/EA 1:1).

**^1^H NMR** (400 MHz, CDCl_3_) δ 5.41 (tq, *J* = 6.9, 1.3 Hz, 1H, H‑2), 5.24 – 5.15 (m, 4H, H‑8 and H‑10), 5.11 (ddq, *J* = 7.1, 4.7, 2.3 Hz, 1H, H‑6), 4.16 (d, *J* = 7.4 Hz, 2H, H‑1), 2.07 (dd, *J* = 9.0, 6.3 Hz, 2H, H‑4), 2.02 – 1.93 (m, 2H, H‑5), 1.67 (d, *J* = 1.3 Hz, 3H, H‑1’).

**^13^C NMR** (101 MHz, CDCl_3_) δ 138.9 (C‑3), 134.3 (C‑7), 124.1 (C‑2), 119.1 (C‑6), 79.6 (C‑8 or C‑10), 78.9 (C‑10 or C‑8), 59.5 (C‑1), 38.9 (C‑4), 26.6 (C‑5), 16.4 (C‑1’).

**ESI-MS** (ESI+) m/z Calculated for C_10_H_16_O_2_Na^+^ [M+Na]^+^ 191.1048; Found 191.1046.

#### *tert*-Butyl((3-methylbut-2-en-1-yl)oxy)diphenylsilane (SI‑19)

|  |
| --- |

To a solution of prenol (1.70 g, 19.7 mmol, 1.00 eq.) in CH_2_Cl_2_ (25 mL) was added imidazole (3.36 g, 49.3 mmol, 2.50 eq.) and TBDPSCl (5.58 mL, 21.7 mmol, 1.10 eq.) at rt. The mixture was stirred for 2 h at this temperature and subsequently *n*‑pentane was added and stirring was continued for another 30 min. The organic layer was washed with water and brine, dried over MgSO_4_, filtered, the solvent was removed under reduced pressure and the title compound was isolated as a colorless oil. It was used without further purification (5.74 g, 17.7 mmol, 90%).

**TLC**: *R*_F_ = 0.95 (SiO_2_; PE/EA 8:1).

**^1^H NMR** (400 MHz, CDCl_3_) δ 7.73 – 7.67 (m, 4H, ArH), 7.43 – 7.35 (m, 6H, ArH), 5.38 (dddd, *J* = 6.4, 5.1, 2.8, 1.4 Hz, 1H, H‑2), 4.20 (d, *J* = 6.4 Hz, 2H, H‑1), 1.70 (d, *J* = 1.4 Hz, 3H, H‑4), 1.46 (d, *J* = 1.3 Hz, 3H, H‑1’), 1.05 (s, 9H, SiC_q_(C*H*_3_)_3_).

**^13^C NMR** (101 MHz, CDCl_3_) δ 135.8 (ArC), 134.2 (ArC_q_), 133.9 (C‑3), 129.6 (ArC), 127.7 (ArC), 124.3 (C‑2), 61.3 (C‑1), 27.0 (SiC_q_(*C*H_3_)_3_), 25.8 (C‑4), 19.3 (Si*C*_q_(CH_3_)_3_), 18.1 (C‑1’).

The analytic data are in accordance with those reported in the literature.^S14^

#### (*E*)-4-((*tert*-Butyldiphenylsilyl)oxy)-2-methylbut-2-en-1-ol (SI‑20)

|  |
| --- |

**Step 1: Allylic oxidation**

To a suspension of SeO_2_ (80.0 mg, 721 μmol, 0.10 eq.) and salicylic acid (99.6 mg, 721 μmol, 0.10 eq.) in CH_2_Cl_2_ (9.0 mL) was added *t*-BuOOH (2.53 mL, 25.2 mmol, 3.50 eq.) at rt. After stirring for 2 h, silyl ether **SI‑19** (2.34 g, 7.21 mmol, 1.00 eq.) was added and the mixture was stirred for another 2 d at this temperature. Subsequently, the reaction was stopped by the addition of water and a sat. aqueous NaHCO_3_ solution. The aqueous layer was extracted with CH_2_Cl_2_ (3 x) and the combined organic layers were washed with brine, dried over MgSO_4_, filtered and concentrated under reduced pressure.

**Step 2: Reduction**

The residue was dissolved in MeOH (9.0 mL) and cooled to 0 °C. NaBH_4_ (273 mg, 7.21 mmol, 1.00 eq.) was added and the mixture was stirred for 40 min at the same temperature. The solvent was evaporated *in vacuo* and the residue was dissolved in water and EA. The aqueous phase was separated and extracted with EA (3 x). The combined organic layers were washed with water and brine, dried over MgSO_4_, filtered and the solvent was removed under reduced pressure. The crude product was purified by flash column chromatography (SiO_2_; PE/EA 15:1) to give alcohol **SI‑20** as a colorless oil (1.09 g, 3.21 mmol, 45%).

**TLC**: *R*_F_ = 0.42 (SiO_2_; PE/EA 8:1).

**^1^H NMR** (400 MHz, CDCl_3_) δ 7.75 – 7.64 (m, 4H, ArH), 7.48 – 7.34 (m, 6H, ArH), 5.62 (ddq, *J* = 6.2, 4.8, 1.4 Hz, 1H, H‑2), 4.28 (dd, *J* = 6.2, 1.0 Hz, 2H, H‑1), 3.96 (d, *J* = 1.0 Hz, 1H, H‑4), 1.48 (s, 3H, H‑1’), 1.05 (s, 9H, SiC_q_(C*H*_3_)_3_).

**^13^C NMR** (101 MHz, CDCl_3_) δ 136.3 (C‑3), 135.8 (ArC), 134.0 (ArC_q_), 129.7 (ArC), 127.8 (ArC), 125.1 (C‑2), 68.4 (C‑4), 60.9 (C‑1), 27.0 (SiC_q_(*C*H_3_)_3_), 19.3 (Si*C*_q_(CH_3_)_3_), 13.9 (C‑1’).

The analytic data are in accordance with those reported in the literature.^S14^

#### (*E*)-((4-Bromo-3-methylbut-2-en-1-yl)oxy)(*tert*-butyl)diphenylsilane (13)

|  |
| --- |

To a solution of PPh_3_ (989 mg, 3.77 mmol, 1.20 eq.) and alcohol **SI‑20** (1.07 g, 3.14 mmol, 1.00 eq.) in CH_2_Cl_2_ (13 mL) was added NBS (615 mg, 3.46 mmol, 1.10 eq.) at 0 °C. The mixture was stirred for 2.5 h at this temperature and in the following diluted by the addition of Et_2_O. Stirring was continued for another 10 min. The reaction mixture was filtered over a pad of Celite® and eluted with Et_2_O. The filtrate was washed with water and brine, dried over MgSO_4_, filtered, the solvent was removed under reduced pressure. The crude product was purified by column chromatography (SiO_2_; PE/EA 20:1). The title compound **13** was isolated as a colorless oil (1.03 g, 2.54 mmol, 81%).

**TLC**: *R*_F_ = 0.77 (SiO_2_; PE/EA 8:1).

**^1^H NMR** (400 MHz, CDCl_3_) δ 7.73 – 7.62 (m, 4H, ArH), 7.43 – 7.34 (m, 6H, ArH), 5.79 (t, *J* = 5.9 Hz, 1H, H‑2), 4.23 (d, *J* = 5.9 Hz, 1H, H‑1), 3.93 (s, 2H, H‑4), 1.57 (d, *J* = 1.1 Hz, 3H, H‑1’), 1.04 (s, 9H, SiC_q_(C*H*_3_)_3_).

**^13^C NMR** (101 MHz, CDCl_3_) δ 135.7 (ArC), 133.7 (ArC_q_), 133.0 (C‑3), 130.4 (C‑2), 129.8 (ArC), 127.8 (ArC), 61.2 (C‑1), 40.8 (C‑4), 26.9 (SiC_q_(*C*H_3_)_3_), 19.3 (Si*C*_q_(CH_3_)_3_), 15.1 (C‑1’).

The analytic data are in accordance with those reported in the literature.^S14^

#### *tert*-Butyl(((*E*)-3-methyl-4-(((*E*)-3-methyl-6-(oxetan-3-ylidene)hex-2-en-1-yl)oxy)but-2-en-1-yl)oxy)diphenylsilane (SI‑21)

|  |
| --- |

To a suspension of NaH (90%, 34.2 mg, 1.43 mmol, 2.00 eq.) in dry THF (2.9 mL) was added a solution of alcohol **25** (120 mg, 713 μmol, 1.00 eq.) in dry THF (3.6 mL) at rt and the resulting mixture was stirred for 1 h at rt. A second solution containing TBAI (26.3 mg, 71.3 μmol, 0.10 eq.) and bromide **13** (374 mg, 927 μmol, 1.30 eq.) in dry THF (1.9 mL) was then added dropwise at rt and the reaction was stirred at this temperature for 3 d. The reaction was stopped by the addition of a sat. aqueous NH_4_Cl solution and the solution was extracted with EA (3 x). The combined organic layers were washed with brine, dried over Na_2_SO_4_, filtered, the solvent concentrated *in vacuo* and the residue purified by column chromatography (SiO_2_; PE/EA 20:1 to 10:1 to 1:1). Ether **SI-21** was isolated as a colorless oil (188 mg, 383 μmol, 54%).

**TLC**: *R*_F_ = 0.37 (SiO_2_; PE/EA 4:1).

**^1^H NMR** (400 MHz, CDCl_3_) δ 7.72 – 7.65 (m, 4H, ArH), 7.43 – 7.35 (m, 6H, ArH), 5.64 (td, *J* = 6.2, 1.3 Hz, 1H, H‑2), 5.34 (td, *J* = 6.5, 5.6, 3.0 Hz, 2H, H‑7), 5.24 – 5.14 (m, 4H, H‑13 and H‑15), 5.12 (ddt, *J* = 7.2, 4.9, 2.4 Hz, 1H, H‑11), 4.26 (d, *J* = 6.4 Hz, 1H, H‑1), 3.91 (d, *J* = 6.7 Hz, 2H, H‑6), 3.83 (s, 2H, H‑4), 2.15 – 2.04 (m, 2H, H‑9), 2.04 – 1.93 (m, 2H, H‑10), 1.64 (d, *J* = 1.4 Hz, 3H, H‑2’), 1.49 (d, *J* = 1.4 Hz, 3H, H‑1’), 1.04 (s, 9H, SiC_q_(C*H*_3_)_3_).

**^13^C NMR** (101 MHz, CDCl_3_) δ 139.2 (C‑8), 135.7 (ArC), 134.2 (C‑12), 134.0 (ArC_q_), 133.8 (C‑3), 129.7 (ArC), 127.8 (ArC), 127.4 (C‑2), 121.7 (C‑7), 119.2 (C‑11), 79.6 (C‑13 or C‑15), 79.0 (C‑15 or C‑13), 75.7 (C‑4), 66.1 (C‑6), 60.9 (C‑1), 38.9 (C‑9), 27.0 (SiC_q_(*C*H_3_)_3_), 26.6 (C‑10), 19.3 (Si*C*_q_(CH_3_)_3_), 16.6 (C‑2’), 14.2 (C‑1’).

**ESI-MS** (ESI+) m/z Calculated for C_31_H_42_O_3_SiNa^+^ [M+Na]^+^ 513.2801; Found 513.2790.

#### (*E*)-3-Methyl-4-(((*E*)-3-methyl-6-(oxetan-3-ylidene)hex-2-en-1-yl)oxy)but-2-en-1-ol (26)

|  |
| --- |

TBAF (1 M in THF, 825 μL, 825 μmol, 3.00 eq.) was added to a solution of silyl ether **SI‑21** (135 mg, 275 μmol, 1.00 eq.) in dry THF (3.3 mL) at 0 °C. The reaction mixture was allowed to reach rt and stirring was continued overnight. Upon completion, the reaction was stopped by the addition of a sat. aqueous NH_4_Cl solution and the mixture was extracted with EA (3 x). The combined organic layers were washed with brine, dried over Na_2_SO_4_, filtered and concentrated under reduced pressure. The crude product was purified via flash column chromatography (SiO_2_; PE/EA 1:1) to yield alcohol **26** as a colorless oil (69.4 mg, 239 μmol, 87%).

**TLC**: *R*_F_ = 0.26 (SiO_2_; PE/EA 1:1).

**^1^H NMR** (400 MHz, CDCl_3_) δ 5.64 (ddq, *J* = 6.7, 5.4, 1.4 Hz, 1H, H-2), 5.32 (tt, *J* = 5.4, 1.3 Hz, 1H, H‑7), 5.19 (d, *J* = 1.4 Hz, 1H, H-11), 5.16 – 5.12 (m, 2H, H-13 or H-15), 5.09 (tt, *J* = 7.1, 2.3 Hz, 1H, H-15 or H‑13), 4.18 (d, *J* = 6.7 Hz, 2H, H-1), 3.93 (d, *J* = 6.7 Hz, 2H, H-6), 3.84 (s, 2H, H-4), 2.09 – 2.02 (m, 3H, H-9), 1.99 – 1.93 (m, 2H, H-10), 1.68 (s, 3H, H‑1’), 1.63 (s, 3H, H-2’).

**^13^C NMR** (101 MHz, CDCl_3_) δ 139.3 (C-8), 135.7 (C-3), 134.1 (C-12), 126.2 (C-2), 121.6 (C-7), 119.1 (C‑11), 79.6 (C-13 or C-15), 78.9 (C-15 or C-13), 75.3 (C-4), 66.4 (C-6), 59.1 (C-1), 38.8 (C-9), 26.5 (C-10), 16.5 (C-2’), 14.1 (C-1’).

**ESI-MS** (ESI+) m/z Calculated for C_15_H_24_O_3_Na^+^ [M+Na]^+^ 275.1623; Found 275.1623.

#### Triammonium (*E*)-3-methyl-4-(((*E*)-3-methyl-6-(oxetan-3-ylidene)hex-2-en-1-yl)oxy)but-2-en-1-yl diphosphate (5c)

|  |
| --- |

**Step 1: Preparation of TEAP solution**

Solution A: Concentrated phosphoric acid (1.50 mL) dissolved in CH_3_CN (9.40 mL); Solution B: Freshly distilled Et_3_N (11.0 mL) dissolved in CH_3_CN (10.0 mL).

To freshly generate TEAP, solution A (0.91 mL) and solution B (1.50 mL) were mixed and stirred for 5 min at 37 °C.

**Step 2: Pyrophosphorylation**

To alcohol **26** (50.5 mg, 200 μmol, 1.00 eq.) in CCl_3_CN (500 μL) was added a freshly prepared TEAP solution in three portions (each 500 μL) in an interval of 5 min between each addition. After the final addition the orange solution was stirred for further 5 min and the reaction mixture was loaded on a flash column and eluted (SiO_2_; IPA/NH_3_/H_2_O 6:3:1). After a second flash column purification under identical conditions pyrophosphate **5c** was isolated as a pale yellow solid (20.4 mg, 44.0 μmol, 22%).

**TLC**: *R*_F_ = 0.23 (SiO_2_; IPA/NH_3_/H_2_O 6:3:1).

**^1^H NMR** (600 MHz, D_2_O) δ 5.69 (t, *J* = 6.8 Hz, 1H, H‑2), 5.36 (td, *J* = 7.3, 1.5 Hz, 1H, H‑7), 5.31 – 5.28 (m, 2H, H‑13 or H‑14), 5.26 – 5.23 (m, 1H, H‑11), 5.23 – 5.21 (m, 2H, H‑14 or H‑13), 4.53 (t, *J* = 6.9 Hz, 1H, H‑1), 4.03 (d, *J* = 7.2 Hz, 2H, H‑6), 3.97 (s, 2H, H‑4), 2.12 (t, *J* = 7.3 Hz, 2H, H‑9), 2.06 – 2.00 (m, 2H, H‑10), 1.72 (s, 3H, H‑1’), 1.67 (s, 3H, H‑2’).

**^13^C NMR** (151 MHz, D_2_O) δ 145.3 (C‑8), 134.8 (C‑12), 126.8 (d, *J* = 8.2 Hz, C‑2), 122.9 (C‑11), 122.3 (C‑7), 83.0 (C‑13 or C‑14), 82.3 (C‑14 or C‑13), 77.3 (C‑4), 68.4 (C‑6), 65.0 (d, *J* = 5.3 Hz, C‑1), 40.7 (C‑9), 28.4 (C‑10), 18.2 (C‑2’), 16.2 (C‑1’).

**^31^P NMR** (162 MHz, D_2_O) δ −9.6, −10.4.

**ESI-MS** (ESI−) m/z Calculated for C_15_H_25_O_9_P_2_^−^ [M−H]^−^ 411.0974; Found 411.0977.

#### Preparation of (*S*)-MTPA-ester 35a and (*R*)-MTPA-ester35b

|  |
| --- |

To determine the absolute stereochemistry of compound **35** (see section 1.3), a Mosher ester analysis was carried out. The NMR sample dissolved in C_6_D_6_ was divided in half and transferred to two 1 ml sample vials, and the solvent was carefully removed as far as possible in a gas stream. The esterifications were carried out with the two samples. Each sample was dissolved in CH_2_Cl_2_ (350 μL) and freshly distilled Et_3_N (9.49 μL, 68.1 μmol), 4-DMAP (4.99 mg, 40.8 μmol) and (*S*)-MTPA-Cl (50.3 μL, 68.1 μmol) or (*R*)-MTPA-Cl (50.3 μL, 68.1 μmol), respectively, were added and the mixture was stirred at rt overnight. The light-brown solution was extracted with a sat. aqueous NaHCO_3_ solution and the organic layer was separated. The solvent was removed under reduced pressure (45 °C, 650 mbar) and both diastereomeric esters were purified by column chromatography (SiO_2_; *n*‑pentane/Et_2_O 10:1). After removal of the solvent under reduced pressure (45 °C, 650 mbar) NMR samples were prepared as described in Section 1.3.

**(*S*)-MTPA-ester 35a**

**TLC**: *R*_F_ = 0.67 (SiO_2_; *n*‑pentane/Et_2_O 10:1).

**^1^H NMR** (600 MHz, C_6_D_6_) δ 7.79 (d, *J* = 7.5 Hz, 2H, ArH), 7.05 – 7.02 (m, 3H, ArH), 5.95 (dd, *J* = 7.2, 3.3 Hz, 1H, H‑1), 5.19 (dd, *J* = 6.9, 1.4 Hz, 1H, H‑2), 4.80 (ddt, *J* = 11.2, 4.6, 1.3 Hz, 1H, H‑6), 3.55 (d, *J* = 1.4 Hz, 3H, OCH_3_), 2.45 (tq, *J* = 8.6, 2.7 Hz, 1H, H‑11), 2.30 – 2.27 (m, 1H, H‑10), 2.26 – 2.22 (m, 1H, H‑8), 1.99 – 1.95 (m, 2H, H‑5 and H‑9), 1.87 – 1.83 (m, 1H, H‑5’), 1.82 – 1.79 (m, 3H, H‑4 and H‑12), 1.64 – 1.61 (m, 1H, H‑8’), 1.57 – 1.53 (m, 2H, H‑13), 1.44 – 1.42 (m, 1H, H‑9’), 1.37 (s, 3H, H‑15), 1.34 (s, 3H, H‑14), 1.30 – 1.29 (m, 1H, H‑12').

**^13^C NMR** (151 MHz, C_6_D_6_) δ 166.5 (C=O), 138.4 (C‑7), 133.6 (ArC_q_), 132.0 (C‑3), 129.6 (ArC), 128.7 (C‑2), 128.6 (ArC), 128.0 (ArC), 125.8 (C‑6), 124.5 (d, *J* = 288.7 Hz, C_q_*C*F_3_), 85.0 (*C*_q_CF_3_), 77.9 (C‑1), 55.7 (d, *J* = 1.7 Hz, OCH_3_), 44.5 (C‑11), 40.2 (C‑8), 39.7 (C‑10), 39.0 (C‑4), 35.7 (C‑9), 28.3 (C‑13), 26.0 (C‑5), 22.9 (C‑12), 18.9 (C‑15), 16.8 (C‑14).

**^19^F NMR** (376 MHz, C_6_D_6_) δ −71.23.

**ESI-MS** (ESI+) m/z Calculated for C_25_H_31_O_3_F_3_Na [M+Na]^+^ 459.2123; Found 459.2116.

Figure S1. Correlations in 2D NMR spectra of **35a-(S)-MTPA-ester**.

Figure S2. ^1^H-^1^H-NOESY NMR correlations of **(S)-MTPA-ester 35a**.

**(*R*)-MTPA-ester 35b**

**TLC**: *R*_F_ = 0.73 (SiO_2_; *n*‑pentane/Et_2_O 10:1).

**^1^H NMR** (500 MHz, C_6_D_6_) δ 7.73 (d, *J* = 7.8 Hz, 2H, ArH), 7.13 – 7.07 (m, 2H, ArH), 7.06 – 7.02 (m, 1H, ArH), 5.86 (dd, *J* = 7.0, 3.2 Hz, 1H, H‑1), 4.99 (d, *J* = 6.9 Hz, 1H, H‑2), 4.77 – 4.68 (m, 1H, H‑6), 3.46 (d, *J* = 1.3 Hz, 3H, OCH_3_), 2.47 (tq, *J* = 8.4, 3.0 Hz, 1H, H‑11), 2.32 (p, *J* = 9.7 Hz, 1H, H‑10), 2.16 (dt, *J* = 12.6, 3.7 Hz, 1H, H‑8), 2.00 – 1.90 (m, 3H, H‑5, H‑12 and H‑13), 1.88 – 1.84 (m, 1H, H‑5’), 1.84 – 1.79 (m, 2H, H‑4), 1.75 – 1.68 (m, 2H, H‑9 and H‑13’), 1.61 (dd, *J* = 12.6, 3.9 Hz, 1H, H‑8’), 1.56 (dd, *J* = 9.0, 2.1 Hz, 1H, H‑12’), 1.37 (s, 3H, H‑15), 1.36 (s, 3H, H‑14), 1.35 – 1.30 (m, 1H, H‑9').

**^13^C NMR** (126 MHz, C_6_D_6_) δ 166.4 (C=O), 138.3 (C‑7), 133.3 (ArC_q_), 131.8 (C‑3), 129.6 (ArC), 128.6 (C‑2), 128.4 (ArC), 128.0 (ArC), 125.7 (C‑6), 124.5 (d, *J* = 288.5 Hz, C_q_*C*F_3_), 85.4 (*C*_q_CF_3_), 78.3 (C‑1), 55.4 (d, *J* = 1.6 Hz, OCH_3_), 44.1 (C‑11), 40.2 (C‑8), 39.8 (C‑10), 38.9 (C‑4), 35.2 (C‑9), 28.5 (C‑13), 25.9 (C‑5), 23.2 (C‑12), 18.9 (C‑15), 16.8 (C‑14).

**^19^F NMR** (376 MHz, C_6_D_6_) δ −71.18.

**ESI-MS** (ESI+) m/z Calculated for C_25_H_31_O_3_F_3_Na [M+Na]^+^ 459.2123; Found 459.2118.

Figure S3. Correlations in 2D NMR spectra of **(R)-MTPA-ester 35b**.

#### 2.2 Biotransformation products

#### Terpenoid 35

|  |
| --- |

Following the general procedure of upscale biotransformations (see Section 1.4.5), Cop4-mediated biotransformation of **4a** yielded the title compound after purification by column chromatography (SiO_2_; *n*‑pentane/Et_2_O 20:1).

**TLC**: *R*_F_ = 0.29 (SiO_2_; *n*‑pentane/Et_2_O 20:1).

**^1^H NMR** (400 MHz, C_6_D_6_) δ 5.15 (dd, *J* = 6.8, 1.5 Hz, 1H, H‑2), 4.87 (ddt, *J* = 11.3, 4.5, 1.4 Hz, 1H, H‑4), 4.38 – 4.31 (m, 1H, H‑5), 2.38 (ddd, *J* = 11.0, 7.5, 3.3 Hz, 2H, H‑6 and H‑8), 2.32 (t, *J* = 3.8 Hz, 1H, H‑7), 2.26 – 2.17 (m, 1H, H‑11), 2.13 – 2.08 (m, 1H, H‑10), 2.07 – 2.04 (m, 1H, H‑12), 2.03 – 1.99 (m, 1H, H‑13), 1.97 – 1.88 (m, 2H, H‑11 and H‑12), 1.89 – 1.84 (m, 2H, H‑9), 1.82 (dq, *J* = 9.0, 1.6 Hz, 1H, H‑13), 1.73 (td, *J* = 12.7, 4.3 Hz, 1H, H‑7), 1.53 (t, *J* = 4.0 Hz, 1H, H‑10), 1.48 (t, *J* = 1.3 Hz, 3H, H‑14), 1.37 – 1.31 (m, 3H, H‑15).

**^13^C NMR** (101 MHz, C_6_D_6_) δ 138.6 (C‑1), 135.1 (C‑2), 128.2 (C‑3), 125.7 (C‑4), 72.8 (C‑5), 45.5 (C‑6), 40.6 (C‑7), 40.2 (C‑8), 39.2 (C‑9), 36.2 (C‑10), 29.7 (C‑11), 26.2 (C‑12), 23.2 (C‑13), 19.0 (C‑14), 16.7 (C‑15).

**CI-MS** (CI+) m/z Calculated for C_15_H_25_O [M+H]^+^ 221.1905; Found 221.1889.

Figure S4. Correlations in 2D NMR spectra of compound **35** (top) and details of the Mosher Ester analysis.^S21^ It allowed to determine the absolute stereochemistry (NOE analysis of compound **35** was hampered due to the overlap of the bridgehead protons H‑6 and H‑8).

#### Terpenoid 32

Following the general procedure of upscale biotransformations (see Section 1.4.5), BcBOT2-mediated biotransformation of **4a** yielded the title compound after purification by column chromatography (SiO_2_; *n*‑pentane/Et_2_O 10:1).

**TLC**: *R*_F_ = 0.49 (SiO_2_; *n*‑pentane/Et_2_O 10:1).

**mp** 68 °C

**^1^H NMR** (600 MHz, C_6_D_6_) δ 5.02 (d, *J* = 7.9 Hz, 1H, H‑2), 4.79 (dt, *J* = 11.7, 2.0 Hz, 1H, H‑6), 4.24 (dd, *J* = 8.1, 2.8 Hz, 1H, H‑1), 2.53 (ddtd, *J* = 11.2, 10.1, 9.1, 1.0 Hz, 1H, H‑13), 2.22 – 2.12 (m, 3H, H‑8, H‑10 and H‑12), 2.06 – 2.03 (m, 1H, H‑5), 2.03 – 2.00 (m, 1H, H‑8’), 1.99 – 1.89 (m, 2H, H‑4, H‑9 and H‑12’), 1.88 – 1.82 (m, 2H, H‑4’ and H‑5’), 1.79 – 1.72 (m, 1H, H‑11), 1.51 (dddd, *J* = 14.3, 5.3, 3.1, 1.9 Hz, 1H, H‑10’), 1.46 (ddtd, *J* = 11.4, 9.8, 4.0, 1.9 Hz, 1H, H‑13’), 1.39 (d, *J* = 1.2 Hz, 3H, H‑15), 1.33 (d, *J* = 1.3 Hz, 3H, H‑14).

**^13^C NMR** (126 MHz, C_6_D_6_) δ 134.7 (C‑1), 132.0 (C‑2), 130.2 (C‑3), 126.7 (C‑4), 70.9 (C‑5), 47.0 (C‑6), 41.3 (C‑7), 39.2 (C‑8), 35.0 (C‑9), 34.8 (C‑10), 28.0 (C‑11), 25.8 (C‑12), 21.3 (C‑13), 16.1 (C‑14), 15.4 (C‑15).

**CI-MS** (CI+) m/z Calculated for C_15_H_25_O [M] 220.1827; Found 220.1825.

**X-ray analysis:** Single colorless needle-shaped crystals of terpenoid **32** were crystallized by slow evaporation from deuterated benzene. A suitable crystal 0.69×0.09×0.03 mm^3^ was selected and mounted on a 18 mm mounted CryoLoop (20 micron, 0.2 - 0.3 mm, Hampton Research) on an XtaLAB AFC12 (RINC): Kappa single diffractometer. The crystal was kept at a steady *T* = 99.95(18) K during data collection. The structure was solved with the ShelXT^S22^ structure solution program using the Intrinsic Phasing solution method and by using **Olex2**^S23^ as the graphical interface. The model was refined with version 2019/3 of ShelXL 2019/3^S24^ using Least Squares minimization.

**Crystal Data.** C_45_H_72_O_3_, *M_r_* = 661.02, trigonal, *P*3_1_ (No. 144), a = 23.5511(15) Å, b = 23.5511(15) Å, c = 6.3246(6) Å, *α* = 90^°^, *β* = 90^°^, *γ* = 120^°^, *V* = 3038.0(5) Å^3^, *T* = 99.95(18) K, *Z* = 3, *Z'* = 1, *m*(Cu K*_a_*) = 0.494, 58195 reflections measured, 7908 unique (*R_int_* = 0.1373) which were used in all calculations. The final *wR_2_* was 0.3350 (all data) and *R_1_* was 0.1195 (I > 2(I)).


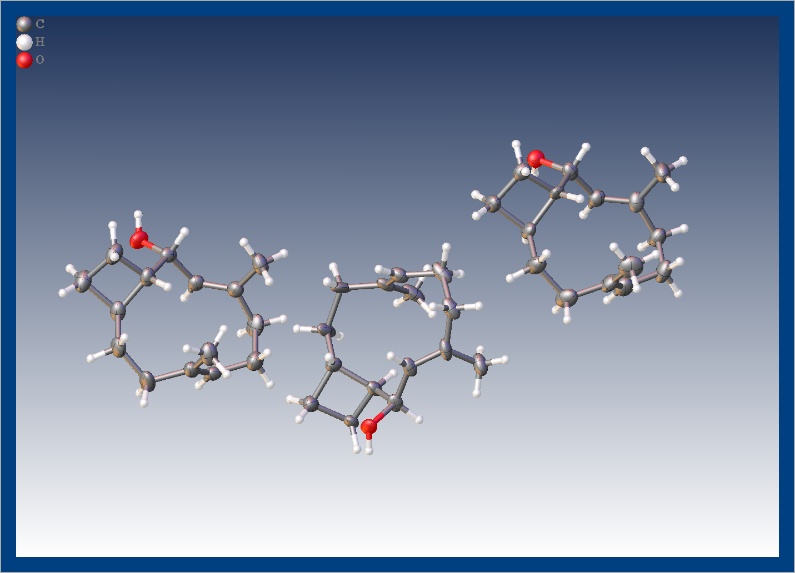


CCDC 2494283 contain the supplementary crystallographic data for this structure. These data can be obtained free of charge via www.ccdc.cam.ac.uk/structures/.

#### Terpenoid 33

|  |
| --- |

Following the general procedure of upscale biotransformations (see Section 1.4.5), Omp7-mediated biotransformation of **4a** yielded the title compound after purification by column chromatography (SiO_2_; *n*‑pentane).

**TLC**: *R*_F_ = 0.76 (SiO_2_; *n*‑pentane).

**^1^H NMR** (600 MHz, C_6_D_6_) δ 5.91 (dd, *J* = 9.6, 2.3 Hz, 1H, H‑6), 5.68 (d, *J* = 9.6 Hz, 1H, H‑4), 5.07 – 4.94 (m, 1H, H‑5), 2.83 – 2.77 (m, 1H, H‑11), 2.77 – 2.73 (m, 1H, H‑7), 2.45 – 2.40 (m, 1H, H‑11’), 2.40 – 2.34 (m, 1H, H‑8), 2.23 – 2.14 (m, 2H, H‑8 and H‑13), 2.14 – 2.11 (m, 1H, H‑12), 1.99 – 1.96 (m, 1H, H‑12’), 1.96 – 1.94 (m, 2H, H‑9), 1.93 – 1.86 (m, 1H, H‑10), 1.59 (s, 3H, H‑14), 1.41 – 1.37 (m, 1H, H‑10’), 1.34 (s, 3H, H‑15), 1.33 – 1.30 (m, 1H, H‑13).

**^13^C NMR** (151 MHz, C_6_D_6_) δ 145.0 (C‑1), 136.9 (C‑2), 128.8 (C‑3), 126.3 (C‑4), 125.6 (C‑5), 117.3 (C‑6), 44.5 (C‑7), 41.3 (C‑8), 38.5 (C‑9), 31.2 (C‑10), 29.9 (C‑11), 28.2 (C‑12), 27.4 (C‑13), 16.7 (C‑14), 14.9 (C‑15).

**CI-MS** (CI+) m/z Calculated for C_15_H_25_O [M] 202.1722; Found 202.1716.

Figure S5. Correlations in 2D NMR spectra of compound **33**.

#### Terpenoid 34

Following the general procedure of upscale biotransformations (see Section 1.4.5), Omp7-mediated biotransformation of **4a** yielded the title compound after purification by column chromatography (SiO_2_; *n*‑pentane).

**TLC**: *R*_F_ = 0.62 (SiO_2_; *n*‑pentane).

**^1^H NMR** (600 MHz, C_6_D_6_) δ 5.02 – 4.95 (m, 2H, H‑4), 2.64 – 2.54 (m, 1H, H‑13), 2.50 – 2.43 (m, 1H, H‑8), 2.43 – 2.37 (m, 1H, H‑13’), 2.19 (ddd, *J* = 15.9, 6.8, 1.7 Hz, 1H, H‑9), 2.10 (dddd, *J* = 14.2, 11.9, 2.2, 1.2 Hz, 1H, H‑11), 2.03 – 1.98 (m, 1H, H‑9’), 1.98 – 1.93 (m, 1H, H‑8’), 1.71 – 1.68 (m, 1H, H‑11’), 1.67 – 1.65 (m, 2H, H‑7, H‑10), 1.64 – 1.62 (m, 1H, H‑12), 1.57 – 1.55 (m, 1H, H‑5), 1.54 – 1.52 (m, 1H, H‑12’), 1.51 (dt, *J* = 2.1, 1.2 Hz, 3H, H‑14), 1.07 (s, 3H, H‑15), 1.04 – 1.00 (m, 1H, H‑10’).

**^13^C NMR** (151 MHz, C_6_D_6_) δ 150.4 (C‑1), 141.7 (C‑2), 127.4 (C‑3), 105.5 (C‑4), 54.7 (C‑5), 46.5 (C‑6), 44.3 (C‑7), 42.0 (C‑8), 34.4 (C‑9), 34.0 (C‑10), 32.7 (C‑11), 31.1 (C‑12), 23.8 (C‑13), 20.1 (C‑14), 16.7 (C‑15).

**CI-MS** (CI+) m/z Calculated for C_15_H_25_O [M] 202.1722; Found 202.1721.

Figure S6. Correlations in 2D NMR spectra of compound **34**.

#### Terpenoid 46

|  |
| --- |

Following the general procedure of upscale biotransformations (see Section 1.4.5) BcBOT2-mediated biotransformation of **5a** yielded the title compound after purification by column chromatography (SiO_2_; *n*‑pentane/Et_2_O 10:1).

**Odor**: camphoric, pepper.

**TLC**: *R*_F_ = 0.51 (SiO_2_; *n*‑pentane/Et_2_O 10:1).

**^1^H NMR** (500 MHz, C_6_D_6_) δ 4.96 (d, *J* = 1.1 Hz, 1H, H‑17), 4.87 (d, *J* = 1.6 Hz, 1H, H‑17’), 3.99 (d, *J* = 5.5 Hz, 2H, H‑4), 3.59 – 3.43 (m, 2H, H‑6), 2.27 (dt, *J* = 12.7, 4.6 Hz, 1H, H‑9), 2.22 – 2.18 (m, 1H, H‑2), 2.17 – 2.10 (m, 2H, H‑7 and H‑11), 2.03 (t, *J* = 9.9 Hz, 1H, H‑1), 1.59 (td, *J* = 12.0, 5.1 Hz, 1H, H‑9’), 1.34 (m, 1H, H‑1’), 1.21 – 1.12 (m, 1H, H‑10), 1.02 (s, 3H, H‑16), 1.01 – 0.98 (m, 1H, H‑10’), 0.58 – 0.30 (m, 2H, H‑14 or H‑15), 0.25 – 0.15 (m, 2H, H‑14 or H‑15).

**^13^C NMR** (151 MHz, C_6_D_6_) δ 149.9 (C‑8), 112.1 (C‑17), 79.1 (C‑4), 72.6 (C‑6), 58.3 (C‑7), 46.2 (C‑3), 44.4 (C‑2), 41.7 (C‑11), 37.5 (C‑9), 31.6 (C‑10), 30.6 (C‑8), 22.2 (C‑12), 22.0 (C‑16), 8.1 (C‑14 or C‑15), 7.9 (C‑14 or C‑15).

**CI-MS** (CI+) m/z Calculated for C_15_H_23_O [M+H]^+^ 219.1749; Found 219.1749.

Figure S7. Correlations in 2D NMR spectra of compound **46**.

Table S2. Comparison of ^13^C NMR chemical shifts (δ) in C_6_D_6_ of structural analogue **46** with **48** (the carbon atoms that make up the cyclopropane ring are excluded) (standard deviation of Δδ = 1.4 ppm).

| Carbon | **46** δ in ppm | **48**^[S14]^ δ in ppm | Δδ in ppm |
| --- | --- | --- | --- |
| C‑8 | 149.9 | 149.9 | 0.0 |
| C‑17 | 112.1 | 112.0 | 0.1 |
| C‑4 | 79.1 | 79.4 | −0.3 |
| C‑6 | 72.6 | 72.5 | 0.1 |
| C‑7 | 58.3 | 58.5 | −0.2 |
| C‑3 | 46.2 | 45.7 | 0.5 |
| C‑11 | 41.7 | 46.2 | −4.5 |
| C‑2 | 44.4 | 40.7 | 3.7 |
| C‑9 | 37.5 | 37.3 | 0.2 |
| C‑8 | 30.6 | 34.9 | −4.3 |
| C‑10 | 31.6 | 29.4 | 2.2 |
| C‑16 | 22.0 | 22.7 | −0.7 |

#### Terpenoid 36

Following the general procedure of upscale biotransformations (see Section 1.4.5) Omp7-mediated biotransformation of **4b** yielded the title compound after purification by column chromatography (SiO_2_; *n*‑pentane).

**TLC**: *R*_F_ = 0.88 (SiO_2_; *n*‑pentane).

**^1^H NMR** (600 MHz, C_6_D_6_) δ 5.97 (d, *J* = 9.7 Hz, 1H, H‑6), 5.82 (dt, *J* = 9.7, 1.2 Hz, 1H, H‑4), 5.06 (ddt, *J* = 10.4, 5.8, 1.5 Hz, 1H, H‑5), 2.60 (t, *J* = 6.7 Hz, 1H, H‑8), 2.41 – 2.34 (m, 1H, H‑12), 2.30 – 2.25 (m, 1H, H‑7), 2.24 – 2.20 (m, 1H, H‑12’), 2.20 – 2.16 (m, 1H, H‑13), 2.09 (td, *J* = 13.3, 3.0 Hz, 1H, H‑7’), 2.06 – 2.03 (m, 1H, H‑13’), 2.02 – 1.98 (m, 2H, H‑9), 1.67 – 1.64 (m, 1H, H‑10), 1.60 (s, 3H, H‑15), 1.59 – 1.51 (m, 3H, H‑11 and H‑14), 1.41 – 1.37 (m, 1H, H‑10’), 1.33 (s, 3H, H‑16), 1.17 – 1.12 (m, 1H, H‑11’).

**^13^C NMR** (126 MHz, C_6_D_6_) δ 149.2 (C-1), 137.0 (C-2), 129.5 (C-3), 128.4 (C-4), 125.8 (C-5), 116.6 (C‑6), 41.2 (C-7), 41.0 (C-8), 38.9 (C-9), 37.0 (C-10), 32.9 (C-11), 31.1 (C-12), 27.9 (C-13), 22.8 (C-14), 16.6 (C‑15), 15.7 (C‑16).

**CI-MS** (CI+) m/z Calculated for C_15_H_25_O [M] 216.1878; Found 216.1881.

Figure S8. Correlations in 2D NMR spectra of compound **36**.

#### Terpenoid 37

Following the general procedure of upscale biotransformations (see Section 1.4.5) BcBOT2-mediated biotransformation of **4b** yielded the title compound after purification by column chromatography (SiO_2_; *n*‑pentane/Et_2_O 5:1).

**TLC**: *R*_F_ = 0.16 (SiO_2_; *n*‑pentane/Et_2_O 5:1).

**^1^H NMR** (600 MHz, C_6_D_6_) δ 5.19 (d, *J* = 9.6 Hz, 1H, H4), 4.94 – 4.88 (m, 1H, H5), 2.25 – 2.18 (m, 2H, H6, H13), 2.17 – 2.10 (m, 3H, H6’, H9, H10), 2.06 – 2.00 (m, 2H, H9’, H13), 1.95 (dd, *J* = 12.9, 7.7 Hz, 1H, H7), 1.81 (ddddd, *J* = 13.0, 10.3, 9.0, 7.7, 2.4 Hz, 1H, H14), 1.75 – 1.69 (m, 2H, H7, H8), 1.66 – 1.62 (m, 1H, H14’), 1.50 (s, 3H, H16), 1.43 – 1.41 (m, 1H, H12), 1.40 (t, *J* = 1.5 Hz, 3H, H15), 1.37 – 1.35 (m, 1H, H12’), 1.34 – 1.32 (m, 1H, H10’), 0.94 – 0.88 (m, 1H, H11), 0.83 – 0.78 (m, 1H, H11’).

**^13^C NMR** (151 MHz, C_6_D_6_) δ 137.6 (C2), 133.3 (C3), 124.7 (C4), 124.0 (C5), 85.0 (C1), 49.2 (C8), 39.2 (C9), 38.4 (C7), 37.6 (C12), 35.6 (C6), 32.4 (C11), 30.5 (C10), 25.0 (C13), 20.7 (C14), 18.2 (C16), 16.1 (C15).

**CI-MS** (CI+) m/z Calculated for C_16_H_26_O [M−H_2_O] 216.1878; Found 216.1875.

Figure S9. Correlations in 2D NMR spectra in compound **37**.

#### Terpenoid 38

Following the general procedure of upscale biotransformations (see Section 1.4.5) Cop4-mediated biotransformation of **4b** yielded the title compound after purification by column chromatography (SiO_2_; *n*‑pentane/Et_2_O 20:1).

**TLC**: *R*_F_ = 0.80 (SiO_2_; *n*‑pentane).

**^1^H NMR** (600 MHz, C_6_D_6_) δ 6.37 (ddd, *J* = 17.6, 10.7, 0.7 Hz, 1H, H‑3), 5.26 (tt, *J* = 5.4, 1.8 Hz, 1H, H‑8), 5.21 (ddt, *J* = 17.6, 1.2, 0.6 Hz, 1H, H‑6), 5.19 – 5.15 (m, 1H, H‑8’), 5.00 – 4.99 (m, 2H, H‑7), 4.99 – 4.96 (m, 1H, H‑5), 2.64 – 2.55 (m, 4H, H‑11 and H‑12), 2.31 – 2.21 (m, 4H, H‑10 and H‑14), 2.12 – 2.00 (m, 4H, H‑9 and H‑13), 1.81 (tt, *J* = 8.2, 7.5 Hz, 2H, H‑15), 1.56 (d, *J* = 1.3 Hz, 3H, H‑16).

**^13^C NMR** (151 MHz, C_6_D_6_) δ 146.5 (C‑1), 139.7 (C‑2), 139.5 (C‑3), 135.3 (C‑4), 124.7 (C‑5), 120.6 (C‑6), 116.0 (C‑7), 113.1 (C‑8), 40.1 (C‑9), 31.9 (C‑10), 31.3 (C‑11), 29.5 (C‑12), 27.1 (C‑13), 27.1 (C‑14), 17.5 (C‑15), 16.1 (C‑16).

**CI-MS** (CI+) m/z Calculated for C_15_H_25_O [M] 216.1878; Found 216.1868.

Figure S10. Correlations in 2D NMR spectra of compound **38**.

#### Terpenoid 49

Following the general procedure of upscale biotransformations (see Section 1.4.5) Omp7-mediated biotransformation of **5b** yielded the title compound after purification by preparative GC.

Odor: camphoric, pepper.

**TLC**: *R*_F_ = 0.57 (SiO_2_; *n*‑pentane/Et_2_O 10:1).

**^1^H NMR** (600 MHz, C_6_D_6_) δ 4.88 (d, *J* = 31.4 Hz, 1H, H‑17), 3.99 – 3.92 (m, 2H, H‑6), 3.49 – 3.44 (m, 2H, H‑4), 2.32 (dt, *J* = 12.5, 4.7 Hz, 1H, H‑9), 2.15 (ddd, *J* = 7.1, 4.9, 1.1 Hz, 1H, H‑7), 2.10 – 2.08 (m, 1H, H‑13), 1.82 – 1.79 (m, 3H, H‑15, H‑2 and H‑14), 1.69 – 1.68 (m, 1H, H‑15’), 1.68 – 1.66 (m, 1H, H‑9), 1.65 (dd, *J* = 5.6, 1.8 Hz, 1H, H‑11), 1.63 – 1.61 (m, 1H, H‑13’), 1.59 – 1.55 (m, 3H, H‑1 and H‑10), 1.22 – 1.20 (m, 1H, H‑10), 0.94 (s, 3H, H‑16).

**^13^C NMR** (151 MHz, C_6_D_6_) δ 149.9 (C‑8), 112.0 (C‑17), 79.4 (C‑4), 72.5 (C‑6), 58.5 (C‑7), 45.7 (C‑3), 45.2 (C‑11), 43.5 (C‑12), 41.6 (C‑2), 37.3 (C‑9), 34.9 (C‑1), 33.7 (C‑14), 30.5 (C‑10), 29.1 (C‑13), 22.2 (C‑16), 17.6 (C‑15).

**EI-MS** (EI+) m/z Calculated for C_15_H_25_O [M] 232.1827; Found 232.1833.

Figure S11. Correlations in 2D NMR spectra of compound **49**.

Figure S12. ^1^H-^1^H-NOESY NMR spectrum highlighting the critical correlations for the structure elucidation of compound **49**.

Table S3. Comparison of ^13^C NMR chemical shifts (δ) in C_6_D_6_ of structural analogue **49** with **48** (the carbon atoms that make up the cyclobutane ring are excluded) (standard deviation of Δδ = 0.3 ppm).

| Carbon | **49** δ in ppm | **48**^[S14]^ δ in ppm | Δδ in ppm |
| --- | --- | --- | --- |
| C‑8 | 149.9 | 149.9 | 0.0 |
| C‑17 | 112.0 | 112.0 | 0.0 |
| C‑4 | 79.4 | 79.4 | 0.0 |
| C‑6 | 72.5 | 72.5 | 0.0 |
| C‑7 | 58.5 | 58.5 | 0.0 |
| C‑3 | 45.7 | 45.7 | 0.0 |
| C‑11 | 45.2 | 46.2 | −1.0 |
| C‑2 | 41.6 | 40.7 | 0.9 |
| C‑9 | 37.3 | 37.3 | 0.0 |
| C‑1 | 34.9 | 34.9 | 0.0 |
| C‑10 | 30.5 | 29.4 | 1.1 |
| C‑16 | 22.2 | 22.7 | −0.5 |

#### Terpenoid 50

Following the general procedure of upscale biotransformations (see Section 1.4.5) Omp7-mediated biotransformation of **5b** yielded the title compound after purification by column chromatography (SiO_2_; *n*‑pentane/Et_2_O 30:1).

**TLC**: *R*_F_ = 0.63 (SiO_2_; *n*‑pentane/Et_2_O 10:1).

**^1^H NMR** (400 MHz, C_6_D_6_) δ 4.52 – 4.43 (m, 1H, H‑6), 4.32 – 4.21 (m, 1H, H‑6’), 3.64 (d, *J* = 8.2 Hz, 1H, H‑4), 3.38 (d, *J* = 8.2 Hz, 1H, H‑4’), 2.21 – 2.09 (m, 2H, H‑9 and H‑13), 1.88 – 1.77 (m, 4H, H‑9’, H‑14, H‑14’ and H‑15), 1.77 – 1.71 (m, 3H, H‑2, H‑13 and H‑15), 1.53 – 1.50 (m, 2H, H‑1 and H‑2), 1.50 – 1.47 (m, 1H, H‑10), 1.45 – 1.40 (m, 1H, H‑1’), 1.37 (q, *J* = 1.3 Hz, 3H, H‑17), 1.23 – 1.17 (m, 1H, H‑10’), 1.12 (d, *J* = 0.9 Hz, 3H, H‑16).

**^13^C NMR** (101 MHz, C_6_D_6_) δ 144.5 (C‑7), 124.9 (C‑8), 81.2 (C‑4), 72.2 (C‑6), 48.2 (C‑11), 47.3 (C‑3), 45.6 (C‑12), 44.3 (C‑2), 35.5 (C‑1), 34.8 (C‑9), 32.5 (C‑14), 28.6 (C‑13), 28.2 (C‑10), 24.1 (C‑17), 17.9 (C‑15), 17.5 (C‑16).

**EI-MS** (EI+) m/z Calculated for C_16_H_4_O [M] 232.1827; Found 232.1838.

Figure S13. Correlations in 2D NMR spectra of compound **50**.

#### Terpenoid 51 (with 49 as by-product)

Following the general procedure of upscale biotransformations (see Section 1.4.5) Omp7-mediated biotransformation of **5b** yielded the title compound **51** (accompanied with **49** in a ratio of 2:1 as judged by NMR integration) after purification by column chromatography (SiO_2_; *n*‑pentane/Et_2_O 30:1). Preparative GC induced the decomposition of the title compound.

**TLC**: *R*_F_ = 0.57 (SiO_2_; *n*‑pentane/Et_2_O 10:1).

**^1^H NMR** (600 MHz, C_6_D_6_) δ 6.37 (dd, *J* = 10.2, 1.2 Hz, 1H, H‑2), 6.11 (dd, *J* = 10.3, 2.0 Hz, 2H, H‑1), 5.49 (ddq, *J* = 9.4, 5.5, 1.3 Hz, 1H, H‑7), 4.45 (dd, *J* = 13.2, 9.1 Hz, 1H, H‑6), 4.18 (d, *J* = 14.9 Hz, 1H, H‑4), 3.91 (dddt, *J* = 13.2, 5.6, 1.6, 0.8 Hz, 1H, H‑6’), 3.84 (d, *J* = 14.7 Hz, 1H, H‑4’), 2.62 (q, *J* = 6.5 Hz, 1H, H‑11), 2.40 – 2.34 (m, 1H, H‑13), 2.24 – 2.17 (m, 1H, H‑13’), 2.06 – 2.02 (m, 1H, H‑9), 1.91 – 1.86 (m, 1H, H‑9’), 1.67 – 1.56 (m, 3H, H‑14, H‑14’ and H‑15), 1.61 (s, 3H, H‑16), 1.54 (s, 3H, H‑17), 1.46 – 1.41 (m, 2H, H‑10), 1.38 – 1.36 (m, 1H, H‑15’).

**^13^C NMR** (151 MHz, C_6_D_6_) δ 150.4 (C‑12), 136.7 (C‑8), 131.3 (C‑3), 127.3 (C‑7), 126.7 (C‑2), 117.1 (C‑1), 78.5 (C‑4), 69.8 (C‑6), 40.0 (C‑11), 37.3 (C‑9), 35.5 (C‑10), 33.4 (C‑15), 31.6 (C‑13), 22.7 (C‑14), 16.5 (C‑17), 14.4 (C‑16).

**EI-MS** (EI+) m/z Calculated for C_15_H_25_O [M] 232.1827; Found 232.1837.

Figure S14. Correlations in 2D NMR spectra of compound **51** (in grey the proposed conformation in case of a (E)-configuration is shown).

#### Terpenoid 39

Following the general procedure of upscale biotransformations (see Section 1.4.5) BcBOT2-mediated biotransformation of **4c** yielded the title compound after purification by column chromatography (SiO_2_; *n*‑pentane/Et_2_O 1:1).

**TLC**: *R*_F_ = 0.30 (SiO_2_; *n*‑pentane/Et_2_O 1:1).

**^1^H NMR** (400 MHz, C_6_D_6_) δ 5.30 (tq, *J* = 6.5, 1.4 Hz, 1H, H‑5), 5.13 – 5.05 (m, 1H, H‑13), 4.90 – 4.84 (m, 1H, H‑9), 4.19 (d, *J* = 5.3 Hz, 2H, H‑1), 4.10 – 4.04 (m, 3H, H‑3, H‑3‘ and H‑4), 4.03 – 4.02 (m, 1H, H‑4‘), 2.12 – 2.05 (m, 2H, H‑8), 2.04 – 1.96 (m, 4H, H‑12, H‑11), 1.95 – 1.90 (m, 2H, H‑7), 1.48 (s, 3H, H‑15), 1.36 (s, 3H, H‑14).

**^13^C NMR** (101 MHz, C_6_D_6_) δ 137.4 (C‑2), 136.7 (C‑6), 133.1 (C‑10), 129.2 (C‑13), 125.8 (C‑9), 124.4 (C‑5), 76.5 (C‑3), 69.5 (C‑4), 60.5 (C‑1), 39.3 (C‑11), 38.8 (C‑7), 25.0 (C‑8), 24.1 (C‑12), 15.78 (C‑15), 15.76 (C‑14).

**CI-MS** (CI+) m/z Calculated for C_15_H_24_O_2_ [M] 236.1776; Found 236.1782.

Figure S15. Correlations in 2D NMR spectra of compound **39**.

#### Terpenoid 40

Following the general procedure of upscale biotransformations (see Section 1.4.5) BcBOT2-mediated biotransformation of **4c** yielded the title compound after purification by column chromatography (SiO_2_; *n*‑pentane/Et_2_O 1:1).

**TLC**: *R*_F_ = 0.19 (SiO_2_; *n*‑pentane/Et_2_O 1:1).

**^1^H NMR** (600 MHz, C_6_D_6_) δ 5.54 – 5.51 (m, 1H, H‑6), 4.48 (ddd, *J* = 10.9, 3.8, 1.4 Hz, 1H, H‑2‘), 4.13 (d, *J* = 12.0 Hz, 1H, H‑1‘), 3.77 (ddd, *J* = 12.2, 2.4, 1.2 Hz, 1H, H‑1), 3.27 (t, *J* = 10.9 Hz, 1H, H‑2), 2.29 – 2.20 (m, 1H, H‑7), 1.84 – 1.79 (m, 1H, H‑8), 1.78 – 1.74 (m, 1H, H‑4), 1.64 – 1.59 (m, 2H, H‑7‘ and H‑11), 1.56 (td, *J* = 11.2, 3.8 Hz, 1H, H‑3), 1.43 – 1.39 (m, 1H, H‑12), 1.39 – 1.36 (m, 1H, H‑9), 1.34 – 1.29 (m, 1H, H‑10), 1.26 – 1.23 (m, 1H, H‑8‘), 1.13 (td, *J* = 13.1, 3.8 Hz, 1H, H‑12‘), 0.86 (s, 3H, H‑14), 0.82 (d, *J* = 6.6 Hz, 3H, H‑15), 0.76 – 0.72 (m, 1H, H‑11‘).

**^13^C NMR** (151 MHz, C_6_D_6_) δ 139.2 (C‑5), 126.4 (C‑6), 76.1 (C‑1), 70.6 (C‑13), 68.5 (C‑2), 52.3 (C‑3), 48.3 (C‑4), 43.0 (C‑10), 42.4 (C‑12), 37.2 (C‑9), 36.6 (C‑8), 29.6 (C‑11), 22.4 (C‑7), 21.2 (C‑15), 21.0 (C‑14).

**CI-MS** (CI+) m/z Calculated for C_15_H_24_O_2_ [M] 236.1776; Found 236.1783.

Figure S16. Correlations in 2D NMR spectra of compound **40**.

#### Terpenoids 41 and 42

Following the general procedure of upscale biotransformations (see Section 1.4.5) BcBOT2-mediated biotransformation of **4c** yielded the title compound after purification by column chromatography (SiO_2_; *n*‑pentane/Et_2_O 10:1).

**TLC**: *R*_F_ = 0.44 (SiO_2_; *n*‑pentane/Et_2_O 10:1).

**Terpenoid 41**

**^1^H NMR** (600 MHz, C_6_D_6_) δ 5.47 – 5.43 (m, 1H, H‑10), 5.37 – 5.32 (m, 1H, H‑4), 4.30 – 4.28 (m, 1H, H‑1), 4.13 (d, *J* = 11.5 Hz, 1H, H‑13), 3.85 – 3.80 (m, 1H, H‑13’), 3.12 (t, *J* = 10.9 Hz, 1H, H‑1’), 2.41 – 2.36 (m, 1H, H‑2), 2.35 – 2.30 (m, 1H, H‑9), 2.00 – 1.90 (m, 1H, H‑5), 1.84 – 1.81 (m, 1H, H‑12), 1.81 – 1.78 (m, 1H, H‑8), 1.74 – 1.70 (m, 1H, H‑9’), 1.60 – 1.54 (m, 2H, H‑5’ and H‑6), 1.47 – 1.45 (m, 1H, H‑7), 1.42 – 1.40 (m, 3H, H‑14), 1.22 – 1.20 (m, 1H, H‑8’), 0.78 (d, *J* = 6.8 Hz, 3H, H‑15).

**^13^C NMR** (151 MHz, C_6_D_6_) δ 138.5 (C‑11), 132.8 (C‑3), 124.1 (C‑4), 123.0 (C‑10), 76.7 C‑13), 72.0 (C‑1), 52.5 (C‑12), 47.5 (C‑2), 39.3 (C‑6), 37.7 (C‑8), 34.9 (C‑7), 32.2 (C‑5), 24.5 (C‑9), 20.4 (C‑15), 20.3 (C‑14).

**Terpenoid 42**

**^1^H NMR** (600 MHz, C_6_D_6_) δ 5.52 – 5.49 (m, 1H, H‑10), 4.63 (d, *J* = 1.6 Hz, 1H, H‑14), 4.33 (d, *J* = 1.6 Hz, 1H, H‑14‘), 4.27 – 4.25 (m, 1H, H‑1), 4.12 – 4.09 (m, 1H, H‑13), 3.77 (d, *J* = 12.6 Hz, 1H, H‑13‘), 3.50 (t, *J* = 10.8 Hz, 1H, H‑1‘), 2.30 – 2.24 (m, 1H, H‑9), 2.20 – 2.12 (m, 1H, H‑12), 2.13 (dt, *J* = 13.0, 3.6 Hz, 1H, H‑4), 1.86 – 1.83 (m, 1H, H‑4‘), 1.80 – 1.79 (m, 1H, H‑8), 1.77 – 1.74 (m, 2H, H‑5 and H‑2), 1.63 – 1.60 (m, 1H, H‑9‘), 1.52 – 1.50 (m, 1H, H‑6), 1.42 – 1.41 (m, 1H, H‑7), 1.23 – 1.22 (m, 1H, H‑8), 0.90 – 0.87 (m, 1H, H‑5), 0.80 (d, *J* = 6.9 Hz, 3H, H‑15).

**^13^C NMR** (151 MHz, C_6_D_6_) δ 149.3 (C‑3), 138.7 (C‑11), 125.7 (C‑10), 104.3 (C‑14), 76.3 (C‑13), 70.7 (C‑1), 53.7 (C‑2), 46.1 (C‑12), 42.0 (C‑6), 36.8 (C‑8), 36.7 (C‑4), 36.4 (C‑7), 33.9 (C‑5), 22.5 (C‑9), 20.9 (C‑15).

**CI-MS** (CI+) m/z Calculated for C_15_H_25_O [M] 218.1671; Found 218.1679.

Figure S17. Correlations observed in 2D NMR spectra of compound **41** and **42** (anti-anti relation within the six membered ring was assigned as done for compound **40**; the triplet multiplicity of H‑12 is taken as indicative information).

Figure S18. ^1^H-^1^H-COSY NMR spectrum highlighting the critical correlations for the structure elucidation of compound **41**.

Figure S19. ^1^H-^1^H-COSY NMR spectrum highlighting the critical correlations for the structure elucidation of compound **42**.

#### Terpenoid 43

Following the general procedure of upscale biotransformations (see Section 1.4.5) PenA-mediated biotransformation of **4c** yielded the title compound after purification by column chromatography (SiO_2_; *n*‑pentane/Et_2_O 10:1).

**TLC**: *R*_F_ = 0.42 (SiO_2_; *n*‑pentane/Et_2_O 10:1).

**^1^H NMR** (600 MHz, C_6_D_6_) δ 4.81 (d, *J* = 1.8 Hz, 1H, H‑2), 4.50 – 4.41 (m, 4H, H‑3 and H‑4), 2.42 (dp, *J* = 8.8, 2.2 Hz, 1H, H‑7), 2.28 (d, *J* = 9.5 Hz, 1H, H‑6), 1.74 – 1.68 (m, 3H, H‑9, H‑11, and H13), 1.64 – 1.60 (m, 1H, H‑10), 1.58 (d, *J* = 13.0 Hz, 1H, H‑9’), 1.56 – 1.52 (m, 1H, H‑12), 1.48 – 1.46 (m, 1H, H‑11’), 1.44 (s, 3H, H‑15), 1.26 – 1.18 (m, 2H, H‑12 and H‑13), 0.78 (d, *J* = 7.0 Hz, 1H, H‑14).

**^13^C NMR** (151 MHz, C_6_D_6_) δ 142.5 (C‑1), 127.6 (C‑2), 83.4 (C‑3 or C‑4), 79.9 (C‑3 or C‑4), 63.7 (C‑5), 61.3 (C‑6), 58.3 (C‑7), 47.4 (C‑8), 45.7 (C‑9), 44.1 (C‑10), 41.9 (C‑11), 33.4 (C‑12), 29.2 (C‑13), 16.4 (C‑14), 15.5 (C‑15).

**CI-MS** (CI+) m/z Calculated for C_15_H_22_O [M] 218.1671; Found 218.1679.

Figure S20. Correlations in 2D NMR spectra of compound **43**.

Table S4. Comparison of ^13^C NMR chemical shifts (δ) in C_6_D_6_ of structural analogue **43** with **28** (the carbon atoms that make up the oxetane ring are excluded) (standard deviation of Δδ = 0.3 ppm).

| Carbon | **43** δ in ppm | **28**^S25^ δ in ppm | Δδ in ppm |
| --- | --- | --- | --- |
| C1 | 142.5 | 140.7 | 1.8 |
| C2 | 127.5 | 130.1 | −2.6 |
| C3 | 63.7 | 62.1 | 1.6 |
| C4 | 61.2 | 62.4 | −1.2 |
| C5 | 58.3 | 59.9 | −1.6 |
| C6 | 47.4 | 40.7 | 6.7 |
| C7 | 45.8 | 49.3 | −3.5 |
| C8 | 44.1 | 45.0 | −0.9 |
| C9 | 41.9 | 47.1 | −5.2 |
| C10 | 33.4 | 33.8 | −0.4 |
| C11 | 29.2 | 27.9 | 1.3 |
| C12 | 16.2 | 17.2 | −1.0 |
| C13 | 15.7 | 15.6 | 0.1 |

#### Terpenoid 44

Following the general procedure of upscale biotransformations (see Section 1.4.5) PenA-mediated biotransformation of **4c** yielded the title compound after purification by column chromatography (SiO_2_; *n*‑pentane/Et_2_O 10:1).

**TLC**: *R*_F_ = 0.68 (SiO_2_; *n*‑pentane/Et_2_O 10:1).

**^1^H NMR** (600 MHz, C_6_D_6_) δ 5.61 (q, *J* = 2.5 Hz, 1H, H‑2), 4.63 (d, *J* = 5.6 Hz, 1H, H­‑3 or H‑4), 4.54 (dd, *J* = 5.6, 1.4 Hz, 1H, H‑3 or H‑4), 4.48 (d, *J* = 5.4 Hz, 1H, H‑3 or H‑4), 4.23 (d, *J* = 5.5 Hz, 1H, H‑3 or H‑4), 2.48 (ddd, *J* = 14.1, 4.7, 2.0 Hz, 1H, H‑8), 2.27 – 2.18 (m, 2H, H‑5 and H‑7), 1.71 (ddt, *J* = 12.2, 6.5, 3.1 Hz, 1H, H‑9), 1.59 (dddd, *J* = 10.6, 8.4, 6.3, 2.5 Hz, 1H, H‑10), 1.51 – 1.44 (m, 2H, H‑8’ and H‑10’), 1.01 (d, *J* = 6.5 Hz, 3H, H‑15), 0.99 – 0.93 (m, 1H, H‑9’), 0.78 (s, 3H, H‑13), 0.47 – 0.41 (m, 2H, H‑12 and H‑14), 0.26 – 0.23 (m, 1H, H‑12’).

**^13^C NMR** (151 MHz, C_6_D_6_) δ 150.8 (C‑1), 123.7 (C‑2), 84.8 (C‑3), 81.8 (C‑4), 46.9 (C‑5), 45.4 (C‑6), 41.5 (C‑7), 36.8 (C‑8), 35.3 (C‑9), 26.5 (C‑10), 23.2 (C‑11), 22.0 (C‑12), 21.1 (C‑13), 20.0 (C‑14), 18.8 (C‑15).

**^13^C NMR** (151 MHz, CDCl_3_) δ 151.3 (C‑1), 123.0 (C‑2), 85.5 (C‑3), 82.6 (C‑4), 46.9 (C‑5), 45.3 (C‑6), 41.3 (C‑7), 36.7 (C‑8), 35.1 (C‑9), 26.4 (C‑10), 23.3 (C‑11), 21.9 (C‑12), 21.2 (C‑13), 19.9 (C‑14), 18.7 (C‑15).

**CI-MS** (CI+) m/z Calculated for C_15_H_21_ [M−H_2_O+H] 201.1643; Found 201.1651.

Figure S21. Correlations observed in 2D NMR spectra of compound **44**.

Table S5. Comparison of ^13^C NMR chemical shifts (δ) in CDCl_3_ of structural analogue **44** with african-1-ene (**45**) (standard deviation of Δδ = 2.2 ppm).

| Carbon | **44** δ in ppm | **45**^S26^ δ in ppm | Δδ in ppm |
| --- | --- | --- | --- |
| C1 | 151.3 | 145.9 | 5.4 |
| C2 | 123.0 | 129.0 | 6.0 |
| C5 | 46.9 | 46.4 | 0.5 |
| C7 | 41.3 | 40.9 | 0.4 |
| C8 | 36.7 | 40.6 | −3.9 |
| C9 | 35.1 | 37.3 | −2.2 |
| C10 | 26.4 | 26.4 | 0.0 |
| C11 | 23.3 | 22.6 | 0.7 |
| C12 | 21.9 | 21.8 | 0.1 |
| C13 | 21.2 | 28.0 | −6.8 |
| C14 | 19.9 | 20.6 | −0.7 |
| C15 | 18.7 | 18.8 | −0.1 |

# Computational Section

**Molecular Modelling**

All mechanistic based substrate docking studies were performed using MOE 2024.06.^S27^ The 3D structures of the substrates **4a-c**, **5a**, **5b** and all the carbocationic intermediates were constructed using ‘Builder’ application in MOE and prepared according to the default protocol of ligand preparation in MOE. Geometry optimizations were performed using B3LYP/6-31G in Gaussian 16.^S28^ The X-ray crystal structures of BcBOT2 (PDB ID: 8H6U)^S29^ and PenA (PDB ID: 1PS1)^S30^ have been used for the docking experiment. The missing loop and residues in PenA were modelled using the structure (PDB ID: 6WKD)^S31^ as a template. The initial position of FPP **1** was manually modelled and refined by docking. For Omp7, homology modelling was performed in YASARA software^S32^ using (PDB ID 8H72) as a template with coverage about 95% and alignment score of 990. All 3D structures were prepared using ‘Quick Prep’ default parameters implemented in MOE. Prior to substrate docking, the active pocket was selected in the receptor molecule where the pyrophosphate of the substrates binds at least two Mg^2+^ ions. The substrates were docked using ‘Dock’ tool in MOE. For each of the ligands, 1000 poses were taken into consideration according to London ∆G Score criteria of which the top 100 poses were selected for visualization based on GBVI/WSA ∆G Score criteria. For all ligands, “Triangle Matcher” was chosen for placement, and the “Induced Fit” method was used for refinement of ligand poses, allowing the interaction of flexible side chains at the active site with the substrates.

The final docking pose(s) for each substrate were selected from the top 100 poses and evaluated by comparing them to the catalytically competent binding pose of FPP (**1**) considering the near attack conformation (NAC) criteria for the ring closure (Figure S1). Based on the reported reaction mechanism, the NAC geometric criteria are defined for each pose to have ionic interactions between the pyrophosphate group and the Mg^2+^ ions and to pose interatomic C-C distance cyclization of about 4.0 Å.

**Molecular Dynamics (MD) Simulations**

To re-evaluate the different binding modes; the FPP-opposite binding mode C1 and the FPP-aligned conformation C2 of **4c** in the BcBOT2 activate site predicted by the docking experiment, we performed molecular dynamics (MD) simulations for the evaluation of how much stable are such conformations within the activate site over 3 µs simulation for each mode. MD simulations were performed using the GPU version of the PMEMD engine integrated with Amber22.^S33^ The amber FF19SB force field was used in all the simulations, and periodic boundary conditions were employed in all simulations.^S34^ Long-range electrostatic interactions were calculated using the Particle Mesh Ewald (PME) method with a direct space and vdW cutoff of 10 Å.^S35^ The minimized systems were first heated for 100 ps by linearly increasing the temperature from 0 to 300 K in a canonical ensemble (NVT) using the Langevin thermostat.^S36^ The heated systems were further subjected to constant temperature heating (at 300 K) for 1 ns in an NPT ensemble. The solute molecules were restrained with the harmonic potential of 10 kcal/(molÅ^2^) during the heating processes. Thereafter, systems were equilibrated within an NPT ensemble at a fixed temperature and pressure of 300 K and 1 bar, respectively, for 4 ns without any restraints on solute molecules. The MD productive runs were performed, in three independent replicates for each conformation separately, for 1000 ns each in an NPT ensemble with a target pressure set at 1 bar and constant pressure coupling of 2 ps. The pressure was held constant using the Berendsen barostat, and the SHAKE algorithm was used to constrain the bond lengths of those bonds involving hydrogen atoms.^S37,S38^ Trajectories were analysed using CPPTRAJ and VMD.^S39,S40^ The MD simulations quality were evaluated by the analyses of the root mean square deviation (RMSD) of the enzymes with respect to the docking-predicted structure, root mean square fluctuations (RMSFs) and the time-evolved hydrogen bonds. The key catalytic distance for NAC was measured between the involved atoms in all simulations and plotted with a Python script.

Figure S22. Close-up depiction of the BcBOT2 pocket, showcasing the catalytic conformations of substrates **4a** and **4b** within the active site. In panel (**A**), the top-scoring docking pose of **4a** satisfies the necessary NAC distance requirements for (11→1) ring formation at 3.42 Å, thereby facilitating the subsequent cationic cascade that leads to the formation of product **32**. Panel (**B**) illustrates a comparable catalytic binding orientation for **4b**, with an (11→1) distance of approximately 3.88 Å, which follows the same enzymatic pathway to yield product **36**. Notably, both substrates exhibit favorable alignment within the active site, akin to the natural substrate **FPP** (**1**).

Figure S23. Close-up view of the BcBOT2 pocket, showcasing the docking poses for the catalytic path of substrates **4a** and the subsequent carbocations **52a**, **53a** and **53c** and product **32**. The 3D representation reveals that the top scoring poses for **4a** carbocations and its product exhibit favorable alignment within the active site of BcBOT2 enzyme.

Figure S24. The 2D representation of the docking poses for **4a** path in BcBOT2 active site. These representations reveal the importance of amino acids residues **Phe99** and **Ile204** in the stabilization of the proposed carbocations through π‑system‑cation interactions. It is shown that all the carbocations exhibit a favorable alignment within the active site without any major shifts.

Figure S25. Close-up view of the BcBOT2 pocket, showcasing the docking poses for the catalytic path of substrates **4b** and the subsequent carbocations **52b**, **53b** along with product **37**. The 3D representation reveals that the top scoring poses for **4b** and the subsequent carbocations along with product **37** exhibit favorable alignment within the active site of BcBOT2.

Figure S26. The 2D representation of the docking poses for **4b** path in the BcBOT2 active site. These representations reveal the importance of amino acids residues **Phe99** and **Ile204** in the stabilization of the proposed carbocations through π‑system‑cation interactions. It is shown that all the carbocations exhibit a favorable alignment within the active site without any major shifts.

Figure S27. Close-up depiction of the PenA pocket, showcasing the catalytic conformations of the substrates **4a** and **4b** within the active site. In panel (**A**), the top-scoring docking pose of **4a** satisfies the necessary NAC distance requirements for (11→1) ring formation at 4.17 Å, thereby facilitating the subsequent cationic cascade that leads to the formation of product **33**. Panel (**B**) illustrates a comparable catalytic binding orientation for **4b**, with an (11→1) distance of approximately 4.5 Å, which follows the same enzymatic pathway to yield product **36**. Notably, both substrates exhibit favorable alignment within the active site, akin to the natural substrate **FPP** (**1**).

Figure S28. Close-up view of the PenA pocket, showcasing the docking poses for the catalytic path of substrates **4a** and the subsequent carbocations **52a**, **53a** and product **33**. The 3D representation reveals that the top scoring poses for **4a** carbocations and its product exhibit favorable alignment within the active site of PenA.

Figure S29. The 2D representation of the docking poses for **4a** path in PenA active site. These representations reveal the importance of amino acids residues **Phe76, Phe77** and **Ile177** in the stabilization of the proposed carbocations through π‑system‑cation interactions. It is shown that all the carbocations exhibit a favorable alignment within the active site without any major shifts.

Figure S30. Close-up view of the PenA pocket, showcasing the docking poses for the catalytic path of substrates **4b** and the subsequent carbocations **52b**, **53b** along with product **36**. The 3D representation reveals that the top scoring poses for **4b** and the subsequent carbocations along with product **36** exhibit favorable alignment within the active site of PenA.

Figure S31. The 2D representation of the docking poses for **4b** path in PenA active site. These representations reveal the importance of amino acids residues **Phe76** and **Phe77** in the stabilization of the proposed carbocations through π‑system‑cation interactions. It is shown that all the carbocations exhibit a favorable alignment within the active site without any major shifts.

Figure S32. Close-up view of the Omp7 pocket, showcasing the docking poses for the catalytic path of substrates **4a** (**A**) and **4b** (**B**) with the NAC distance close to 4.0 Å for (11→1) cyclization.

Figure S33. The electrostatic properties of the active sites of BcBOT2 (panel **A**) and PenA (panel **B**). The BcBOT2 active site displays more localized negative charges compared to the more extensive negative electrostatic regions in the PenA active site. These contrasting electrostatic environments enable compound **4c** to adopt two distinct catalytic conformations within each enzyme's active site, resulting in divergent product formation pathways.

Figure S34. The pocket volume of the active sites of BcBOT2 (panel **A**) and PenA (panel **B**). The BcBOT2 active site displays a tighter volume (575 Å^3^) compared to the larger one in the PenA active site (1450 Å^3^). These contrasting pocket sizes also enable compound **4c** to adopt two distinct catalytic conformations within each enzyme's active site, resulting in divergent product formation pathways.

Figure S35. Close-up view of the BcBOT2 pocket, showcasing the docking poses for the catalytic path of substrates **4c** and the subsequent carbocations **57**, **58** and **59**.

Figure S36. The 2D representation of the docking poses for **4c** path in BcBOT2 active site. These representations reveal the importance of amino acids residues **Phe99** and **Ile204** in the stabilization of the proposed carbocations through π‑system‑cation interactions. It is shown that all the carbocations exhibit a favorable alignment within the active site without any major shifts.

Figure S37. Evaluation of the quality of MD simulation for the different three runs for conformation 1 (C1) of **4c** in BcBOT2. The backbone RMSD in panel (**A**) and the RMSF in panel (**B**) show good stability of the simulated system (**4c**-BcBOT2, conformation 1) over the 3 different runs.

Figure S38. Evaluation of the quality of MD simulation for the different three runs for conformation 2 (C2) of **4c** in BcBOT2. The backbone RMSD in panel (**A**) and the RMSF in panel (**B**) show good stability of the simulated system (**4c**-BcBOT2, conformation 2) over the 3 different runs with the note that the loop 168-175 has much fluctuation compared to that of C1.

Figure S39. Detailed analysis of the catalytic criteria for **4c** conformation 1. (**A**) The RMSD of **4c** shows conserved stability of the binding mode mainly in run 3 which shows the more conserved catalytic distances around 4 Å.

Figure S40. Detailed analysis of the catalytic criteria for **4c** conformation 2. (**A**) The RMSD of **4c** shows conserved stability of the binding mode mainly in run 3 which shows the more conserved catalytic distances around 4 Å for C6-C12, (6→12 cyclization) as shown in panel (**B**), it is lacking that distance for C1-O13, (13→1) shown in panel (**C**).

Figure S41. The hydrogen bond interaction profile over the MD simulation triplicates for **4c** conformations 1 and 2. (**A**) In **4c** conformation 1, the time-conserved hydrogen bond interaction between **4c** O7 (oxetane oxygen) and **Tyr335** is the key interaction (beside Mg^2+^-pyrophosphate) which stabilizes such a catalytic conformation which is missing in conformation 2 (**B**).

Figure S42. Representative snapshots from the MD simulation for both conformations show the conserved interaction of the oxetane ring with either **Tyr335** or **Asn246** through hydrogen bond interactions in panel (**A**) for conformation 1 which is missing on the other hand for conformation 2 in panel (**B**). It is important to mention that the main driving force for the substrate binding is the Mg^2+^-pyrophosphate electrostatic interaction.

Figure S43. Close-up view of the PenA pocket, showcasing the docking poses for **4c** catalytic path “a” carbocations **60**, **61** and **62,** and product **43**. The 3D representation reveals that the top scoring poses for **4c** and the subsequent carbocations along with product **43** exhibit favorable alignment within the active site of PenA.

Figure S44. The 2D representation of the docking poses for **4c** path “a” in PenA active site. These representations reveal the importance of amino acids residues **Ile177** and **Asn215** in the stabilization of the proposed carbocations. It is shown that all the carbocations exhibit a favorable alignment within the active site without any major shifts.

Figure S45. Close-up view of the PenA pocket, showcasing the docking poses for **4c** catalytic path “b”**.** The 3D representation reveals that the top scoring poses for **4c** and the subsequent carbocations **60**, **60a** (formed by (9→7) and (6→2) cyclization) and **63** along with product **44** exhibit favorable alignment within the active site of PenA enzyme.

Figure S46. The 2D representation of the docking poses for **4c** path “b” in PenA active site. These representations reveal the importance of amino acids residues **Phe76**, **Phe77** and **Ile177** in the stabilization of the proposed carbocations. It is shown that all the carbocations exhibit a favorable alignment within the active site without any major shifts.

Figure S47. Close-up depiction of the Omp7 (panel **A**) and PenA (panel **B**) pockets, showcasing the catalytic conformations of **5b**. The top scoring docking poses showed that two concurrent cyclization reactions (annotated as **5b2**) occur after 12→1 (annotated as **5b1**) ring closure attributed to the short catalytic distance between the involved carbon atoms (11→2) at 2.66 Å and (7→3) at 3.20 Å for Omp7 enzyme (panel **A**), and (11→2) at 2.71 Å and (7→3) at 3.33 Å for PenA enzyme (panel **B**). These favorable catalytic distances are absent from the docking poses of **4a** and **4b** which instead proceed via distinct ring-expansion pathways.

Figure S48. Close-up view of the PenA pocket, showcasing the docking poses for **5b** catalytic. The 3D representation reveals that the top scoring poses for **5b** and the subsequent carbocations **5b1** and **5b2** along with product **49** exhibit favorable alignment within the active site of the enzyme.

Figure S49. The 2D representation of the docking poses for **5b** path PenA active site. These representations reveal the importance of amino acids residues **Phe76** and **Ile177** in the stabilization of the proposed carbocations. It is shown that all the carbocations exhibit a favorable alignment within the active site without any major shifts.

Figure S50. Close-up view of the Omp7 pocket, showcasing the docking poses for **5b** catalytic. The 3D representation reveals that the top scoring poses for **5b** and the subsequent carbocations **5b1** and **5b2** along with product **49** exhibit favorable alignment within the active site of the enzyme.

Figure S51. The 2D representation of the docking poses for **5b** path Omp7 active site. These representations reveal the importance of amino acids residues **Met77** and **Ile180** in the stabilization of the proposed carbocations. It is shown that all the carbocations exhibit a favorable alignment within the active site without any major shifts.

# Abbreviations

Ac acetate

BHT butylated hydroxytoluene

CI chemical ionization

COSY correlation spectroscopy

DMAP dimethylamino pyridine

DMF dimethylformamide

DCE dichloroethane

DTT dithiothreitol

EA ethyl acetate

EI electron ionization

ESI electro spray ionization

Et ethyl

FID flame ionization detector

GC gas chromatography

H2BC heteronuclear 2-bond correlation

HMBC heteronuclear multiple-bond correlation

HRMS high resolution mass spectrometry

HSQC heteronuclear single-quantum correlation

IEB ion exchange buffer

IMAC immobilized metal affinity chromatography

IPA isopropanol

*m*CPBA *meta*-chlorperoxybenzoic acid

MPS multi-purpose sampler

Ms mesyl

MS mass spectrometry

MSD mass spectrometer detector

NBS *N*-bromosuccinimide

NMR nuclear magnetic resonance

NOESY nuclear Overhauser effect spectroscopy

NTA nitrioltriacetic acid

OD optical density

PE petroleum ether

PTFE polytetrafluorethylene

QTOF quadrupole time-of-flight

*R*_F_ retention factor

RI retention index

rpm revolutions per minute

rt room temperature

sat. saturated

TBAB tetra-*n*-butylammonium bromide

TBAF tetra-*n*-butylammonium fluoride

TBAI tetra-*n*-butylammonium iodide

TBDPS *tert*-butyldiphenyl silyl

TDA tris[2-(2-methoxyethoxy)ethyl]amine

TEAP triethylammonium phosphate

TIC total ion chromatogram

TLC thin layer chromatography

MTPA α-methoxy-α-(trifluoromethyl)phenylacetyl

o2s over two steps

THF tetrahydrofuran

# GC-MS Data

## GC-MS assays of sesquiterpene synthases used in scale-up biotransformations

Screening results were generated following the procedures described in Section 1.2 and Section 1.4.4. Enzymes BcBOT2, Omp7, PenA and Cop4 were employed for upscaling as described in 1.4.5. Also data are provided for products formed in minute amounts. They are numbered according to the enzyme employed (e.g. BcBOT-1 for the first signal of the BcBOT2-promoted biotransformation detected by GC which was not isolated). Screening results collected for sesquiterpene synthases that could not be upscaled due to inefficient product production are summarized separately in Section 5.2.

### **4a** Biotransformation GC-MS assays

Table S6. GC-MS results for the transformation of FPP derivative **4a** with four sesquiterpene synthases.

| **Biotransformation assay for 4a** | | | | | | |
| --- | --- | --- | --- | --- | --- | --- |
| Enzyme | Molecule | *t*_R_ | RI | A[%]_total_ | m/z | Elucidation/Notes |
| BcBOT2 | BcBOT2-1 | 9.624 | 1564 | 3.585 | 202 | detected |
|  | **32** | 11.148 | 1836 | 96.415 | 220 | X-ray |
| Omp7 | **34** | 9.323 | 1514 | 20.674 | 202 | NMR |
|  | **33** | 10.163 | 1651 | 73.386 | 202 | NMR |
|  | Omp7-1 | 10.601 | 1734 | 5.941 | 220 | detected |
| PenA | PenA-1 | 8.958 | 1457 | 14.370 | 202 | detected |
|  | PenA -2 | 9.566 | 1554 | 4.320 | 202 | detected |
|  | PenA -3 | 9.623 | 1564 | 25.392 | 202 | detected |
|  | PenA -4 | 10.067 | 1639 | 4.936 | 220 | detected |
|  | **33** | 10.159 | 1655 | 38.199 | 202 | MS, RI match |
|  | PenA -5 | 10.355 | 1689 | 12.783 | 220 | detected |
| Cop4 | Cop4-1 | 9.420 | 1530 | 14.605 | 202 | detected |
|  | **33** | 10.173 | 1657 | 43.589 | 202 | MS, RI match |
|  | Cop4-2 | 10.805 | 1771 | 4.294 | 202 | detected |
|  | Cop4-3 | 10.862 | 1781 | 9.853 | 220 | detected |
|  | Cop4-4 | 11.138 | 1834 | 4.342 | 220 | detected |
|  | **35** | 11.210 | 1848 | 23.318 | 220 | NMR |


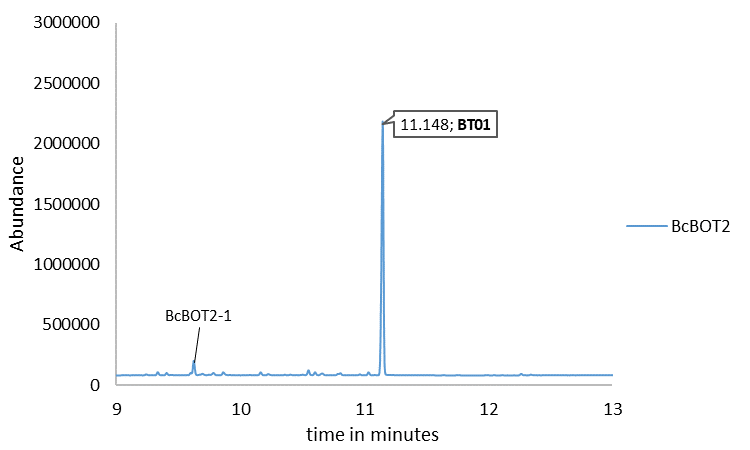


Figure S52. GC chromatogram collected after the biotransformation of FPP derivative **4a** with BcBOT2.


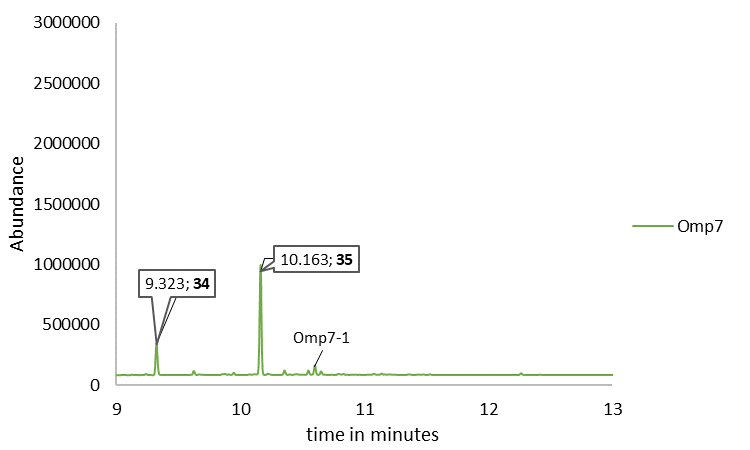


Figure S53. GC chromatogram collected after the biotransformation of FPP derivative **4a** with Omp7.


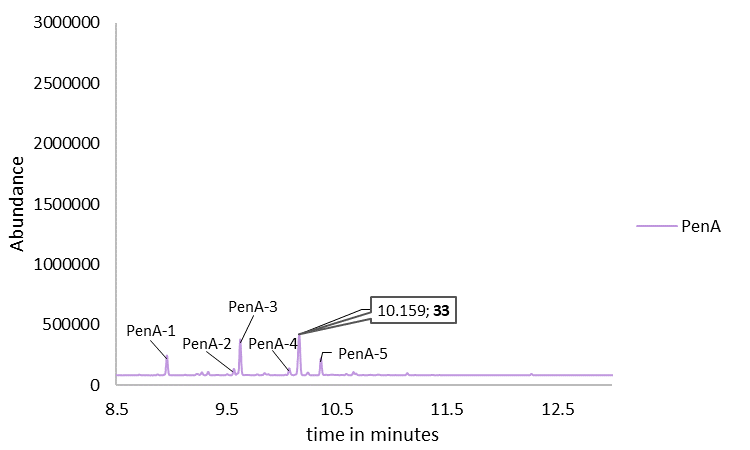


Figure S54. GC chromatogram collected after the biotransformation of FPP derivative **4a** with PenA.


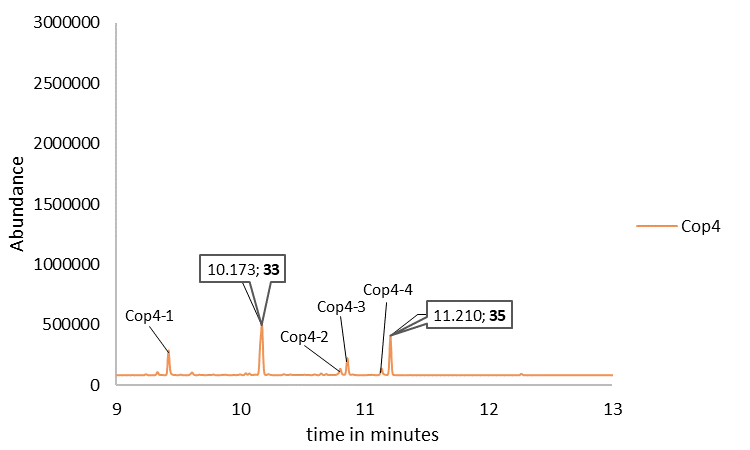


Figure S55. GC chromatogram collected after the biotransformation of FPP derivative **4a** with Cop4.

### **5a** Biotransformation GC-MS assays

Table S7. GC-MS results for the transformation of FPP derivative **5a** with four sesquiterpene synthases.

| **Biotransformation assay for 5a** | | | | | | |
| --- | --- | --- | --- | --- | --- | --- |
| Enzyme | Molecule | *t*_R_ | RI | A[%]_total_ | m/z | Elucidation/Notes |
| BcBOT2 | BcBOT2-1 | 10.198 | 1653 | 7.207 | 218 | detected |
|  | **46** | 10.316 | 1678 | 65.725 | 218 | NMR |
|  | BcBOT2-2 | 10.738 | 1759 | 4.080 | 218 | detected |
|  | BcBOT2-3 | 11.763 | 1957 | 18.138 | 236 | detected |
| Omp7 | **46** | 10.313 | 1678 | 63.240 | 218 | MS, RI match |
|  | Omp7-1 | 10.414 | 1699 | 4.633 | 218 | detected |
|  | Omp7-2 | 11.004 | 1808 | 32.127 | 218 | detected |
| PenA | **46** | 10.312 | 1677 | 65.614 | 218 | MS, RI match |
|  | PenA-1 | 10.529 | 1720 | 10.353 | 218 | detected |
|  | PenA-2 | 10.996 | 1806 | 15.324 | 218 | detected |
|  | PenA-3 | 11.338 | 1872 | 5.555 | 236 | detected |
| Cop4 |  |  |  |  |  | No/low turnover |


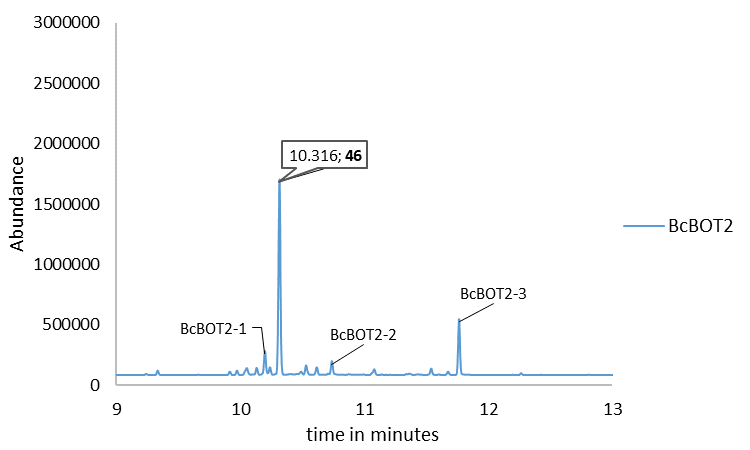


Figure S56. GC chromatogram collected after the biotransformation of FPP derivative **5a** with BcBOT2.


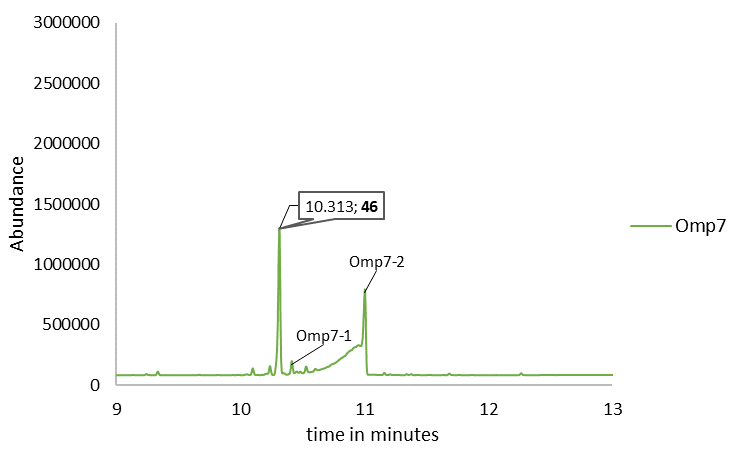


Figure S57. GC chromatogram collected after the biotransformation of FPP derivative **5a** with Omp7.


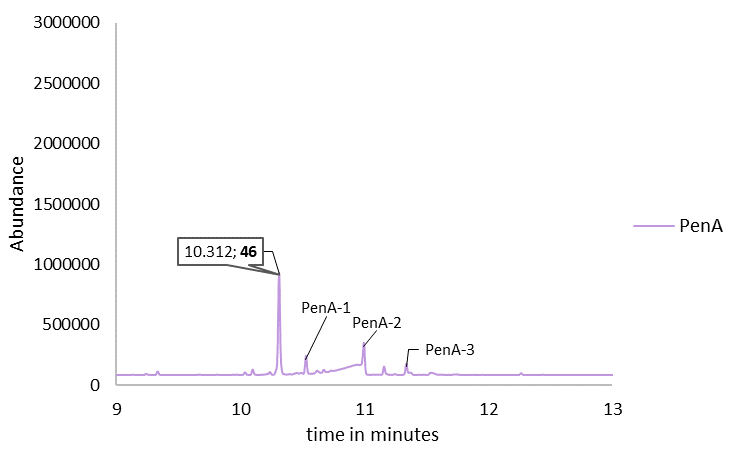


Figure S58. GC chromatogram collected after the biotransformation of FPP derivative **5a** with PenA.


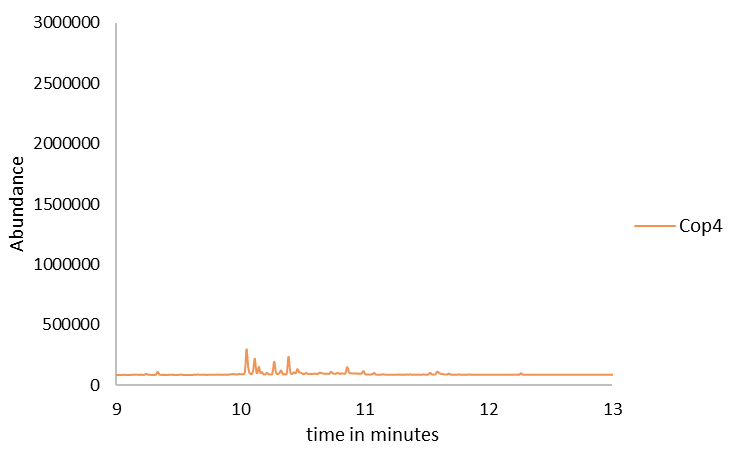


Figure S59. GC chromatogram collected after the biotransformation of FPP derivative **5a** with Cop4

### **4b** Biotransformation GC-MS assays

Table S8. GC-MS results for the transformation of FPP derivative **4b** with four sesquiterpene synthases.

| **Biotransformation assay for 4b** | | | | | | |
| --- | --- | --- | --- | --- | --- | --- |
| Enzyme | Molecule | *t*_R_ | RI | A[%]_total_ | m/z | Elucidation/Notes |
| BcBOT2 | BcBOT2-1 | 9.508 | 1625 | 3.635 | 216 | detected |
|  | BcBOT2-2 | 9.667 | 1628 | 3.902 | 216 | detected |
|  | BcBOT2-3 | 10.271 | 1736 | 5.899 | 216 | detected |
|  | **36** | 10.361 | 1753 | 15.095 | 216 | MS, RI match |
|  | BcBOT2-4 | 11.176 | 1891 | 3.562 | 216 | detected |
|  | **37** | 11.221 | 1919 | 47.863 | 234 | NMR |
|  | BcBOT2-5 | 11.319 | 1939 | 20.043 | 234 | detected |
| Omp7 | Omp7-1 | 10.184 | 1720 | 3.620 | 216 | detected |
|  | Omp7-2 | 10.254 | 1733 | 7.753 | 216 | detected |
|  | **36** | 10.368 | 1754 | 83.844 | 216 | NMR |
|  | Omp7-3 | 10.992 | 1873 | 4.783 | 234 | Only detected |
| PenA | PenA-3 | 10.698 | 1736 | 15.594 | 216 | Only detected |
|  | **36** | 10.804 | 1756 | 84.406 | 216 | MS, RI match |
| Cop4 | **38** | 10.078 | 1633 | 52.041 | 216 | NMR |
|  | Cop4-1 | 10.154 | 1643 | 5.639 | 216 | detected |
|  | Cop4-2 | 10.469 | 1698 | 9.434 | 234 | detected |
|  | Cop4-3 | 10.608 | 1732 | 19.371 | 234 | detected |
|  | **36** | 10.766 | 1753 | 13.516 | 216 | MS, RI match |


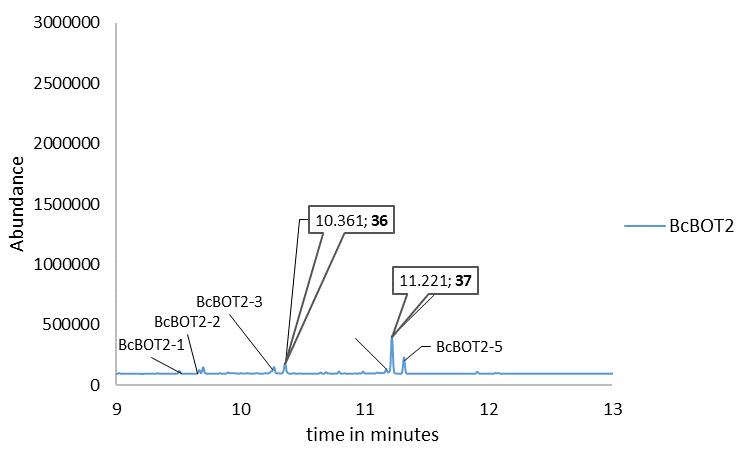


BcBOT2-4

Figure S60. GC chromatogram collected after the biotransformation of FPP derivative **4b** with BcBOT2.


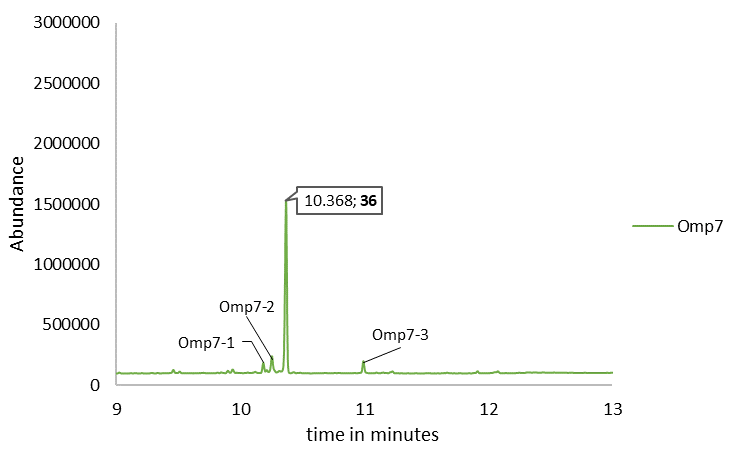


Figure S61. GC chromatogram collected after the biotransformation of FPP derivative **4b** with Omp7.


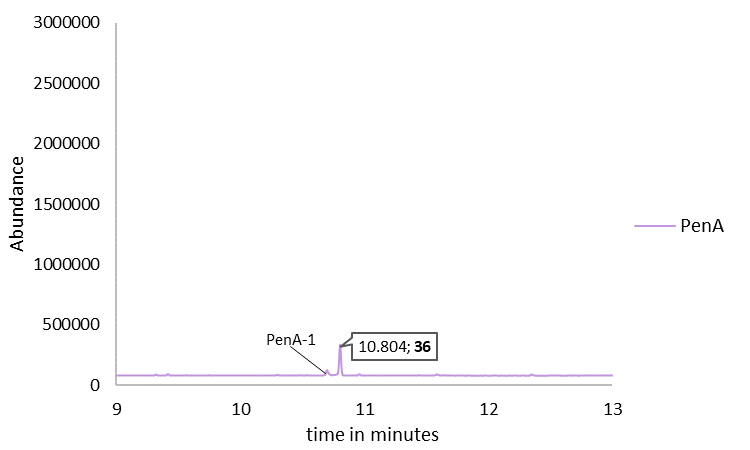


Figure S62. GC chromatogram collected after the biotransformation of FPP derivative **4b** with PenA.


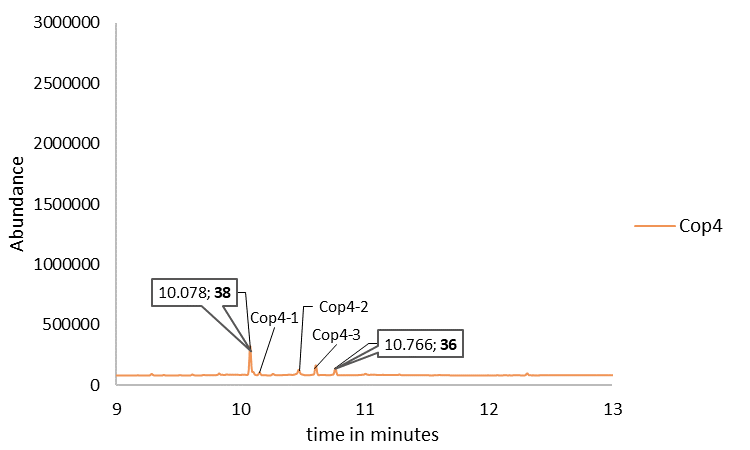


Figure S63. GC chromatogram collected after the biotransformation of FPP derivative **4b** with Cop4.

### **5b** Biotransformation GC-MS assays

Table S9. GC-MS results for the transformation of FPP derivative **5b** with four sesquiterpene synthases.

| **Biotransformation assay for 5b** | | | | | | |
| --- | --- | --- | --- | --- | --- | --- |
| Enzyme | Molecule | *t*_R_ | RI | A[%]_total_ | m/z | Elucidation/Notes |
| BcBOT2 |  |  |  |  |  | No/low turnover |
| Omp7 | **49 + 50** | 10.878 | 1782 | 53.422 | 232 | NMR |
|  | **51** | 11.505 | 1902 | 46.568 | 232 | NMR |
| PenA | PenA-1 | 10.805 | 1756 | 3.983 | 232 | detected |
|  | **49 + 50** | 10.956 | 1783 | 53.044 | 232 | MS, RI match |
|  | PenA-2 | 11.108 | 1812 | 3.22 | 232 | detected |
|  | **51** | 11.579 | 1902 | 39.753 | 232 | NMR |
| Cop4 |  |  |  |  |  | No/low turnover |


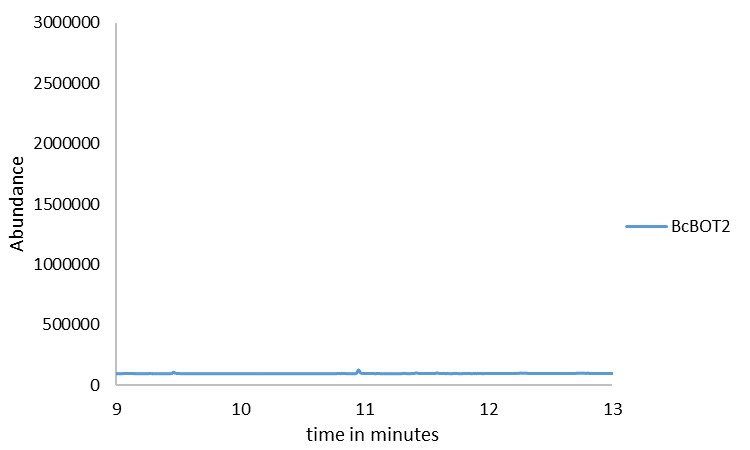


Figure S64. GC chromatogram collected after the biotransformation of FPP derivative **5b** with BcBOT2.


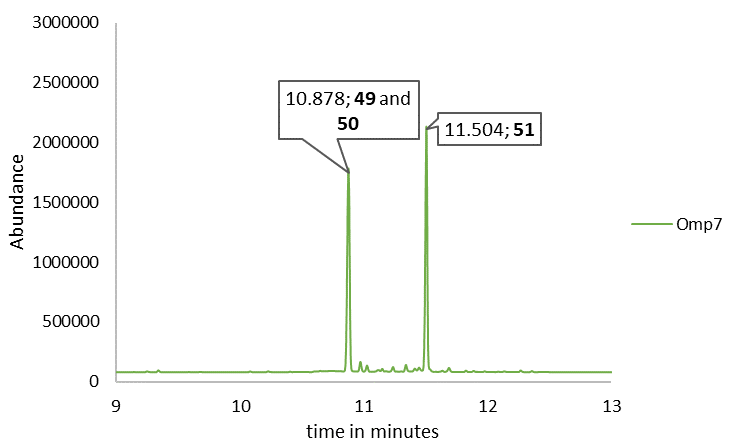


Figure S65. GC chromatogram collected after the biotransformation of FPP derivative **5b** with Omp7.


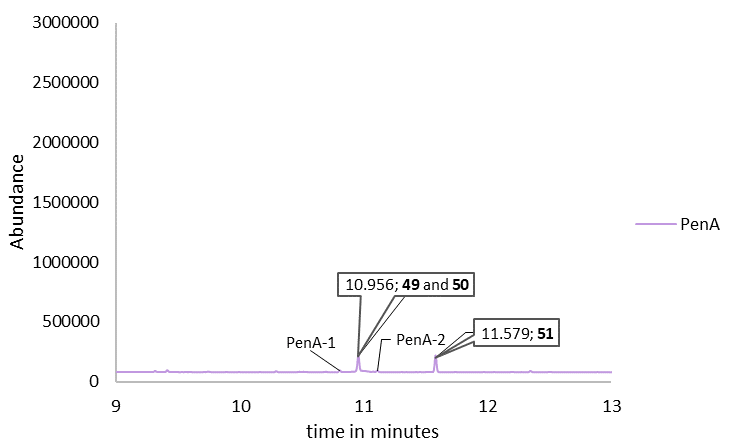


Figure S66. GC chromatogram collected after the biotransformation of FPP derivative **5b** with PenA.


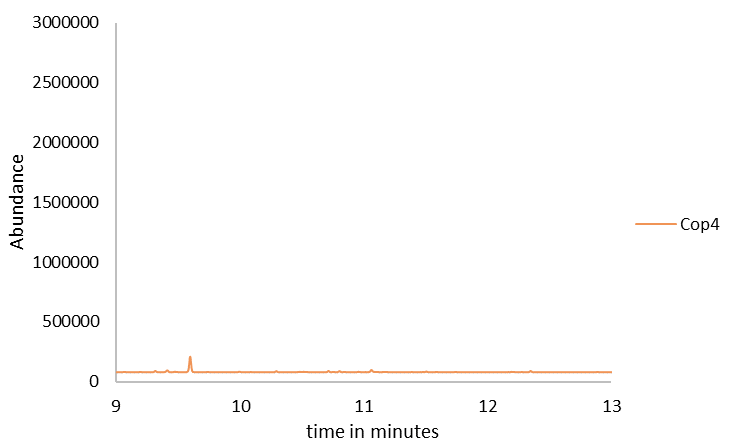


Figure S67. GC chromatogram collected after the biotransformation of FPP derivative **5b** with Cop4 (no m/z match).

### **4c** Biotransformation GC-MS assays

Table S10. GC-MS results for the transformation of FPP derivative **4c** with four sesquiterpene synthases.

| **Biotransformation assay for 4c** | | | | | | |
| --- | --- | --- | --- | --- | --- | --- |
| Enzyme | Molecule | *t*_R_ | RI | A[%]_total_ | m/z | Elucidation/Notes |
| BcBOT2 | BcBOT2-1 | 10.313 | 1640 | 3.079 | 218 | detected |
|  | BcBOT2-2 | 10.889 | 1743 | 6.383 | 218 | detected |
|  | BcBOT2-3 | 11.076 | 1777 | 22.937 | 218 | detected |
|  | BcBOT2-4 | 11.283 | 1816 | 3.126 | 218 | detected |
|  | **41** and **42** | 11.525 | 1863 | 42.214 | 218 | NMR |
|  | **39** | 12.152 | 1989 | 20.018 | 236 | NMR |
|  | **40** | 12.474 | 2052 | 2.243 | 236 | NMR |
| Omp7 |  |  |  |  |  | No/low turnover |
| PenA | **43** | 10.571 | 1685 | 68.470 | 218 | NMR |
|  | **44** | 10.693 | 1706 | 10.194 | 218 | NMR |
|  | PenA-1 | 10.723 | 1712 | 5.107 | 218 | detected |
|  | PenA-2 | 10.910 | 1747 | 10.164 | 218 | Only detected |
|  | PenA-3 | 11.226 | 1805 | 4.273 | 218 | Only detected |
| Cop4 |  |  |  |  |  | No/low turnover |


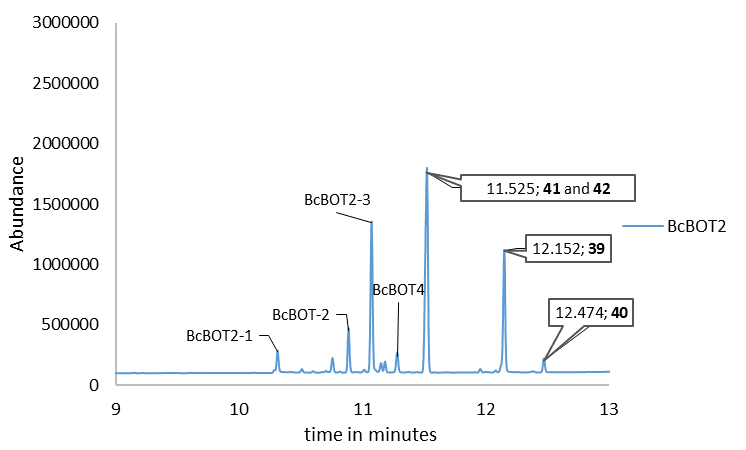


Figure S68. GC chromatogram collected after the biotransformation of FPP derivative **4c** with BcBOT2.


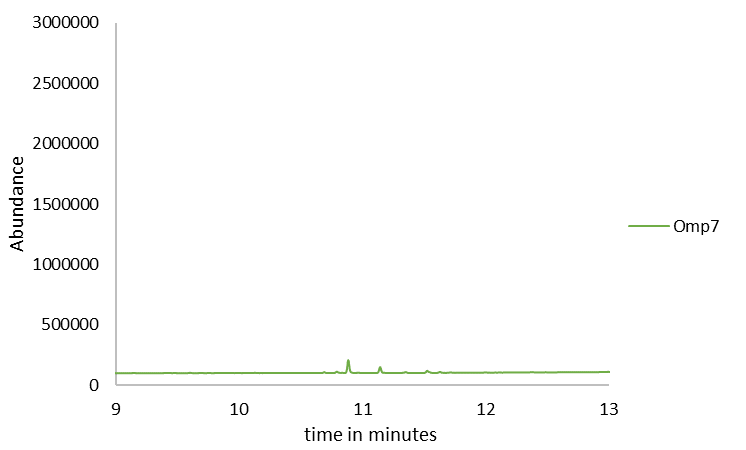


Figure S69. GC chromatogram collected after the biotransformation of FPP derivative **4c** with Omp7.


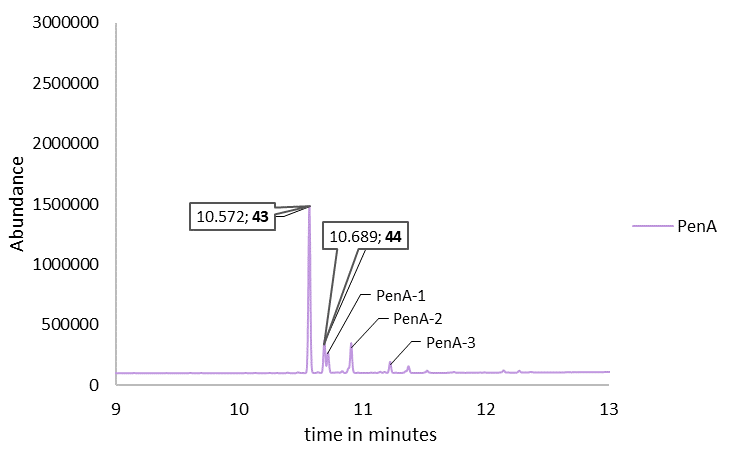


Figure S70. GC chromatogram collected after the biotransformation of FPP derivative **4c** with PenA.


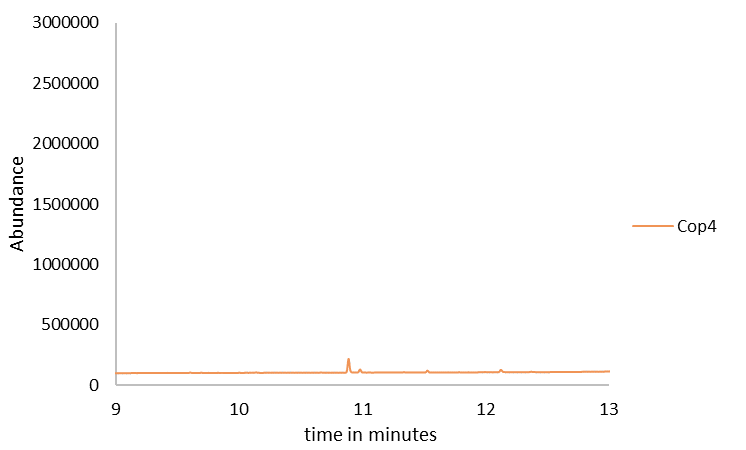


Figure S71. GC chromatogram collected after the biotransformation of FPP derivative **4c** with Cop4 (no m/z match).

### **5c** Biotransformation GC-MS assays

GC chromatograms for the sesquiterpene synthases BcBOT2, Omp7, PenA and Cop4 neither show the expected m/z values for an elimination nor for a hydrolysis product (m/z 234 and 252) when FPP derivative **5c** served as substrate.


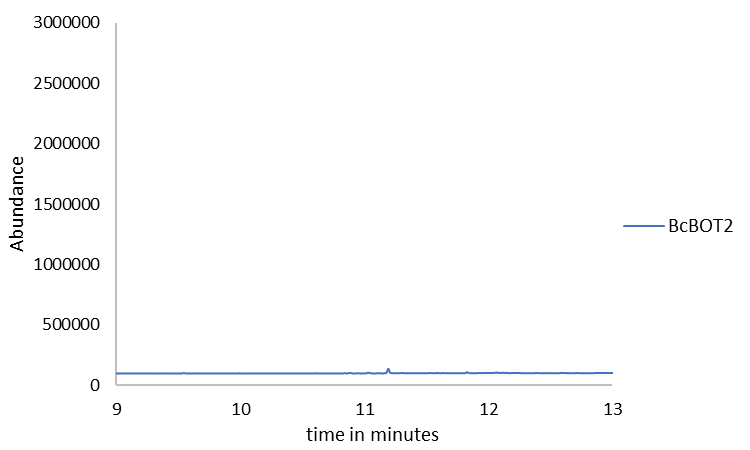


Figure S72. GC chromatogram collected after the biotransformation of FPP derivative **5c** with BcBOT2.


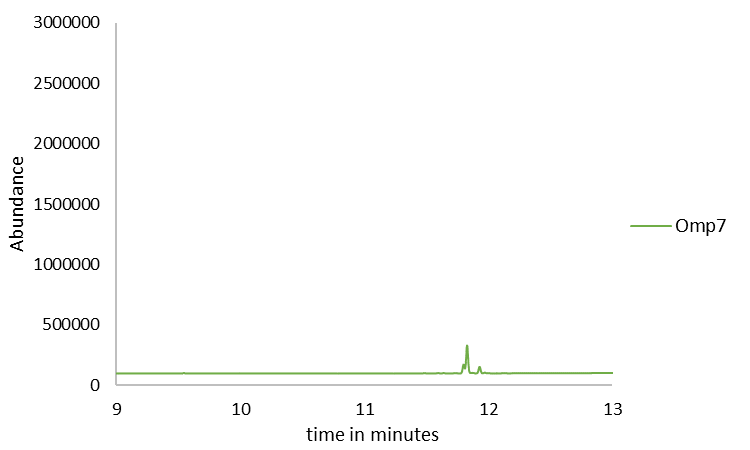


Figure S73. GC chromatogram collected after the biotransformation of FPP derivative **5c** with Omp7.


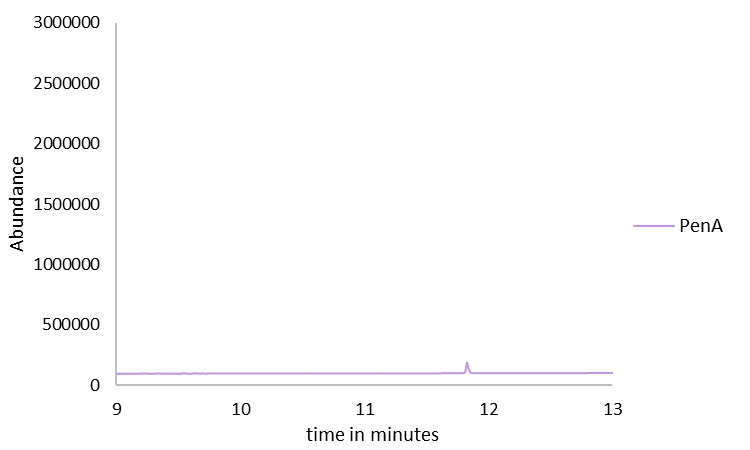


Figure S74. GC chromatogram of the biotransformation reaction of FPP derivative **5c** with PenA.


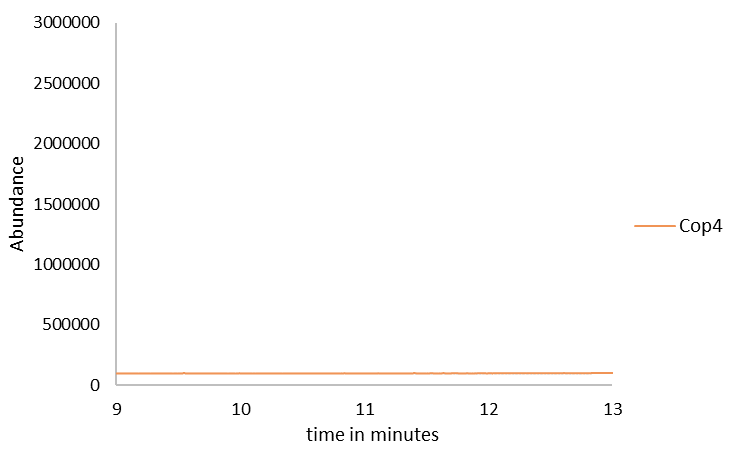


Figure S75. GC chromatogram of the biotransformation reaction of FPP derivative **5c** with Cop4.

## GC-MS assays collected from biotransformations not further upscaled

### **4a** Biotransformations with Hvs1, Tri5, Cyc1, Tps32 and GcoA


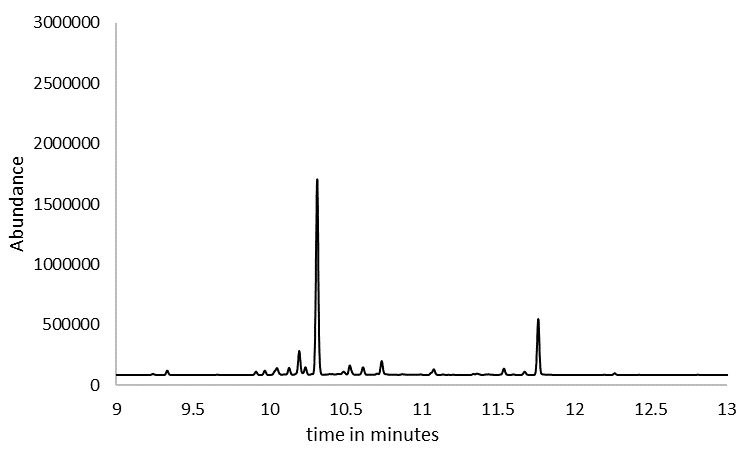


Figure S76. GC chromatogram collected after the biotransformation of FPP derivative **4a** with Hvs1.


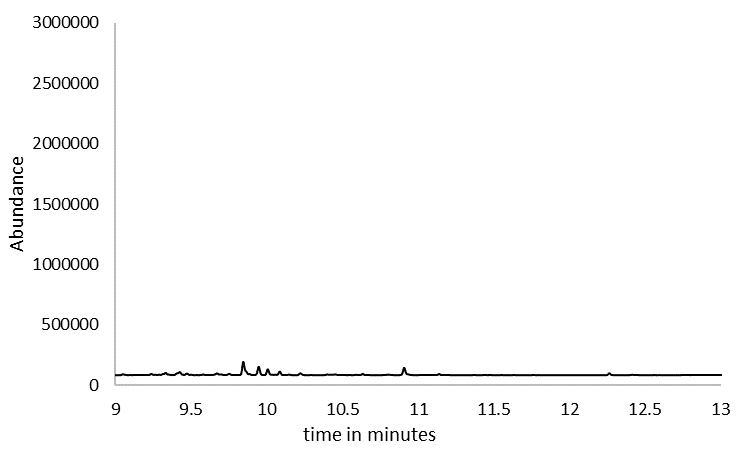


Figure S77. GC chromatogram collected after the biotransformation of FPP derivative **4a** with Tri5.


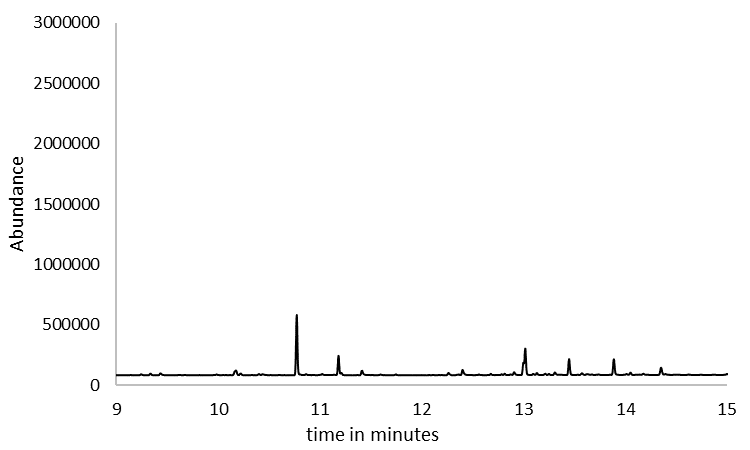


Figure S78. GC chromatogram collected after the biotransformation of FPP derivative **4a** with Cyc1.


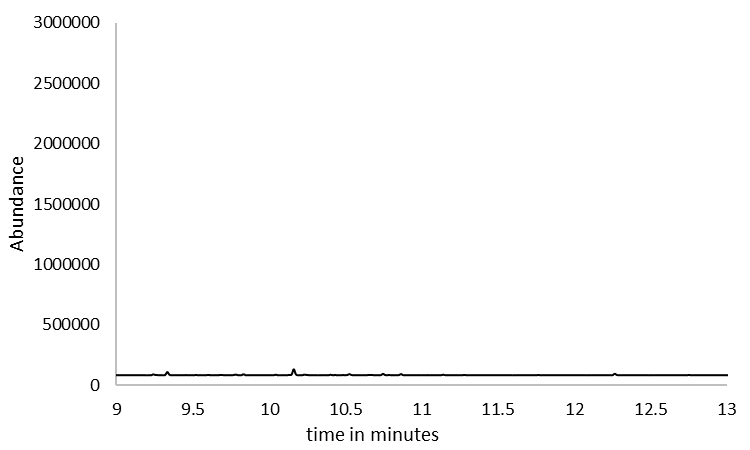


Figure S79. GC chromatogram collected after the biotransformation of FPP derivative **4a** with Tps32.

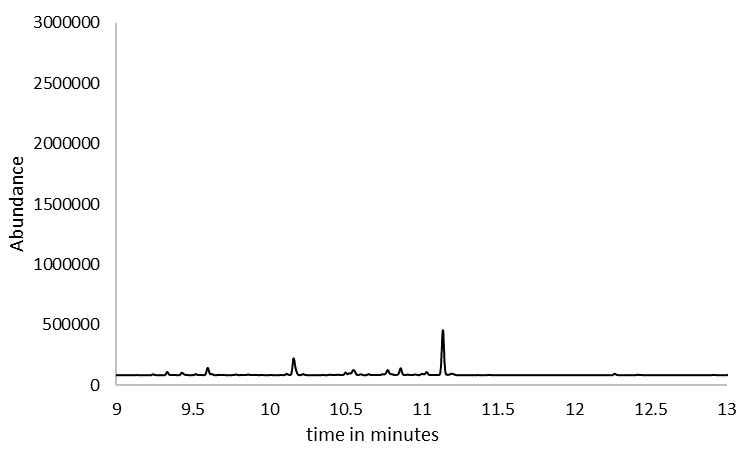


Figure S80. GC chromatogram collected after the biotransformation of FPP derivative **4a** with GcoA.

### **5a** Biotransformations with Hvs1, Tri5, Cyc1, Tps32 and GcoA


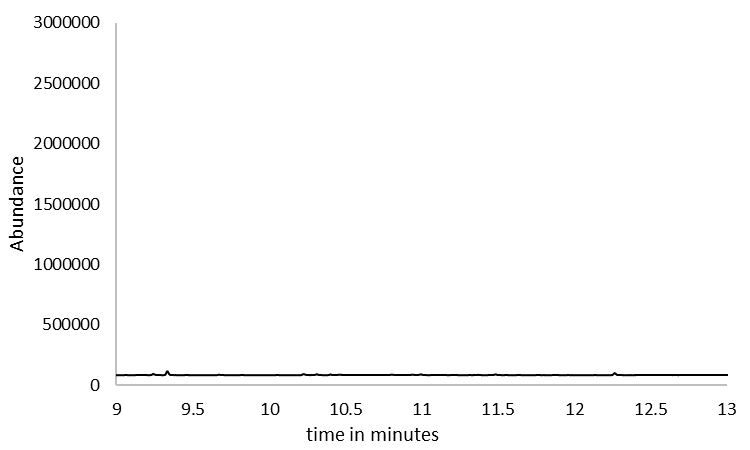


Figure S81. GC chromatogram collected after the biotransformation of FPP derivative **5a** with Hvs1.


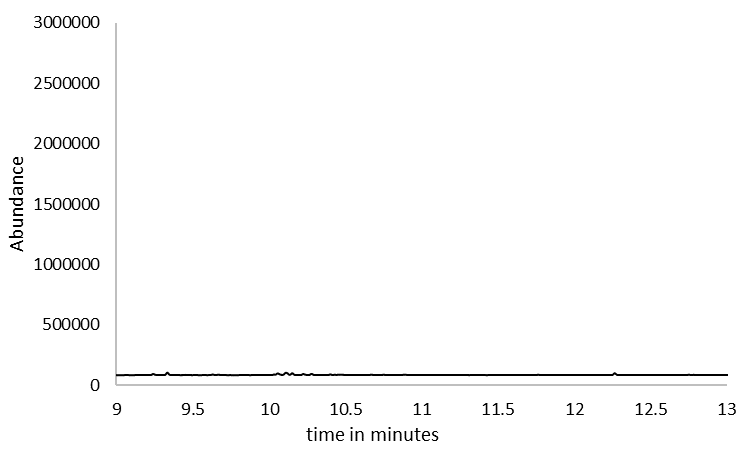


Figure S82. GC chromatogram collected after the biotransformation of FPP derivative **5a** with Tri5.


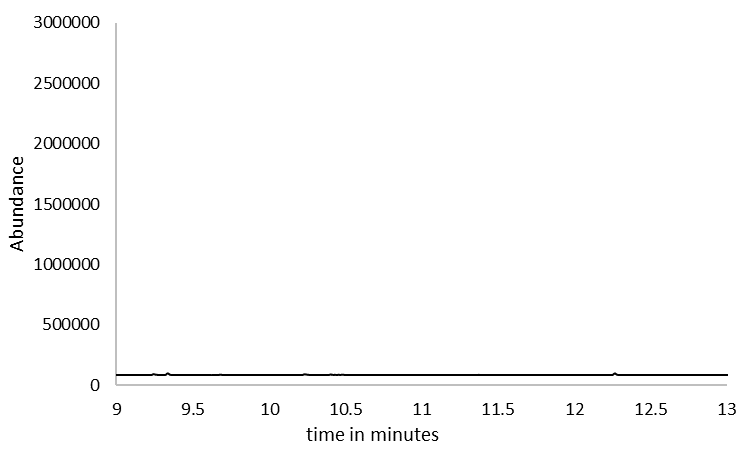


Figure S83. GC chromatogram collected after the biotransformation of FPP derivative **5a** with Cyc1.


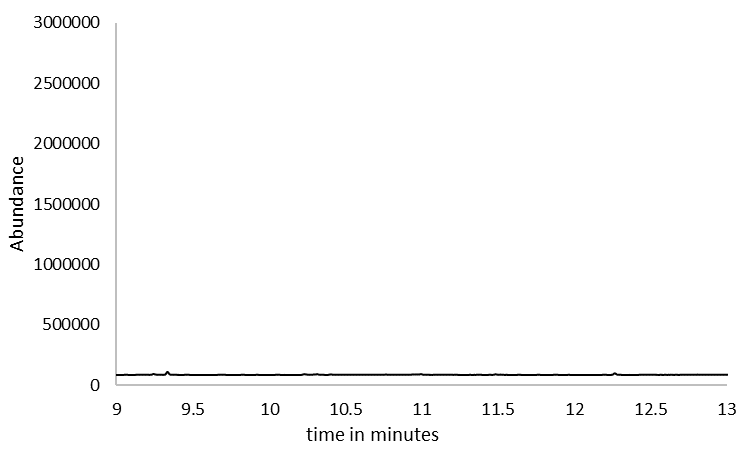


Figure S84. GC chromatogram collected after the biotransformation of FPP derivative **5a** with Tps32.


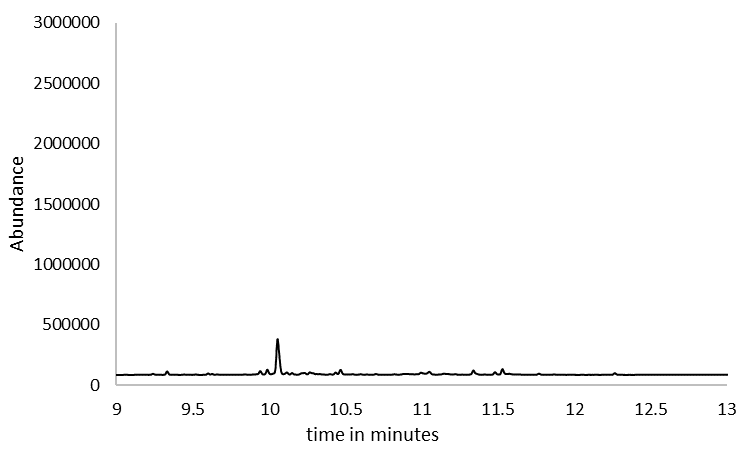


Figure S85. GC chromatogram of the biotransformation reaction of FPP derivative **5a** with GcoA.

### **4b** Biotransformations with Hvs1, Tri5, Cyc1, Tps32 and GcoA


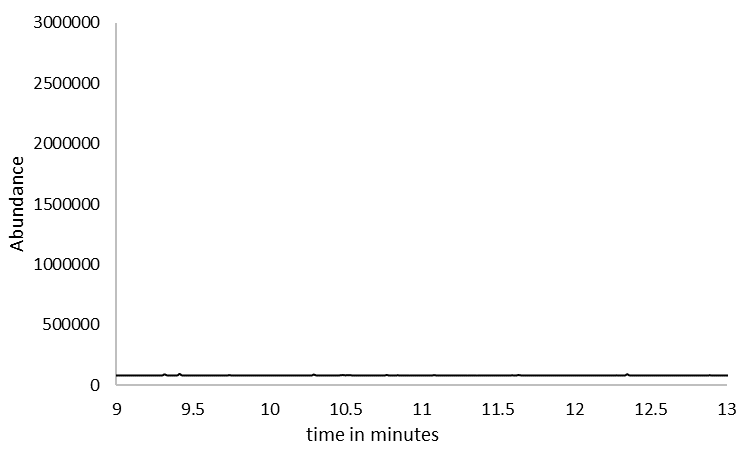


Figure S86. GC chromatogram collected after the biotransformation of FPP derivative **4b** with Hvs1.


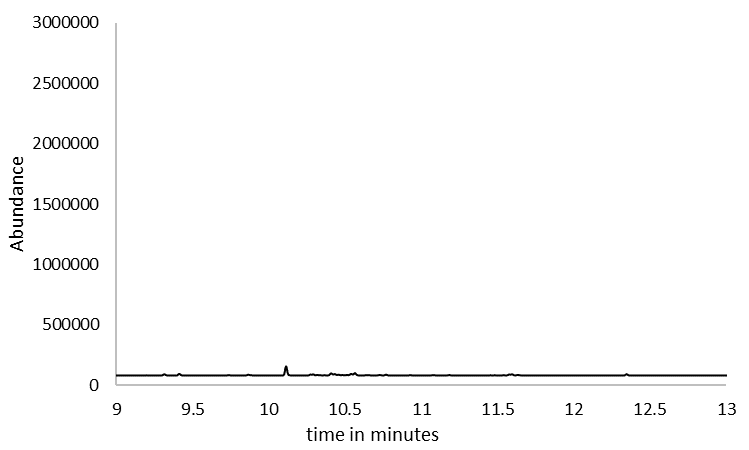


Figure S87. GC chromatogram collected after the biotransformation of FPP derivative **4b** with Tri5.


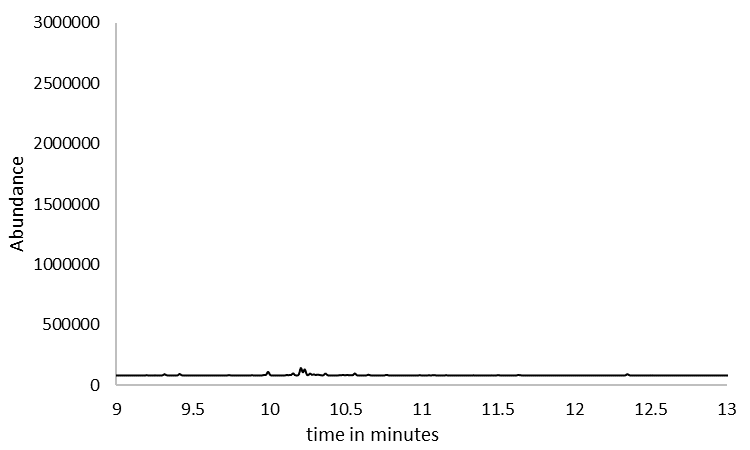


Figure S88. GC chromatogram of the biotransformation reaction of FPP derivative **4b** with Cyc1.


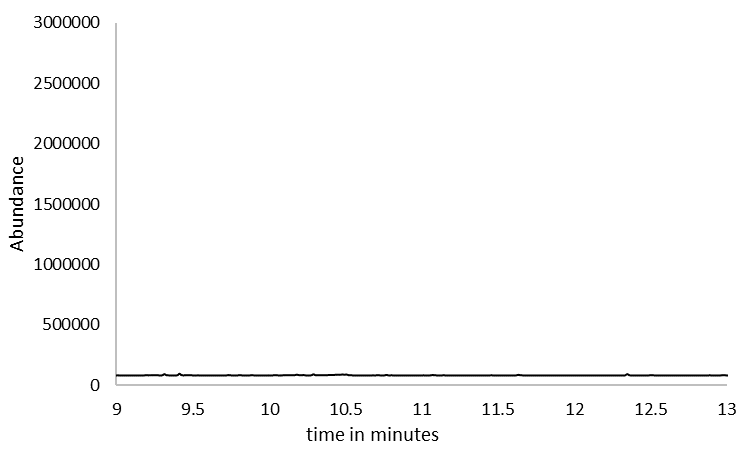


Figure S89. GC chromatogram of the biotransformation reaction of FPP derivative **4b** with Tps32.


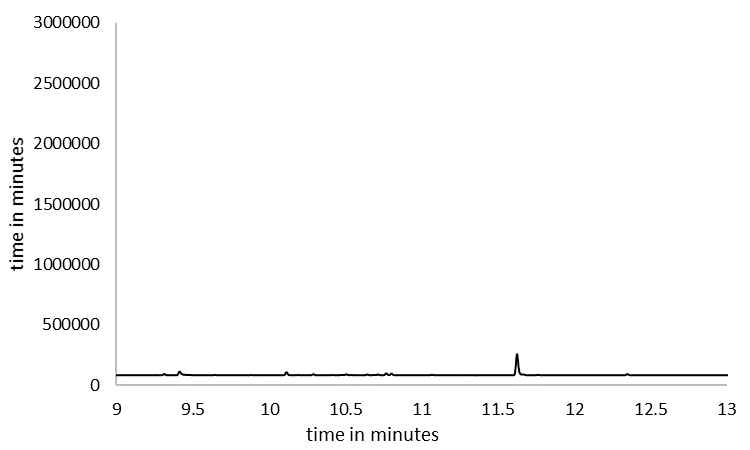


Figure S90. GC chromatogram of the biotransformation reaction of FPP derivative **4b** with GcoA.

### **5b** Biotransformations with Hvs1, Tri5, Cyc1, Tps32 and GcoA


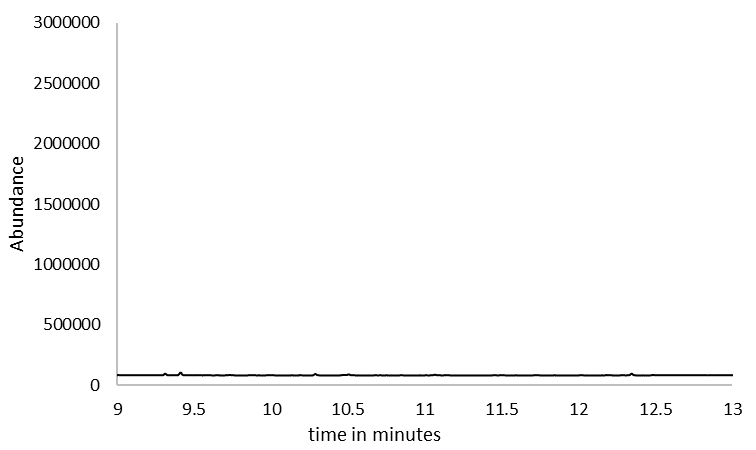


Figure S91. GC chromatogram collected after the biotransformation of FPP derivative **5b** with Hvs1.


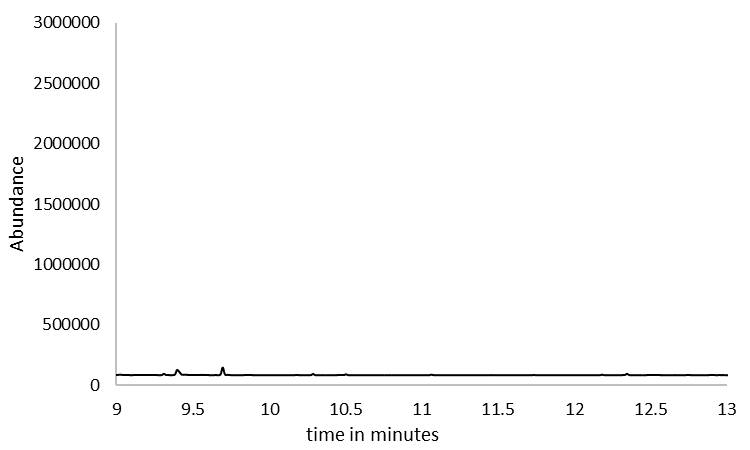


Figure S92. GC chromatogram collected after the biotransformation of FPP derivative **5b** with Tri5.


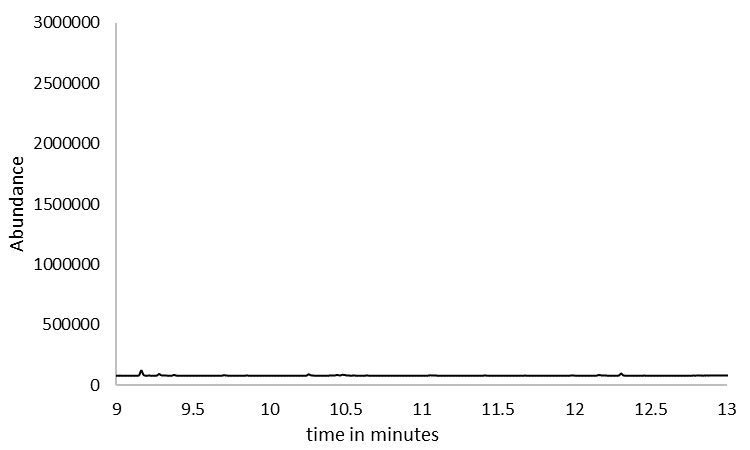


Figure S93. GC chromatogram collected after the biotransformation of FPP derivative **5b** with Cyc1.


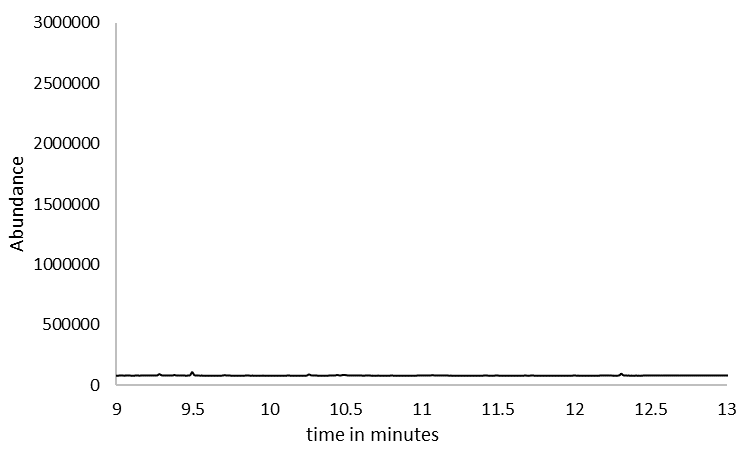


Figure S94. GC chromatogram of the biotransformation reaction of FPP derivative **5b** with Tps32.


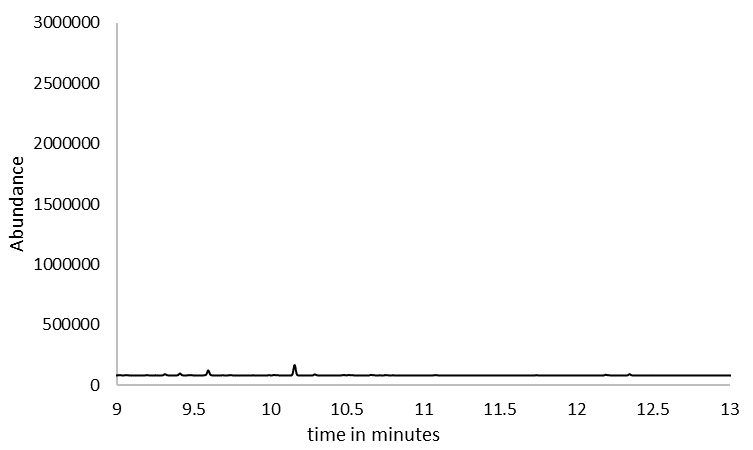


Figure S95. GC chromatogram collected after the biotransformation of FPP derivative **5b** with GcoA.

### **4c** Biotransformations with Hvs1, Tri5, Cyc1, Tps32 and GcoA


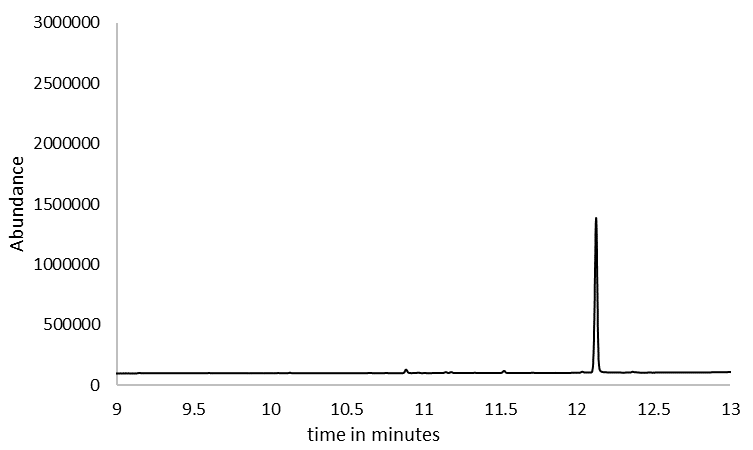


Figure S96. GC chromatogram collected after the biotransformation of FPP derivative **4c** with Hvs1.


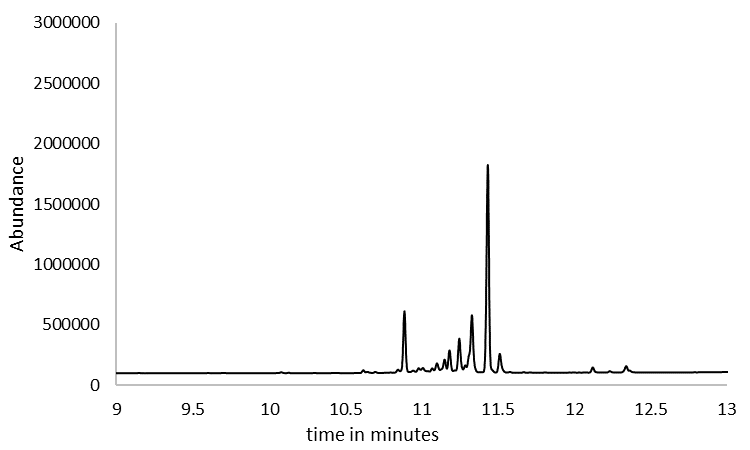


Figure S97. GC chromatogram of the biotransformation reaction of FPP derivative **4c** with Tri5.


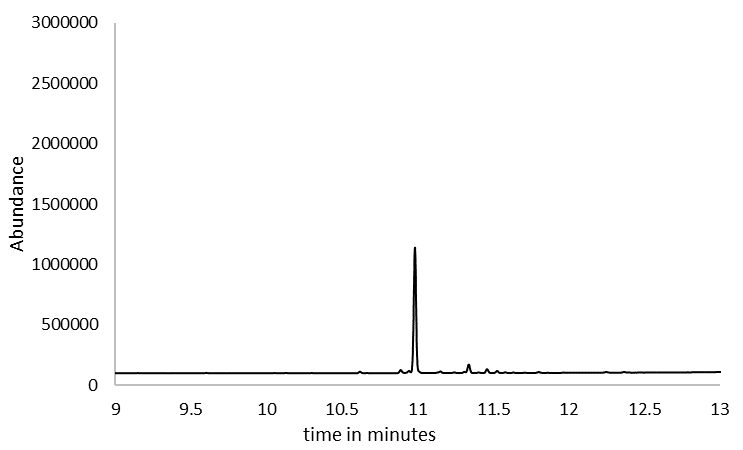


Figure S98. GC chromatogram collected after the biotransformation of FPP derivative **4c** with Cyc1.


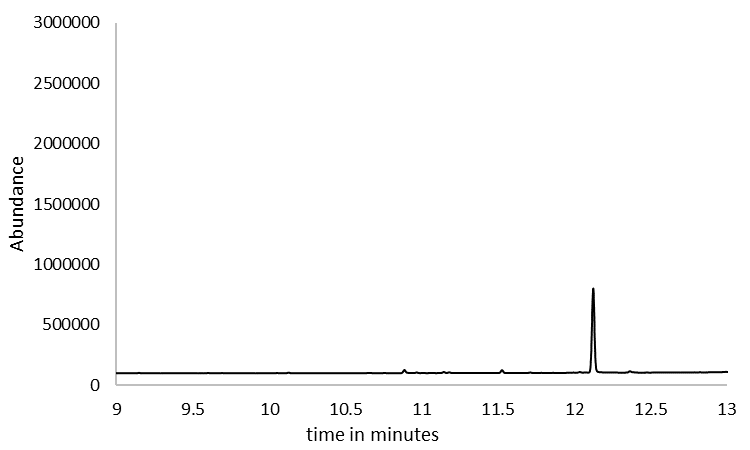


Figure S99. GC chromatogram collected after the biotransformation of FPP derivative **4c** with Tps32.


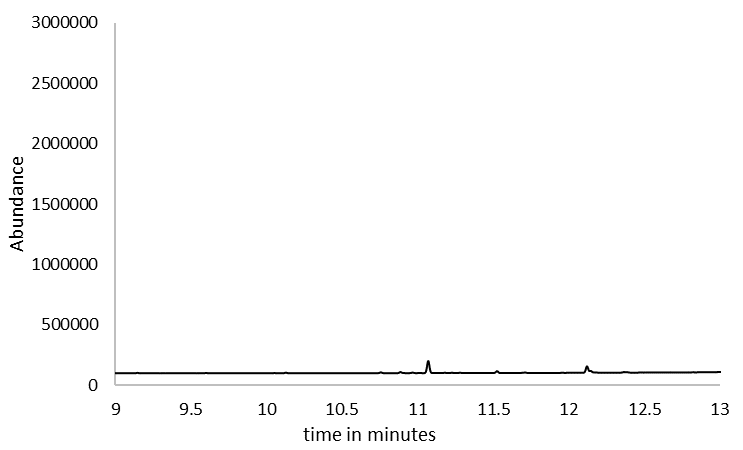


Figure S100. GC chromatogram collected after the biotransformation of FPP derivative **4c** with GcoA.

### **5c** Biotransformations with Hvs1, Tri5, Cyc1, Tps32 and GcoA


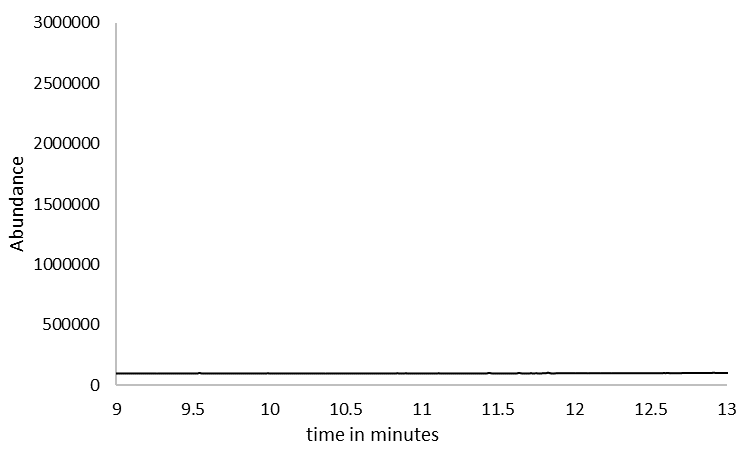


Figure S101. GC chromatogram collected after the biotransformation of FPP derivative **5c** with Hvs1.


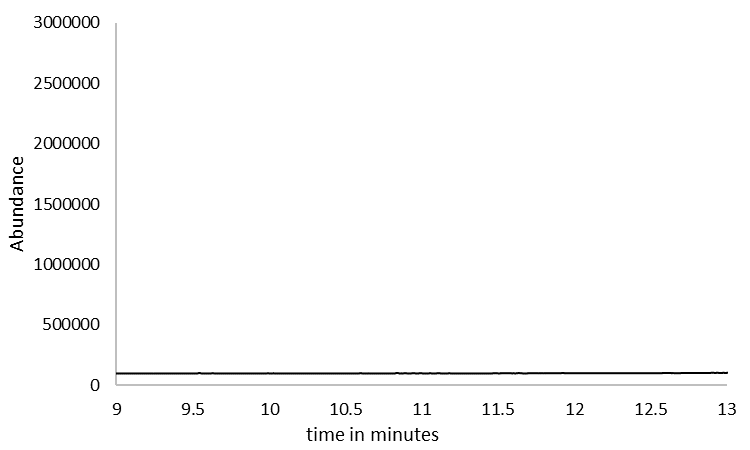


Figure S102. GC chromatogram collected after the biotransformation of FPP derivative **5c** with Tri5.


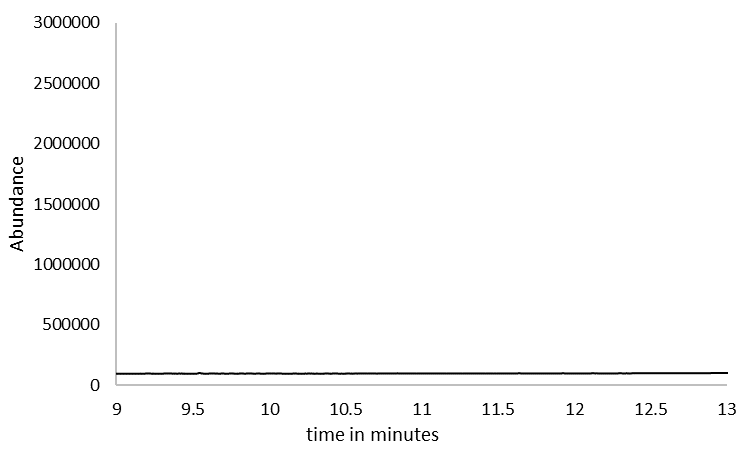


Figure S103. GC chromatogram collected after the biotransformation of FPP derivative **5c** with Cyc1.


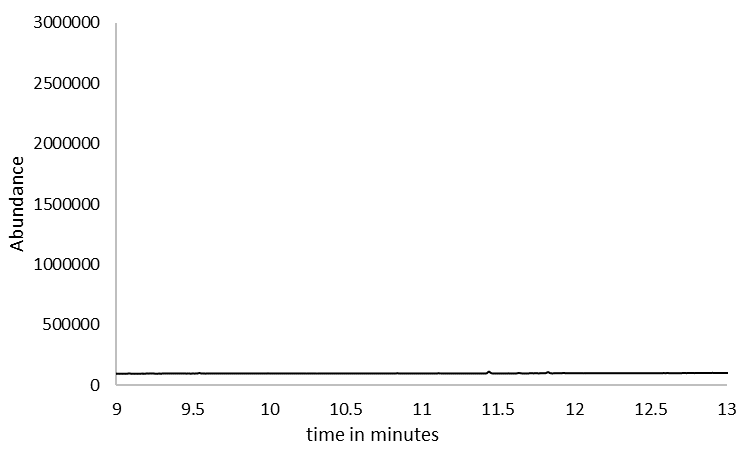


Figure S104. GC chromatogram collected after the biotransformation of FPP derivative **5c** with Tps32.


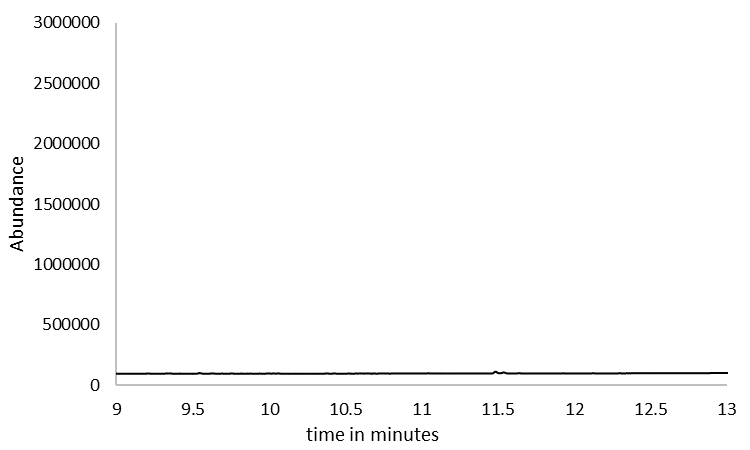


Figure S105. GC chromatogram collected after the biotransformation of FPP derivative **5c** with GcoA.

## GC chromatograms and mass spectra of new terpenoids isolated

#### Terpenoid 32; RI = 1836; m/z 220


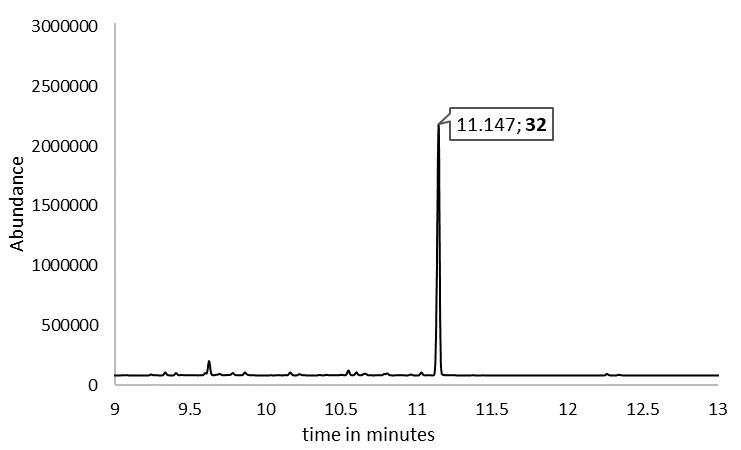


Figure S106. GC chromatogram of compound **32**.


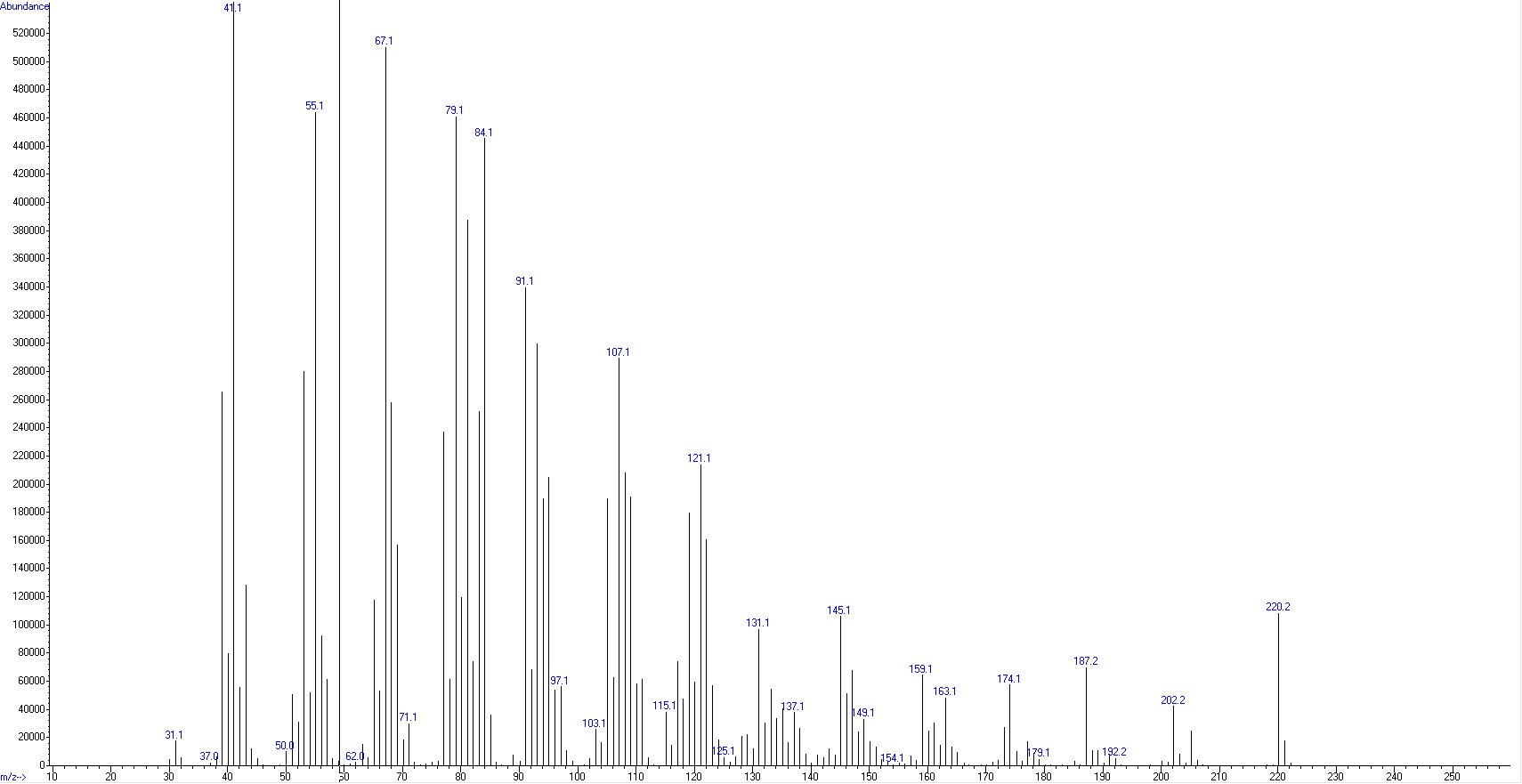


Figure S107. Mass spectrum of compound **32** (EI-MS).

#### Terpenoid 33; RI = 1651; m/z 202


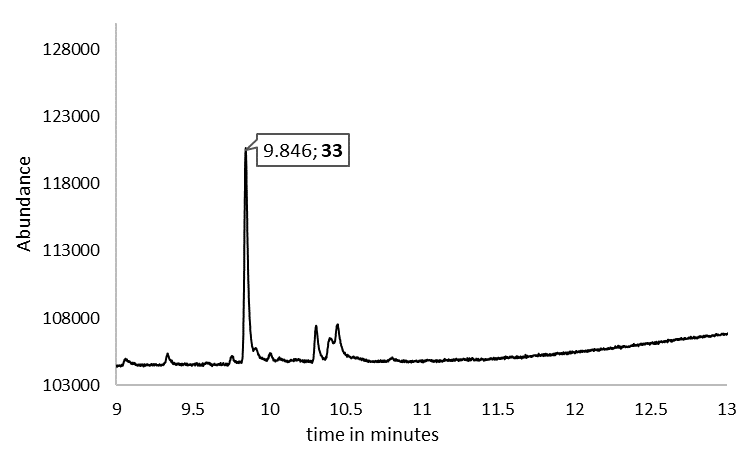


Figure S108. GC chromatogram of compound **33**.


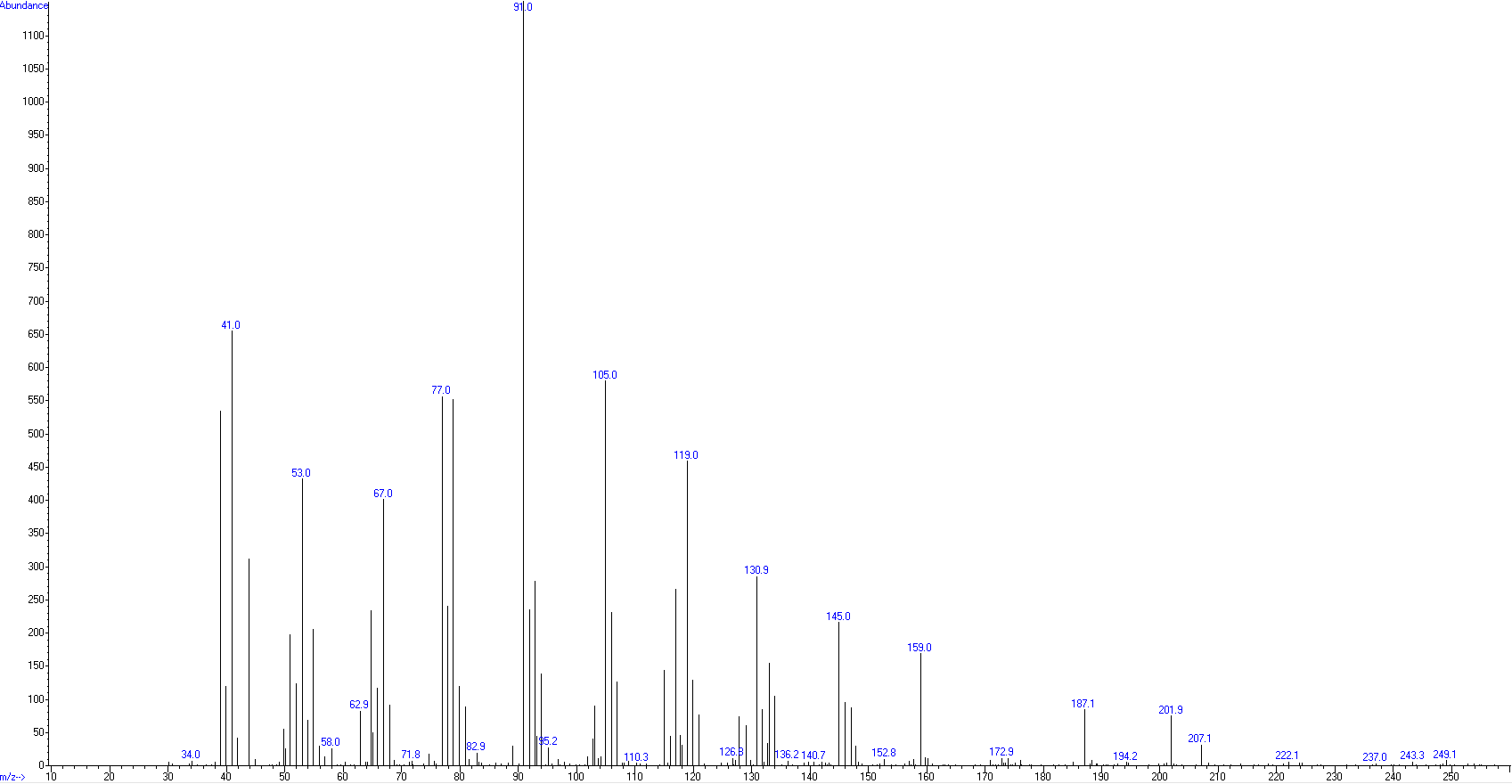


Figure S109. Mass spectrum of compound **33** (EI-MS).

#### Terpenoid 34; RI = 1514; m/z 202


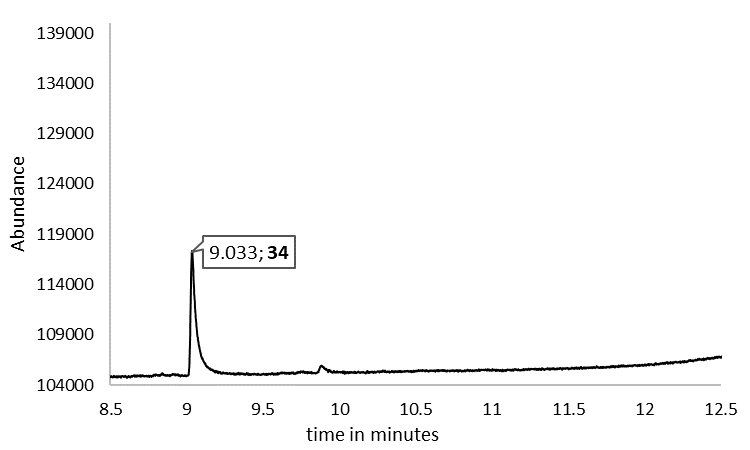


Figure S110. GC chromatogram of compound **34**.


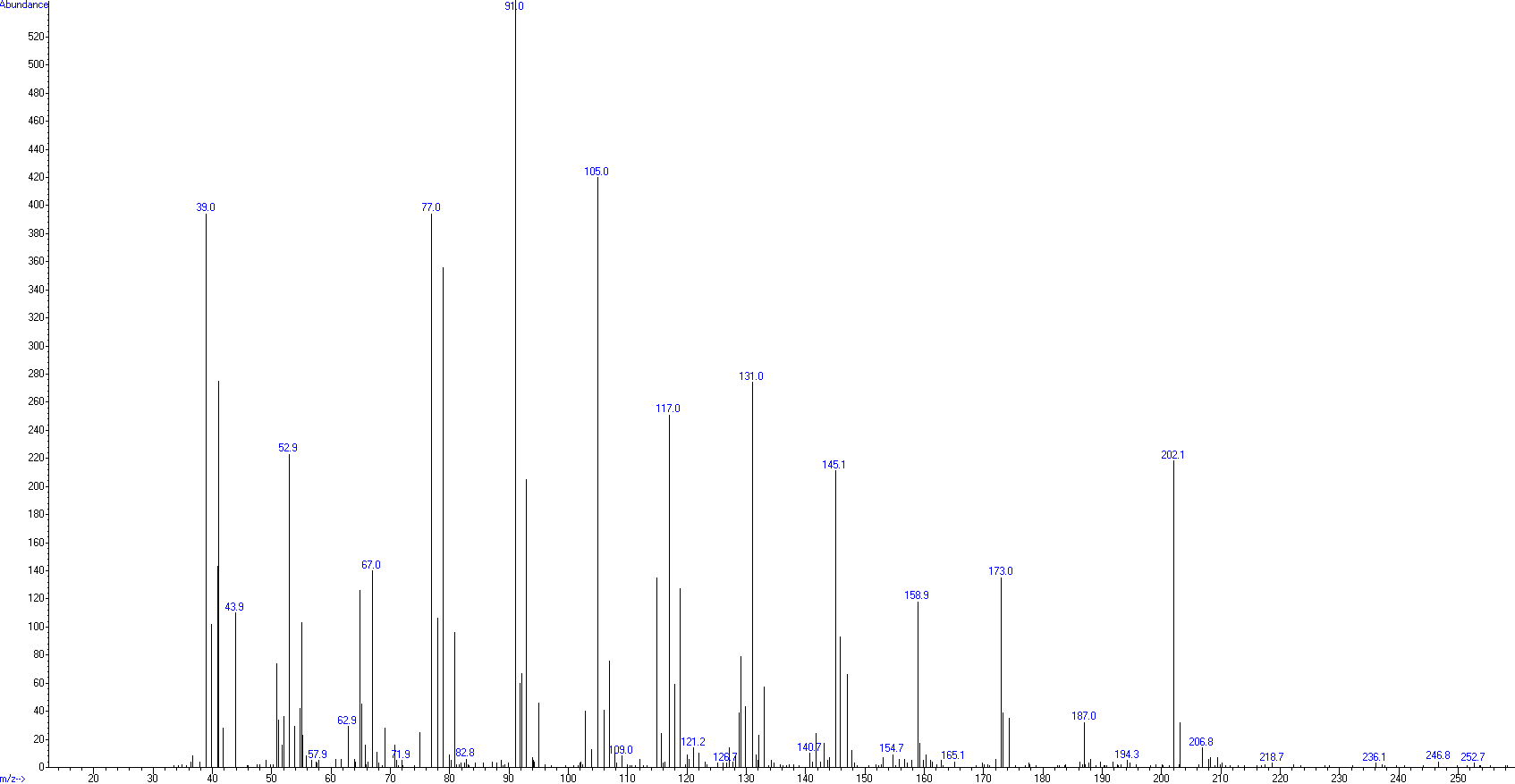


Figure S111. Mass spectrum of compound **34** (EI-MS).

#### Terpenoid 35; RI = 1848; m/z 220


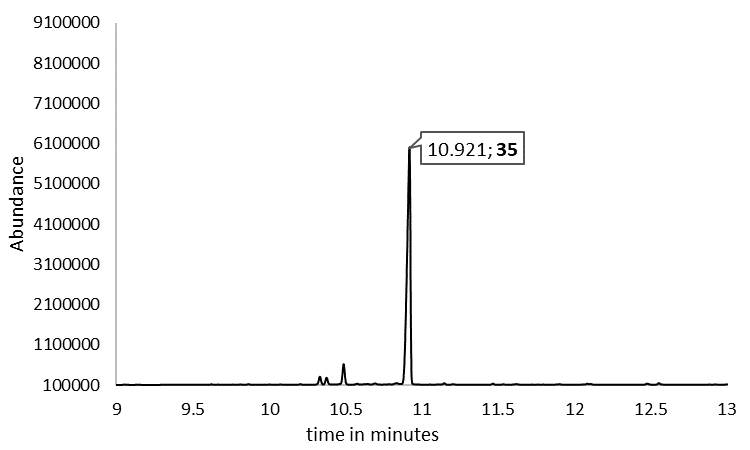


Figure S112. GC chromatogram of compound **35**.


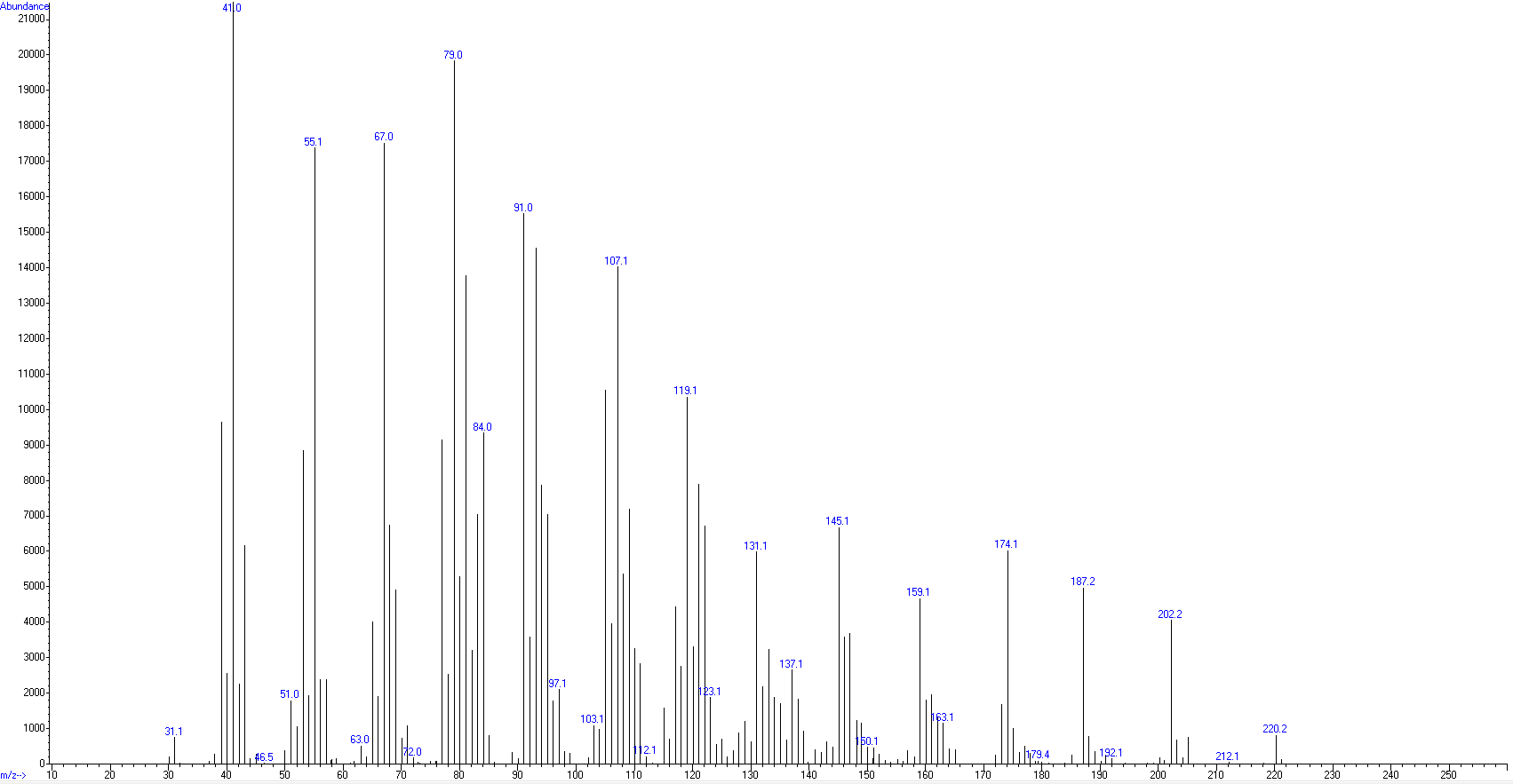


Figure S113. Mass spectrum of compound **35** (EI-MS).

#### Terpenoid 46; RI = 1678; m/z 218


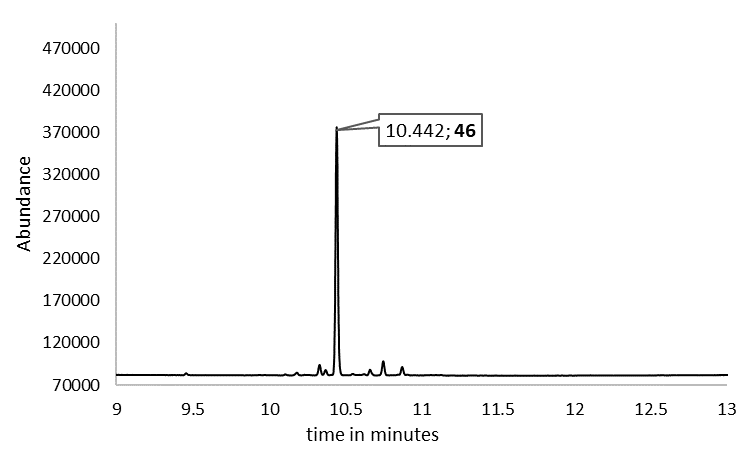


Figure S114. GC chromatogram of compound **46**.


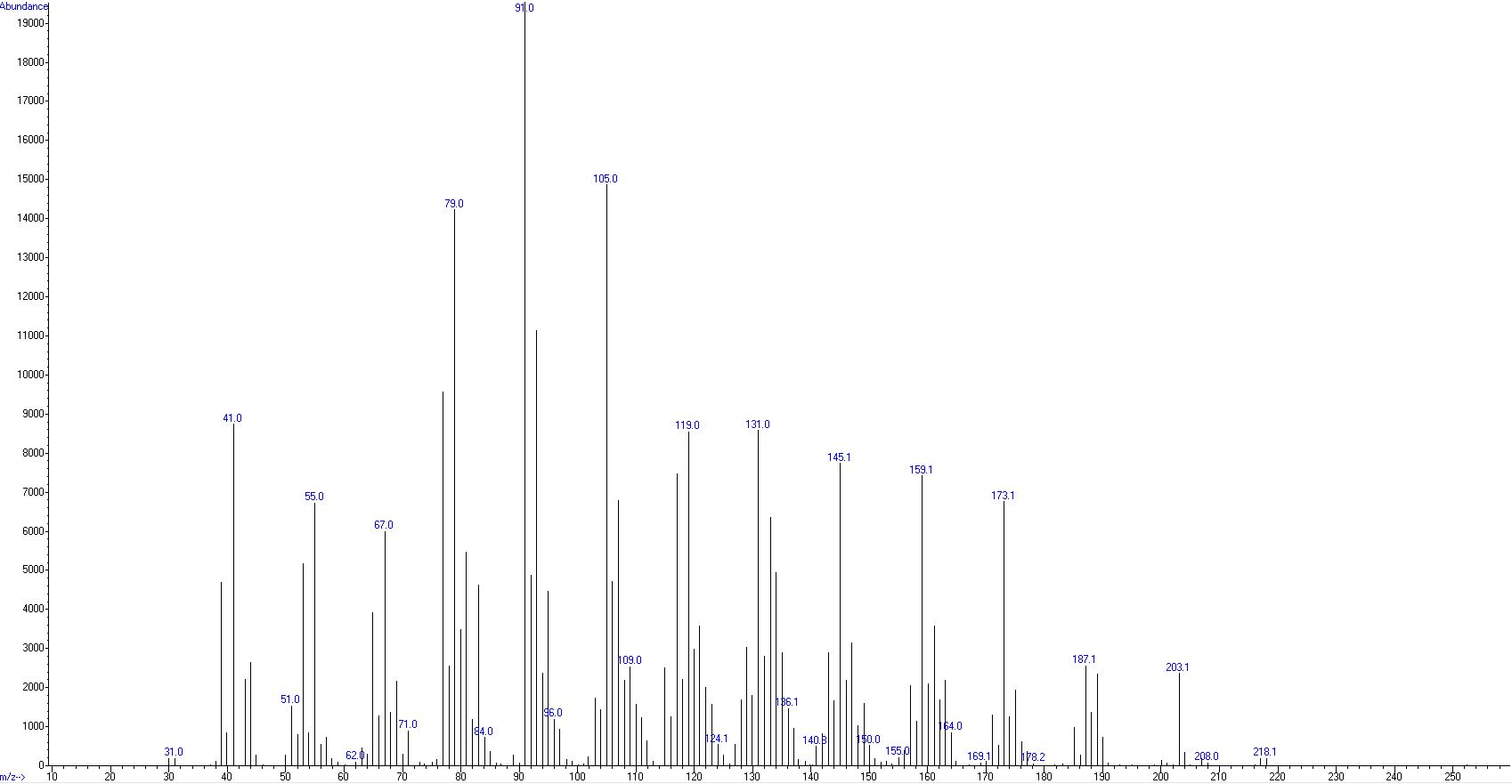


Figure S115. Mass spectrum of compound **46** (EI-MS).

#### Terpenoid 36; RI = 1754; m/z 216


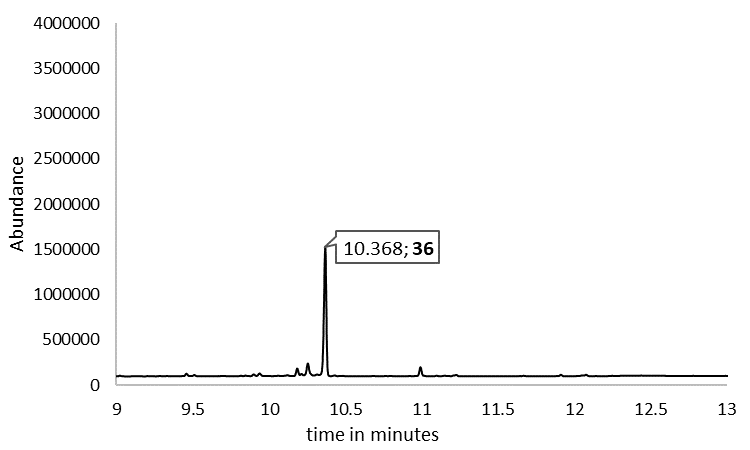


Figure S116. GC chromatogram of compound **36**.


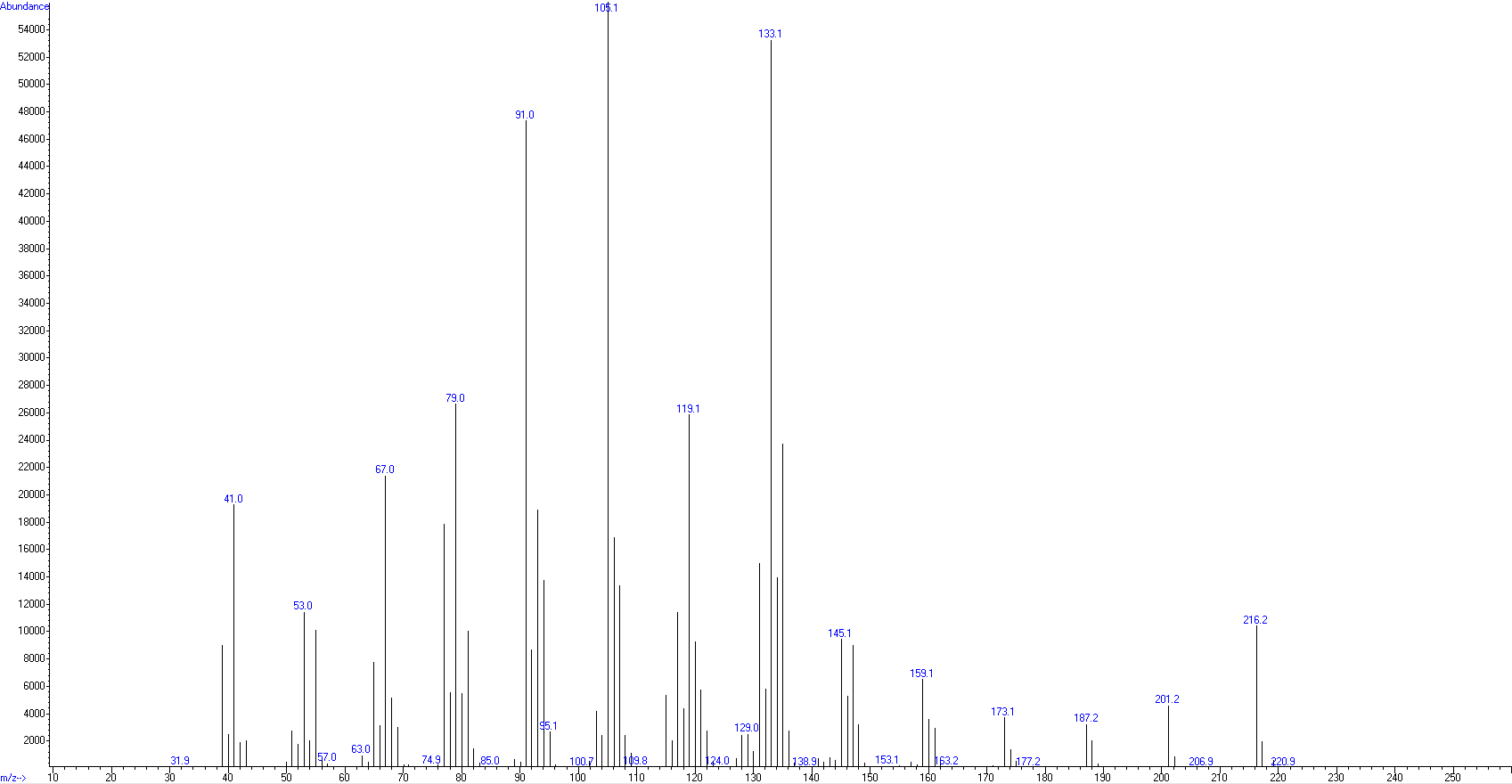


Figure S117. Mass spectrum of compound **36** (EI-MS).

#### Terpenoid 37; RI = 1919; m/z 234


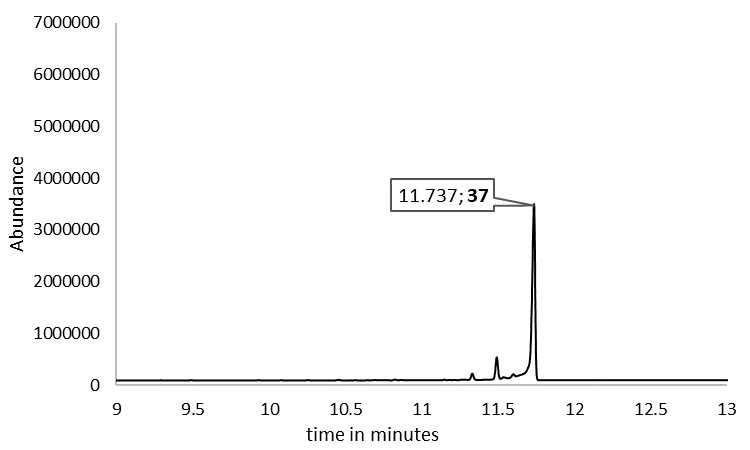


Figure S118. GC chromatogram of compound **37**.


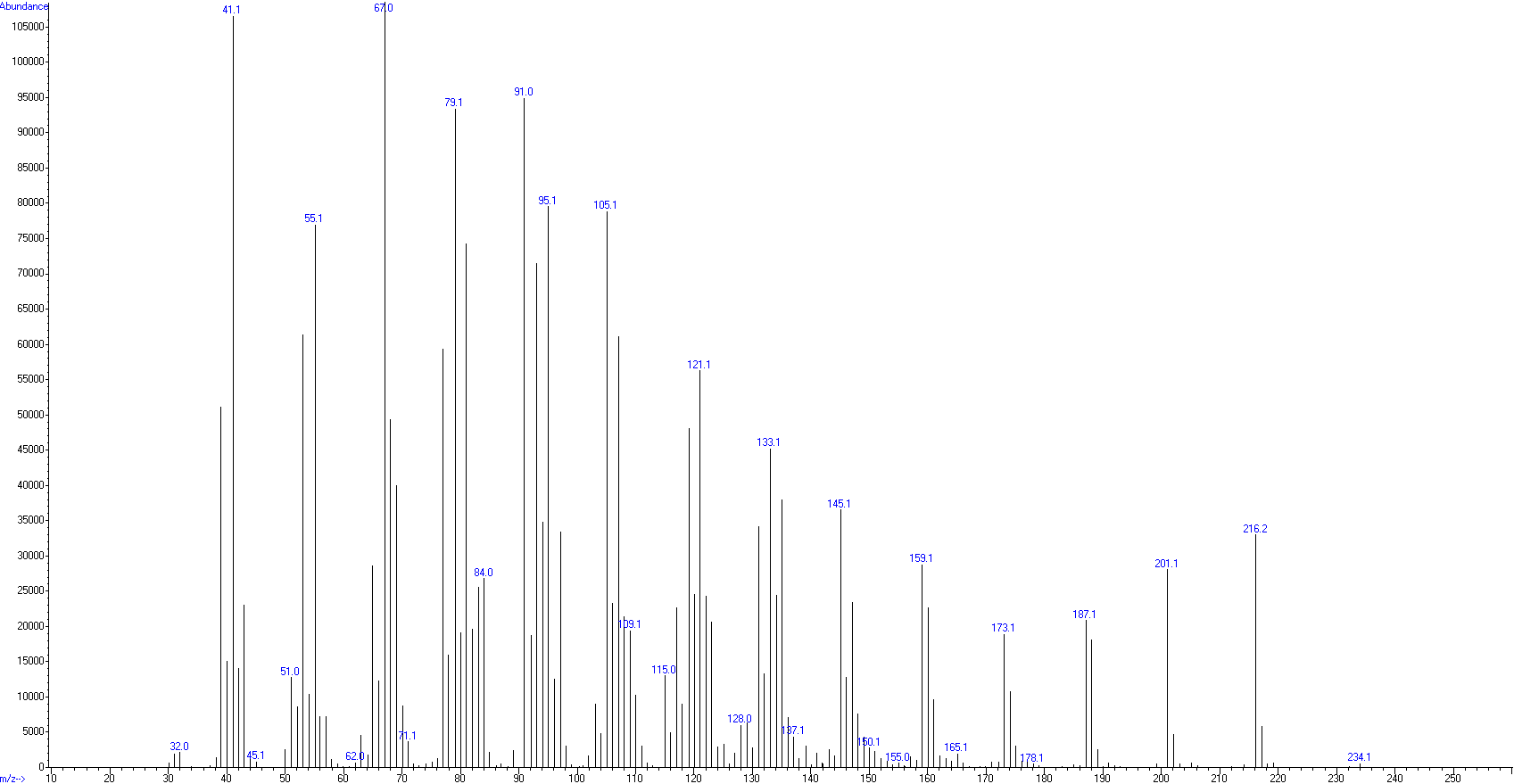


Figure S119. Mass spectrum of compound **37** (EI-MS).

#### Terpenoid 38; RI = 1633; m/z 216


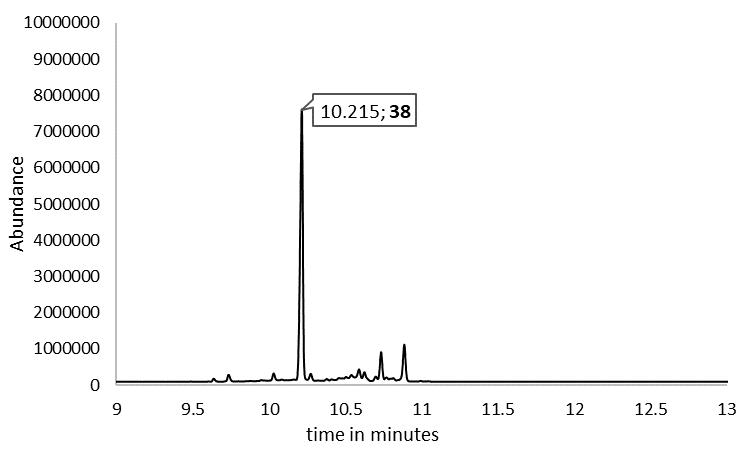


Figure S120. GC chromatogram of compound **38**.


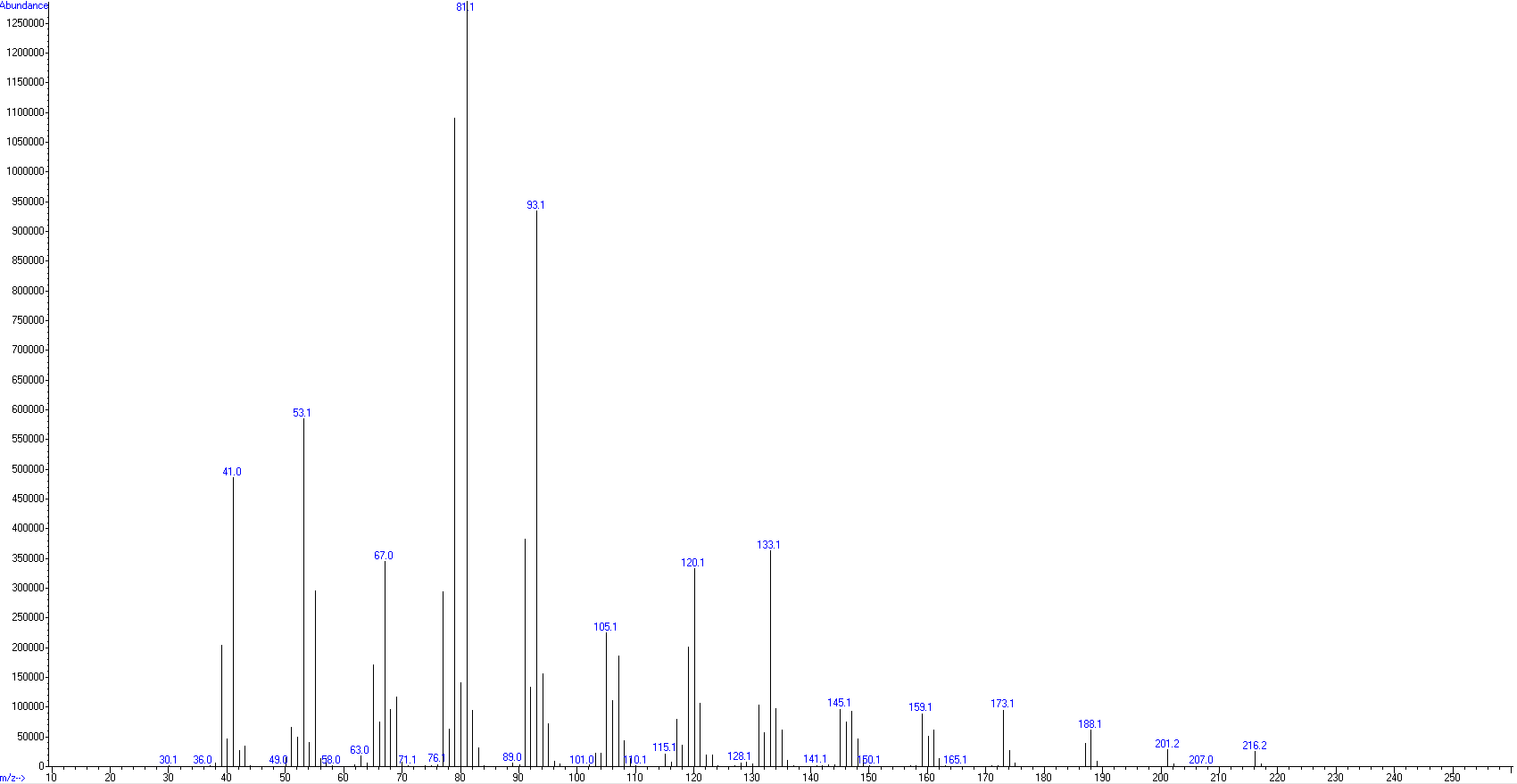


Figure S121. Mass spectrum of compound **38** (EI-MS).

#### Terpenoid 49; RI = 1782; m/z 232


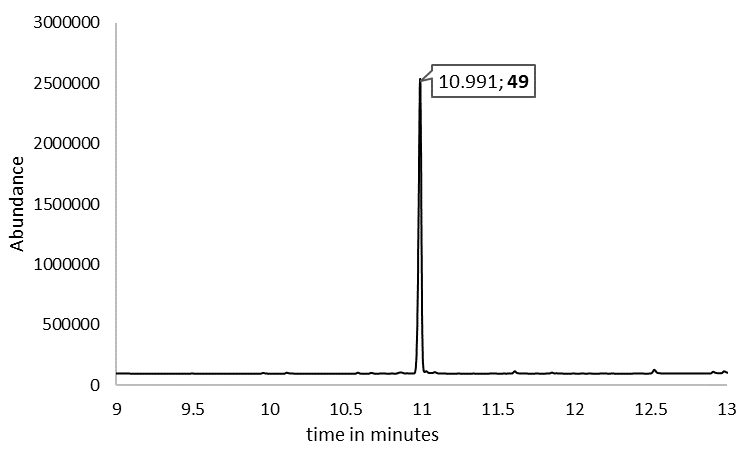


Figure S122. GC chromatogram of compound **49**.


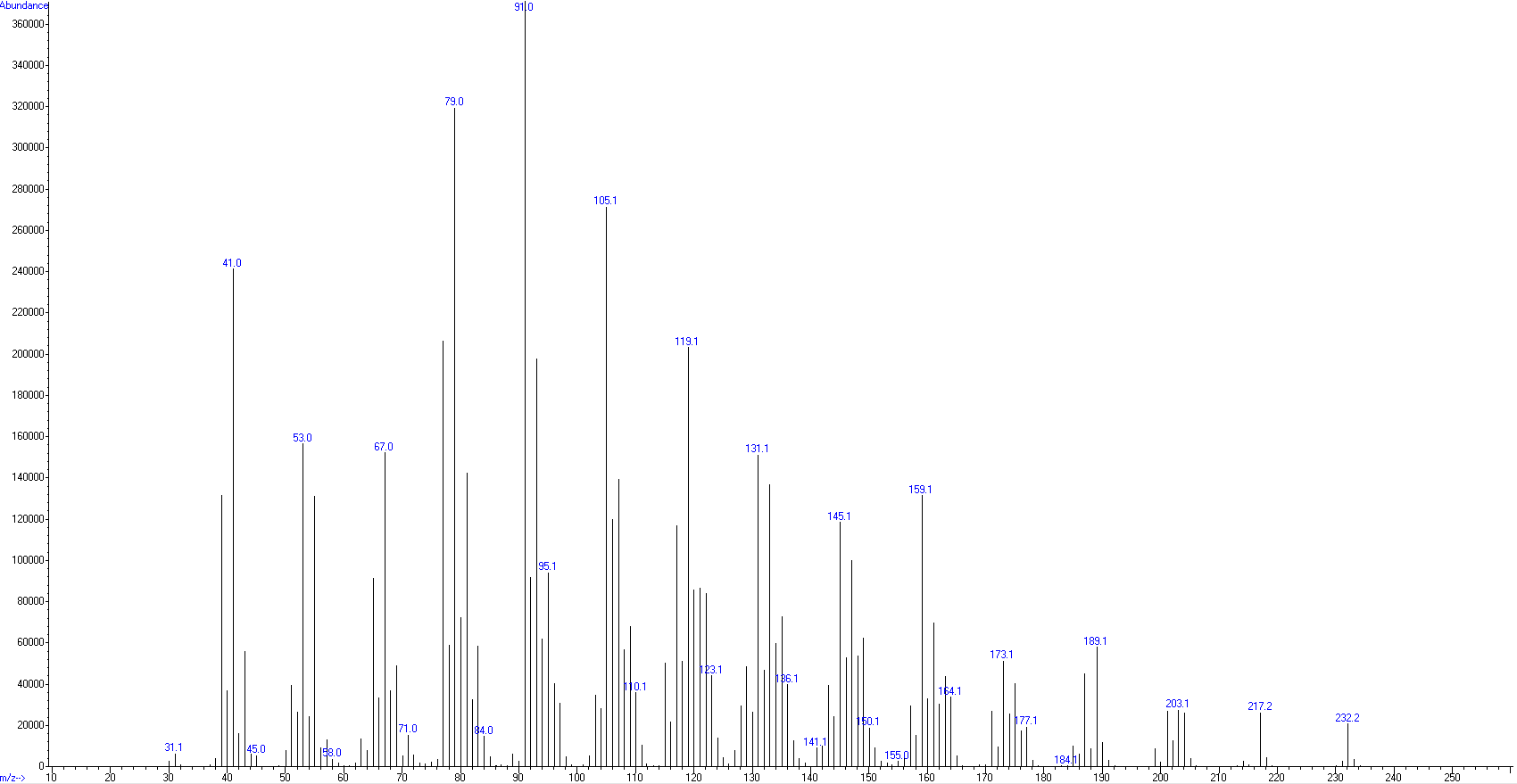


Figure S123. Mass spectrum of compound **49** (EI-MS).

#### Terpenoid 50; RI = 1782; m/z 232


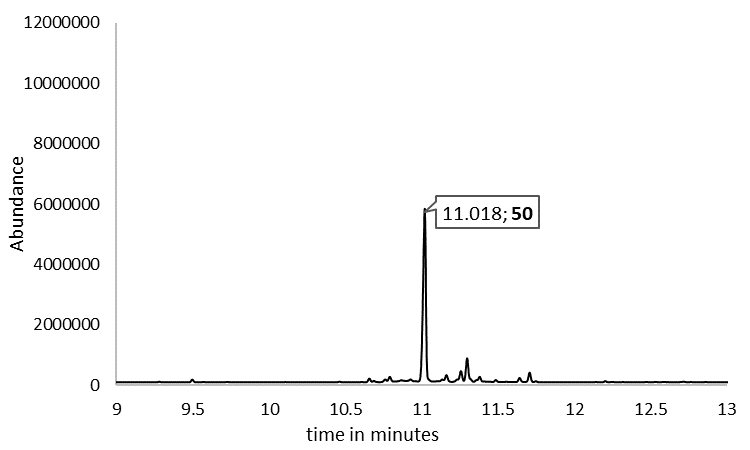


Figure S124. GC chromatogram of compound **50**.


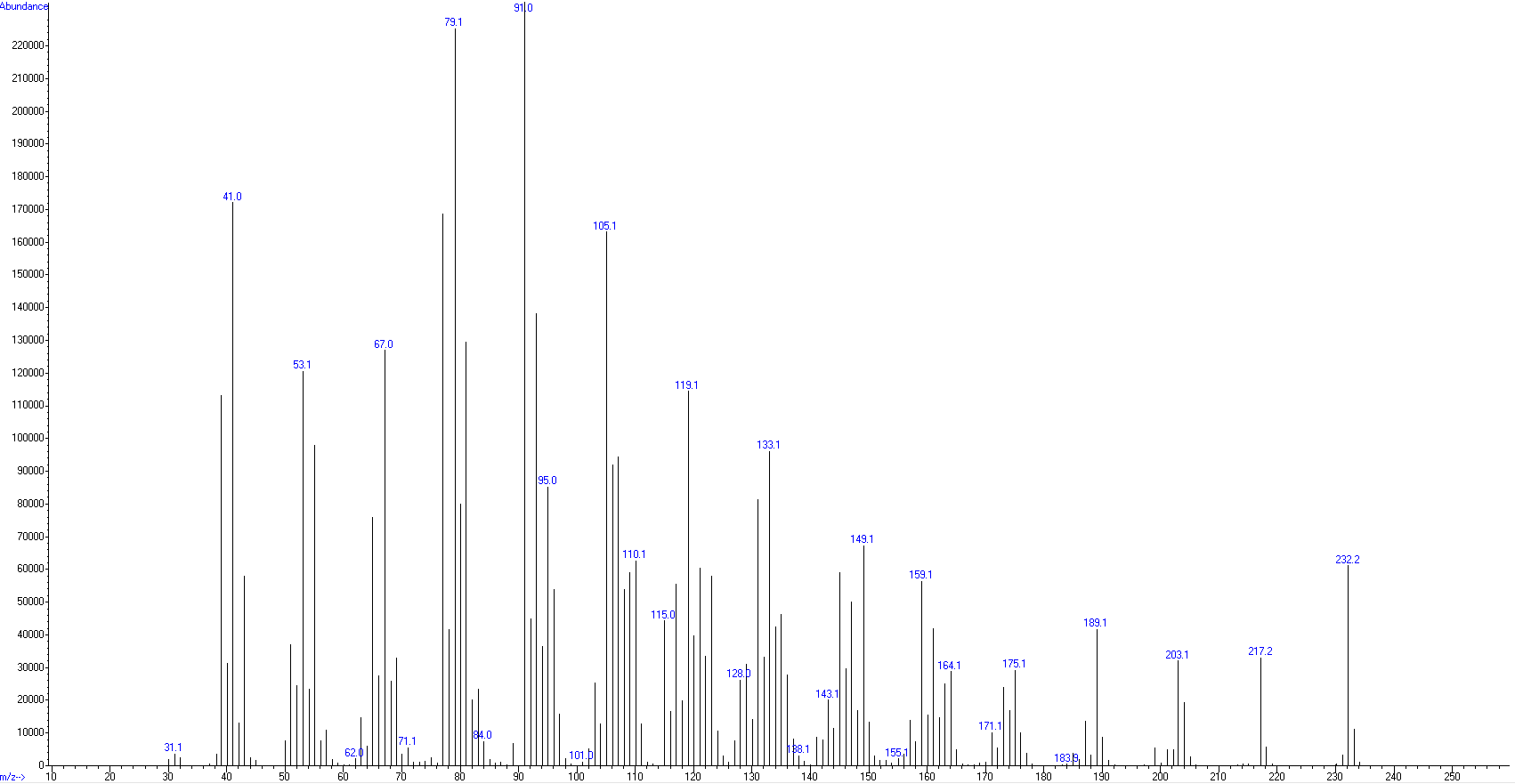


Figure S125. Mass spectrum of compound **50** (EI-MS).

#### Terpenoid 51; RI = 1902; m/z 232


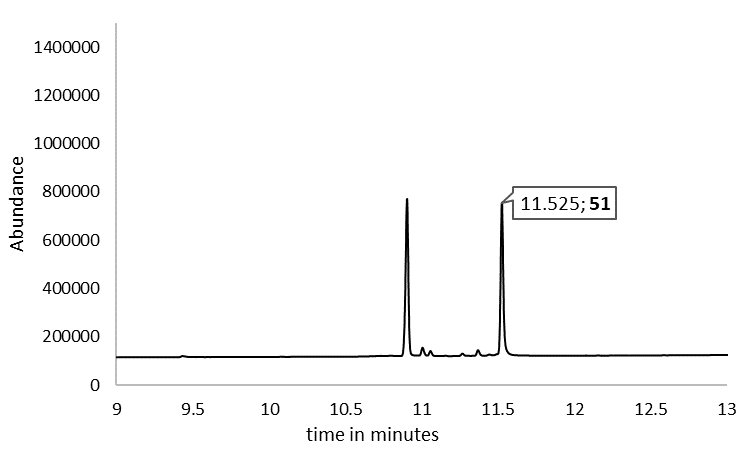


Figure S126. GC chromatogram of compound **51**.


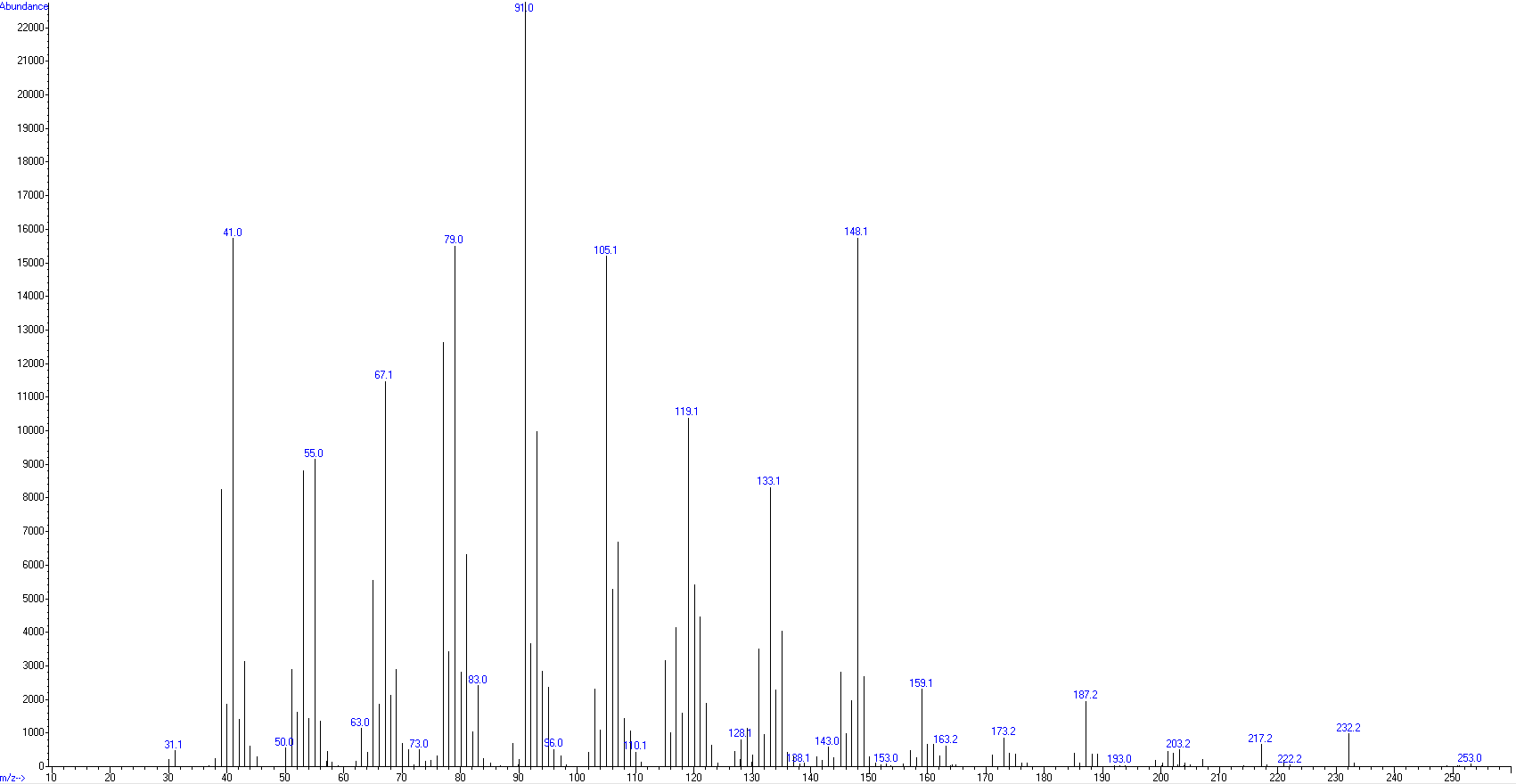


Figure S127. Mass spectrum of compound **51** (EI-MS).

#### Terpenoid 39; RI = 1989; m/z 218


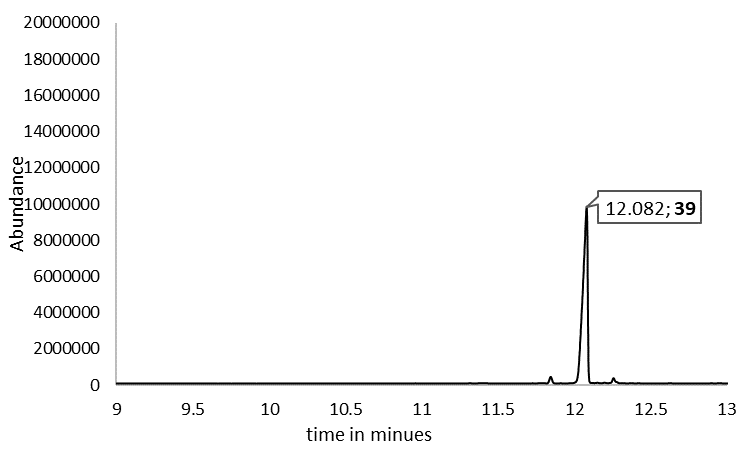


Figure S128. GC chromatogram of compound **39**.


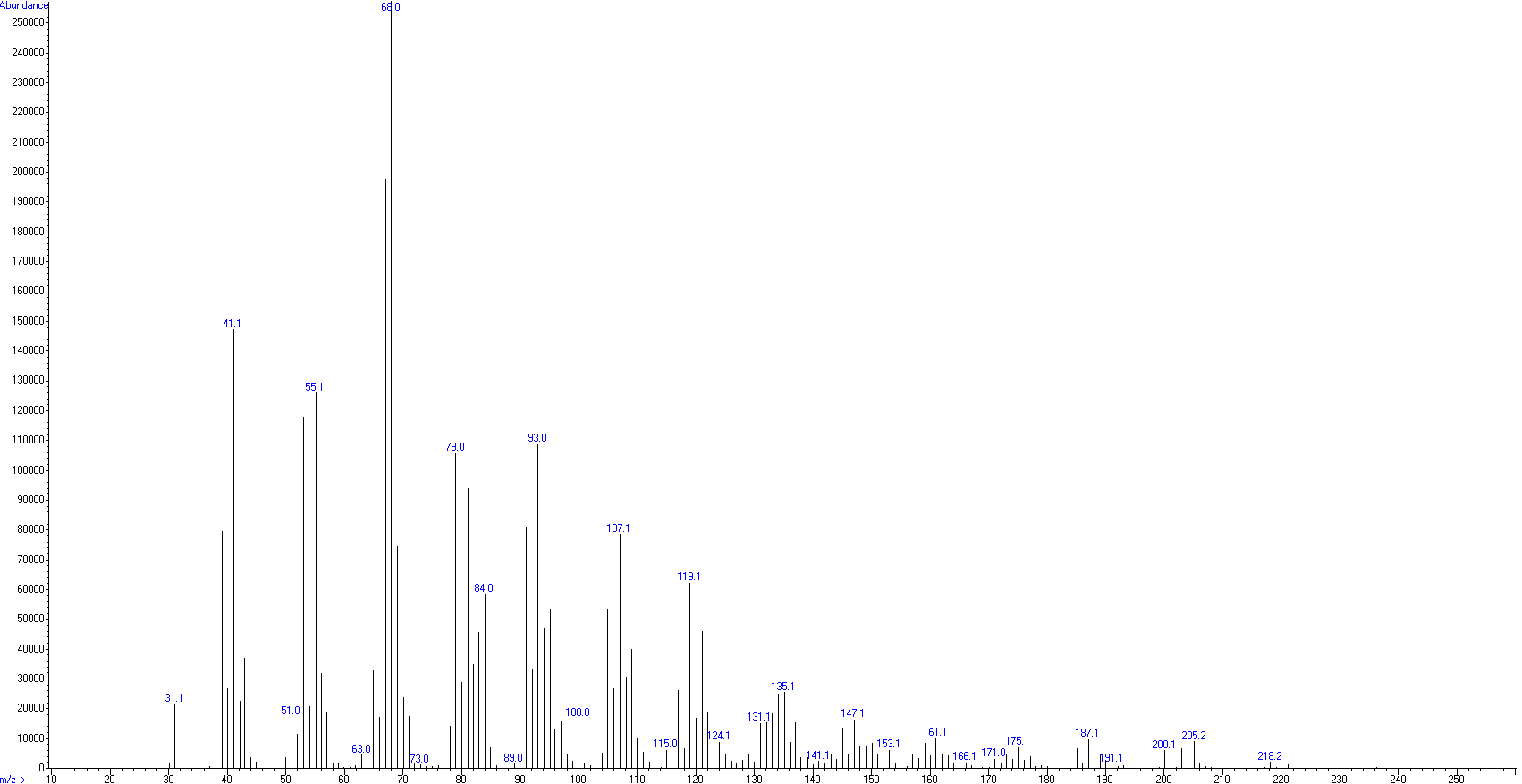


Figure S129. Mass spectrum of compound **39** (EI-MS).

#### Terpenoid 40; RI = 2057; m/z 236


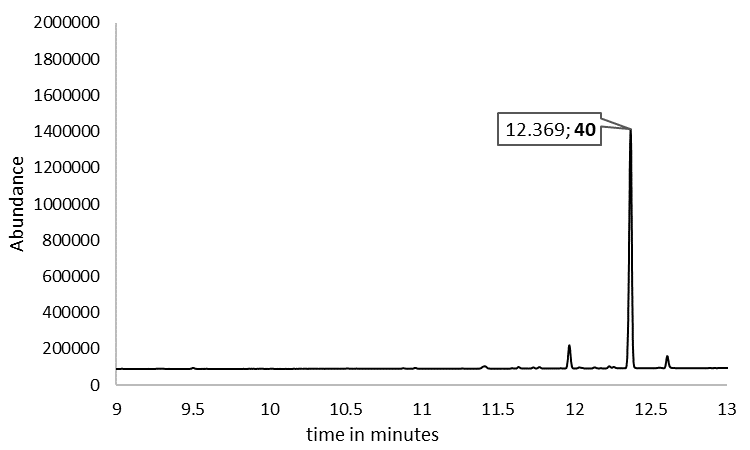


Figure S130. GC chromatogram of compound **40**.


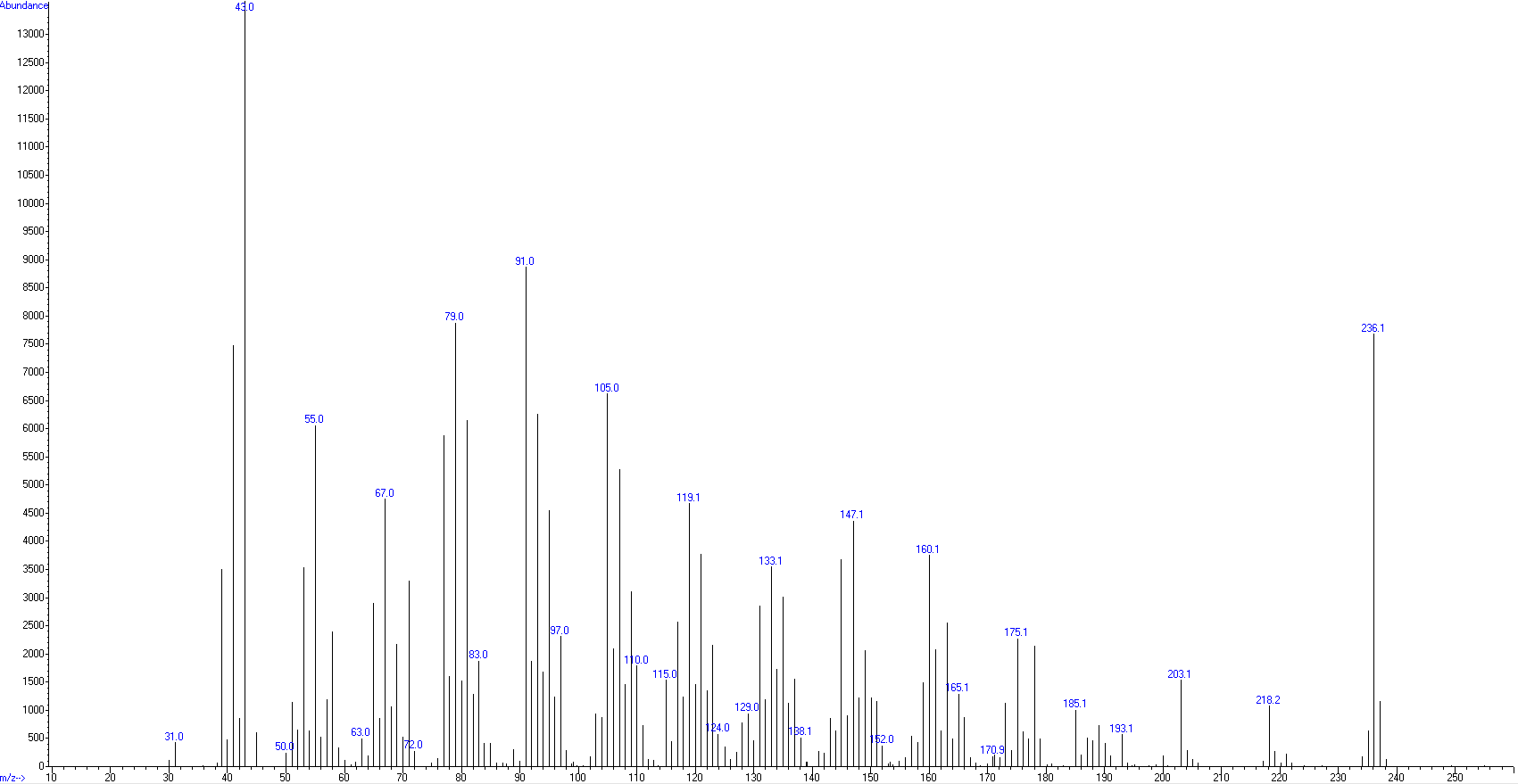


Figure S131. Mass spectrum of compound **40** (EI-MS).

#### Terpenoids 41 and 42; RI = 1863; m/z 218


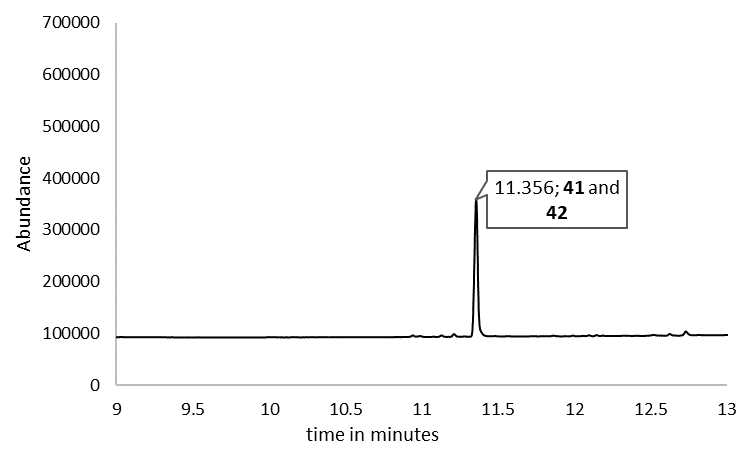


Figure S132. GC chromatogram of compounds **41** and **42**.


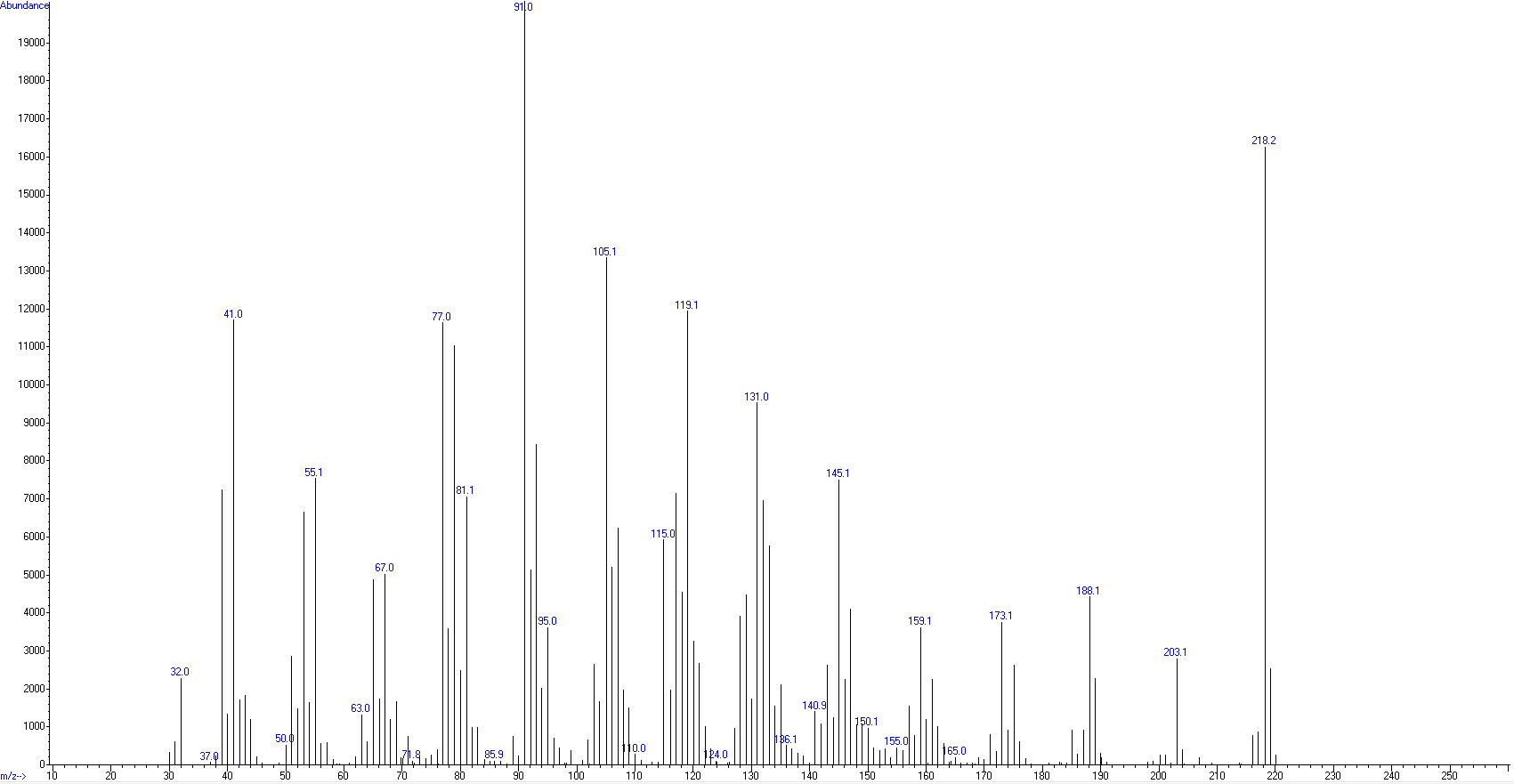


Figure S133. Mass spectrum of compounds **41** and **42** (EI-MS).

#### Terpenoid 43; RI = 1685; m/z 218


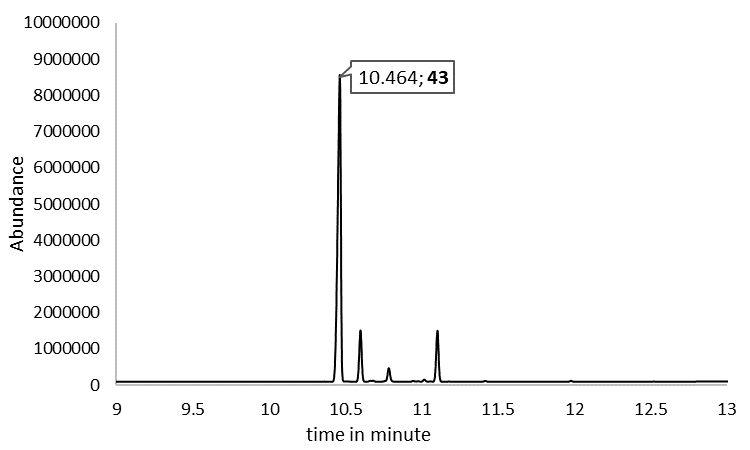


Figure S134. GC chromatogram of compound **43**.


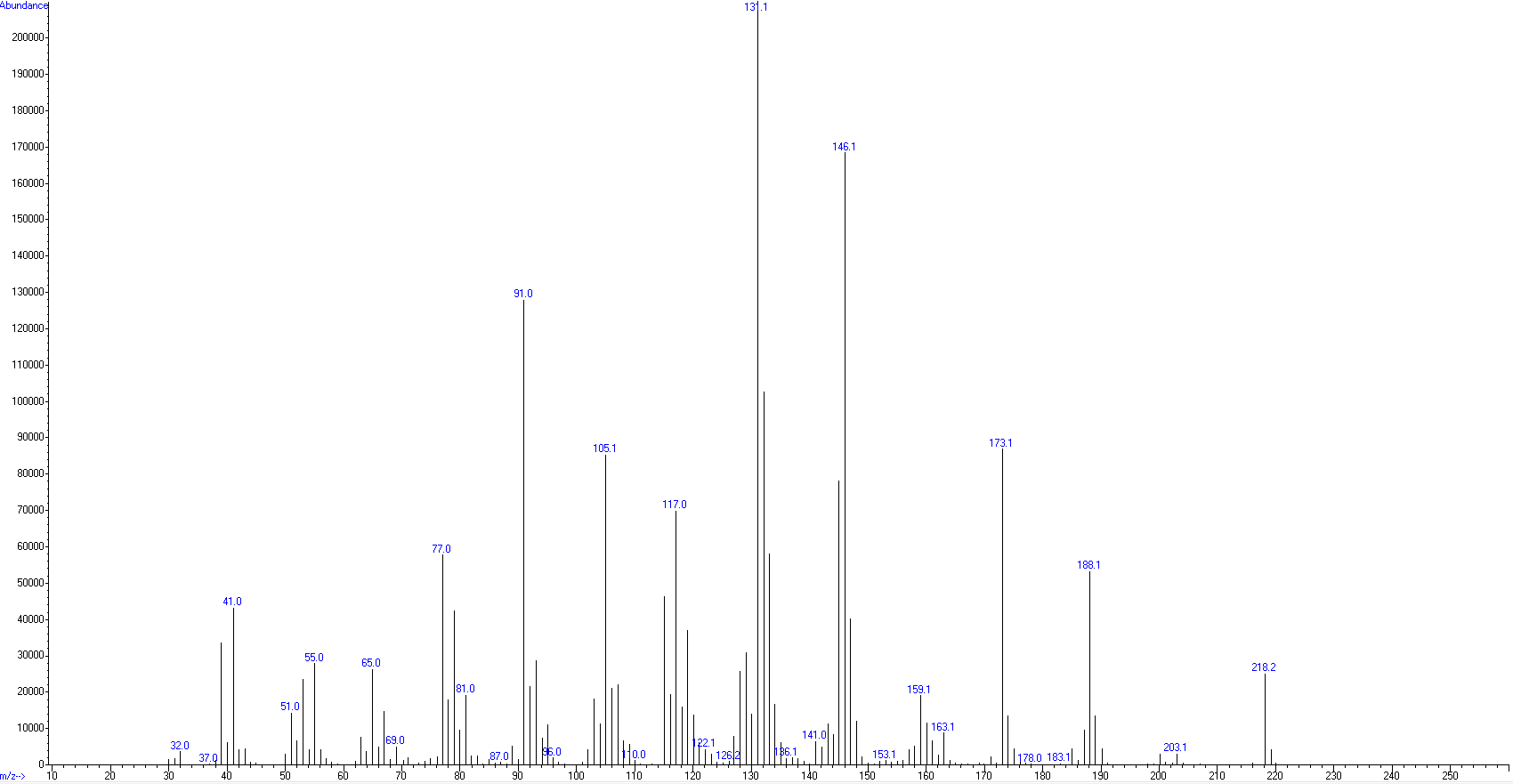


Figure S135. Mass spectrum of compound **43** (EI-MS).

#### Terpenoid 44; RI = 1707; m/z 218


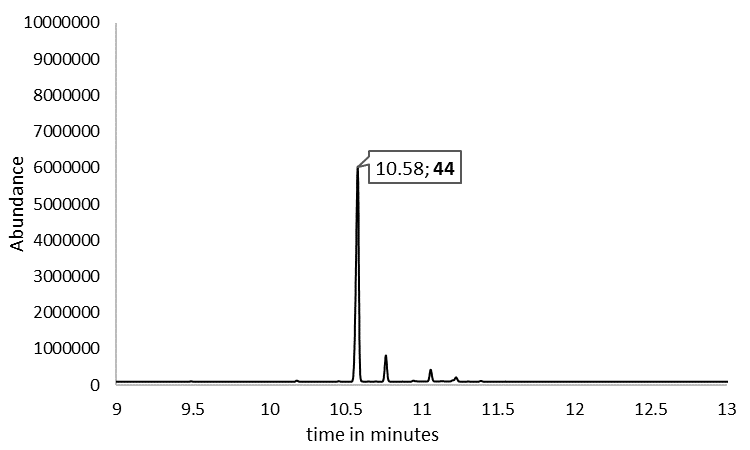


Figure S136. GC chromatogram of compound **44**.


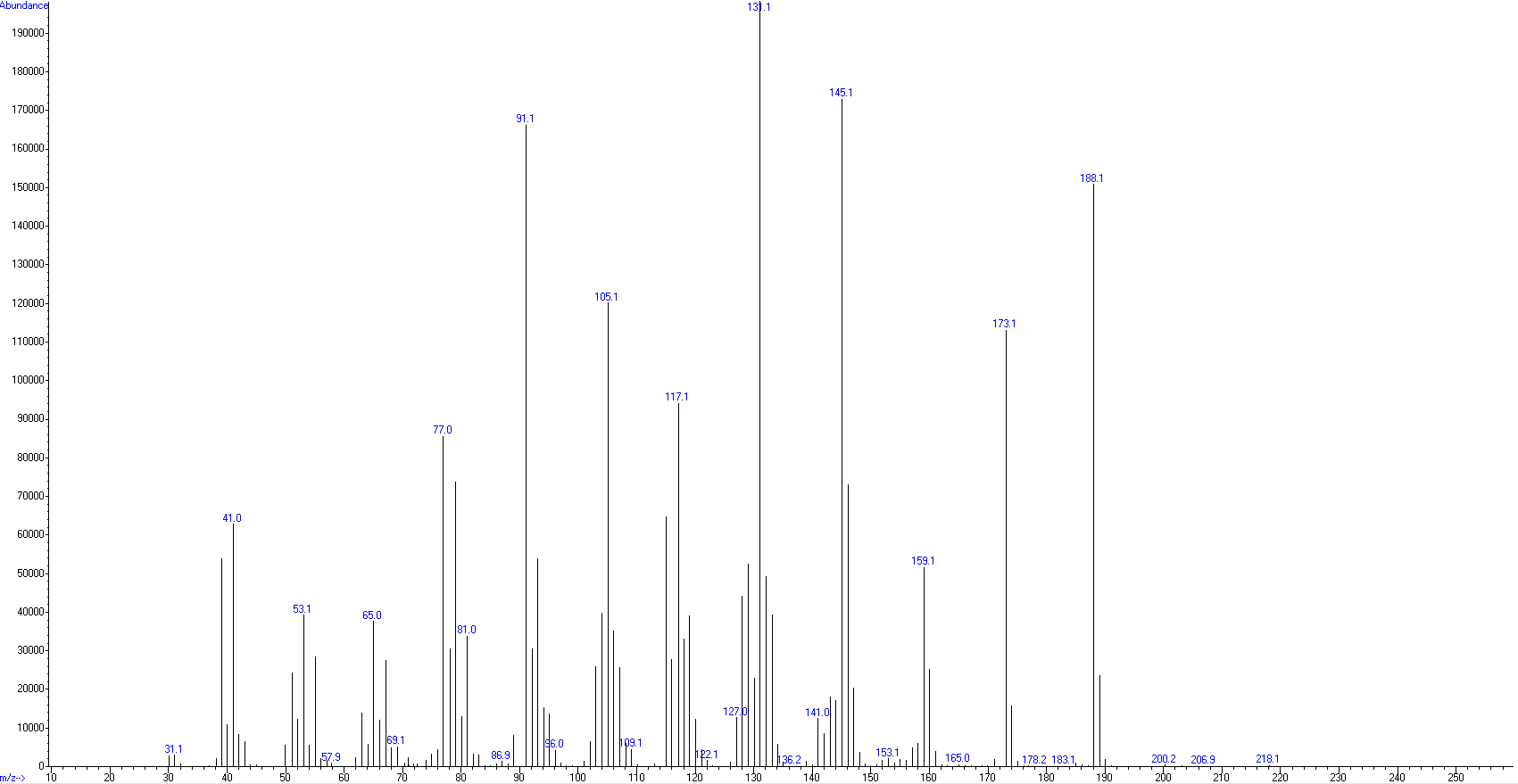


Figure S137. Mass spectrum of compound **44** (EI-MS).

# Copies of NMR spectra

#### (2*E*,6*E*)-3,7,11-Trimethyldodeca-2,6,10-trien-1-ol (SI‑1)

Figure S138. ^1^H NMR spectrum of compound **SI‑1** (400 MHz, CDCl_3_, 298 K).

Figure S139. ^13^C NMR spectrum of compound **SI-1** (101 MHz, CDCl_3_, 298 K).

#### (2*E*,6*E*)-3,7,11-Trimethyldodeca-2,6,10-trien-1-yl acetate (**7)**

Figure S140. ^1^H NMR spectrum of compound **7** (400 MHz, CDCl_3_, 298 K).

Figure S141. ^13^C NMR spectrum of compound **7** (101 MHz, CDCl_3_, 298 K).

#### (2*E*,6*E*)-10-Cyclopropylidene-3,7-dimethyldeca-2,6-dien-1-yl acetate (SI-2)

Figure S142. ^1^H NMR spectrum of compound **SI-2** (400 MHz, CDCl_3_, 298 K).

Figure S143. ^13^C NMR spectrum of compound **SI-2** (101 MHz, CDCl_3_, 298 K).

#### (2*E*,6*E*)-10-Cyclopropylidene-3,7-dimethyldeca-2,6-dien-1-ol (9)

Figure S144. ^1^H NMR spectrum of compound **9** (400 MHz, CDCl_3_, 298 K).

Figure S145. ^13^C NMR spectrum of compound **9** (101 MHz, CDCl_3_, 298 K).

#### Triammonium (2*E*,6*E*)-10-cyclopropylidene-3,7-dimethyldeca-2,6-dien-1-yl diphosphate (4a)

Figure S146. ^1^H NMR spectrum of compound **4a** (400 MHz, D_2_O, 298 K).

Figure S147. ^13^C NMR spectrum of compound **4a** (101 MHz, D_2_O, 298 K).

Figure S148. ^31^P NMR spectrum of compound **4a** (162 MHz, D_2_O, 298 K).

#### 1-(*tert*-Butyl)-1*H*-tetrazole-5-thiol (SI-3)

Figure S149. ^1^H NMR spectrum of compound **SI-3** (400 MHz, CDCl_3_, 298 K).

Figure S150. ^13^C NMR spectrum of compound **SI‑3** (101 MHz, CDCl_3_, 298 K).

#### 5-((3-Bromopropyl)thio)-1-(*tert*-butyl)-1*H*-tetrazole (SI‑4)

Figure S151. ^1^H NMR spectrum of compound **SI‑4** (400 MHz, CDCl_3_, 298 K).

Figure S152. ^13^C NMR spectrum of compound **SI‑4** (101 MHz, CDCl_3_, 298 K).

#### 1-(*tert*-Butyl)-5-(cyclopropylthio)-1*H*-tetrazole (8b)

Figure S153. ^1^H NMR spectrum of compound **8b** (400 MHz, CDCl_3_, 298 K).

Figure S154. ^13^C NMR spectrum of compound **8b** (101 MHz, CDCl_3_, 298 K).

#### (*E*)-3,7-Dimethylocta-2,6-dien-1-yl acetate (SI‑5)

Figure S155. ^1^H NMR spectrum of compound **SI‑5** (400 MHz, CDCl_3_, 298 K).

Figure S156. ^13^C NMR spectrum of compound **SI‑5** (101 MHz, CDCl_3_, 298 K).

#### (*E*)-5-(3,3-Dimethyloxiran-2-yl)-3-methylpent-2-en-1-yl acetate (SI‑6)

Figure S157. ^1^H NMR spectrum of compound **SI-6** (400 MHz, CDCl_3_, 298 K).

Figure S158. ^13^C NMR spectrum of compound **SI‑6** (101 MHz, CDCl_3_, 298 K).

#### (*E*)-3-Methyl-6-oxohex-2-en-1-yl acetate (11)

Figure S159. ^1^H NMR spectrum of compound **11** (400 MHz, CDCl_3_, 298 K).

Figure S160. ^13^C NMR spectrum of compound **11** (101 MHz, CDCl_3_, 298 K).

#### (*E*)-6-Cyclopropylidene-3-methylhex-2-en-1-ol (12)

Figure S161. ^1^H NMR spectrum of compound **12** (400 MHz, CDCl_3_, 298 K).

Figure S162. ^13^C NMR spectrum of compound **12** (101 MHz, CDCl_3_, 298 K).

#### *tert*-Butyl(((*E*)-4-(((*E*)-6-cyclopropylidene-3-methylhex-2-en-1-yl)oxy)-3-methylbut-2-en-1-yl)oxy)diphenylsilane (SI‑7)

Figure S163. ^1^H NMR spectrum of compound **SI‑7** (400 MHz, CDCl_3_, 298 K).

Figure S164. ^13^C NMR spectrum of compound **SI‑7** (101 MHz, CDCl_3_, 298 K).

#### (*E*)-4-(((*E*)-6-Cyclopropylidene-3-methylhex-2-en-1-yl)oxy)-3-methylbut-2-en-1-ol (14)

Figure S165. ^1^H NMR spectrum of compound **14** (400 MHz, CDCl_3_, 298 K).

Figure S166. ^13^C NMR spectrum of compound **14** (101 MHz, CDCl_3_, 298 K).

#### Triammonium (*E*)-4-(((*E*)-6-cyclopropylidene-3-methylhex-2-en-1-yl)oxy)-3-methylbut-2-en-1-yl diphosphate (5a)

Figure S167. ^1^H NMR spectrum of compound **5a** (400 MHz, D_2_O, 298 K).

Figure S168. ^13^C NMR spectrum of compound **5a** (101 MHz, D_2_O, 298 K).

Figure S169. ^31^P NMR spectrum of compound **5a** (162 MHz, D_2_O, 298 K).

#### (2*E*,6*E*)-10-Cyclobutylidene-3,7-dimethyldeca-2,6-dien-1-yl acetate (SI‑8)

Figure S170. ^1^H NMR spectrum of compound **SI‑8** (400 MHz, CDCl_3_, 298 K).

Figure S171. ^13^C NMR spectrum of compound **SI‑8** (101 MHz, CDCl_3_, 298 K).

#### (2*E*,6*E*)-10-Cyclobutylidene-3,7-dimethyldeca-2,6-dien-1-ol (16)

Figure S172. ^1^H NMR spectrum of compound **16** (400 MHz, CDCl_3_, 298 K).

Figure S173. ^13^C NMR spectrum of compound **16** (101 MHz, CDCl_3_, 298 K).

#### Triammonium (2*E*,6*E*)-10-cyclobutylidene-3,7-dimethyldeca-2,6-dien-1-yl diphosphate (4b)

Figure S174. ^1^H NMR spectrum of compound **4b** (400 MHz, D_2_O, 298 K).

Figure S175. ^13^C NMR spectrum of compound **4b** (126 MHz, D_2_O, 298 K).

Figure S176. ^31^P NMR spectrum of compound **4b** (162 MHz, D_2_O, 298 K).

#### (*E*)-6-Cyclobutylidene-3-methylhex-2-en-1-ol (17)

Figure S177. ^1^H NMR spectrum of compound **17** (400 MHz, CDCl_3_, 298 K).

Figure S178. ^13^C NMR spectrum of compound **17** (101 MHz, CDCl_3_, 298 K).

#### *tert*-Butyl(((*E*)-4-(((*E*)-6-cyclobutylidene-3-methylhex-2-en-1-yl)oxy)-3-methylbut-2-en-1-yl)oxy)diphenylsilane (SI‑9)

Figure S179. ^1^H NMR spectrum of compound **SI‑9** (400 MHz, CDCl_3_, 298 K).

Figure S180. ^13^C NMR spectrum of compound **SI‑9** (101 MHz, CDCl_3_, 298 K).

#### (*E*)-3-(((*E*)-6-Cyclobutylidene-3-methylhex-2-en-1-yl)oxy)but-2-en-1-ol (18)

Figure S181. ^1^H NMR spectrum of compound **18** (400 MHz, CDCl_3_, 298 K).

Figure S182. ^13^C NMR spectrum of compound **18** (101 MHz, CDCl_3_, 298 K).

#### Triammonium (*E*)-4-(((*E*)-6-cyclobutylidene-3-methylhex-2-en-1-yl)oxy)-3-methylbut-2-en-1-yl diphosphate (5b)

Figure S183. ^1^H NMR spectrum of compound **5b** (400 MHz, D_2_O, 298 K).

Figure S184. ^13^C NMR spectrum of compound **5b** (151 MHz, D_2_O, 298 K).

Figure S185. ^31^P NMR spectrum of compound **5b** (162 MHz, D_2_O, 298 K).

#### *tert*-Butyldiphenyl(((2*E*,6*E*)-3,7,11-trimethyldodeca-2,6,10-trien-1-yl)oxy)silane (SI‑10)

Figure S186. ^1^H NMR spectrum of compound **SI‑10** (400 MHz, CDCl_3_, 298 K).

Figure S187. ^13^C NMR spectrum of compound **SI‑10** (101 MHz, CDCl_3_, 298 K).

#### (4*E*,8*E*)-10-((*tert*-Butyldiphenylsilyl)oxy)-4,8-dimethyldeca-4,8-dienal (19)

Figure S188. ^1^H NMR spectrum of compound **19** (400 MHz, CDCl_3_, 298 K).

Figure S189. ^13^C NMR spectrum of compound **19** (101 MHz, CDCl_3_, 298 K).

#### (4*E*,8*E*)-10-((*tert*-Butyldiphenylsilyl)oxy)-4,8-dimethyldeca-4,8-dien-1-ol (SI‑11)

Figure S190. ^1^H NMR spectrum of compound **SI‑11** (400 MHz, CDCl_3_, 298 K).

Figure S191. ^13^C NMR spectrum of compound **SI‑11** (101 MHz, CDCl_3_, 298 K).

#### *tert*-Butyl(((2*E*,6*E*)-10-iodo-3,7-dimethyldeca-2,6-dien-1-yl)oxy)diphenylsilane (SI‑12)

Figure S192. ^1^H NMR spectrum of compound **SI‑12** (400 MHz, CDCl_3_, 298 K).

Figure S193. ^13^C NMR spectrum of compound **SI­‑12** (101 MHz, CDCl_3_, 298 K).

#### ((4*E*,8*E*)-10-((*tert*-Butyldiphenylsilyl)oxy)-4,8-dimethyldeca-4,8-dien-1-yl)iodotriphenyl-l5-phosphane (20)

Figure S194. ^1^H NMR spectrum of compound **20** (400 MHz, CDCl_3_, 298 K).

Figure S195. ^13^C NMR spectrum of compound **20** (101 MHz, CDCl_3_, 298 K).

#### *tert*-Butyl(((2*E*,6*E*)-3,7-dimethyl-10-(oxetan-3-ylidene)deca-2,6-dien-1-yl)oxy)diphenylsilane (SI‑13)

Figure S196. ^1^H NMR spectrum of compound **SI‑13** (400 MHz, CDCl_3_, 298 K).

Figure S197. ^13^C NMR spectrum of compound **SI‑13** (101 MHz, CDCl_3_, 298 K).

#### (2*E*,6*E*)-3,7-Dimethyl-10-(oxetan-3-ylidene)deca-2,6-dien-1-ol (22)

Figure S198. ^1^H NMR spectrum of compound **22** (400 MHz, CDCl_3_, 298 K).

Figure S199. ^13^C NMR spectrum of compound **22** (101 MHz, CDCl_3_, 298 K).

#### Triammonium (2*E*,6*E*)-3,7-dimethyl-10-(oxetan-3-ylidene)deca-2,6-dien-1-yl diphosphate (4c)

Figure S200. ^1^H NMR spectrum of compound **4c** (400 MHz, D_2_O, 298 K).

Figure S201. ^13^C NMR spectrum of compound **4c** (151 MHz, D_2_O, 298 K).

Figure S202. ^31^P NMR spectrum of compound **4c** (162 MHz, D_2_O, 298 K).

#### (*E*)-*tert*-Butyl((3,7-dimethylocta-2,6-dien-1-yl)oxy)diphenylsilane (SI‑14)

Figure S203. ^1^H NMR spectrum of compound **SI‑14** (400 MHz, CDCl_3_, 298 K).

Figure S204. ^13^C NMR spectrum of compound **SI‑14** (101 MHz, CDCl_3_, 298 K).

#### (*E*)-6-((*tert*-Butyldiphenylsilyl)oxy)-4-methylhex-4-enal (23)

Figure S205. ^1^H NMR spectrum of compound **23** (400 MHz, CDCl_3_, 298 K).

Figure S206. ^13^C NMR spectrum of compound **23** (101 MHz, CDCl_3_, 298 K).

#### (*E*)-6-((*tert*-Butyldiphenylsilyl)oxy)-4-methylhex-4-en-1-ol (SI‑15)

Figure S207. ^1^H NMR spectrum of compound **SI‑15** (400 MHz, CDCl_3_, 298 K).

Figure S208. ^13^C NMR spectrum of compound **SI‑15** (101 MHz, CDCl_3_, 298 K).

#### (*E*)-6-((*tert*-Butyldiphenylsilyl)oxy)-4-methylhex-4-en-1-yl methanesulfonate (SI‑16)

Figure S209. ^1^H NMR spectrum of compound **SI‑16** (400 MHz, CDCl_3_, 298 K).

Figure S210. ^13^C NMR spectrum of compound **SI‑16** (101 MHz, CDCl_3_, 298 K).

#### (*E*)-*tert*-Butyl((6-iodo-3-methylhex-2-en-1-yl)oxy)diphenylsilane (SI‑17)

Figure S211. ^1^H NMR spectrum of compound **SI‑17** (400 MHz, CDCl_3_, 298 K).

Figure S212. ^13^C NMR spectrum of compound **SI‑17** (101 MHz, CDCl_3_, 298 K).

#### (*E*)-(6-((*tert*-Butyldiphenylsilyl)oxy)-4-methylhex-4-en-1-yl)iodotriphenyl-l5-phosphane (24)

Figure S213. ^1^H NMR spectrum of compound **24** (400 MHz, CDCl_3_, 298 K).

Figure S214. ^13^C NMR spectrum of compound **24** (101 MHz, CDCl_3_, 298 K).

#### (*E*)-*tert*-Butyl((3-methyl-6-(oxetan-3-ylidene)hex-2-en-1-yl)oxy)diphenylsilane (SI‑18)

Figure S215. ^1^H NMR spectrum of compound **SI‑18** (400 MHz, CDCl_3_, 298 K).

Figure S216. ^13^C NMR spectrum of compound **SI‑18** (101 MHz, CDCl_3_, 298 K).

#### (*E*)-3-Methyl-6-(oxetan-3-ylidene)hex-2-en-1-ol (25)

Figure S217. ^1^H NMR spectrum of compound **25** (400 MHz, CDCl_3_, 298 K).

Figure S218. ^13^C NMR spectrum of compound **25** (101 MHz, CDCl_3_, 298 K).

#### *tert*-Butyl((3-methylbut-2-en-1-yl)oxy)diphenylsilane (SI‑19)

Figure S219. ^1^H NMR spectrum of compound **SI‑19** (400 MHz, CDCl_3_, 298 K).

Figure S220. ^13^C NMR spectrum of compound **SI‑19** (101 MHz, CDCl_3_, 298 K).

#### (*E*)-4-((*tert*-Butyldiphenylsilyl)oxy)-2-methylbut-2-en-1-ol (SI‑20)

Figure S221. ^1^H NMR spectrum of compound **SI‑20** (400 MHz, CDCl_3_, 298 K).

Figure S222. ^13^C NMR spectrum of compound **SI‑20** (101 MHz, CDCl_3_, 298 K).

#### (*E*)-((4-Bromo-3-methylbut-2-en-1-yl)oxy)(tert-butyl)diphenylsilane (13)

Figure S223. ^1^H NMR spectrum of compound **13** (400 MHz, CDCl_3_, 298 K).

Figure S224. ^13^C NMR spectrum of compound **13** (101 MHz, CDCl_3_, 298 K).

#### *tert*-Butyl(((*E*)-3-methyl-4-(((*E*)-3-methyl-6-(oxetan-3-ylidene)hex-2-en-1-yl)oxy)but-2-en-1-yl)oxy)diphenylsilane (SI‑21)

Figure S225. ^1^H NMR spectrum of compound **SI‑21** (400 MHz, CDCl_3_, 298 K).

Figure S226. ^13^C NMR spectrum of compound **SI‑21** (101 MHz, CDCl_3_, 298 K).

#### (*E*)-3-Methyl-4-(((*E*)-3-methyl-6-(oxetan-3-ylidene)hex-2-en-1-yl)oxy)but-2-en-1-ol (26)

Figure S227. ^1^H NMR spectrum of compound **26** (400 MHz, CDCl_3_, 298 K).

Figure S228. ^13^C NMR spectrum of compound **26** (101 MHz, CDCl_3_, 298 K).

#### Triammonium (*E*)-3-methyl-4-(((*E*)-3-methyl-6-(oxetan-3-ylidene)hex-2-en-1-yl)oxy)but-2-en-1-yl diphosphate (5c)

Figure S229. ^1^H NMR spectrum of compound **5c** (600 MHz, D_2_O, 298 K).

Figure S230. ^13^C NMR spectrum of compound **5c** (151 MHz, D_2_O, 298 K).

Figure S231. ^31^P NMR spectrum of compound **5c** (162 MHz, D_2_O, 298 K).

#### Mosher ester 35a

Figure S232. ^1^H NMR spectrum of compound **35a** (600 MHz, C_6_D_6_, 298 K).

Figure S233. ^13^C NMR spectrum of compound **35a** (151 MHz, C_6_D_6_, 298 K).

Figure S234. ^19^F NMR spectrum of compound **35a** (376 MHz, C_6_D_6_, 298 K).

Figure S235. ^1^H-^1^H-COSY NMR spectrum of compound **35a** (500 MHz, C_6_D_6_, 298 K).

Figure S236. ^1^H-^13^C-H2BC NMR spectrum of compound **35a** (^1^H: 500 MHz, ^13^C: 126 MHz, C_6_D_6_, 298 K).

Figure S237. ^1^H-^13^C-HMBC NMR spectrum of compound **35a** (^1^H: 500 MHz, ^13^C: 126 MHz, C_6_D_6_, 298 K).

Figure S238. ^1^H-^13^C-HSQC NMR spectrum of compound **35a** (^1^H: 500 MHz, ^13^C: 126 MHz, C_6_D_6_, 298 K).

Figure S239. ^1^H-^1^H-NOESY NMR spectrum of compound **35a** (500 MHz, C_6_D_6_, 298 K).

#### Mosher ester 35b

Figure S240. ^1^H NMR spectrum of compound **35b** (500 MHz, C_6_D_6_, 298 K).

Figure S241. ^13^C NMR spectrum of compound **35b** (126 MHz, C_6_D_6_, 298 K).

Figure S242. ^19^F NMR spectrum of compound **35b** (376 MHz, C_6_D_6_, 298 K).

Figure S243. ^1^H-^1^H-COSY NMR spectrum of compound **35b** (500 MHz, C_6_D_6_, 298 K).

Figure S244. ^1^H-^13^C-H2BC NMR spectrum of compound **35b** (^1^H: 500 MHz, ^13^C: 126 MHz, C_6_D_6_, 298 K).

Figure S245. ^1^H-^13^C-HMBC NMR spectrum of compound **35b** (^1^H: 500 MHz, ^13^C: 126 MHz, C_6_D_6_, 298 K).

Figure S246. ^1^H-^13^C-HSQC NMR spectrum of compound **35b** (^1^H: 500 MHz, ^13^C: 126 MHz, C_6_D_6_, 298 K).

Figure S247. ^1^H-^1^H-NOESY NMR spectrum of compound **35b** (500 MHz, C_6_D_6_, 298 K).

#### Terpenoid 35

Figure S248. ^1^H NMR spectrum of compound **35** (400 MHz, C_6_D_6_, 298 K).

Figure S249. ^13^C NMR spectrum of compound **35** (101 MHz, C_6_D_6_, 298 K).

Figure S250. ^1^H-^1^H-COSY NMR spectrum of compound **35** (400 MHz, C_6_D_6_, 298 K).

Figure S251. ^1^H-^13^C-H2BC NMR spectrum of compound **35** (^1^H: 500 MHz, ^13^C: 126 MHz, C_6_D_6_, 298 K).

Figure S252. ^1^H-^13^C-HMBC NMR spectrum of compound **35** (^1^H: 400 MHz, ^13^C: 101 MHz, C_6_D_6_, 298 K).

Figure S253. ^1^H-^13^C-HSQC NMR spectrum of compound **35** (^1^H: 400 MHz, ^13^C: 101 MHz, C_6_D_6_, 298 K).

Figure S254. ^1^H-^1^H-NOESY NMR spectrum of compound **35** (500 MHz, C_6_D_6_, 298 K).

#### Terpenoid 32

Figure S255. ^1^H NMR spectrum of compound **32** (600 MHz, C_6_D_6_, 298 K).

Figure S256. ^13^C NMR spectrum of compound **32** (126 MHz, C_6_D_6_, 298 K).

Figure S257. ^1^H-^1^H-COSY NMR spectrum of compound **32** (500 MHz, C_6_D_6_, 298 K).

Figure S258. ^1^H-^13^C-HMBC NMR spectrum of compound **32** (^1^H: 500 MHz, ^13^C: 126 MHz, C_6_D_6_, 298 K).

Figure S259. ^1^H-^13^C-HSQC NMR spectrum of compound **32** (^1^H: 500 MHz, ^13^C: 126 MHz, C_6_D_6_, 298 K).

Figure S260. ^1^H-^1^H-NOESY NMR spectrum of compound **32** (500 MHz, C_6_D_6_, 298 K).

#### Terpenoid 33

Figure S261. ^1^H NMR spectrum of compound **33** (600 MHz, C_6_D_6_, 298 K).

Figure S262. ^13^C NMR spectrum of compound **33** (151 MHz, C_6_D_6_, 298 K).

Figure S263. ^1^H-^1^H-COSY NMR spectrum of compound **33** (600 MHz, C_6_D_6_, 298 K).

Figure S264. ^1^H-^13^C-H2BC NMR spectrum of compound **33** (^1^H: 500 MHz, ^13^C: 126 MHz, C_6_D_6_, 298 K).

Figure S265. ^1^H-^13^C-HMBC NMR spectrum of compound **33** (^1^H: 600 MHz, ^13^C: 151 MHz, C_6_D_6_, 298 K).

Figure S266. ^1^H-^13^C-HSQC NMR spectrum of compound **33** (^1^H: 600 MHz, ^13^C: 151 MHz, C_6_D_6_, 298 K).

Figure S267. ^1^H-^1^H-NOESY NMR spectrum of compound **33** (600 MHz, C_6_D_6_, 298 K).

#### Terpenoid 34

Figure S268. ^1^H NMR spectrum of compound **34** (600 MHz, C_6_D_6_, 298 K).

Figure S269. ^13^C NMR spectrum of compound **34** (151 MHz, C_6_D_6_, 298 K).

Figure S270. ^1^H-^1^H-COSY NMR spectrum of compound **34** (600 MHz, C_6_D_6_, 298 K).

Figure S271. ^1^H-^13^C-H2BC NMR spectrum of compound **34** (^1^H: 500 MHz, ^13^C: 126 MHz, C_6_D_6_, 298 K).

Figure S272. ^1^H-^13^C-HMBC NMR spectrum of compound **34** (^1^H: 600 MHz, ^13^C: 151 MHz, C_6_D_6_, 298 K).

Figure S273. ^1^H-^13^C-HSQC NMR spectrum of compound **34** (^1^H: 600 MHz, ^13^C: 151 MHz, C_6_D_6_, 298 K).

Figure S274. ^1^H-^1^H-NOESY NMR spectrum of compound **34** (600 MHz, C_6_D_6_, 298 K).

#### Terpenoid 46

Figure S275. ^1^H NMR spectrum of compound **46** (500 MHz, C_6_D_6_, 298 K).

Figure S276. ^13^C NMR spectrum of compound **46** (151 MHz, C_6_D_6_, 298 K).

Figure S277. ^1^H-^1^H-COSY NMR spectrum of compound **46** (500 MHz, C_6_D_6_, 298 K).

Figure S278. ^1^H-^13^C-HMBC NMR spectrum of compound **46** (^1^H: 500 MHz, ^13^C: 126 MHz, C_6_D_6_, 298 K).

Figure S279. ^1^H-^13^C-HSQC NMR spectrum of compound **46** (^1^H: 500 MHz, ^13^C: 126 MHz, C_6_D_6_, 298 K).

Figure S280. ^1^H-^1^H-NOESY NMR spectrum of compound **46** (400 MHz, C_6_D_6_, 298 K).

#### Terpenoid 36

Figure S281. ^1^H NMR spectrum of compound **36** (600 MHz, C_6_D_6_, 298 K).

Figure S282. ^13^C NMR spectrum of compound **36** (126 MHz, C_6_D_6_, 298 K).

Figure S283. ^1^H-^1^H-COSY NMR spectrum of compound **36** (600 MHz, C_6_D_6_, 298 K).

Figure S284. ^1^H-^13^C-H2BC NMR spectrum of compound **36** (^1^H: 500 MHz, ^13^C: 126 MHz, C_6_D_6_, 298 K).

Figure S285. ^1^H-^13^C-HMBC NMR spectrum of compound **36** (^1^H: 600 MHz, ^13^C: 151 MHz, C_6_D_6_, 298 K).

Figure S286. ^1^H-^13^C-HSQC NMR spectrum of compound **36** (^1^H: 600 MHz, ^13^C: 151 MHz, C_6_D_6_, 298 K).

Figure S287. ^1^H-^1^H-NOESY NMR spectrum of compound **36** (600 MHz, C_6_D_6_, 298 K).

#### Terpenoid 37

Figure S288. ^1^H NMR spectrum of compound **37** (600 MHz, C_6_D_6_, 298 K).

Figure S289. ^13^C NMR spectrum of compound **37** (151 MHz, C_6_D_6_, 298 K).

Figure S290. ^1^H-^13^C-H2BC NMR spectrum of compound **37** (^1^H: 500 MHz, ^13^C: 126 MHz, C_6_D_6_, 298 K).

Figure S291. ^1^H-^13^C-HMBC NMR spectrum of compound **37** (^1^H: 600 MHz, ^13^C: 151 MHz, C_6_D_6_, 298 K).

Figure S292. ^1^H-^13^C-HSQC NMR spectrum of compound **37** (^1^H: 600 MHz, ^13^C: 151 MHz, C_6_D_6_, 298 K).

Figure S293. ^1^H-^1^H-NOESY NMR spectrum of compound **37** (600 MHz, C_6_D_6_, 298 K).

#### Terpenoid 38

Figure S294. ^1^H NMR spectrum of compound **38** (600 MHz, C_6_D_6_, 298 K).

Figure S295. ^13^C NMR spectrum of compound **38** (151 MHz, C_6_D_6_, 298 K).

Figure S296. ^1^H-^1^H-COSY NMR spectrum of compound **38** (500 MHz, C_6_D_6_, 298 K).

Figure S297. ^1^H-^13^C-H2BC NMR spectrum of compound **38** (^1^H: 500 MHz, ^13^C: 126 MHz, C_6_D_6_, 298 K).

Figure S298. ^1^H-^13^C-HMBC NMR spectrum of compound **38** (^1^H: 600 MHz, ^13^C: 151 MHz, C_6_D_6_, 298 K).

Figure S299. ^1^H-^13^C-HSQC NMR spectrum of compound **38** (^1^H: 600 MHz, ^13^C: 151 MHz, C_6_D_6_, 298 K).

#### Terpenoid 49

Figure S300. ^1^H NMR spectrum of compound **49** (600 MHz, C_6_D_6_, 298 K).

Figure S301. ^13^C NMR spectrum of compound **49** (151 MHz, C_6_D_6_, 298 K).

Figure S302. ^1^H-^1^H-COSY NMR spectrum of compound **49** (500 MHz, C_6_D_6_, 298 K).

Figure S303. ^1^H-^13^C-H2BC NMR spectrum of compound **49** (^1^H: 500 MHz, ^13^C: 126 MHz, C_6_D_6_, 298 K).

Figure S304. ^1^H-^13^C-HMBC NMR spectrum of compound **49** (^1^H: 600 MHz, ^13^C: 151 MHz, C_6_D_6_, 298 K).

Figure S305. ^1^H-^13^C-HSQC NMR spectrum of compound **49** (^1^H: 600 MHz, ^13^C: 151 MHz, C_6_D_6_, 298 K).

Figure S306. ^1^H-^1^H-NOESY NMR spectrum of compound **49** (500 MHz, C_6_D_6_, 298 K).

#### Terpenoid 50

Figure S307. ^1^H NMR spectrum of compound **50** (400 MHz, C_6_D_6_, 298 K).

Figure S308. ^13^C NMR spectrum of compound **50** (101 MHz, C_6_D_6_, 298 K).

Figure S309. ^1^H-^1^H-COSY NMR spectrum of compound **50** (500 MHz, C_6_D_6_, 298 K).

Figure S310. ^1^H-^13^C-H2BC NMR spectrum of compound **50** (^1^H: 500 MHz, ^13^C: 126 MHz, C_6_D_6_, 298 K).

Figure S311. ^1^H-^13^C-HMBC NMR spectrum of compound **50** (^1^H: 400 MHz, ^13^C: 101 MHz, C_6_D_6_, 298 K).

Figure S312. ^1^H-^13^C-HMBC NMR spectrum of compound **50** with reduced area of measurement (^1^H: 500 MHz 4 ppm to 3 ppm, ^13^C: 126 MHz 150 ppm to 10 ppm , C_6_D_6_, 298 K).

Figure S313. ^1^H-^13^C-HSQC NMR spectrum of compound **50** (^1^H: 400 MHz, ^13^C: 101 MHz, C_6_D_6_, 298 K).

Figure S314. ^1^H-^1^H-NOESY NMR spectrum of compound **50** (500 MHz, C_6_D_6_, 298 K).

#### Terpenoid 51 as a mixture with compound 49

Figure S315. ^1^H NMR spectrum of compound **51** as a mixture with **49** (600 MHz, C_6_D_6_, 298 K).

Figure S316. ^13^C NMR spectrum of compound **51** as a mixture with **49** (151 MHz, C_6_D_6_, 298 K).

Figure S317. ^1^H-^1^H-COSY NMR spectrum of compound **51** as a mixture with **49** (600 MHz, C_6_D_6_, 298 K).

Figure S318. ^1^H-^13^C-H2BC NMR spectrum of compound **51** as a mixture with **49** - Correlations essential for the elucidation of **51** are highlighted (^1^H: 500 MHz, ^13^C: 126 MHz, C_6_D_6_, 298 K).

Figure S319. ^1^H-^13^C-HMBC NMR spectrum of compound **51** as a mixture with **49** - Correlations essential for the elucidation of **51** are highlighted (^1^H: 600 MHz, ^13^C: 151 MHz, C_6_D_6_, 298 K).

Figure S320. ^1^H-^13^C-HSQC NMR spectrum of compound **51** as a mixture with **49** - Correlations essential for the elucidation of **51** are highlighted (^1^H: 600 MHz, ^13^C: 151 MHz, C_6_D_6_, 298 K).

Figure S321. ^1^H-^1^H-NOESY NMR spectrum of compound **51** as a mixture with **49** - Correlations essential for the elucidation of **51** are highlighted (600 MHz, C_6_D_6_, 298 K).

#### Terpenoid 39

Figure S322. ^1^H NMR spectrum of compound **39** (400 MHz, C_6_D_6_, 298 K).

Figure S323. ^13^C NMR spectrum of compound **39** (101 MHz, C_6_D_6_, 298 K).

Figure S324. ^1^H-^1^H-COSY NMR spectrum of compound **39** (400 MHz, C_6_D_6_, 298 K).

Figure S325. ^1^H-^13^C-H2BC NMR spectrum of compound **39** (^1^H: 500 MHz, ^13^C: 126 MHz, C_6_D_6_, 298 K).

Figure S326. ^1^H-^13^C-HMBC NMR spectrum of compound **39** (^1^H: 400 MHz, ^13^C: 101 MHz, C_6_D_6_, 298 K).

Figure S327. ^1^H-^13^C-HSQC NMR spectrum of compound **39** (^1^H: 400 MHz, ^13^C: 101 MHz, C_6_D_6_, 298 K).

Figure S328. ^1^H-^1^H-NOESY NMR spectrum of compound **39** (400 MHz, C_6_D_6_, 298 K).

#### Terpenoid 40

Figure S329. ^1^H NMR spectrum of compound **40** (600 MHz, C_6_D_6_, 298 K).

Figure S330. ^13^C NMR spectrum of compound **40** (151 MHz, C_6_D_6_, 298 K).

Figure S331. ^1^H-^1^H-COSY NMR spectrum of compound **40** (600 MHz, C_6_D_6_, 298 K).

Figure S332. ^1^H-^13^C-H2BC NMR spectrum of compound **40** (^1^H: 500 MHz, ^13^C: 126 MHz, C_6_D_6_, 298 K).

Figure S333. ^1^H-^13^C-HMBC NMR spectrum of compound **40** (^1^H: 500 MHz, ^13^C: 126 MHz, C_6_D_6_, 298 K).

Figure S334. ^1^H-^13^C-HSQC NMR spectrum of compound **40** (^1^H: 600 MHz, ^13^C: 151 MHz, C_6_D_6_, 298 K).

Figure S335. ^1^H-^1^H-NOESY NMR spectrum of compound **40** (600 MHz, C_6_D_6_, 298 K).

#### Terpenoid 41 and 42

Figure S336. ^1^H NMR spectrum of compound **41** and **42** (600 MHz, C_6_D_6_, 298 K).

Figure S337. ^13^C NMR spectrum of compound **41** and **42** (151 MHz, C_6_D_6_, 298 K).

Figure S338. ^1^H-^1^H-COSY NMR spectrum of compound **41** and **42** (500 MHz, C_6_D_6_, 298 K).

Figure S339. ^1^H-^13^C-H2BC NMR spectrum of compound **41** and **42** (^1^H: 500 MHz, ^13^C: 126 MHz, C_6_D_6_, 298 K).

Figure S340. ^1^H-^13^C-HMBC NMR spectrum of compound **41** and **42** (^1^H: 500 MHz, ^13^C: 126 MHz, C_6_D_6_, 298 K).

Figure S341. ^1^H-^13^C-HSQC NMR spectrum of compound **41** and **42** (^1^H: 600 MHz, ^13^C: 151 MHz, C_6_D_6_, 298 K).

Figure S342. ^1^H-^13^C-HSQC NMR spectrum of compound **41** and **42** with reduced area of measurement (^1^H: 600 MHz 3 ppm to 0 ppm, ^13^C: 151 MHz 60 ppm to 0 ppm, C_6_D_6_, 298 K).

Figure S343. ^1^H-^1^H-NOESY NMR spectrum of compound **41** and **42** (400 MHz, C_6_D_6_, 298 K).

#### Terpenoid 43

Figure S344. ^1^H NMR spectrum of compound **43** (600 MHz, C_6_D_6_, 298 K).

Figure S345. ^13^C NMR spectrum of compound **43** (151 MHz, C_6_D_6_, 298 K).

Figure S346. ^1^H-^1^H-COSY NMR spectrum of compound **43** (600 MHz, C_6_D_6_, 298 K).

Figure S347. ^1^H-^13^C-H2BC NMR spectrum of compound **43** (^1^H: 500 MHz, ^13^C: 126 MHz, C_6_D_6_, 298 K).

Figure S348. ^1^H-^13^C-HMBC NMR spectrum of compound **43** (^1^H: 600 MHz, ^13^C: 151 MHz, C_6_D_6_, 298 K).

Figure S349. ^1^H-^13^C-HSQC NMR spectrum of compound **43** (^1^H: 600 MHz, ^13^C: 151 MHz, C_6_D_6_, 298 K).

Figure S350. ^1^H-^1^H-NOESY NMR spectrum of compound **43** (600 MHz, C_6_D_6_, 298 K).

#### Terpenoid 44

Figure S351. ^1^H NMR spectrum of compound **44** (600 MHz, C_6_D_6_, 298 K).

Figure S352. ^13^C NMR spectrum of compound **44** (151 MHz, C_6_D_6_, 298 K).

Figure S353. ^13^C NMR spectrum of compound **44** (151 MHz, CDCl_3_, 298 K).

Figure S354. ^1^H-^1^H-COSY NMR spectrum of compound **44** (600 MHz, C_6_D_6_, 298 K).

Figure S355. ^1^H-^13^C-H2BC NMR spectrum of compound **44** (^1^H: 500 MHz, ^13^C: 126 MHz, C_6_D_6_, 298 K).

Figure S356. ^1^H-^13^C-HMBC NMR spectrum of compound **44** (^1^H: 600 MHz, ^13^C: 151 MHz, C_6_D_6_, 298 K).

Figure S357. ^1^H-^13^C-HSQC NMR spectrum of compound **44** (^1^H: 600 MHz, ^13^C: 151 MHz, C_6_D_6_, 298 K).

Figure S358. ^1^H-^1^H-NOESY NMR spectrum of compound **44** (600 MHz, C_6_D_6_, 298 K).

# References

S1 Wang, C.-M.; Hopson, R.; Lin, X.; Cane, D. E. Biosynthesis of the Sesquiterpene Botrydial in *Botrytis Cinerea* . Mechanism and Stereochemistry of the Enzymatic Formation of Presilphiperfolan-8β-Ol. *J. Am. Chem. Soc.* **2009**, *131*, 8360–8361.

S2 Wawrzyn, G. T.; Quin, M. B.; Choudhary, S.; López-Gallego, F.; Schmidt-Dannert, C. Draft Genome of *Omphalotus Olearius* Provides a Predictive Framework for Sesquiterpenoid Natural Product Biosynthesis in Basidiomycota. *Chem. Bio.l* **2012**, *19*, 772–783.

S3 Cane, D. E.; Sohng, J.-K.; Lamberson, C. R.; Rudnicki, S. M.; Wu, Z.; Lloyd, M. D.; Oliver, J. S.; Hubbard, B. R. Pentalenene Synthase. Purification, Molecular Cloning, Sequencing, and High-Level Expression in *Escherichia Coli* of a Terpenoid Cyclase from Streptomyces UC5319. *Biochemistry* **1994**, *33*, 5846–5857.

S4 Lopez‐Gallego, F.; Agger, S. A.; Abate‐Pella, D.; Distefano, M. D.; Schmidt‐Dannert, C. Sesquiterpene Synthases Cop4 and Cop6 from *Coprinus Cinereus* : Catalytic Promiscuity and Cyclization of Farnesyl Pyrophosphate Geometric Isomers. *ChemBioChem* **2010**, *11*, 1093–1106.

S5 Back, K.; Chappell, J. Cloning and Bacterial Expression of a Sesquiterpene Cyclase from *Hyoscyamus Muticus* and Its Molecular Comparison to Related Terpene Cyclases. *J. Biol. Chem.* **1995**, *270*, 7375–7381.

S6 Hohn, T. M.; Beremand, P. D. Isolation and Nucleotide Sequence of a Sesquiterpene Cyclase Gene from the Trichothecene-Producing Fungus *Fusarium Sporotrichioides*. *Gene* **1989**, *79*, 131–138.

S7 Bentley, S. D.; Chater, K. F.; Cerdeño-Tárraga, A.-M.; Challis, G. L.; Thomson, N. R.; James, K. D.; Harris, D. E.; Quail, M. A.; Kieser, H.; Harper, D.; Bateman, A.; Brown, S.; Chandra, G.; Chen, C. W.; Collins, M.; Cronin, A.; Fraser, A.; Goble, A.; Hidalgo, J.; Hornsby, T.; Howarth, S.; Huang, C.-H.; Kieser, T.; Larke, L.; Murphy, L.; Oliver, K.; O’Neil, S.; Rabbinowitsch, E.; Rajandream, M.-A.; Rutherford, K.; Rutter, S.; Seeger, K.; Saunders, D.; Sharp, S.; Squares, R.; Squares, S.; Taylor, K.; Warren, T.; Wietzorrek, A.; Woodward, J.; Barrell, B. G.; Parkhill, J.; Hopwood, D. A. Complete Genome Sequence of the Model Actinomycete *Streptomyces Coelicolor* A3. *Nature* **2002**, *417*, 141–147.

S8 Tomato Genome Consortium. The Tomato Genome Sequence Provides Insights into Fleshy Fruit Evolution. *Nature* **2012**, *485*, 635.

S9 Yasuo, O.; Jun, I.; Hirofumi, H.; Hirokazu, S.; Miwa, I.; Haruo, I.; Atsushi, Y.; Masahira, H.; Sueharu, H. Genome Sequence of the Streptomycin-Producing Microorganism *Streptomyces Griseus* IFO 13350. *J. Bacteriol.* **2008**, *190*, 4050–4060.

S10 Cane, D. E.; Iyengar, R.; Shiao, M.-S. Cyclonerodiol Biosynthesis and the Enzymic Conversion of Farnesyl to Nerolidyl Pyrophosphate. *J. Am. Chem. Soc.* **1981**, *103*, 914–931.

S11 Cane, D. E.; Bowser, T. E. Trichodiene Synthase: Mechanism-Based Inhibition of a Sesquiterpene Cyclase. *Bioorg. Med. Chem. Lett.* **1999**, *9*, 1127–1132.

S12 Zhu, L.; Ni, C.; Zhao, Y.; Hu, J. 1-*tert*-Butyl-1*H*-Tetrazol-5-Yl Fluoromethyl Sulfone (TBTSO_2_CH_2_F): A Versatile Fluoromethylidene Synthon and Its Use in the Synthesis of Monofluorinated Alkenes via Julia–Kocienski Olefination. *Tetrahedron* **2010**, *66*, 5089–5100.

S13 Fürstner, A.; Aïssa, C. PtCl_2_-Catalyzed Rearrangement of Methylenecyclopropanes. *J. Am. Chem. Soc.* **2006**, *128*, 6306–6307.

S14 Oberhauser, C.; Harms, V.; Seidel, K.; Schröder, B.; Ekramzadeh, K.; Beutel, S.; Winkler, S.; Lauterbach, L.; Dickschat, J. S.; Kirschning, A. Exploiting the Synthetic Potential of Sesquiterpene Cyclases for Generating Unnatural Terpenoids. *Angew. Chem. Int. Ed.* **2018**, *57*, 11802–11806.

S15 Surendra, K.; Rajendar, G.; Corey, E. J. Useful Catalytic Enantioselective Cationic Double Annulation Reactions Initiated at an Internal π-Bond: Method and Applications. *J. Am. Chem. Soc.* **2014**, *136*, 642–645.

S16 Toullec, P. Y.; Blarre, T.; Michelet, V. Mimicking Polyolefin Carbocyclization Reactions: Gold-Catalyzed Intramolecular Phenoxycyclization of 1,5-Enynes. *Org. Lett.* **2009**, *11*, 2888–2891.

S17 Temple, K. J.; Wright, E. N.; Fierke, C. A.; Gibbs, R. A. Synthesis of Non-Natural, Frame-Shifted Isoprenoid Diphosphate Analogues. *Org. Lett.* **2016**, *18*, 6038–6041.

S18 Rabe, P.; Barra, L.; Rinkel, J.; Riclea, R.; Citron, C. A.; Klapschinski, T. A.; Janusko, A.; Dickschat, J. S. Conformational Analysis, Thermal Rearrangement, and EI‐MS Fragmentation Mechanism of (1(10)*E*, 4*E*,6*S*,7*R*)‐Germacradien‐6‐ol by ^13^C‐Labeling Experiments. *Angew. Chem. Int. Ed.* **2015**, *54*, 13448–13451.

S19 Li, C.; Wang, S.; Yin, X.; Guo, A.; Xie, K.; Chen, D.; Sui, S.; Han, Y.; Liu, J.; Chen, R. Functional Characterization and Cyclization Mechanism of a Diterpene Synthase Catalyzing the Skeleton Formation of Cephalotane‐Type Diterpenoids. *Angew. Chem. Int. Ed.* **2023**, *62*: e202306020.

S20 George, N. S.; Anderson, K. E.; Barrett, A. G. M. Total Synthesis of Cristatic Acid Based on Late‐Stage Decarboxylative Allylic Migration and Biomimetic Aromatization of a Diketo Dioxinone. *Eur. J. Org. Chem.* **2013**, *2013*, 7604–7610.

S21 Hoye, T. R.; Jeffrey, C. S.; Shao, F. Mosher Ester Analysis for the Determination of Absolute Configuration of Stereogenic (Chiral) Carbinol Carbons. *Nat. Protoc.* **2007**, *2*, 2451–2458.

S22 Sheldrick, G. M. *SHELXT* – Integrated Space-Group and Crystal-Structure Determination. *Acta Crystallogr. A Found. Adv.* 2015, *71*, 3–8.

S23 Dolomanov, O. V.; Bourhis, L. J.; Gildea, R. J.; Howard, J. A. K.; Puschmann, H. *OLEX2* : A Complete Structure Solution, Refinement and Analysis Program. *J. Appl. Crystallogr.* 2009, *42*, 339–341.

S24 Lübben, J.; Wandtke, C. M.; Hübschle, C. B.; Ruf, M.; Sheldrick, G. M.; Dittrich, B. Aspherical Scattering Factors for *SHELXL* – Model, Implementation and Application. *Acta Crystallogr. A Found. Adv.* 2019, *75*, 50–62.

S25 Xu, H.; Köllner, T. G.; Chen, F.; Dickschat, J. S. Mechanistic Characterisation of a Sesquiterpene Synthase for Asterisca-1,6-Diene from the Liverwort *Radula Lindenbergiana* and Implications for Pentalenene Biosynthesis. *Org. Biomol. Chem.* **2024**, *22*, 1360–1364.

S26 Fricke, C.; Hardt, I. H.; König, W. A.; Joulain, D.; Zygadlo, J. A.; Guzmàn, C. A. Sesquiterpenes from *Lippia Integrifolia* Essential Oil. *J. Nat. Prod.* **1999**, *62*, 694–696.

S27 Chemical Computing Group ULC: Montreal, Q. Chemical Computing Group ULC; Molecular Operating Environment (MOE), **2024**.

S28 Frisch, M. J. ea; Trucks, G. W.; Schlegel, H. B.; Scuseria, G. E.; Robb, Ma.; Cheeseman, J. R.; Scalmani, G.; Barone, V.; Petersson, G. A.; Nakatsuji, H. Gaussian 16. Gaussian, Inc. Wallingford, CT **2016**.

S29 Lou, T.; Li, A.; Xu, H.; Pan, J.; Xing, B.; Wu, R.; Dickschat, J. S.; Yang, D.; Ma, M. Structural Insights into Three Sesquiterpene Synthases for the Biosynthesis of Tricyclic Sesquiterpenes and Chemical Space Expansion by Structure-Based Mutagenesis. *J. Am. Chem. Soc.* **2023**, *145*, 8474–8485.

S30 Lesburg, C. A.; Zhai, G.; Cane, D. E.; Christianson, D. W. Crystal Structure of Pentalenene Synthase: Mechanistic Insights on Terpenoid Cyclization Reactions in Biology. *Science* **1997**, *277*, 1820–1824.

S31 Matos, J. O.; Kumar, R. P.; Ma, A. C.; Patterson, M.; Krauss, I. J.; Oprian, D. D. Mechanism Underlying Anti-Markovnikov Addition in the Reaction of Pentalenene Synthase. *Biochemistry* **2020**, *59*, 3271–3283.

S32 Krieger, E.; Koraimann, G.; Vriend, G. Increasing the Precision of Comparative Models with YASARA NOVA—a Self‐parameterizing Force Field. *Proteins: Struct. Funct. Bioinform.* **2002**, *47*, 393–402.

S33 Case, D. A.; Aktulga, H. M.; Belfon, K.; Ben-Shalom, I. Y.; Berryman, J. T.; Brozell, S. R.; Cerutti, D. S.; Cheatham, T. E., III; Cisneros, G. A.; Cruzeiro, V. W. D.; Darden, T. A.; Forouzesh, N.; Giambasu, G.; Giese, T.; Gilson, M. K.; Gohlke, H.; Goetz, A. W.; Harris, J.; Izadi, S.; Izmailov, S. A.; Kasavajhala, K.; Kaymak, M. C.; King, E.; Kovalenko, A.; Kurtzman, T.; Lee, T.; Li, P.; Lin, C.; Liu, J.; Luchko, T.; Luo, R.; Machado, M.; Man, V.; Manathunga, M.; Merz, K. M.; Miao, Y.; Mikhailovskii, O.; Monard, G.; Nguyen, H.; O’Hearn, K. A.; Onufriev, A.; Pan, F.; Pantano, S.; Qi, R.; Rahnamoun, A.; Roe, D. R.; Roitberg, A.; Sagui, C.; Schott-Verdugo, S.; Shajan, A.; Shen, J.; Simmerling, C. L.; Skrynnikov, N. R.; Smith, J.; Swails, J.; Walker, R. C.; Wang, J.; Wang, J.; Wei, H.; Wu, X.; Xiong, Y.; Xue, Y.; York, D. M.; Zhao, S.; Zhu, Q.; Kollman, P. A. *Amber 2023*; University of California, San Francisco, **2023**.

S34 Tian, C.; Kasavajhala, K.; Belfon, K. a. A.; Raguette, L.; Huang, H.; Migues, A. N.; Bickel, J.; Wang, Y.; Pincay, J.; Wu, Q.; Simmerling, C. ff19SB: Amino-Acid-Specific Protein Backbone Parameters Trained against Quantum Mechanics Energy Surfaces in Solution. *J. Chem. Theory Comput.* **2019**, *16* (1), 528–552.

S35 Deserno, M.; Holm, C. How to mesh up Ewald sums. I. A theoretical and numerical comparison of various particle mesh routines. *J. Chem. Phys.* **1998**, *109* (18), 7678–7693.

S36 Davidchack, R. L.; Ouldridge, T. E.; Tretyakov, M. V. New Langevin and gradient thermostats for rigid body dynamics. *J. Chem. Phys.* **2015**, *142* (14), 144114.

S37 Bresme, F. Equilibrium and nonequilibrium molecular-dynamics simulations of the central force model of water. *J. Chem. Phys.* **2001**, *115* (16), 7564–7574.

S38 Ryckaert, J.-P.; Ciccotti, G.; Berendsen, H. J. C. Numerical integration of the cartesian equations of motion of a system with constraints: molecular dynamics of n-alkanes. *J. Comput. Phys.* **1977**, *23* (3), 327–341.

S39 Roe, D. R.; Cheatham, T. E. PTRAJ and CPPTRAJ: Software for processing and analysis of Molecular Dynamics Trajectory data. *J. Chem. Theory Comput.* **2013**, *9* (7), 3084–3095.

S40 Humphrey, W.; Dalke, A.; Schulten, K. VMD: Visual molecular dynamics. *J. Mol. Graph.* **1996**, *14* (1), 33–38.
